# Identification of gene-drug interactions that impact patient survival in TCGA

**DOI:** 10.1186/s12859-016-1255-7

**Published:** 2016-10-06

**Authors:** John Christian Givhan Spainhour, Peng Qiu

**Affiliations:** Department of Biomedical Engineering, Georgia Institute of Technology and Emory University, 313 Ferst Dr. NW, Atlanta, GA 30332 USA

**Keywords:** TCGA, Survival, Gene-drug interaction

## Abstract

**Background:**

With the advent of large scale biological data collection for various diseases, data analysis pipelines and workflows need to be established to build frameworks for integrative analysis. Here the authors present a pipeline for identifying disease specific gene-drug interactions using CNV (Copy Number Variation) and clinical data from the TCGA (The Cancer Genome Atlas) project. Two cancer types were selected for analysis, LGG (Brain lower grade glioma) and GBM (Glioblastoma multiforme), due to the possible progression from LGG to GBM in some cases. The copy number and clinical data were then used to preform survival analysis on a gene by gene basis on sub-populations of patients exposed to a given drug.

**Results:**

Several gene-drug interactions are identified, where the copy number of a gene is associated to survival of a patient exposed to a certain drug. Both Irinotecan/HAS2 (Hyaluronan synthase 2) and Bevacizumab/PGAM1 (Phosphoglycerate mutase 1) are interactions found in this study with independent confirmation. Independent work in colon, breast cancer and leukemia (Györffy, Breast Cancer Res Treat 123:725-731, 2010; Mueller, Mol Cancer Ther 11:3024–3032, 2010; Hitosugi, Cancer Cell 13:585-600, 2012) showed these two interactions can lead to increased survival.

**Conclusion:**

While the pipeline produced several possible interactions where increased survival is linked to normal or increased copy number of a given gene for patients treated with a given drug, no instance of low copy number or full deletion was linked to increased survival. The development of this pipeline shows a promising utility to identify possible beneficial gene-drug interactions that could improve patient survival and may illustrate some of the problems inherent in this kind of analysis on these data.

**Electronic supplementary material:**

The online version of this article (doi:10.1186/s12859-016-1255-7) contains supplementary material, which is available to authorized users.

## Background

The analysis of large scale biological data has multiple challenges including noise filtering and data integration [1, 2] but can provide fruitful queries into questions about various diseases. Here a pipeline is implemented for integrative analysis of CNV data, drug treatment and survival data, for the purpose of identifying beneficial gene-drug interactions, where the copy number of a gene is associated to survival of patients exposed to a certain drug. The pipeline is applied to data from LGG and GBM cancer patients in the TCGA database as an example and results are presented. The use of omics data in survival analysis has been previously shown to help answer questions at the genetic level in cancer survival [3]. However, that analysis is not without its difficulties. Variation in levels of gene expression between patient profiles and obtaining the necessary number of patients for sufficient statistical power are just a few of the hurdles that need to be addressed with any study of this nature.

TCGA provides a large and robust data set for the analysis of multiple diseases [4–7]. Survival analysis is an excellent tool for the identification of various traits or treatments that are predictive of patient survival. The combination of genomic and proteomic features with patient information provides an excellent resource for predicting patient survival [8]. Previous studies have performed extensive analysis to identify biomarkers predictive of survival. Those survival analyses typically focused on survival data and genomic features, but did not provide any treatment-specific insights. The identification of treatment-specific survival predictors would be a useful step toward personalized medicine. Treatment-specific survival prediction can be accomplished by combining genomic, drug, and survival data from TCGA, stratifying patients into treatment groups and perform survival analysis for each separately. This introduces several challenges since drug data contains alternating names of drugs, misspellings, and other confusing information.

The use of CNV has different applications in survival analysis when compared to the use of expression levels. While gene copy number, RNA expression and protein expression can be related, it is usually not a linear relationship [9, 10]. These are various ways used to represent the level of activity a gene may have in a biological system. CNV has intrinsic cutoffs in the form of fewer number of gene copies (one or zero), healthy or normal number of copies (two for a typical gene) and more copies (three plus). This allows for easy categorization for survival analysis using a physical change in the genome of the cancer. The use of CNV does not preclude the use of other information but can serve as a way to identify genes of interest using survival analysis before looking at other omics data. LGG and GBM were selected for analysis since it is believed that they may share some common genetic elements and it has been proposed that LGG may lead to GBM in some instances [11]. This connection may allow for the pooling of the LGG and GBM patients to increase the statistical power of the survival analysis and detect genes that may otherwise be missed. GBM has been characterized in the past by members of the TCGA network [4–7, 12, 13] and analyses have yielded novel results such as the correlation between GBM subtypes and expression of PDGFRA, IDH1, EGFR and NF1 [12] and the identification of the CpG methylator phenotype [5].

**Fig. 1:**
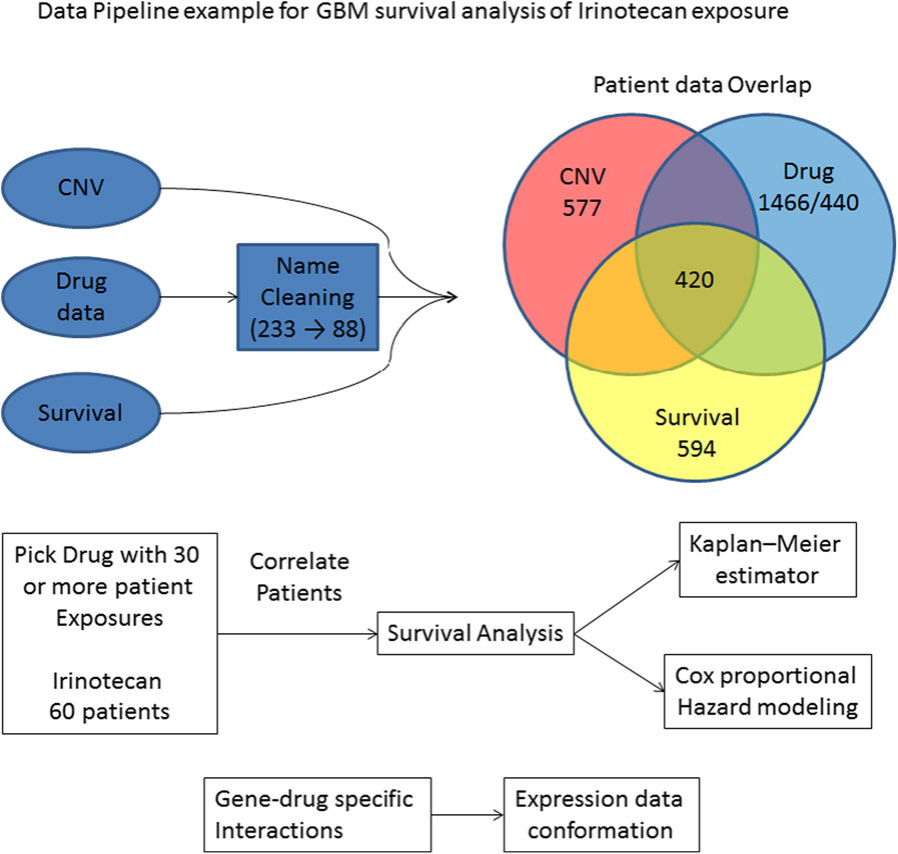
Data processing pipeline. A simple outline of the data processing pipeline for searching TCGA data for gene-drug interactions in individual and pooled cancer types

Our pipeline (Fig. 1) starts with using the CNV, survival and drug treatment TCGA data, which involves an amount of data annotation and cleaning drug names. Cleaning drug names is key since the objective of the pipeline is to preform drug-specific survival analysis for gene-drug interactions. The number of patients exposed to a given drug varies, as shown in Table 1. In our analysis, we only considered drugs with more than 30 patients exposed in the LGG and GBM data in TCGA. For a given drug, all LGG and GBM patients exposed to the drug are selected for analysis. Survival analysis is preformed to correlate copy number of each gene and survival data using Kaplan–Meier curves and Cox proportional hazard modeling. Because the number of patients exposed to a given drug is small, we performed two survival analyses, and identified genes that are significant in both analyses. Since we combined LGG and GBM patients in our analysis and two cancers have different survival, cancer type becomes a confounding factor. To avoid this confounding factor, a fisher test is preformed to genes identified in both survival analyses. The genes whose copy numbers show significant correlation with cancer type are filtered out. Finally, expression data can be used as one form of validation, examining whether the drug-specific correlation between copy number and survival also manifests at the gene expression level. This data is not available for all genes that have copy number data in TCGA, but can be a potent parallel of a proposed gene-drug interaction.

**Table 1:** Patient drug exposure summary

Patient numbers			
Drug	LGG	GBM	LGG + GBM
Temozolomide	241	239	580
Bevacizumab	41	92	133
Lomustine	32	49	84
Irinotecan	16	60	76
Dexamethasone	0	60	60
Carmustine	12	39	51
Gliadel Wafer	5	35	40
Etoposide	10	28	38
Procarbazine	17	22	39

Total numbers of patients in each cancer type who have been exposed to each drug. Only drugs with 30 or more total patients exposed between both cancer types are shown

This multistep analysis pipeline serves the dual purpose of looking for gene-drug interactions and as a way to look for similarities between multiple cancer types. The pooling of cancer types allows for the possible enrichment of signals in the data when the cancer types appear to be related. Many genes significant in the pooled analysis appear near but do not cross the threshold for significance in the individual analysis of LGG and GBM separately, and few genes found to be significant in the individual analyses were lost in the pooled analyses.

## Methods

### Data cleaning and stratification by drug exposure

When dealing with drug exposure data from TCGA sources, there are several nomenclature problems that must be dealt with. The drugs listed in TCGA can be recorded by one of many names, an ID number, an abbreviation or an associated study. These identifiers also contain misspellings of these names or miss-entries (0 instead of o or 6 instead of G). These errors make it impossible to use drug exposure data systematically in an integrative omics analysis. The drug data requires cleaning and standardization. In this paper, all data cleaning was performed by referencing the NCI Drug dictionary [14] (2015 accession) and Broad GDAC Firehose [15]. The NCI Drug dictionary was queried for each entry and corrections or standardization to the preferred name of the drug.

Once the drug names were standardized, patients were grouped by drug exposure. This stratification allows us to use copy number data in survival analysis to identify genes that predict survival in a drug-specific way. These identified genes may not correlate with survival when the survival analysis is applied to the entire set of all patients. Hence, patient stratification by drug exposure allows the proposed analysis to identify gene-drug interactions that impact patient survival.

### Survival analysis

Survival analysis was performed using a standard Kaplan–Meier curve with a Bonferroni correction to *p*-values based on the number of gene clusters with identical CNV patterns. Secondary analysis using a standard Cox proportional hazard model without any *p*-value correction was used on the same gene clusters. A standard acceptance cutoff for *p* < =.05 was used. Fisher test was used as a filtering step to prevent possible bias in the analysis due to the pooling of two cancer types. The fisher test was performed by cancer type and CNV, forming a 2x3 fisher’s matrix to filter out genes whose copy numbers are significantly different in the two cancer types. All statistical tests used standard R [16] functions without editing.

### Software

All software for analysis was written in an R [16] environment and code samples are available in the Additional file 1 associated with this paper. The survival package supplied in the base R environment was used for all survival analysis.

## Results

### Data preprocessing

Drug data consisted of multiple entries per patient of each drug they were given. These drugs are listed in a semi-temporal (by date of entry into the database) order but many drugs appear in multiple names. This required manually cleaning the drug names using the NCI [14] drug dictionary database as a reference. After drug data was cleaned to remove multiple names for drugs in a given cancer type, patients were stratified by drug exposure. Multiple drug exposures were treated as independent for the purposes of this analysis, a patient would be listed separately for each drug they were exposed to regardless of order, number or length of treatments. During cleaning of drug names, certain combinations of drugs such as Procarbazine, Lomustine, and Vincristine were treated as a single drug treatment since they are usually prescribe together and listed under one heading (PVC) in the data. These combinations of drugs did not play an important role because the numbers of patients exposed to the combinations were too small to perform survival analysis.

Data cleaning changed the names of the 279 unique differently named drug exposures in the data down to 100 unique drug treatments in the LGG and GBM patients. The number of patients exposed to each drug examined can be found in Table 1. The number of patients for each drug is a small portion of the total pool of patients, and for many drugs too small for survival analysis in an individual cancer. By pooling the LGG and GBM patient, we were able to increase the number of patients with a given drug treatment to perform an analysis on.

GDAC [15] CNV data was used for this analysis where GISTIC2.0 [17] was used on raw TCGA data with amplification and deletion cutoffs of 0.1 on a log base 2 scale, such that no copy number change is 0, genes with amplifications have positive values (1, 2), and genes with deletions have negative values (-1 or -2). This CNV data was accessed in April of 2015, as was the survival and drug treatment data from the TCGA data portal.

### Analysis of gene-drug interactions

Drugs with 30 or more patients were considered in our analysis. For each drug a separate analysis was performed, focusing on patients exposed to that drug. CNV data was used to place patients into three categories of CNV based on full or partial deletion, no change in copy number, or increased copy number. Kaplan-Meier analysis and cox proportional hazard modeling were performed using death or last visit time for a censored date using standard R [16] functions. Both of these tests were subject to *p* < = .05 significance.

Separate analysis of the individual cancer types provided promising gene-drug interactions in LGG and GBM separately. However, due to the above minimum required number of patients (Table 1), there were no results that could be compared between the two cancer types (Table 2, colm. 1&2).

**Table 2:** Genes found through analysis

Genes							
Drug	LGG	GBM	LGG + GBM	Shared significant genes	Fisher test confirmed (p-sig means reject)	Expression data	Genes
Temozolomide	2914	0	5072	2531	0	0	
Bevacizumab	1050	0	611	232	8	7	MIR607, LCOR, MMS19, SFXN2, EXOSC1, ZDHHC16, C10orf12, PGAM1
Lomustine	0	0	9	0	0	0	
Irinotecan	NA	404	97	78	1	1	HAS2
Dexamethasone	NA	0	0	0	0	0	
Carmustine	NA	0	7	0	0	0	
Gliadel Wafer	NA	0	0	0	0	0	
Etoposide	NA	396	142	115	115	89	See Additional file 1
Procarbazine	NA	NA	8	0	0	0	

The number of genes found to be significant at each step of the pipeline are shown. The first two columns show number of identified genes when LGG and GBM were analyzed separately. NA entries represent combinations of cancer and drug that could not be analyzed because the number of available patients was below our threshold. The zero entries in the table represent that no significant genes were found at the given stage of the analysis. Third column shows the number of genes identified in the pooled analysis. Column four shows the number of genes shared between individual and pooled analysis. Column five shows the application of a fisher test to excluded genes differentially expressed between cancer types, and hence were artificially significant because of pooling. The final columns show the number and names of the genes with available expression data, out of the genes found significant up to this point in the pipeline

Pooling of LGG and GBM patient data provided a more robust data set and a larger number of patients for each drug. The pooled data was analyzed as stated above to identify gene-drug interactions (Table 2, column 3). The overlap between the pooled and individual analyses was shown in Table 2 column 4. Since LGG and GBM patients in general have different survival, any gene whose copy number differs between the two cancers would show up as significant in the pooled survival analysis. To remove the artificially significant genes by pooling, a 2x3 Fisher’s test (cancer type by gene CNV) on the overlapping genes was performed, to identify and remove genes whose copy numbers show significant difference between LGG and GBM. Finally the gene-drug interactions identified were reported (Table 2). Additionally, expression data for the genes of interest can be examined with boxplots and *t*-test to confirm the identified gene-drug interactions at gene expression level. Across LGG and GBM patients, 128 genes were found to have survival impact when combined with three drugs (Table 2). Ninety-seven of these genes had expression data that could be examined as part of the follow up analysis.

### HAS2-irienotecan interaction

**Fig. 2:**
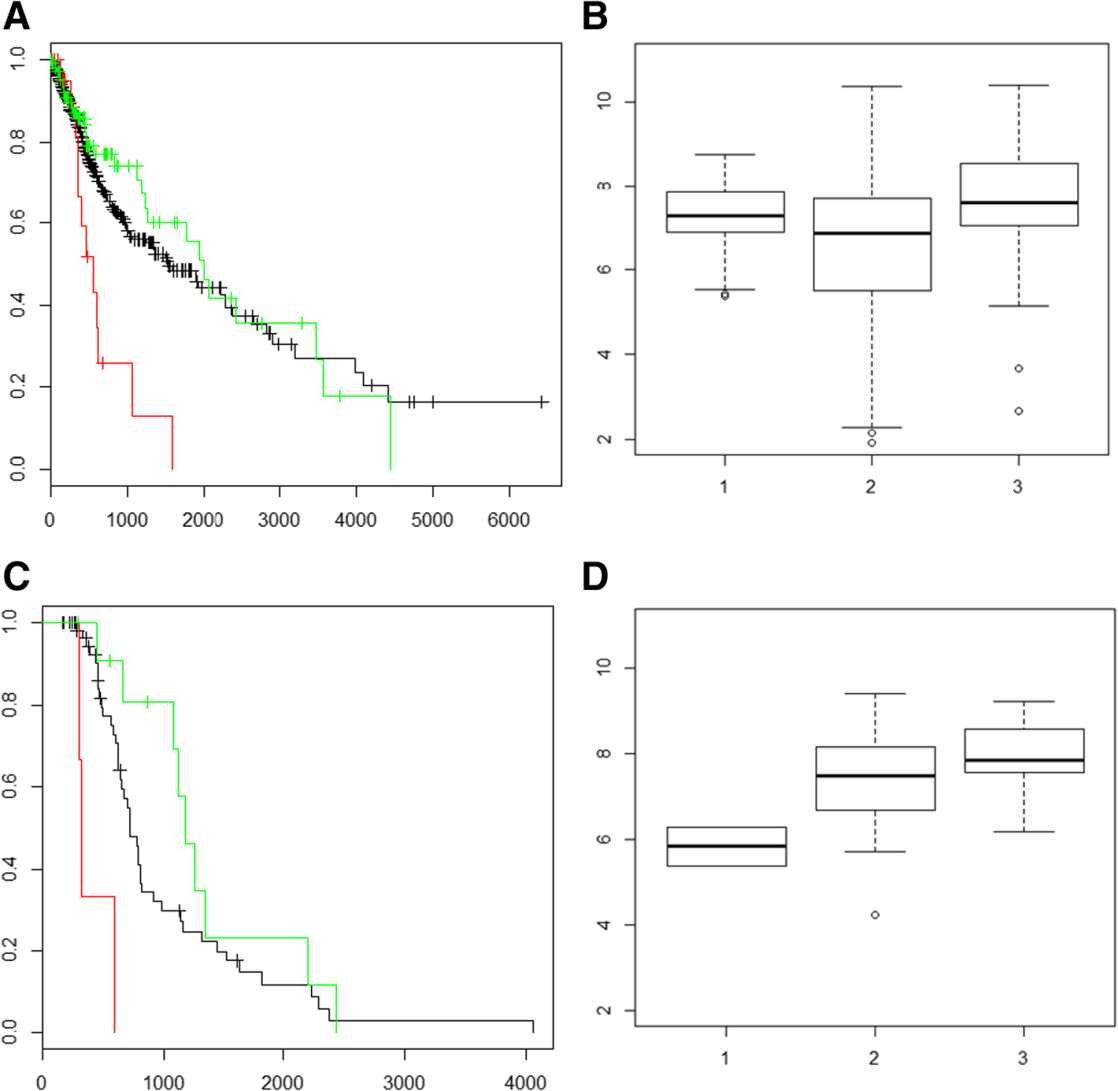
HAS2-Irinotecan Survival Analysis. **a** Survival of HAS2 in pooled LGG + GBM, *n* = 645. KM survival analysis of pooled LGG and GBM patients for HAS2 CNV. Green illustrates increased CNV while red shows decreased CNV and black shows survival for patients with normal copy number. **b** HAS2 expression and CNV for LGG + GBM, *n* = 645. Expression values of HAS2 by CNV where group 1 has decreased copy number, group 2 has a normal copy number of HAS2 and group 3 has an increased copy number. There is no statistical difference by *t*-test between these groups when all LGG and GBM patients are examined. This does not take into account drug exposure. Expression in log 2 (x + 1) form. **c** Survival analysis of HAS2 CNV and Irinotecan exposure in pooled LGG and GBM patients, *n* = 76. KM survival curve of LGG and GBM pooled patients exposed to Irinotecan. Green illustrates increased copy number while red shows decreased copy number and black shows survival for patients with normal copy number. Decreased copy number of HAS2 has a distinct decrease in survival when Irinotecan exposure is considered. **d** HAS2 expression and CNV for pooled LGG + GBM, Irinotecan Exposure, *n* = 29. Expression values of HAS2 in pooled LGG and GBM patients by CNV where group 1 has decreased copy number, group 2 has a normal copy number of HAS2 and group 3 has an increased copy number. This plot is derived from patients who have expression, CNV, drug and survival data recorded in TCGA. There is a statistical difference in expression (*p* = .045) between group 1 and group 3, with a close to significant (*p* = .11) difference between group 1 and group 2

Following one identified gene-drug interaction, HAS2 and Irinotecan, shows that among patients treated with Irinotecan, normal or elevated copy number of HAS2 correlates with increased survival. This example illustrates how the drug-specific focus on survival analysis of gene-drug interactions can highlight possible beneficial therapeutic strategies. Looking at all LGG and GBM patients, the Kaplan–Meier curves show no difference between patients with normal and elevated copy number of HAS2, and a marked decrease in survival in patients with low copy number of HAS2 (Fig. 2a). No difference in expression can be seen among patients with different copy numbers of HAS2 (Fig. 2b). The survival difference seen between LGG and GBM patients with a loss of HAS2 and normal copy number is not supported by a corresponding difference in HAS2 expression in the same patients. These discrepancies require a narrowing focus for this type of analysis. When focusing on patients exposed to Irinotecan, survival analysis through the lens of drug treatment presents a clearer picture. The loss of HAS2 and exposure to Irinotecan showed a marked and clear decrease in survival (Fig. 2c). The difference between normal and increased copy number, while not statistically significant (*p* = .067), is far more clearly defined than in the first analysis (Fig. 2a). Compared to Fig. 2b where no expression difference was observed among patients with different copy number of HAS2, when focusing on patients exposed to Irinotecan in Fig. 2d, we observed a significant (*p* = .045) expression difference between the low and normal/increased copy number patients. This shows that the HAS2-Irinotecan interaction identified based on copy number also manifests at the gene expression level. This analysis was also performed for LGG and GBM separately, but did not reveal significant interactions due to the small numbers of patients exposed to this drug. Being drug-specific and pooling of LGG and GBM has the combined effect of highlighting gene drug interactions of interest and providing more patients to power the detection of these important interactions.

A review of the literature after this interaction was identified showed that there has already been some independent investigation of this interaction. Irinotecan is topoisomerase inhibitor that in inhibits cell division and has been shown to repress tumor growth in cell lines with HAS2 over production. The previous analysis of this interaction was performed in a mouse xenograft model [8] and it was suggested that Irinotecan inhibited tumor regrowth after chemotherapy. Hyaluronan is believed to act as a carrier molecule for Irinotecan improving its delivery to tumor cells. This interaction has been studied in clinical trials and has shown promise in treating colorectal cancer [18]. Here our method has identified an interaction that can be verified from an independent source.

### PGAM1-bevacizumab interaction

Our analysis identified another gene-drug interaction, PGAM1 and Bevacizumab, which also has literature support. PGAM1 is an enzyme that plays a role in allowing the cell to balance glycolysis and biosynthesis [19]. Bevacizumab is an anti-vascular endothelial cell growth factor antibody that is used to restrict the growth of new blood vessels causing hypoxia in tumors. This interaction was explored in cell lines and the loss of PGAM1 was shown to decrease the effects of hypoxia on the tumor by inhibiting the ability of the cell to regulate balance between glycolysis and biosynthesis allowing tumor growth that would be inhibited in a hypoxic state [20]. Figures can be found in the Additional file 2.

### COL22A1-etoposide interaction

**Fig. 3:**
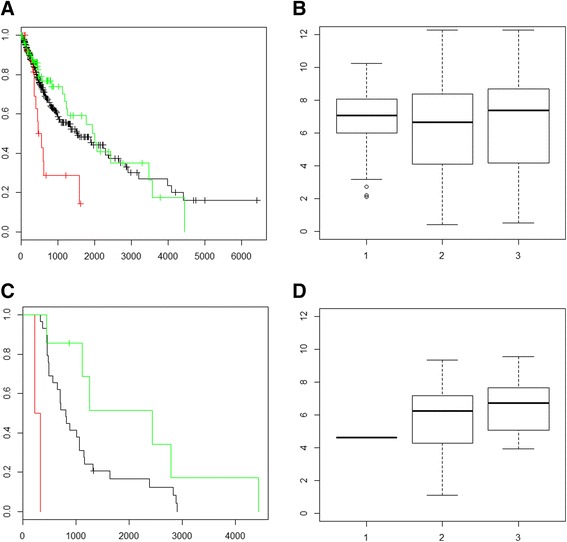
COL22A1- Etoposide Survival Analysis. **a** COL22A1 expression and CNV for LGG + GBM, *n* = 645. KM survival analysis of pooled LGG and GBM patients for COL22A1 CNV. Green illustrates increased CNV while red shows decreased CNV and black shows survival for patients with normal copy number. **b** COL22A1 expression and CNV for LGG + GBM, *n* = 645. Expression values of COL22A1 by CNV where group 1 has decreased copy number, group 2 has a normal copy number of COL22A1 and group 3 has an increased copy number. There is no statistical difference by *t*-test between these groups when all LGG and GBM patients are examined. This does not take into account drug exposure. Expression in log 2 (x + 1) form. **c** Survival analysis of COL22A1 CNV and Etoposide exposure in pooled LGG and GBM patients, *n* = 34. KM survival curve of LGG and GBM pooled patients exposed to Irinotecan. Green illustrates increased copy number while red shows decreased copy number and black shows survival for patients with normal copy number. Decreased copy number of COL22A1 has a distinct decrease in survival when Irinotecan exposure is considered. **d** COL22A1 expression and CNV for pooled LGG + GBM, Etoposide Exposure, *n* = 16. Expression values of COL22A1 in pooled LGG and GBM patients by CNV where group 1 has decreased copy number, group 2 has a normal copy number of COL22A1 and group 3 has an increased copy number. This is derived from patients who have expression, CNV, drug and survival data recorded in TCGA

An instance of an unknown gene-drug interaction, that of COL22A1 and Etoposide, can be seen in Fig. 3. While loss of COL22A1 copy number can be linked to lowered survival over the entire pool of patients (Fig. 3a), patients with different COL22A1 copy number do not show significant difference in COL22A1 expression (Fig. 3b). Similar to the HAS2 example, the correlation between COL22A1 and survival is not supported by gene expression when all LGG and GBM patients are considered. The real difference in expression that aids in validation of the survival analysis is highlighted when only patients with Etoposide exposure are considered. While the number of patients for this analysis is lower than that in previous examples, there is still a difference between survival and copy number, which is mirrored in gene expression levels (Fig. 3c & d).

There is no current research in the literature that shows a possible link between COL22A1 and Etoposide. Etoposide is a topoisomerase inhibitor and COL22A1 is a collagen protein that acts as a cell adhesion ligand for skin epithelial cells and fibroblasts [21]. COL22A1 upregulation has been linked to prognosis in head and neck cancers [22]. Here we have identified an interaction that has not been explored in the existing literature.

## Discussion

This method of survival analysis using drug exposure to highlight gene drug interactions for various cancer types shows promise. We are able to focus this analysis by looking at small sub populations of patients exposed to a particular drug, preform survival analysis using gene copy number variation, and look for significant differences in survival. Once these differences are identified, expression data can be used to further confirm a biological basis for the proposed gene-drug interaction. This method has shown confirmation of the HAS2/Irinotecan link in survival which has been shown previously in the literature [8] in different cancers. This method has also produced several interesting drug and gene combinations (Table 2) that can be examined experimentally for possible strategies for personalized medicine.

## Conclusion

This data processing pipeline is far from perfect. This multiple layer filtering process removed many individuals from the patient pool at each stage of the analysis, giving a limited number of patients for any given cancer/gene-drug combination. The currently available data collected in projects such as TCGA is an excellent start. Future queries along this idea of drug-specific analysis need to address the hurdles of formalizing drug exposure by name, and loss of information such as different parts of patient omics and clinical data (CNV, expression, drug exposure, survival). More data of higher quality will be needed in the foreseeable future for accurate analyses of this type. The pipeline itself can be improved by exploring other possible methods for examining survival and alternative strategies for *p*-value correction and reordering. Methods for incorporating expression data in a more direct manner might also improve the identification of gene-drug interaction. Exploring methods to account for drug treatment order and removing the assumption that drug treatments are independent would help illustrate synergistic or antagonistic effects of multiple drug treatments.

During our analysis, one interesting and unexpected pattern was seen. For all the identified gene-drug interactions, increased survival was always associated to normal or increased copy number. No increase in survival was seen in the case of a gene deletion or decrease in copy number. The reasons for this could be multiform. Current trends in cancer treatment focus on inhibiting oncogenes, inhibiting the increased activity from these genes or promoting apoptosis. Therefore, for patients with increased copy number or expression of a target gene, the drug is more likely to show an effect. For a patient with a deletion, the drug is less likely to show strong effect. This is a possible explanation as to why all identified gene-drug interactions have the same survival pattern where deletion lowers survival.

## Additional files

Additional file 1: This document contains descriptions of all material located in Additional file 2 as well as descriptions of how to access data used in this analysis. A more indepth description of the PGAM1-Bevacizumab interaction is also included. (DOCX 59 kb)
Additional file 2: This file contains additional figures, sample code, a gene list for table 2 and a drug name change list. (ZIP 1090 kb)

## Abbreviations

CNV: Copy number variation
COL22A1: Collagen, type XXII, alpha-1
GBM: Glioblastoma multiforme
HAS2: Hyaluronan synthase 2
LGG: Brain lower grade glioma
NCI: National cancer institute
PGAM1: Phosphoglycerate mutase 1
TCGA: The Cancer Genome Atlas
