# Supplementary material for: Identification of gene-drug interactions that impact patient survival in TCGA
Source: BMC Bioinformatics. 2016 Oct 6;17:409. doi: 10.1186/s12859-016-1255-7 (PMC5053348; doi:10.1186/s12859-016-1255-7)
Supplement: Additional file 1: — This document contains descriptions of all material located in Additional file 2 as well as descriptions of how to access data used in this analysis. A more indepth description of the PGAM1-Bevacizumab interaction is also included. (DOCX 59 kb) [file 12859_2016_1255_MOESM1_ESM.docx]

**Supplementary Information**

**Supplemental Figures:**

These figures show one gene per page, following the format of figures 2 and 3 in the main manuscript, for each gene found in the analysis. Due to the limited number of patients from the stringent filtering process used in this analysis, many genes shared the same copy number profile. This identical copy number profile leads to identical results for the survival analysis of many of the genes examined. We refer to these sets of genes with identical copy numbers as copy number segments. The segments are further compounded by the simplification of copy number data into three categories; this was done since the number of available patients was not great enough to support more than a three-group survival analysis. These segments can reflect genome regions that have been lost or amplified because the GDAC GISTIC 2.0 analysis results used to supply copy number arranged the genes by cytoband location.

Due to the adherence to a stringent p-value < .05 along with the overlap of the two methods of survival analysis, the number of significant gene segments is greatly reduced. This is further reduced by the Fisher’s test filtering step. The end result is that for each drug used in the analysis, a single copy number segment was found to be significant. This is reflected in the KM survival curves seen in the supplemental figures, and a detailed list of genes in each segment can be found in Table 2 and the Gene list supplemental document.

**Gene List:**

A list of the genes found to be significant in the Etoposide drug exposure analysis, a supplementary list for table 2.

**Drug List:**

The list of drugs used in the analysis. The left column designates the drug as called in our analysis. The right column shows each drug as listed in the TCGA data, grouped by our data cleaning.

**Sample R code:**

Sample one contains an example of the analysis and filtering preformed. Sample two shows how the figures were generated.

**Data:**

Data Sets mentioned in the sample R code are simple combinations of the individual *nationwidechildrens.org_clinical_drug.csv, *nationwidechildrens.org_clinical_patient.csv, and *all_thresholded.by_genes.csv files for each cancer type available from TCGA and firebrowse, which checks to make sure row and column data placement is consistent. These files cannot be included in the supplemental information since they exceed the file size allowed in supplemental Information. Instructions of how to obtain these files are in the following subsection. The authors will also make these files available upon request.

**Data Acquisition:**

Data files can be obtained by using the GDAC fisehose site or though the TCGA data portal. For example: Go to <http://gdac.broadinstitute.org/>. Select cancer and click on Browse under Analysis column. Click on CopyNumber Analyses and select CopyNumber Gistic2. In the provided window scroll down to Methods, click, and the click on Output. Scroll down to Download Results and select Analysis results to download the compressed file that contains the “all_thresholded.by_genes.csv” file.

**PGAM1-Bevacizumab Interaction:**

The survival of patients with a normal copy number PGAM1 is greater in both patient groups (all patients in the upper left plot, and patients exposed to Bevacizumab in the bottom left). The P-values are similar (Bonferroni corrected .047 vs. .049 respectively). Compare to the group with all patients (374), the number of patients in the Bevacizumab exposed group is much smaller (65), yet achieving similar p-value, which is an indication of possible interaction between PGAM1 and Bevacizmuab. The expression data is inconclusive, we do not see a statistically significant difference in expression between low and normal copy number groups in either the total patient population or the Bevacizumab exposed population.

PGAM1 is an enzyme that plays a role in allowing cells to balance glycolysis and biosynthesis. Bevacizumab is an anti-vascular endothelial cell growth factor antibody that is used to restrict the growth of new blood vessels causing hypoxia in tumors. The loss of PGAM1 decreases the effects of hypoxia on the tumor by inhibiting the ability of the cell to regulate balance between glycolysis and biosynthesis, allowing tumor growth that would be inhibited in a hypoxic state.
