# Supplementary material for: Identification of gene-drug interactions that impact patient survival in TCGA
Source: BMC Bioinformatics. 2016 Oct 6;17:409. doi: 10.1186/s12859-016-1255-7 (PMC5053348; doi:10.1186/s12859-016-1255-7)
Supplement: Additional file 2: — This file contains additional figures, sample code, a gene list for table 2 and a drug name change list. (ZIP 1090 kb) [file 12859_2016_1255_MOESM2_ESM.zip › Supplemental.pdf]

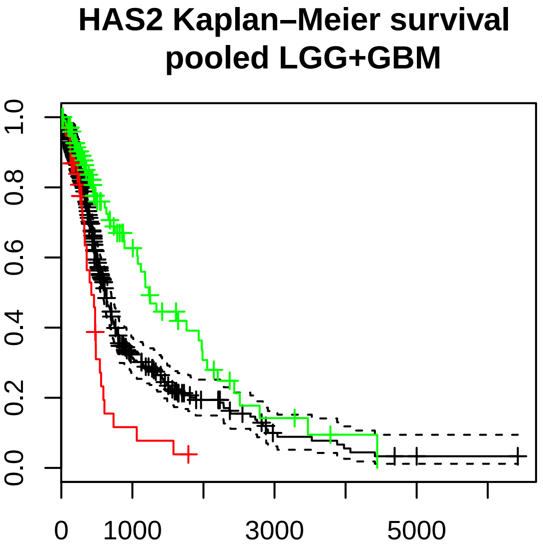
HAS2 Kaplan–Meier survival
pooled LGG+GBM
0.0
0.2
0.4
0.6
0.8
1.0
0
1000
3000
5000

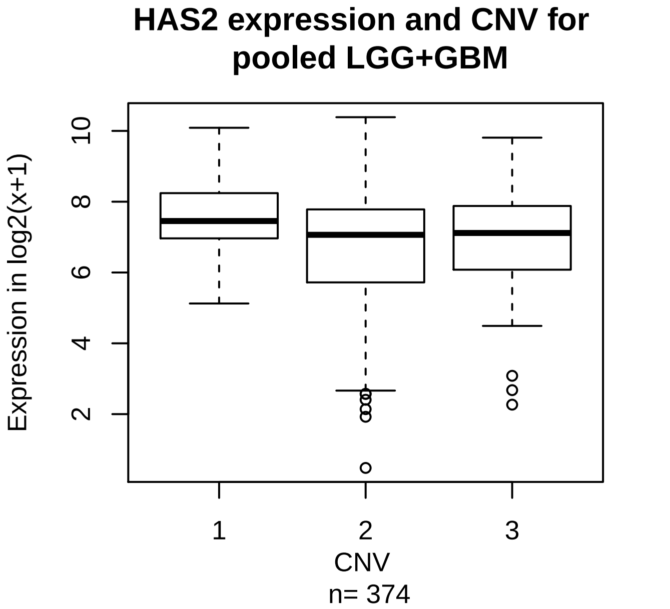
HAS2 expression and CNV for
pooled LGG+GBM
Expression in log2(x+1)
2
4
6
8
10
1
2
3
CNV
n= 374

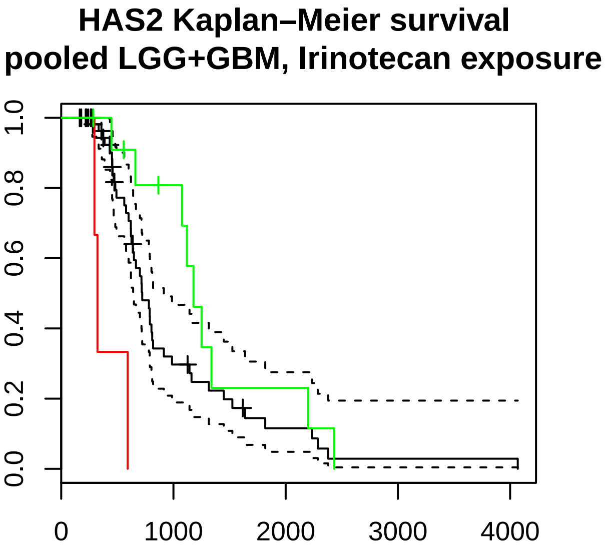
HAS2 Kaplan–Meier survival
pooled LGG+GBM, Irinotecan exposure
0.0
0.2
0.4
0.6
0.8
1.0
0
1000
2000
3000
4000

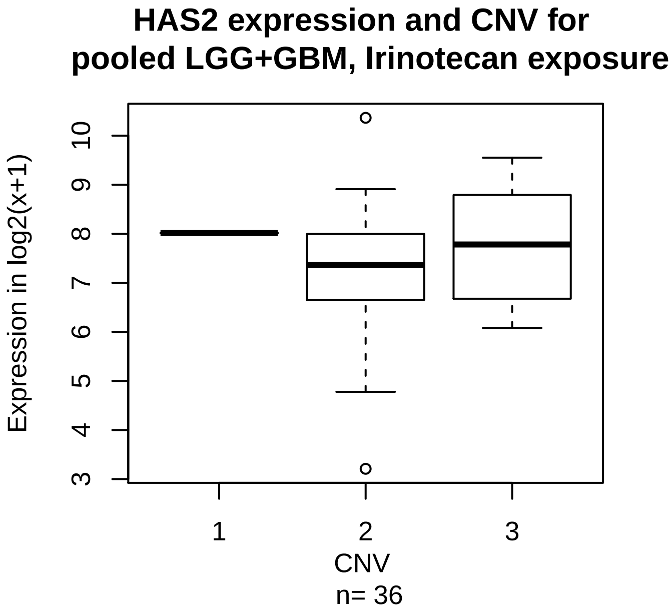
HAS2 expression and CNV for
pooled LGG+GBM, Irinotecan exposure
Expression in log2(x+1)
3
4
5
6
7
8
9
10
1
2
3
CNV
n= 36

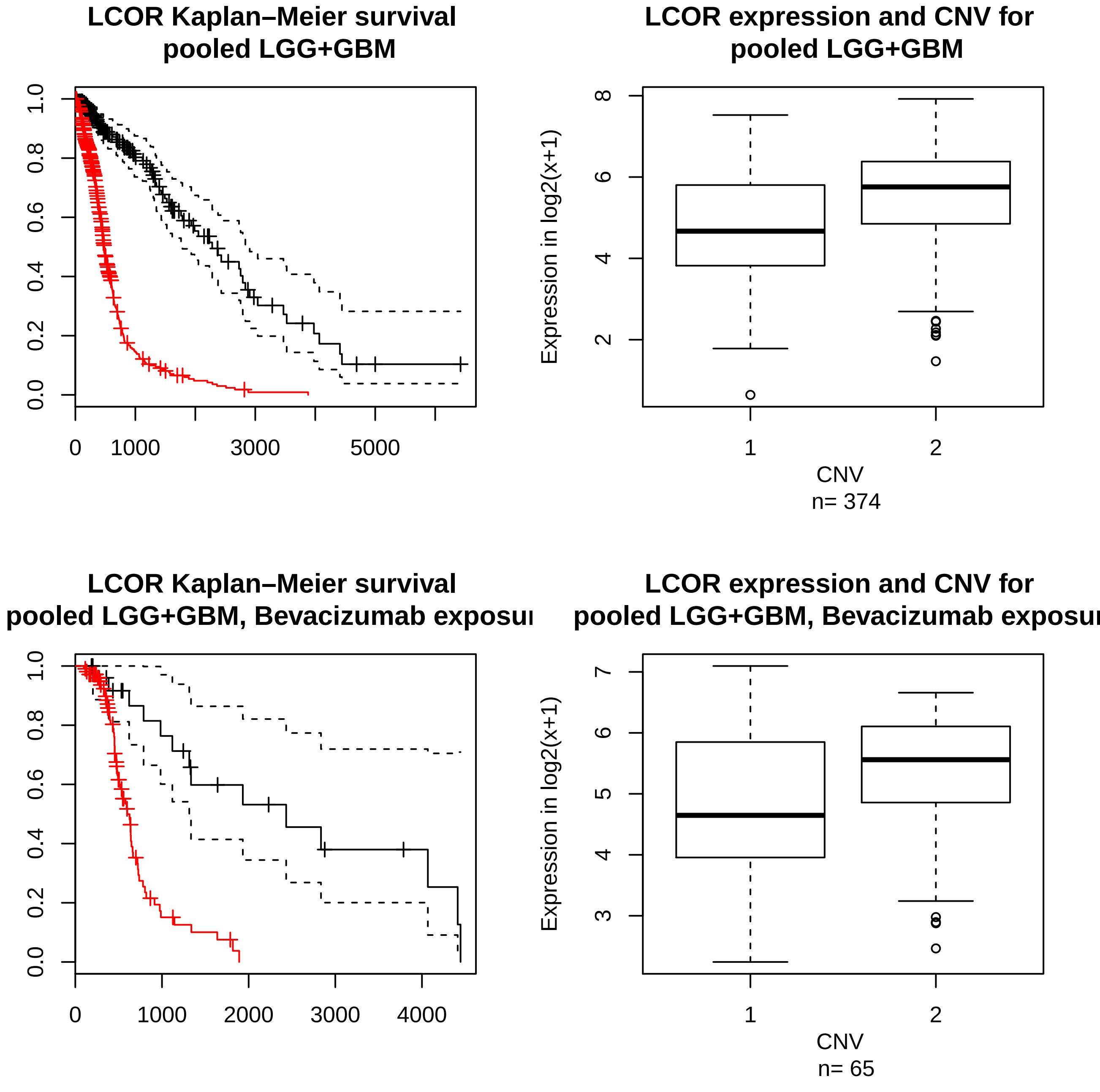

LCOR Kaplan–Meier survival
pooled LGG+GBM
0.0
0.2
0.4
0.6
0.8
1.0
0
1000
3000
5000
LCOR expression and CNV for
pooled LGG+GBM
Expression in log2(x+1)
2
4
6
8
1
2
CNV
n= 374
LCOR Kaplan–Meier survival
pooled LGG+GBM, Bevacizumab exposu
0.0
0.2
0.4
0.6
0.8
1.0
0
1000
2000
3000
4000
LCOR expression and CNV for
pooled LGG+GBM, Bevacizumab exposu
Expression in log2(x+1)
3
4
5
6
7
1
2
CNV
n= 65

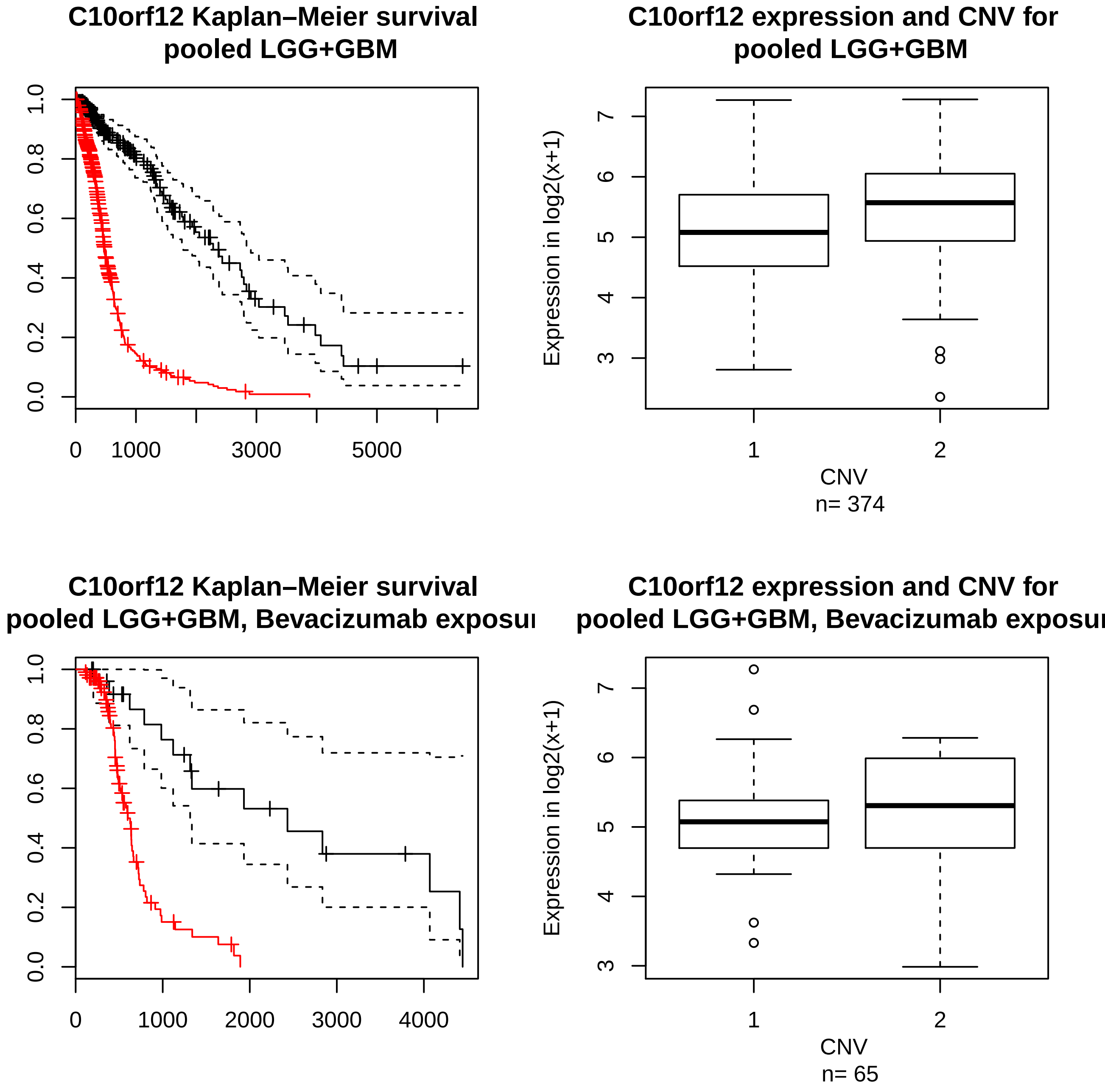
C10orf12 Kaplan–Meier survival
pooled LGG+GBM
1.0
0.8
0.6
0.4
0.2
0.0
0
1000
3000
5000
C10orf12 expression and CNV for
pooled LGG+GBM
Expression in log2(x+1)
7
6
5
4
3
1
2
CNV
n= 374
C10orf12 Kaplan–Meier survival
pooled LGG+GBM, Bevacizumab exposu
1.0
0.8
0.6
0.4
0.2
0.0
0
1000
2000
3000
4000
C10orf12 expression and CNV for
pooled LGG+GBM, Bevacizumab exposu
Expression in log2(x+1)
7
6
5
4
3
1
2
CNV
n= 65

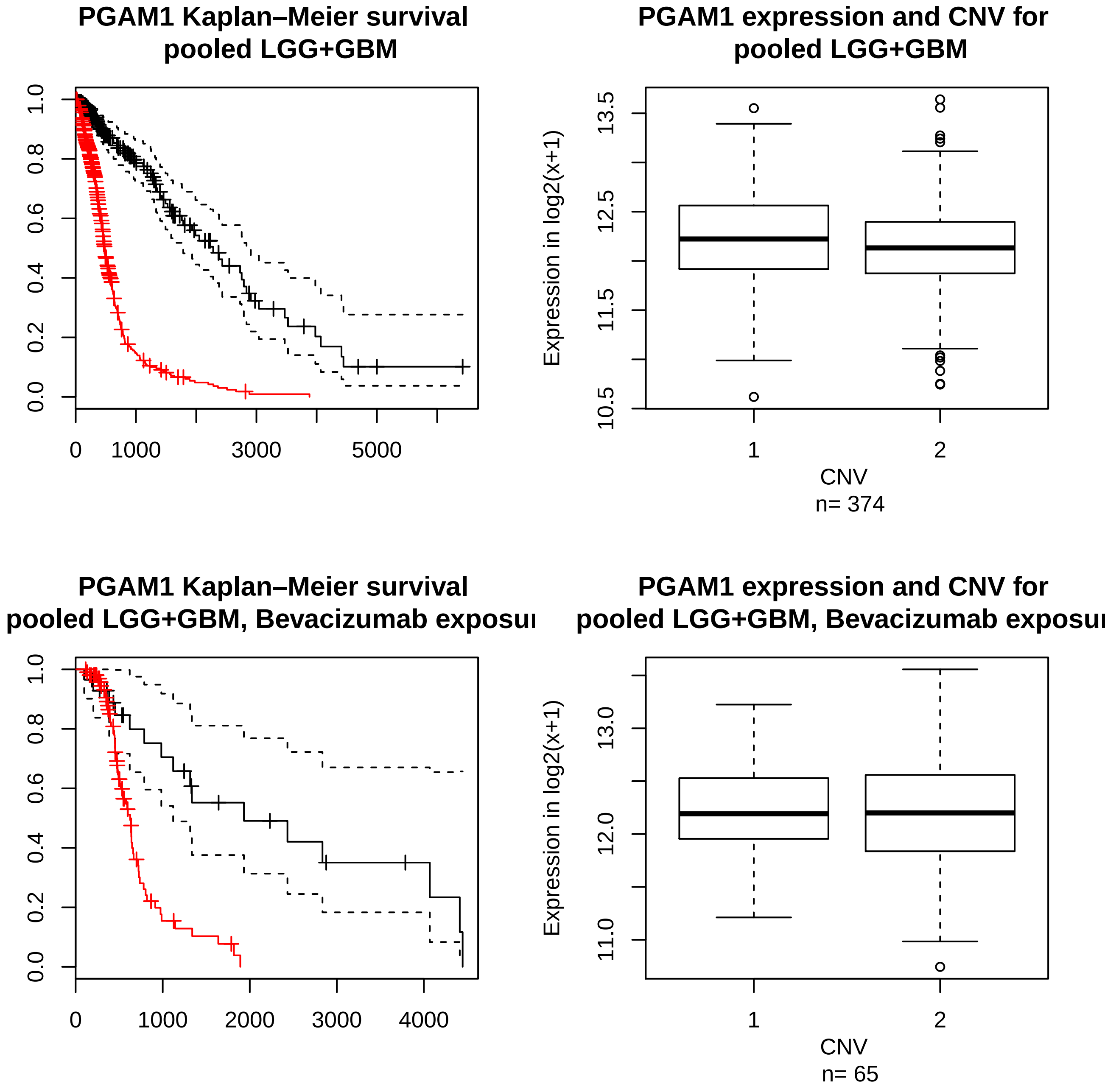
PGAM1 Kaplan–Meier survival
pooled LGG+GBM
1.0
0.8
0.6
0.4
0.2
0.0
0
1000
3000
5000
PGAM1 expression and CNV for
pooled LGG+GBM
Expression in log2(x+1)
13.5
12.5
11.5
10.5
1
2
CNV
n= 374
PGAM1 Kaplan–Meier survival
pooled LGG+GBM, Bevacizumab exposu
1.0
0.8
0.6
0.4
0.2
0.0
0
1000
2000
3000
4000
PGAM1 expression and CNV for
pooled LGG+GBM, Bevacizumab exposu
Expression in log2(x+1)
13.0
12.0
11.0
1
2
CNV
n= 65

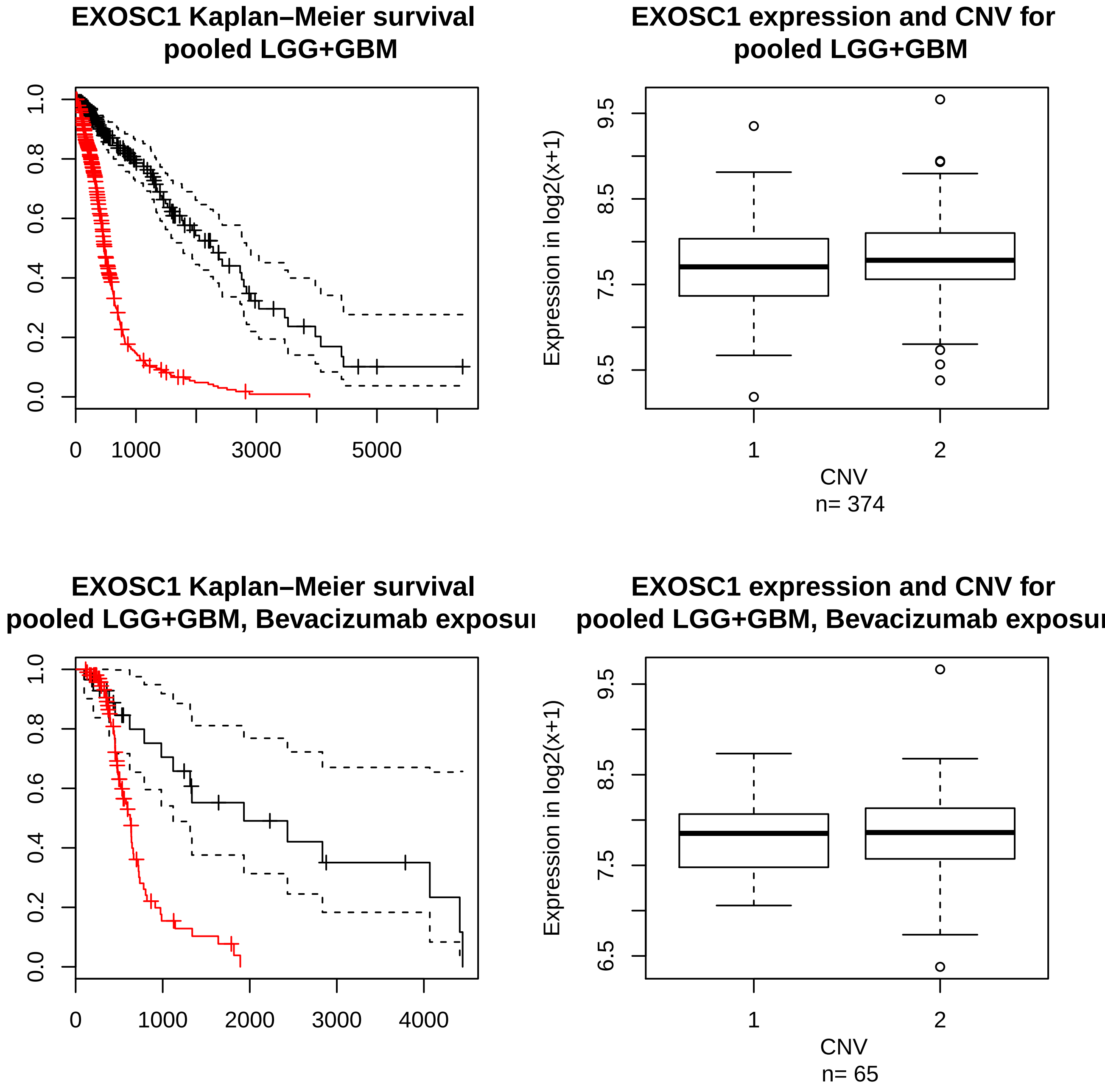
EXOSC1 Kaplan–Meier survival
pooled LGG+GBM
0.0 0.2 0.4 0.6 0.8 1.0
0 1000 3000 5000
EXOSC1 expression and CNV for
pooled LGG+GBM
Expression in log2(x+1)
6.5 7.5 8.5 9.5
1 2
CNV
n= 374
EXOSC1 Kaplan–Meier survival
pooled LGG+GBM, Bevacizumab exposu
0.0 0.2 0.4 0.6 0.8 1.0
0 1000 2000 3000 4000
EXOSC1 expression and CNV for
pooled LGG+GBM, Bevacizumab exposu
Expression in log2(x+1)
6.5 7.5 8.5 9.5
1 2
CNV
n= 65

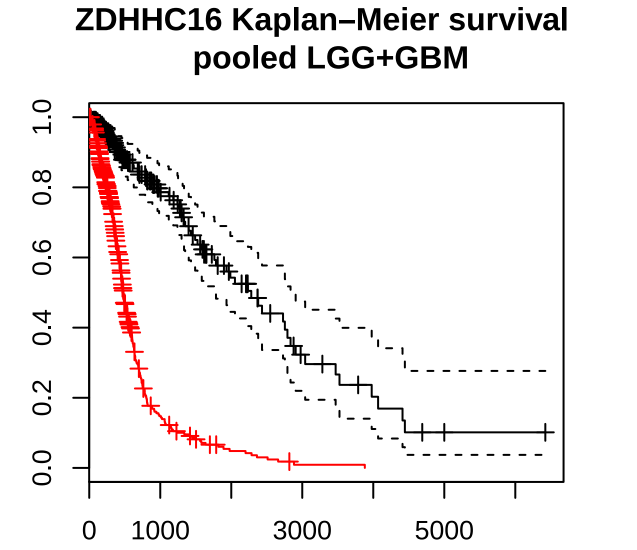
ZDHHC16 Kaplan–Meier survival
pooled LGG+GBM
0.0
0.2
0.4
0.6
0.8
1.0
0
1000
3000
5000

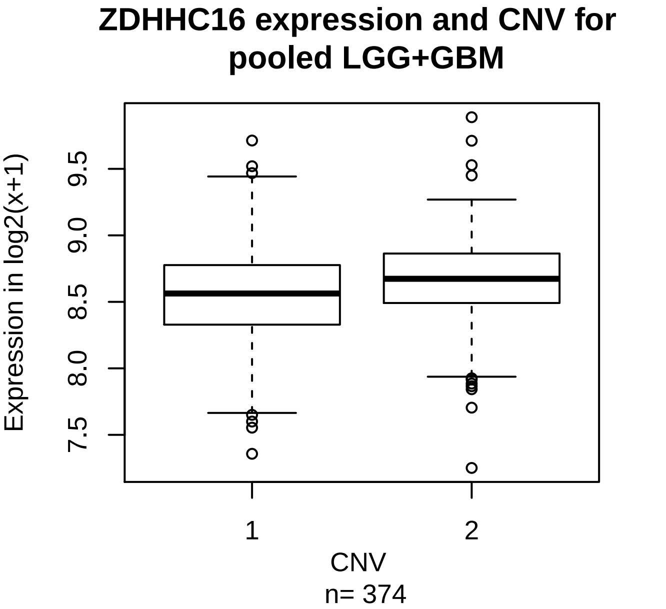
ZDHHC16 expression and CNV for
pooled LGG+GBM
Expression in log2(x+1)
7.5
8.0
8.5
9.0
9.5
1
2
CNV
n= 374

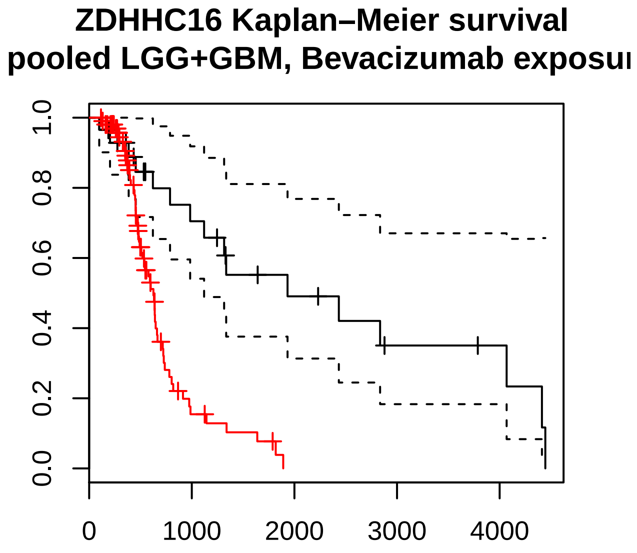
ZDHHC16 Kaplan–Meier survival
pooled LGG+GBM, Bevacizumab exposui
0.0
0.2
0.4
0.6
0.8
1.0
0
1000
2000
3000
4000

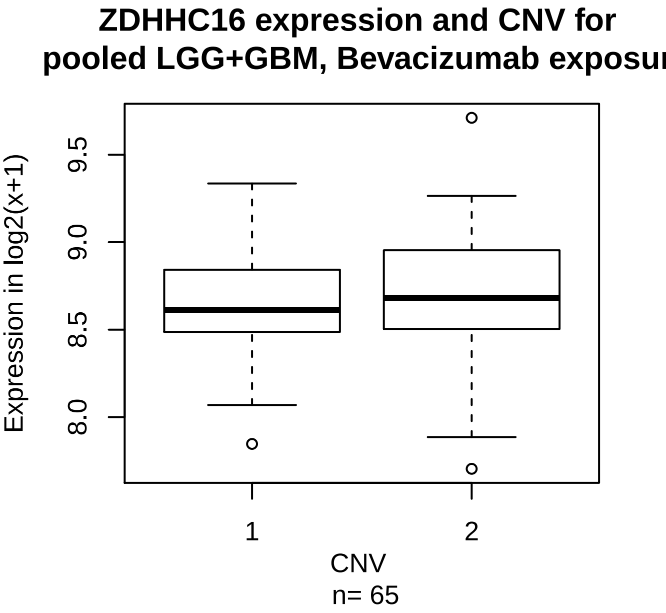
ZDHHC16 expression and CNV for
pooled LGG+GBM, Bevacizumab exposur
Expression in log2(x+1)
8.0
8.5
9.0
9.5
1
2
CNV
n= 65

MMS19 Kaplan–Meier survival
pooled LGG+GBM

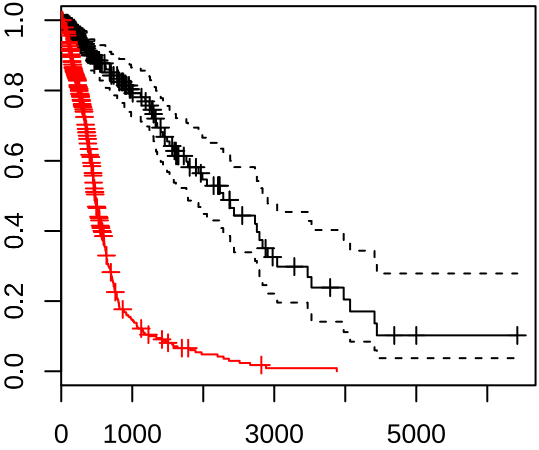

MMS19 expression and CNV for
pooled LGG+GBM

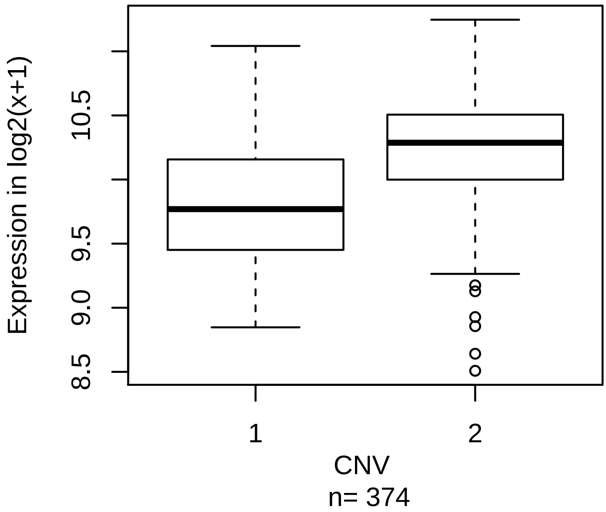

MMS19 Kaplan–Meier survival
pooled LGG+GBM, Bevacizumab exposui

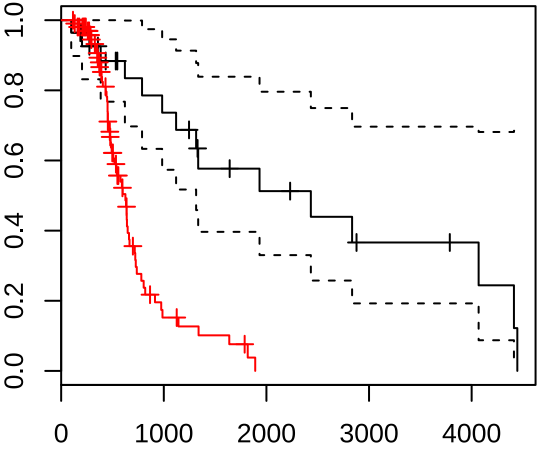

MMS19 expression and CNV for
pooled LGG+GBM, Bevacizumab exposur

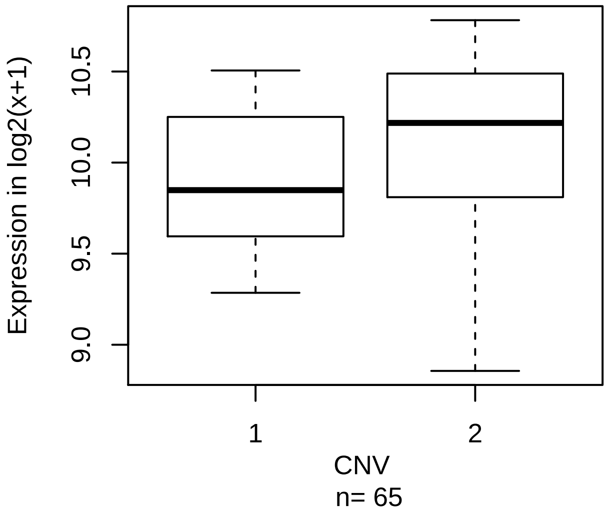

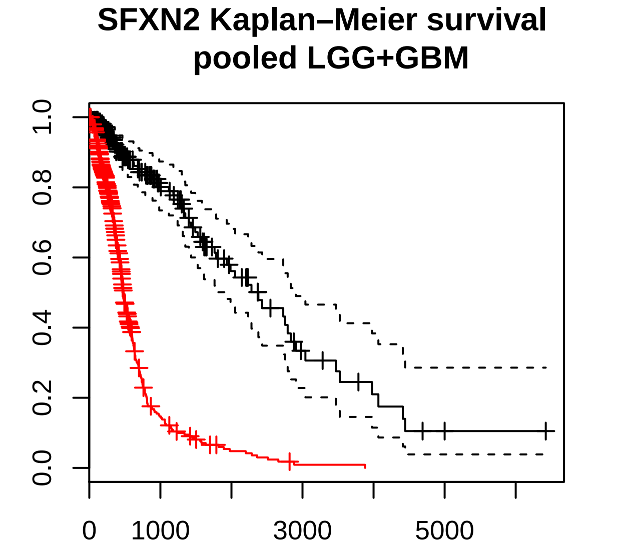
SFXN2 Kaplan–Meier survival
pooled LGG+GBM
0.0
0.2
0.4
0.6
0.8
1.0
0
1000
3000
5000

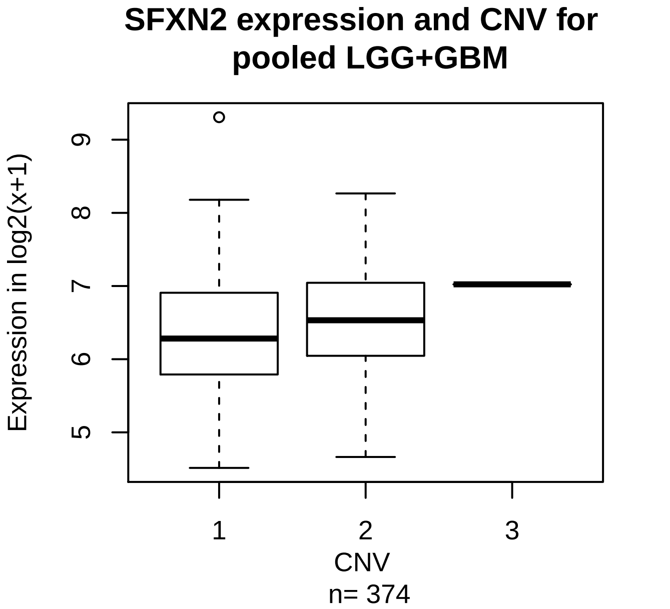
SFXN2 expression and CNV for
pooled LGG+GBM
Expression in log2(x+1)
5
6
7
8
9
1
2
3
CNV
n= 374

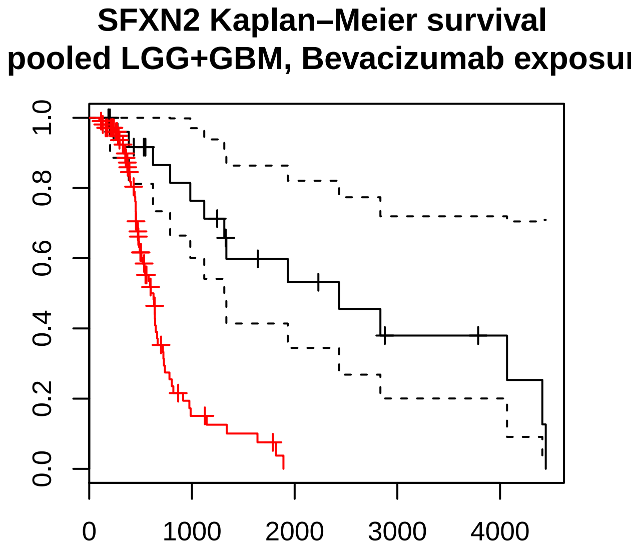
SFXN2 Kaplan–Meier survival
pooled LGG+GBM, Bevacizumab exposu
0.0
0.2
0.4
0.6
0.8
1.0
0
1000
2000
3000
4000

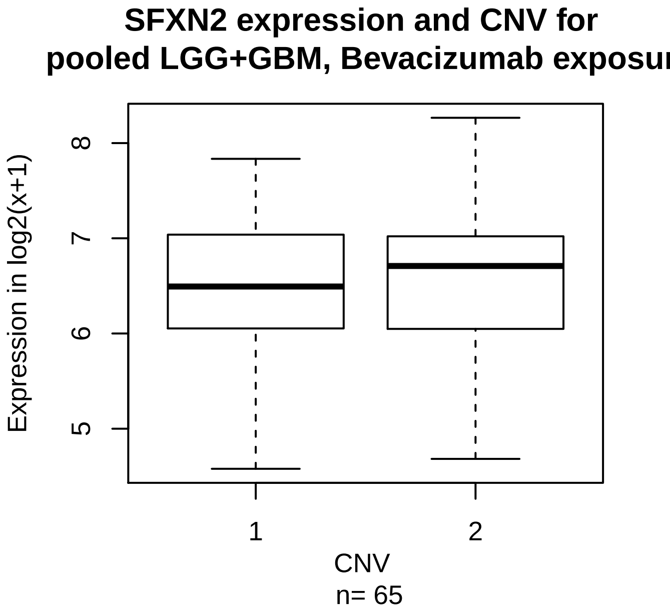
SFXN2 expression and CNV for
pooled LGG+GBM, Bevacizumab exposu
Expression in log2(x+1)
5
6
7
8
1
2
CNV
n= 65

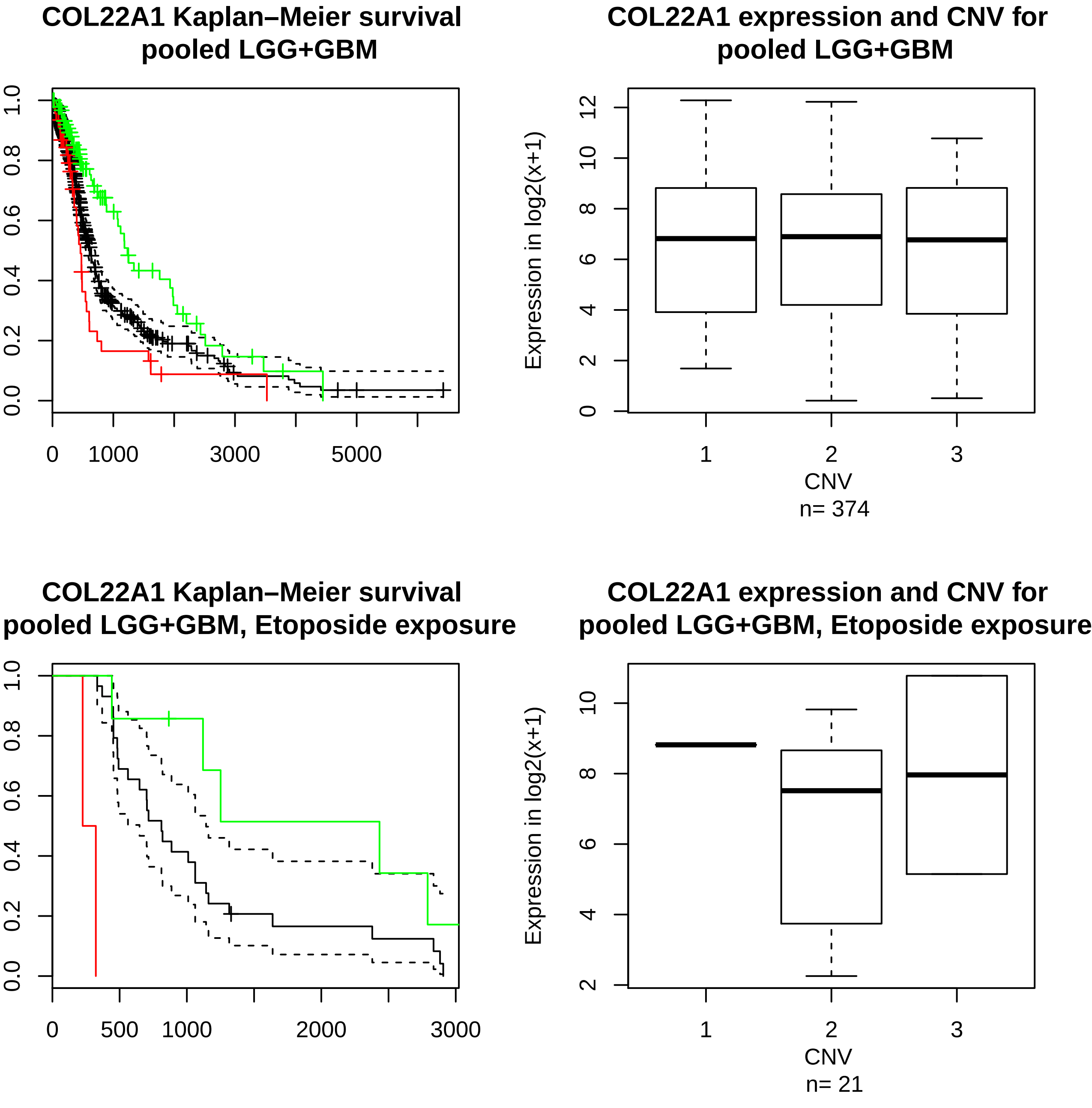
COL22A1 Kaplan–Meier survival
pooled LGG+GBM
0.0 0.2 0.4 0.6 0.8 1.0
0 1000 3000 5000
COL22A1 expression and CNV for
pooled LGG+GBM
Expression in log2(x+1)
0 2 4 6 8 10 12
1 2 3
CNV
n= 374
COL22A1 Kaplan–Meier survival
pooled LGG+GBM, Etoposide exposure
0.0 0.2 0.4 0.6 0.8 1.0
0 500 1000 2000 3000
COL22A1 expression and CNV for
pooled LGG+GBM, Etoposide exposure
Expression in log2(x+1)
2 4 6 8 10
1 2 3
CNV
n= 21

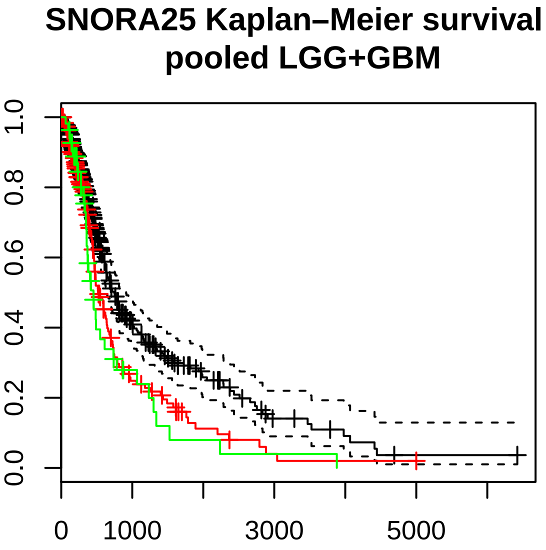
SNORA25 Kaplan–Meier survival
pooled LGG+GBM
0.0
0.2
0.4
0.6
0.8
1.0
0
1000
3000
5000

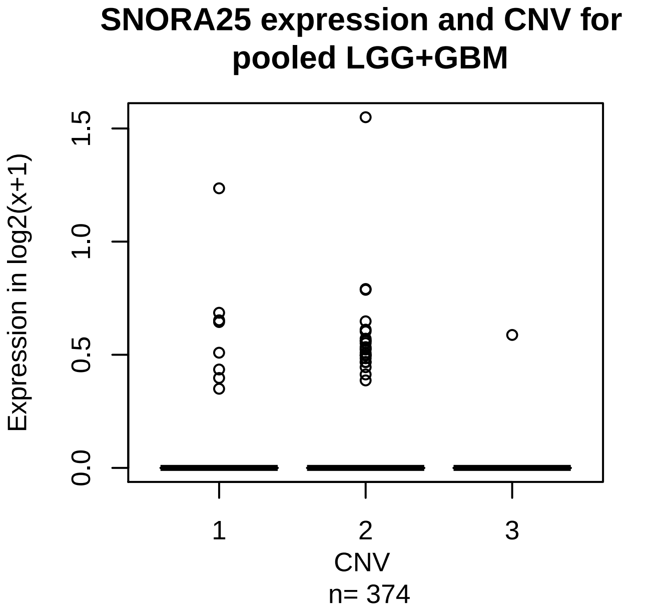
SNORA25 expression and CNV for
pooled LGG+GBM
Expression in log2(x+1)
0.0
0.5
1.0
1.5
1
2
3
CNV
n= 374

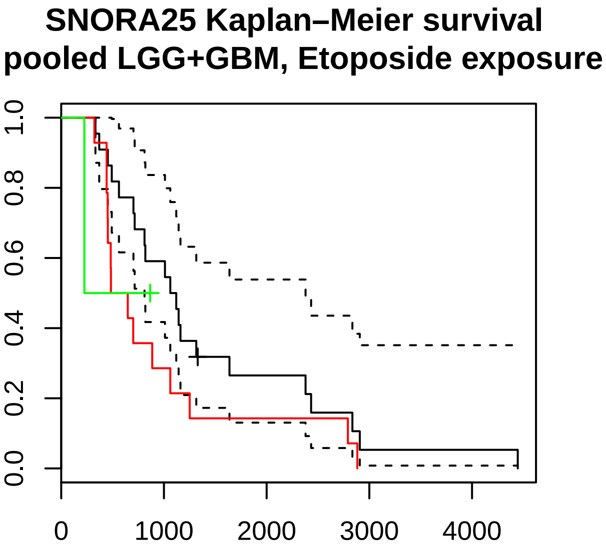
SNORA25 Kaplan–Meier survival
pooled LGG+GBM, Etoposide exposure
0.0
0.2
0.4
0.6
0.8
1.0
0
1000
2000
3000
4000

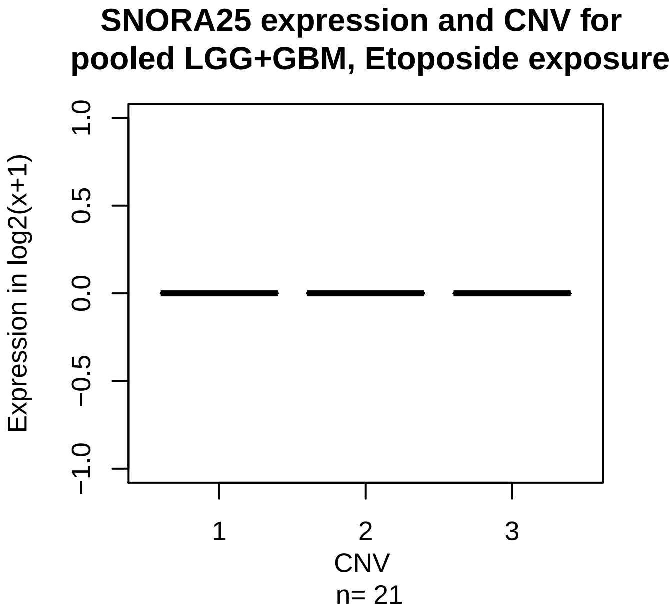
SNORA25 expression and CNV for
pooled LGG+GBM, Etoposide exposure
Expression in log2(x+1)
−1.0
−0.5
0.0
0.5
1.0
1
2
3
CNV
n= 21

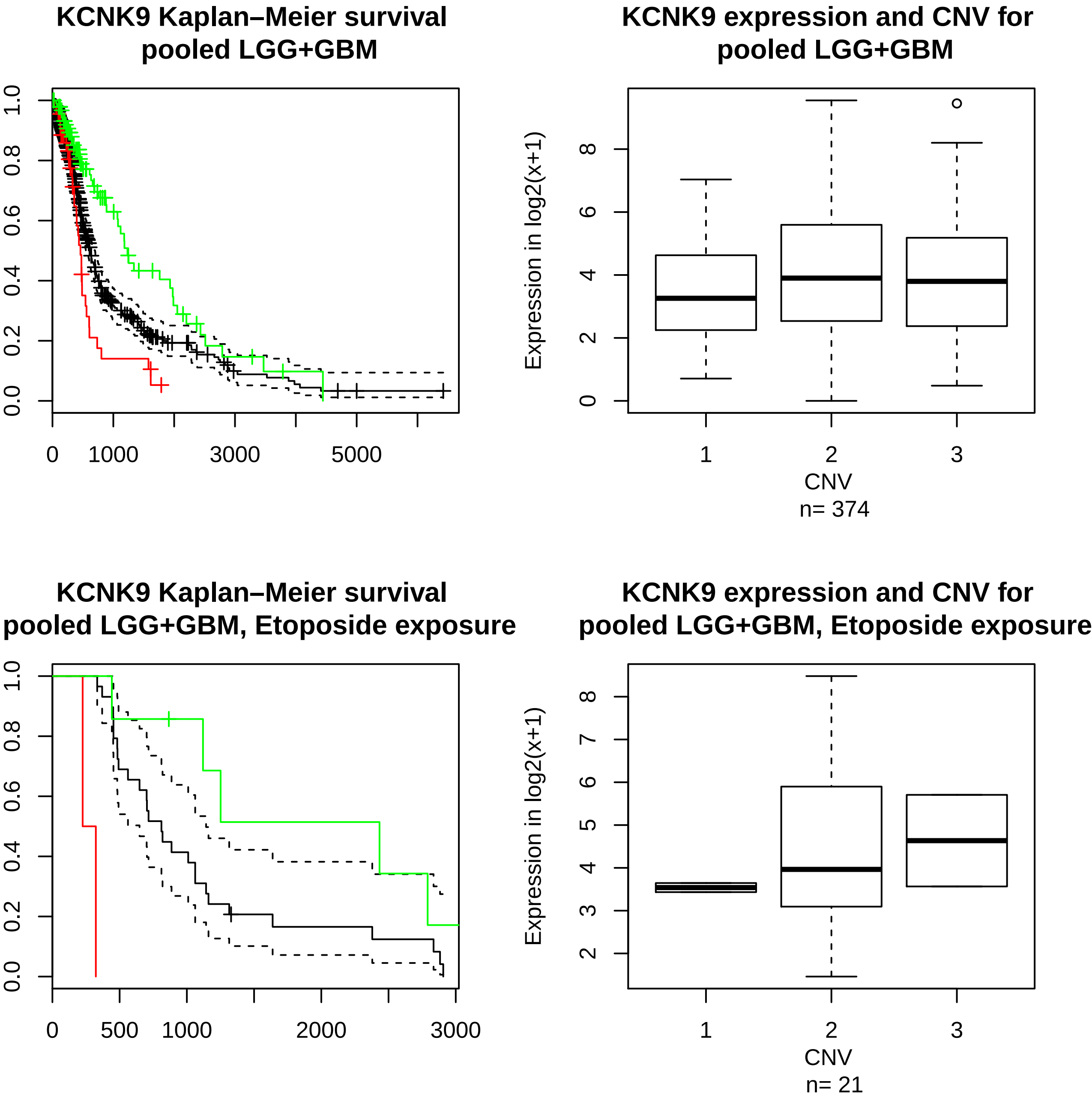
KCNK9 Kaplan–Meier survival
pooled LGG+GBM
0.0
0.2
0.4
0.6
0.8
1.0
0
1000
3000
5000
KCNK9 expression and CNV for
pooled LGG+GBM
Expression in log2(x+1)
0
2
4
6
8
1
2
3
CNV
n= 374
KCNK9 Kaplan–Meier survival
pooled LGG+GBM, Etoposide exposure
0.0
0.2
0.4
0.6
0.8
1.0
0
500
1000
2000
3000
KCNK9 expression and CNV for
pooled LGG+GBM, Etoposide exposure
Expression in log2(x+1)
2
3
4
5
6
7
8
1
2
3
CNV
n= 21

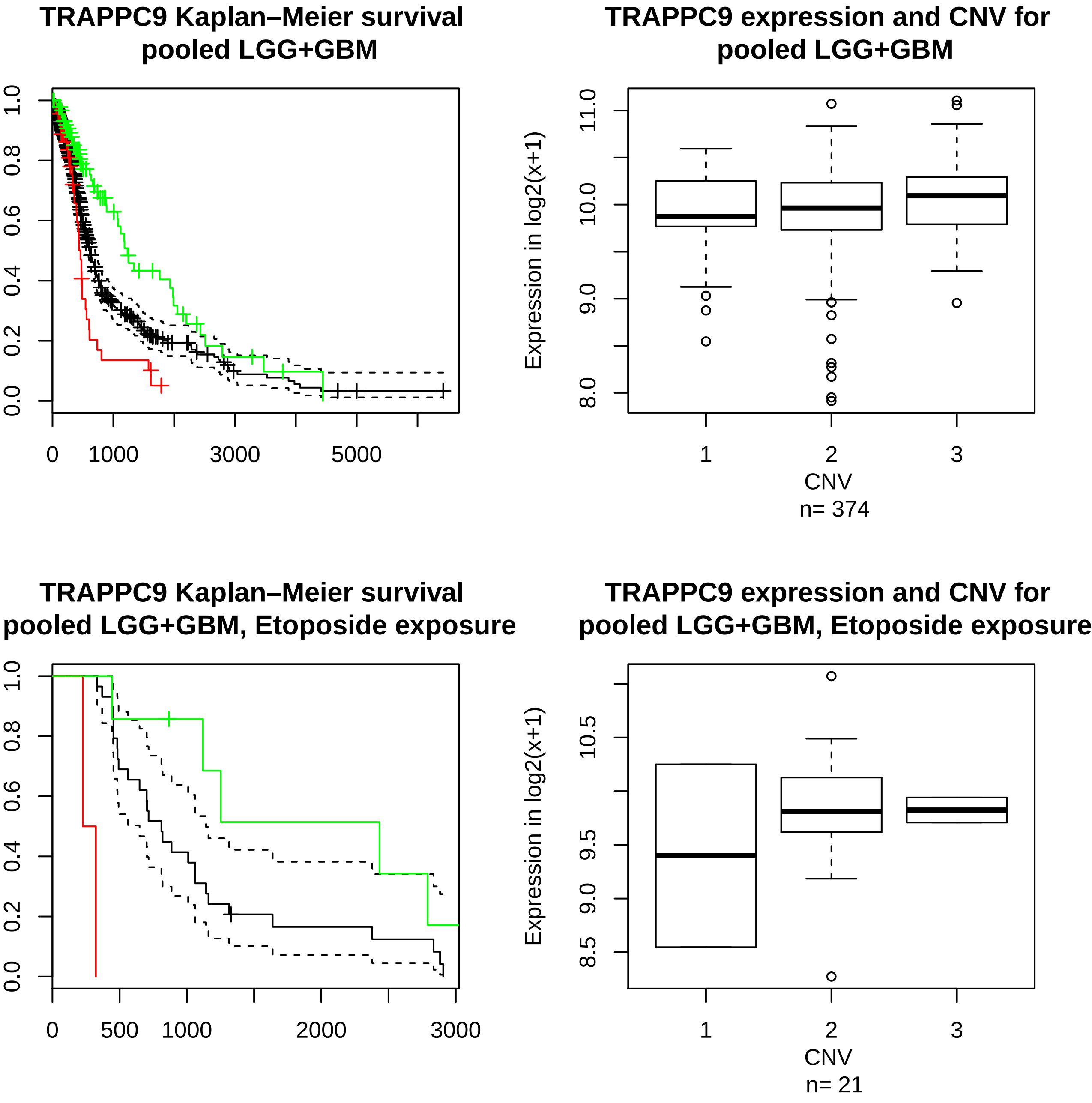
TRAPPC9 Kaplan–Meier survival pooled LGG+GBM
0.0
0.2
0.4
0.6
0.8
1.0
0
1000
3000
5000
TRAPPC9 expression and CNV for pooled LGG+GBM
Expression in log2(x+1)
8.0
9.0
10.0
11.0
1
2
3
CNV
n= 374
TRAPPC9 Kaplan–Meier survival pooled LGG+GBM, Etoposide exposure
0.0
0.2
0.4
0.6
0.8
1.0
0
500
1000
2000
3000
TRAPPC9 expression and CNV for pooled LGG+GBM, Etoposide exposure
Expression in log2(x+1)
8.5
9.0
9.5
10.5
1
2
3
CNV
n= 21

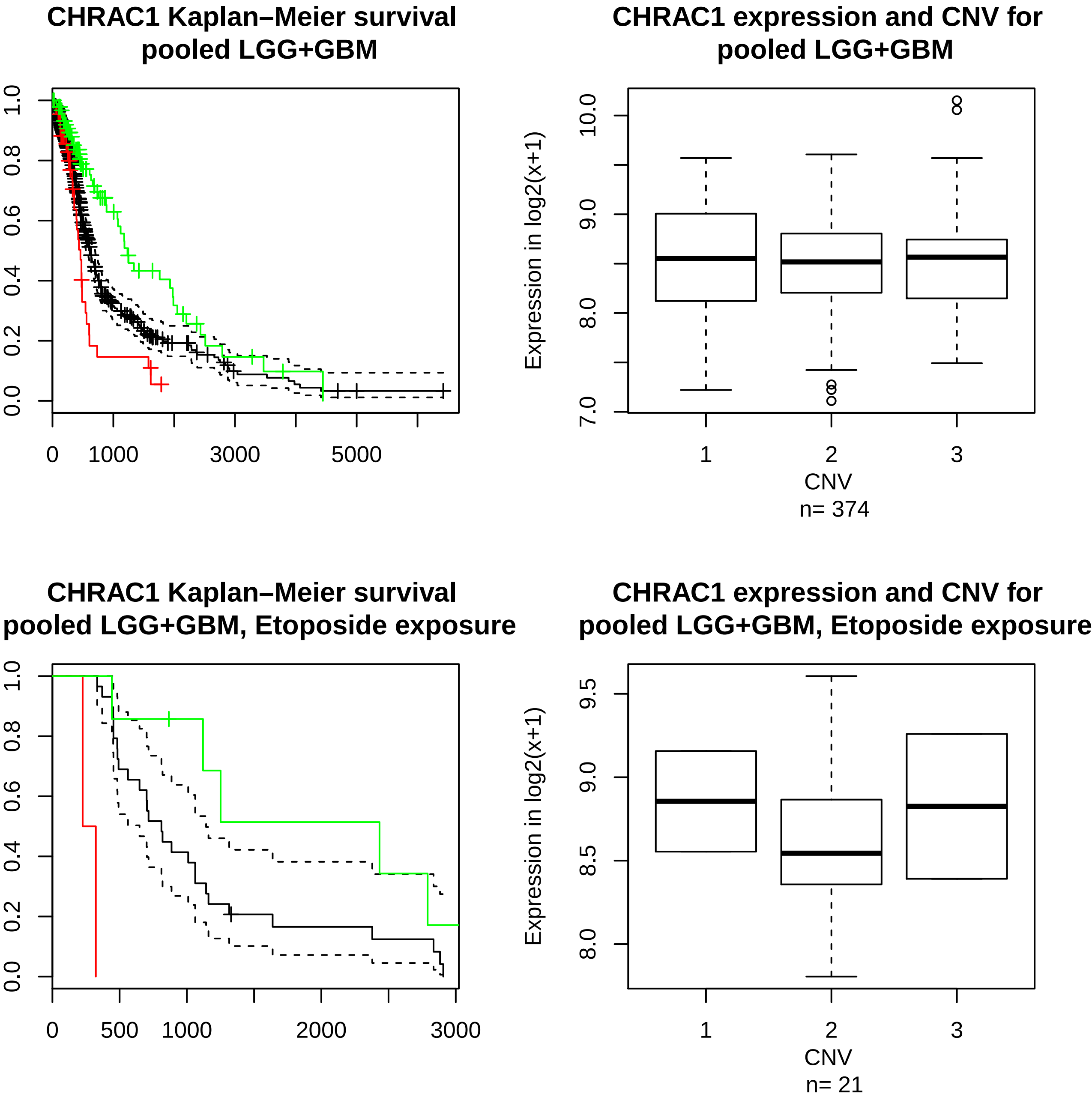
CHRAC1 Kaplan–Meier survival
pooled LGG+GBM
0.0
0.2
0.4
0.6
0.8
1.0
0
1000
3000
5000
CHRAC1 expression and CNV for
pooled LGG+GBM
Expression in log2(x+1)
7.0
8.0
9.0
10.0
1
2
3
CNV
n= 374
CHRAC1 Kaplan–Meier survival
pooled LGG+GBM, Etoposide exposure
0.0
0.2
0.4
0.6
0.8
1.0
0
500
1000
2000
3000
CHRAC1 expression and CNV for
pooled LGG+GBM, Etoposide exposure
Expression in log2(x+1)
8.0
8.5
9.0
9.5
1
2
3
CNV
n= 21

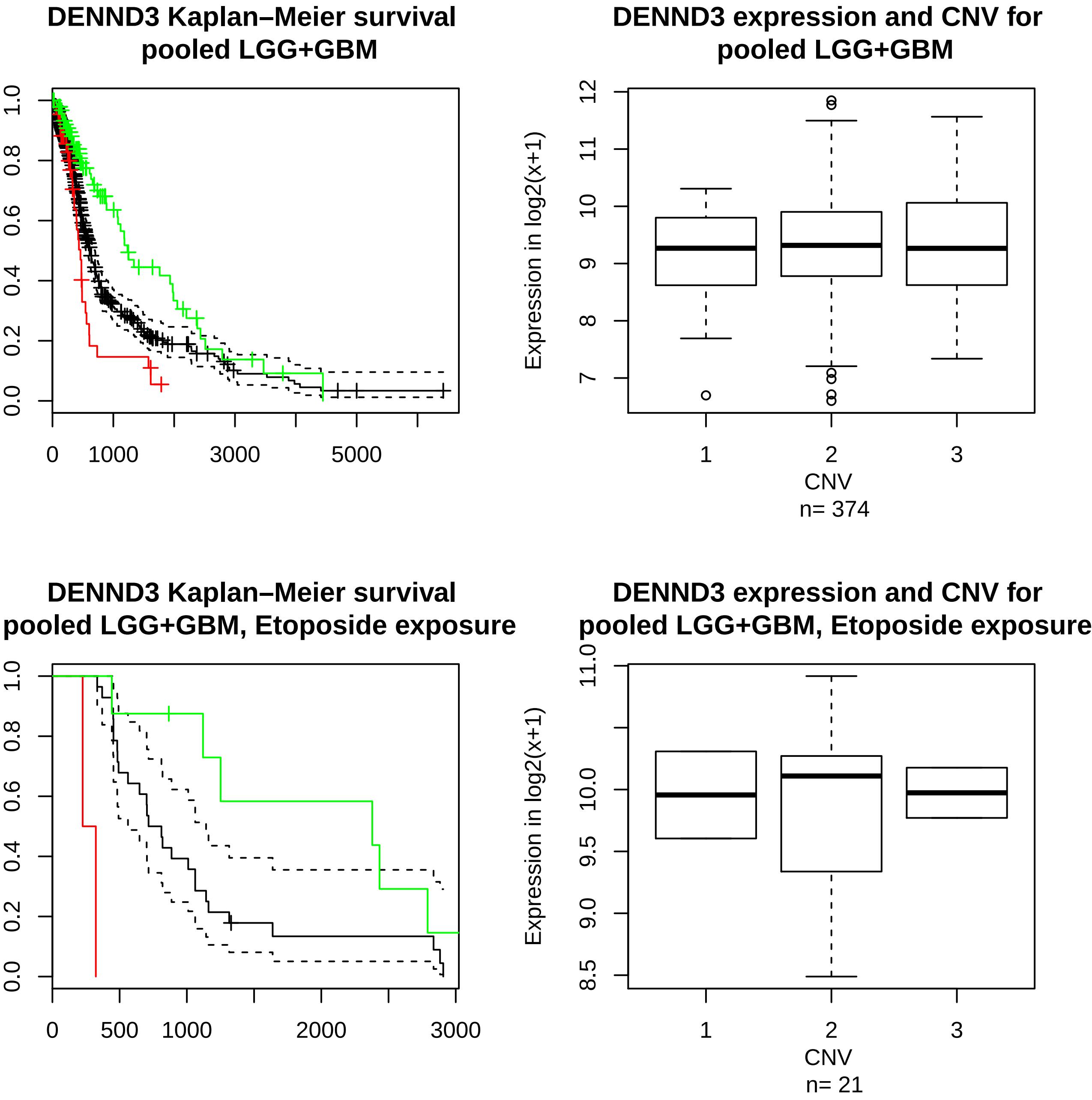

DENND3 Kaplan–Meier survival
pooled LGG+GBM
0.0
0.2
0.4
0.6
0.8
1.0
0
1000
3000
5000
DENND3 expression and CNV for
pooled LGG+GBM
Expression in log2(x+1)
7
8
9
10
11
12
1
2
3
CNV
n= 374
DENND3 Kaplan–Meier survival
pooled LGG+GBM, Etoposide exposure
0.0
0.2
0.4
0.6
0.8
1.0
0
500
1000
2000
3000
DENND3 expression and CNV for
pooled LGG+GBM, Etoposide exposure
Expression in log2(x+1)
8.5
9.0
9.5
10.0
11.0
1
2
3
CNV
n= 21

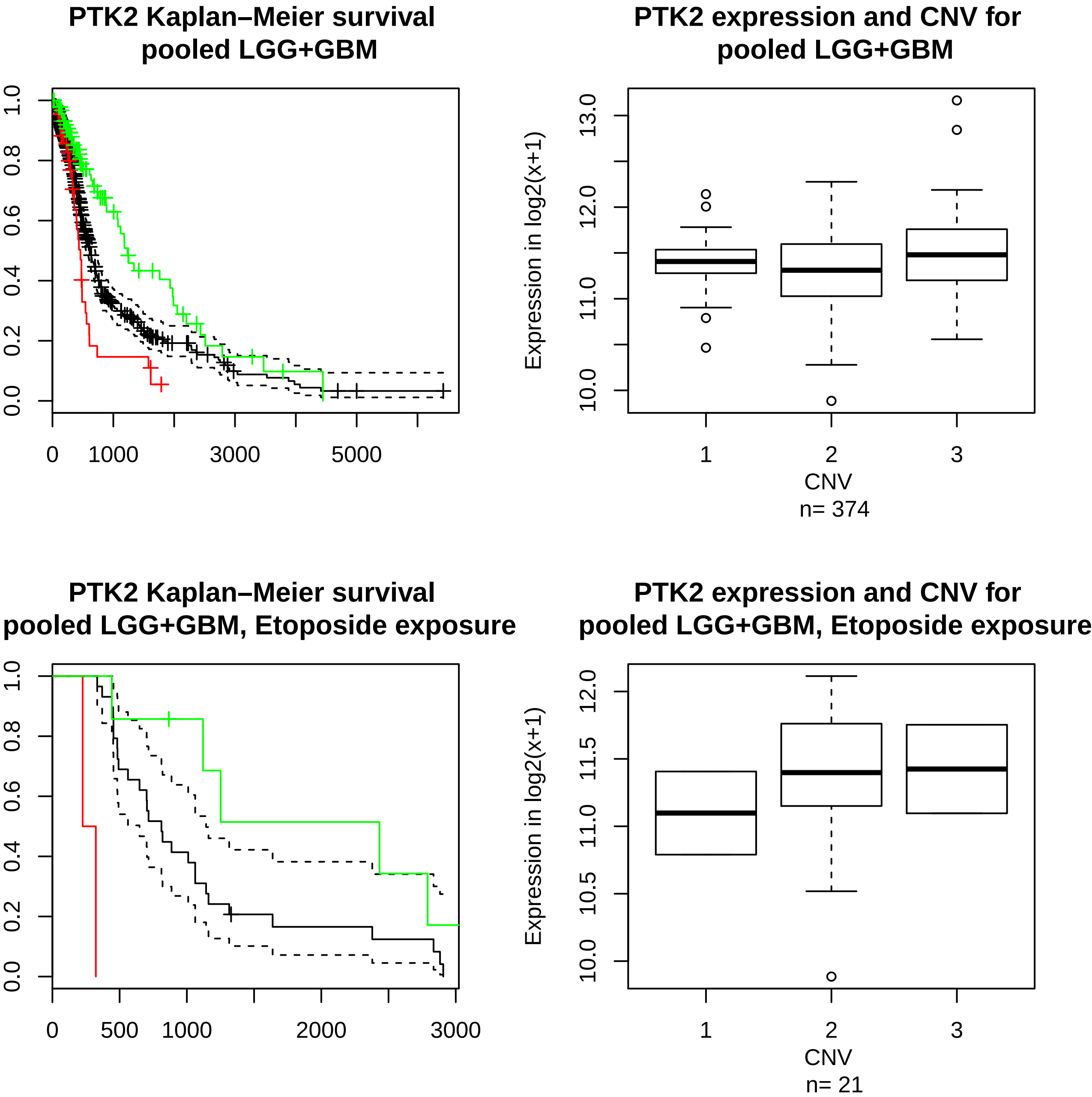
PTK2 Kaplan–Meier survival
pooled LGG+GBM
0.0
0.2
0.4
0.6
0.8
1.0
0
1000
3000
5000
PTK2 expression and CNV for
pooled LGG+GBM
Expression in log2(x+1)
10.0
11.0
12.0
13.0
1
2
3
CNV
n= 374
PTK2 Kaplan–Meier survival
pooled LGG+GBM, Etoposide exposure
0.0
0.2
0.4
0.6
0.8
1.0
0
500
1000
2000
3000
PTK2 expression and CNV for
pooled LGG+GBM, Etoposide exposure
Expression in log2(x+1)
10.0
10.5
11.0
11.5
12.0
1
2
3
CNV
n= 21

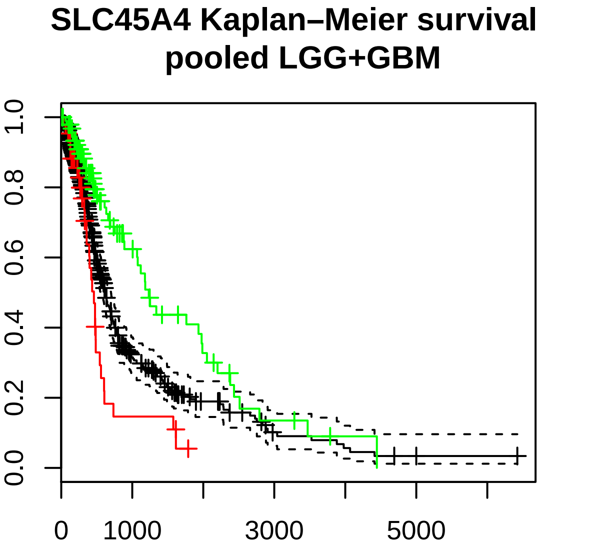
SLC45A4 Kaplan–Meier survival
pooled LGG+GBM
1.0
0.8
0.6
0.4
0.2
0.0
0
1000
3000
5000

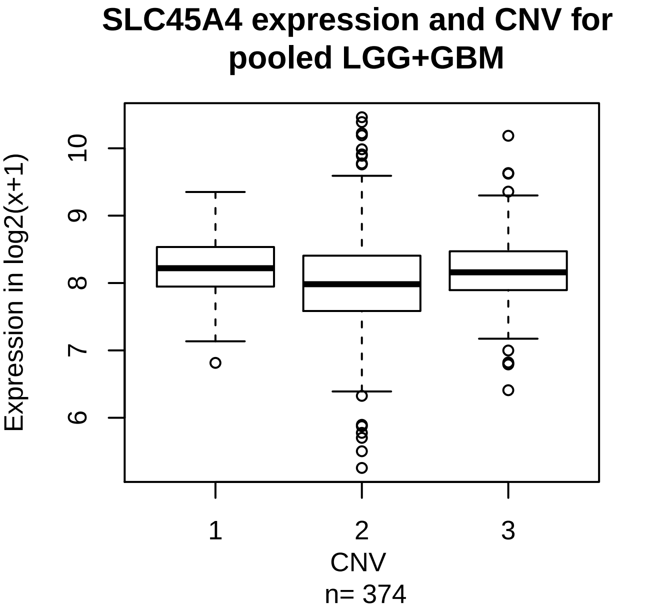
SLC45A4 expression and CNV for
pooled LGG+GBM
Expression in log2(x+1)
10
9
8
7
6
1
2
3
CNV
n= 374

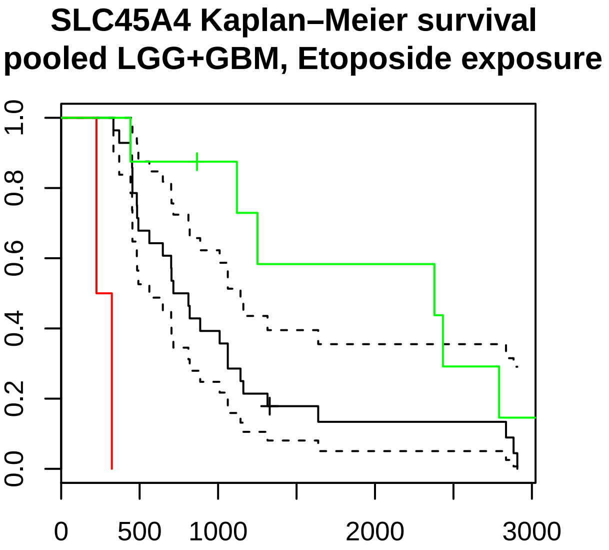
SLC45A4 Kaplan–Meier survival
pooled LGG+GBM, Etoposide exposure
1.0
0.8
0.6
0.4
0.2
0.0
0
500
1000
2000
3000

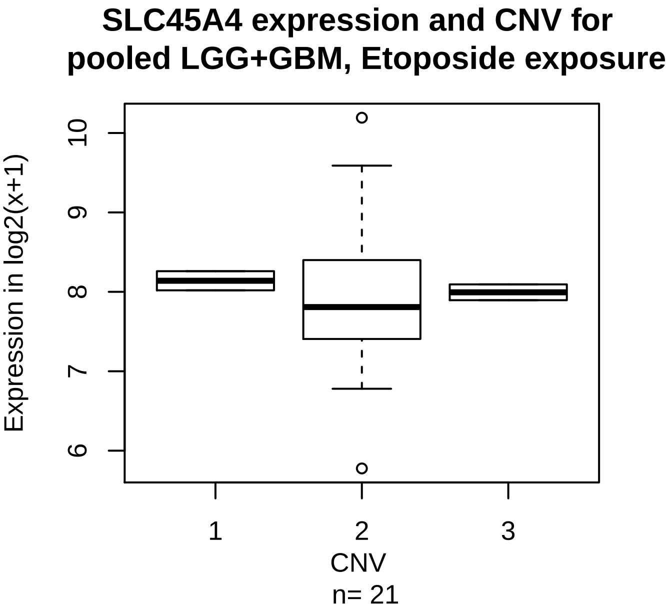
SLC45A4 expression and CNV for
pooled LGG+GBM, Etoposide exposure
Expression in log2(x+1)
10
9
8
7
6
1
2
3
CNV
n= 21

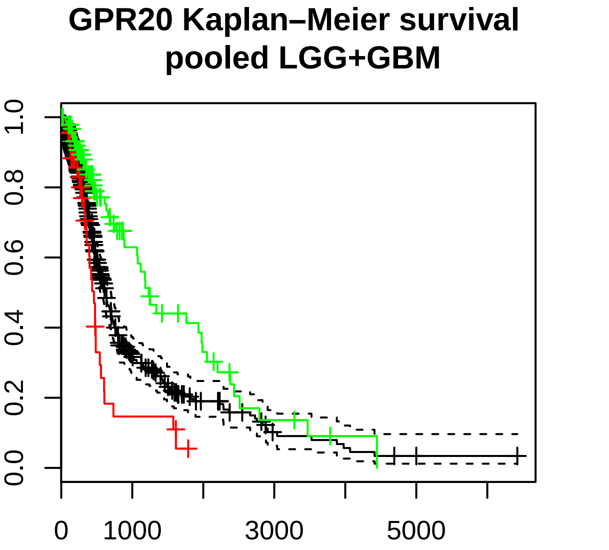
GPR20 Kaplan–Meier survival
pooled LGG+GBM
0.0
0.2
0.4
0.6
0.8
1.0
0
1000
3000
5000

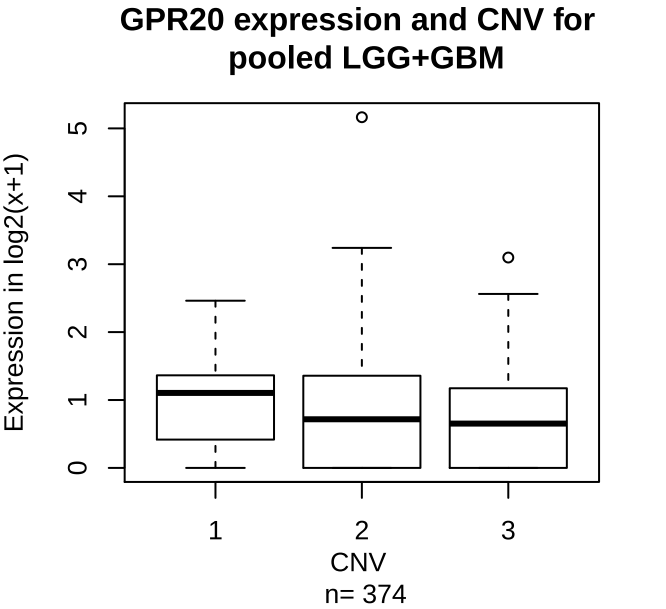
GPR20 expression and CNV for
pooled LGG+GBM
Expression in log2(x+1)
0
1
2
3
4
5
1
2
3
CNV
n= 374

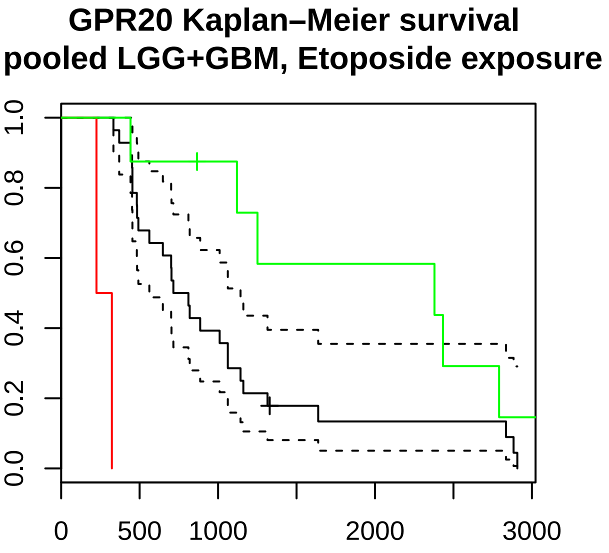
GPR20 Kaplan–Meier survival
pooled LGG+GBM, Etoposide exposure
0.0
0.2
0.4
0.6
0.8
1.0
0
500
1000
2000
3000

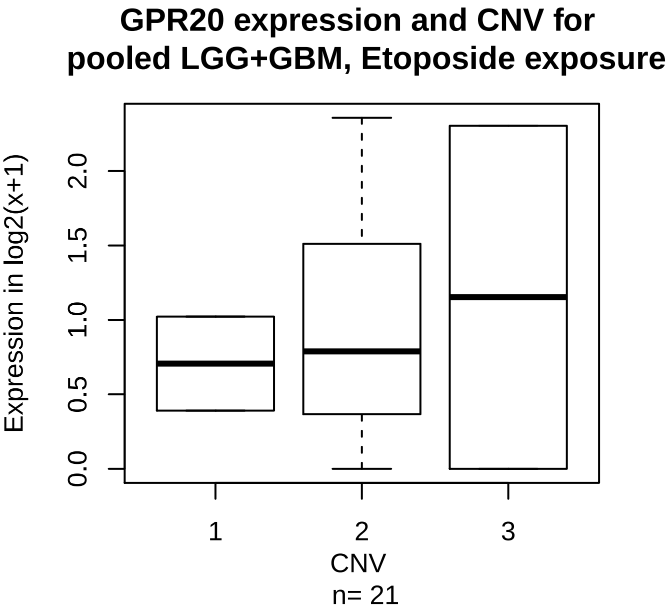
GPR20 expression and CNV for
pooled LGG+GBM, Etoposide exposure
Expression in log2(x+1)
0.0
0.5
1.0
1.5
2.0
1
2
3
CNV
n= 21

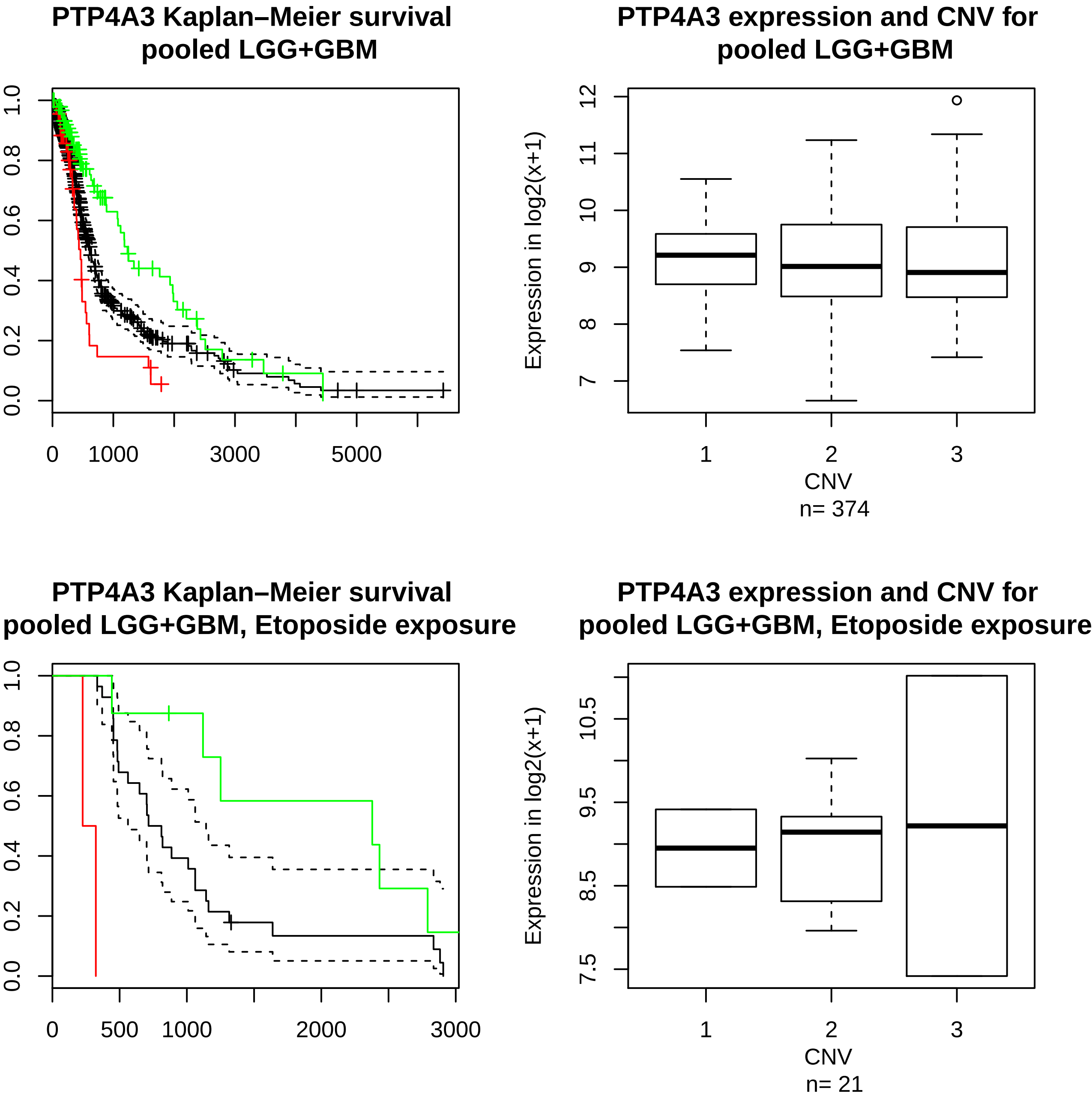
PTP4A3 Kaplan–Meier survival
pooled LGG+GBM
PTP4A3 expression and CNV for
pooled LGG+GBM
Expression in log2(x+1)
CNV
n= 374
PTP4A3 Kaplan–Meier survival
pooled LGG+GBM, Etoposide exposure
PTP4A3 expression and CNV for
pooled LGG+GBM, Etoposide exposure
Expression in log2(x+1)
CNV
n= 21

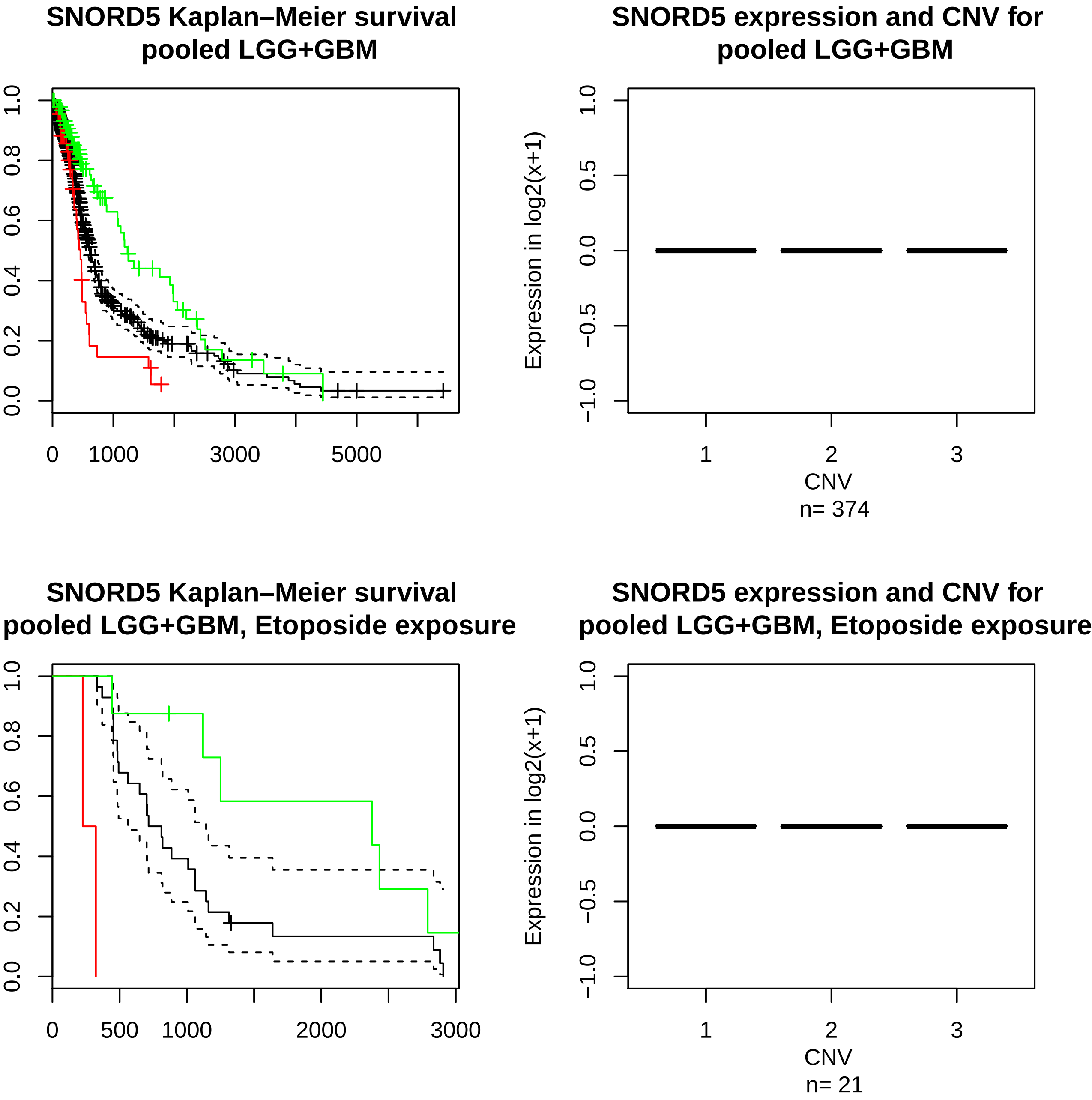
SNORD5 Kaplan–Meier survival
pooled LGG+GBM
0.0
0.2
0.4
0.6
0.8
1.0
0
1000
3000
5000
SNORD5 expression and CNV for
pooled LGG+GBM
Expression in log2(x+1)
−1.0
−0.5
0.0
0.5
1.0
1
2
3
CNV
n= 374
SNORD5 Kaplan–Meier survival
pooled LGG+GBM, Etoposide exposure
0.0
0.2
0.4
0.6
0.8
1.0
0
500
1000
2000
3000
SNORD5 expression and CNV for
pooled LGG+GBM, Etoposide exposure
Expression in log2(x+1)
−1.0
−0.5
0.0
0.5
1.0
1
2
3
CNV
n= 21

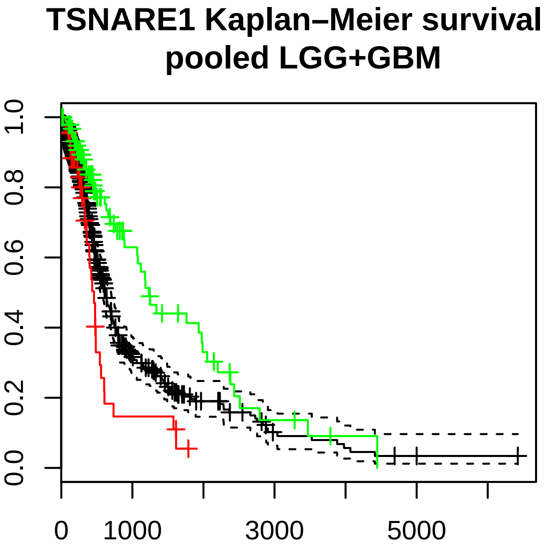
TSNARE1 Kaplan–Meier survival
pooled LGG+GBM
0.0
0.2
0.4
0.6
0.8
1.0
0
1000
3000
5000

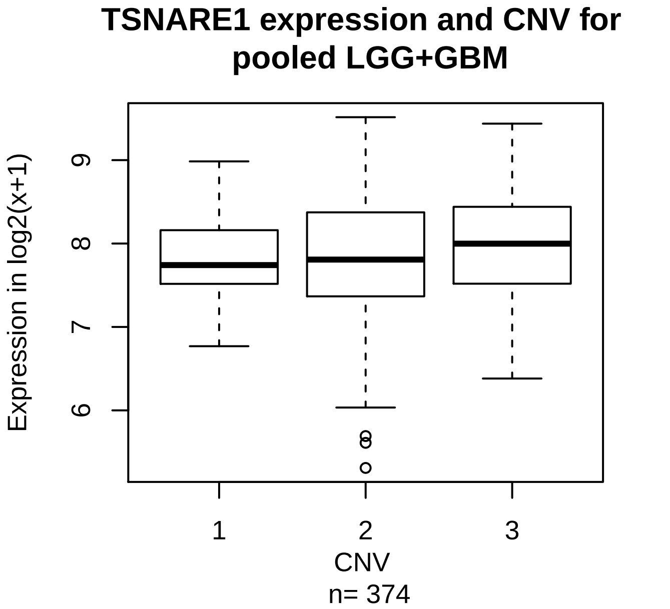
TSNARE1 expression and CNV for
pooled LGG+GBM
Expression in log2(x+1)
6
7
8
9
1
2
3
CNV
n= 374

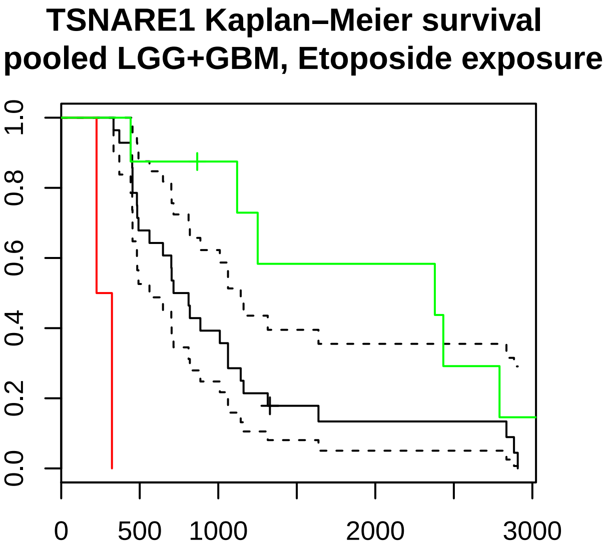
TSNARE1 Kaplan–Meier survival
pooled LGG+GBM, Etoposide exposure
0.0
0.2
0.4
0.6
0.8
1.0
0
500
1000
2000
3000

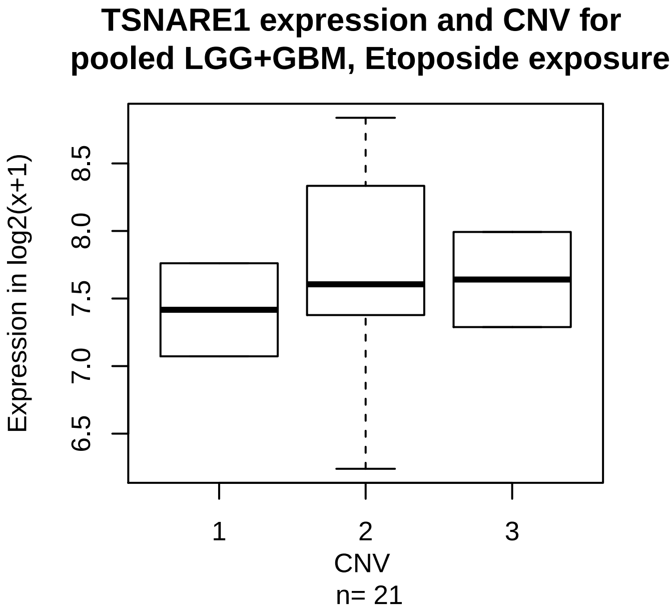
TSNARE1 expression and CNV for
pooled LGG+GBM, Etoposide exposure
Expression in log2(x+1)
6.5
7.0
7.5
8.0
8.5
1
2
3
CNV
n= 21

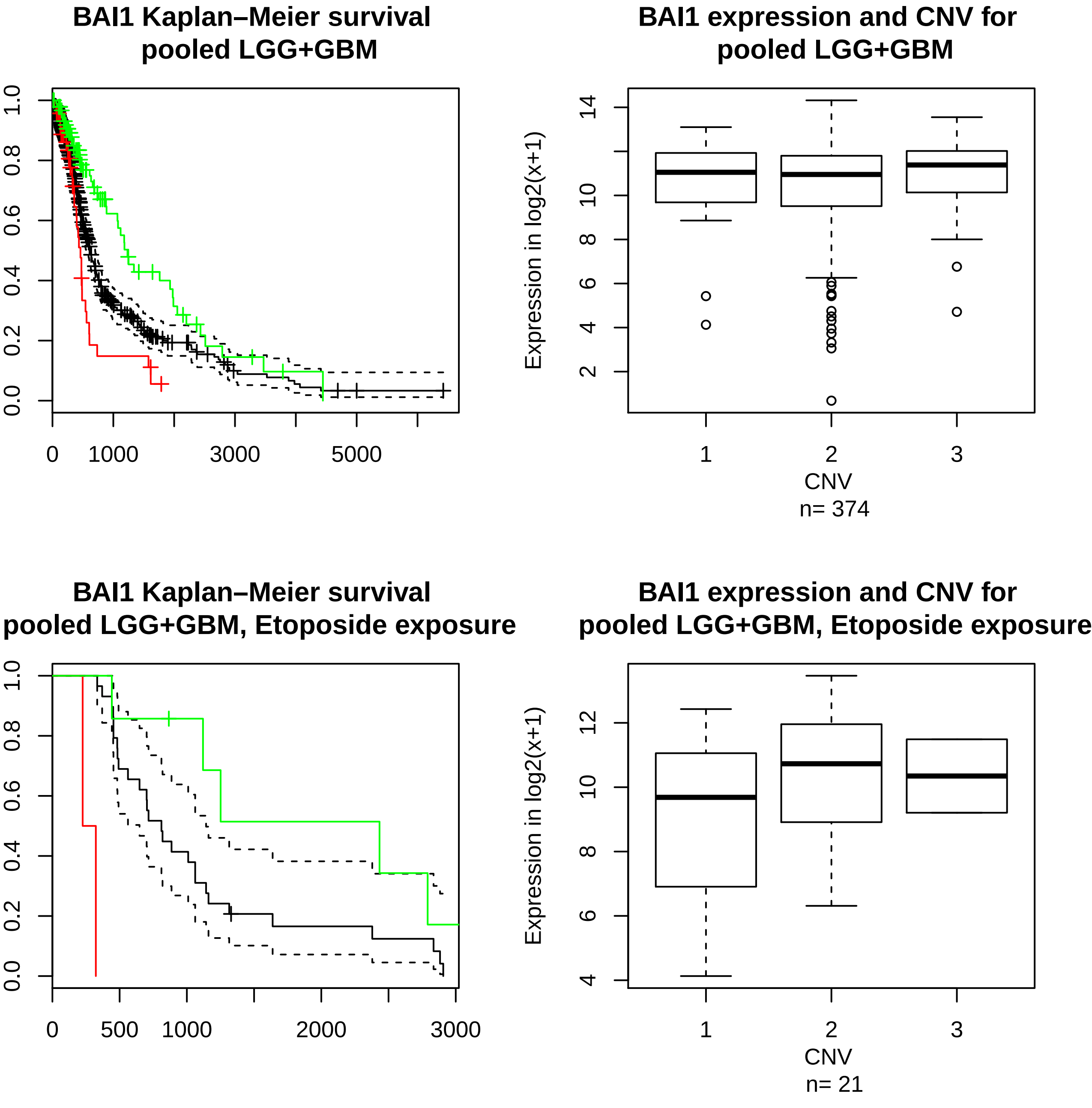
BAI1 Kaplan–Meier survival
pooled LGG+GBM
0.0
0.2
0.4
0.6
0.8
1.0
0
1000
3000
5000
BAI1 expression and CNV for
pooled LGG+GBM
Expression in log2(x+1)
2
4
6
8
10
14
1
2
3
CNV
n= 374
BAI1 Kaplan–Meier survival
pooled LGG+GBM, Etoposide exposure
0.0
0.2
0.4
0.6
0.8
1.0
0
500
1000
2000
3000
BAI1 expression and CNV for
pooled LGG+GBM, Etoposide exposure
Expression in log2(x+1)
4
6
8
10
12
1
2
3
CNV
n= 21

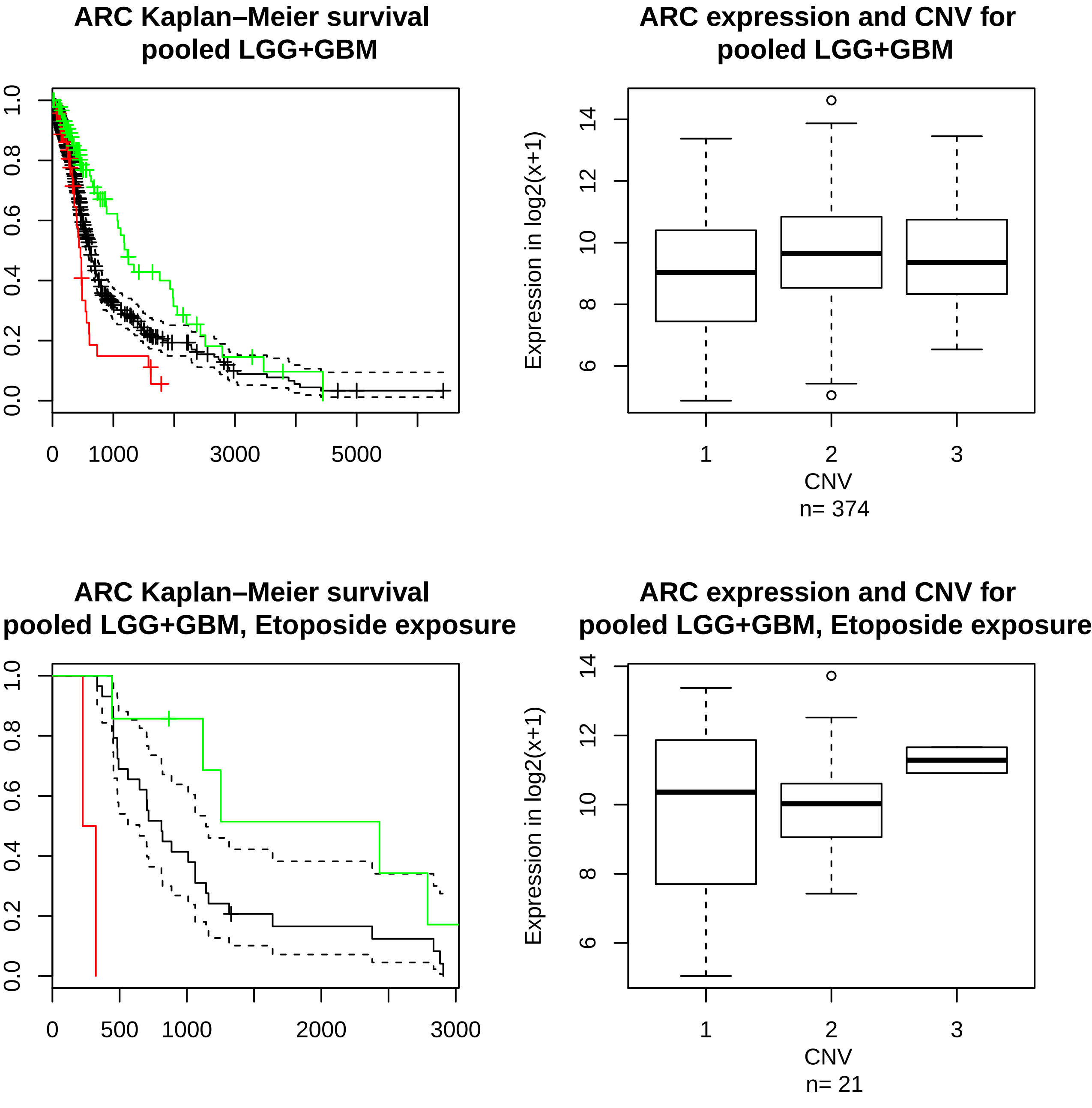
ARC Kaplan–Meier survival
pooled LGG+GBM
0.0 0.2 0.4 0.6 0.8 1.0
0 1000 3000 5000
ARC expression and CNV for
pooled LGG+GBM
Expression in log2(x+1)
6 8 10 12 14
1 2 3
CNV
n= 374
ARC Kaplan–Meier survival
pooled LGG+GBM, Etoposide exposure
0.0 0.2 0.4 0.6 0.8 1.0
0 500 1000 2000 3000
ARC expression and CNV for
pooled LGG+GBM, Etoposide exposure
Expression in log2(x+1)
6 8 10 12 14
1 2 3
CNV
n= 21

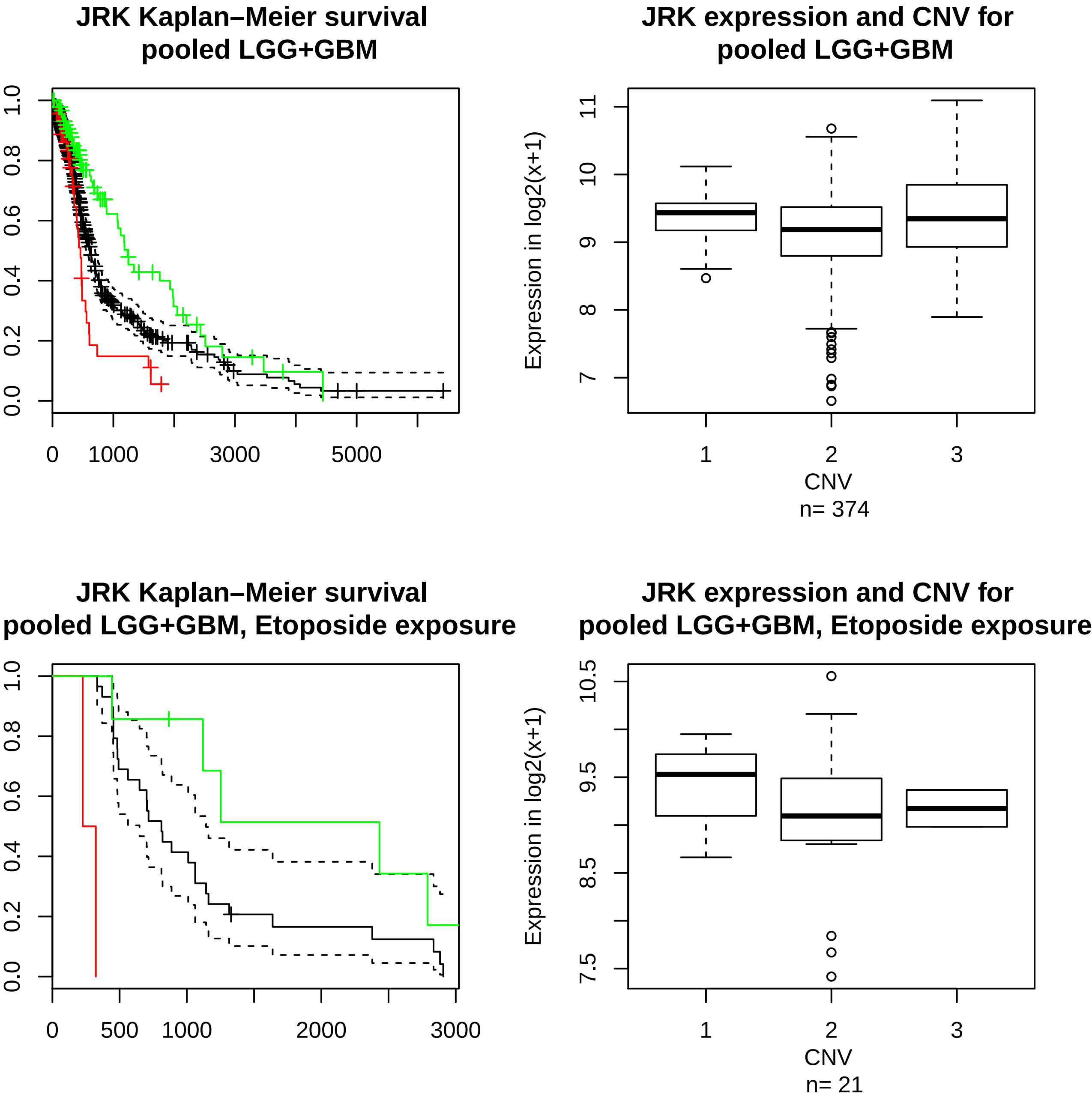

JRK Kaplan–Meier survival
pooled LGG+GBM
0.0 0.2 0.4 0.6 0.8 1.0
0 1000 3000 5000
JRK expression and CNV for
pooled LGG+GBM
Expression in log2(x+1)
7 8 9 10 11
1 2 3
CNV
n= 374
JRK Kaplan–Meier survival
pooled LGG+GBM, Etoposide exposure
0.0 0.2 0.4 0.6 0.8 1.0
0 500 1000 2000 3000
JRK expression and CNV for
pooled LGG+GBM, Etoposide exposure
Expression in log2(x+1)
7.5 8.5 9.5 10.5
1 2 3
CNV
n= 21

PSCA Kaplan–Meier survival
pooled LGG+GBM

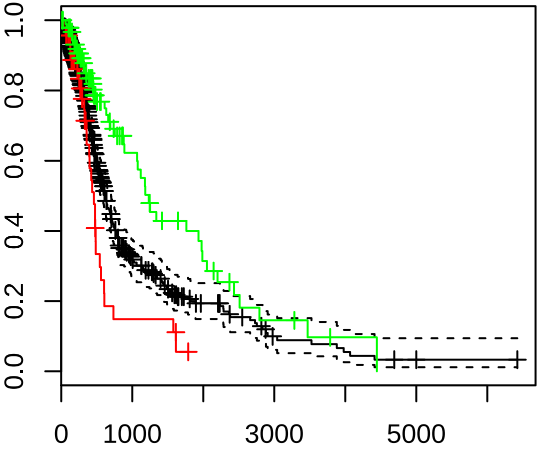

PSCA expression and CNV for
pooled LGG+GBM

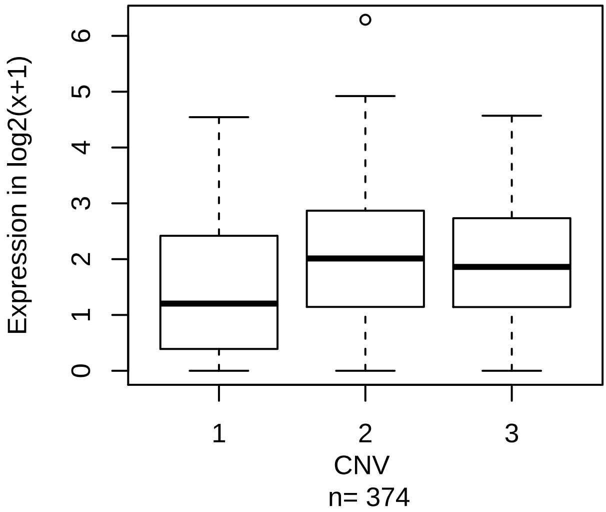

PSCA Kaplan–Meier survival
pooled LGG+GBM, Etoposide exposure

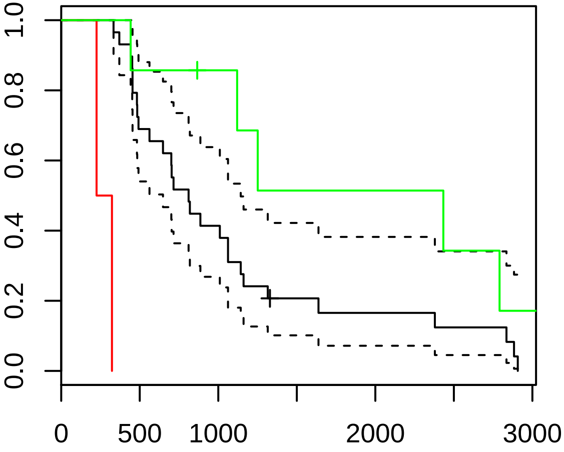

PSCA expression and CNV for
pooled LGG+GBM, Etoposide exposure

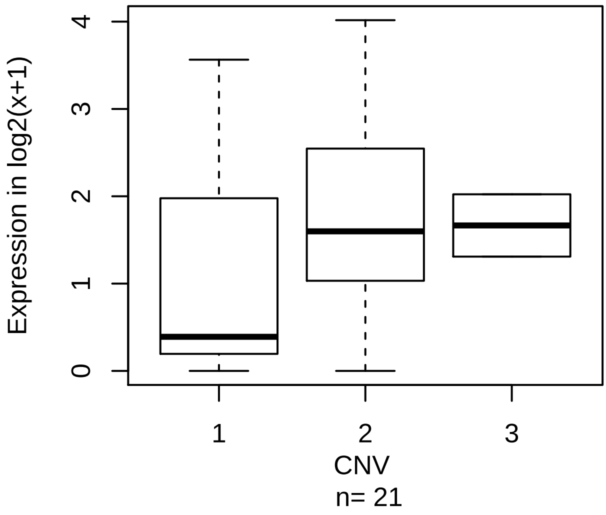

LY6K Kaplan–Meier survival pooled LGG+GBM

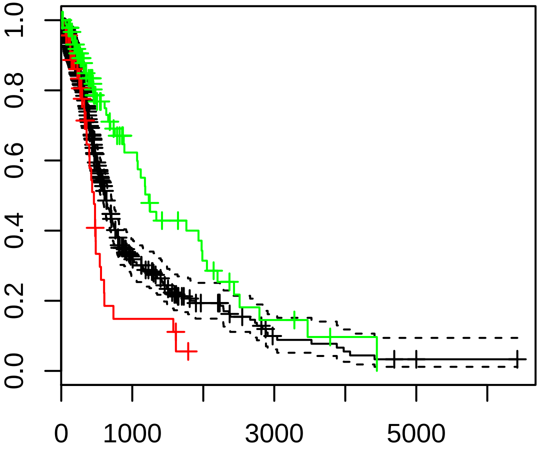

LY6K expression and CNV for pooled LGG+GBM

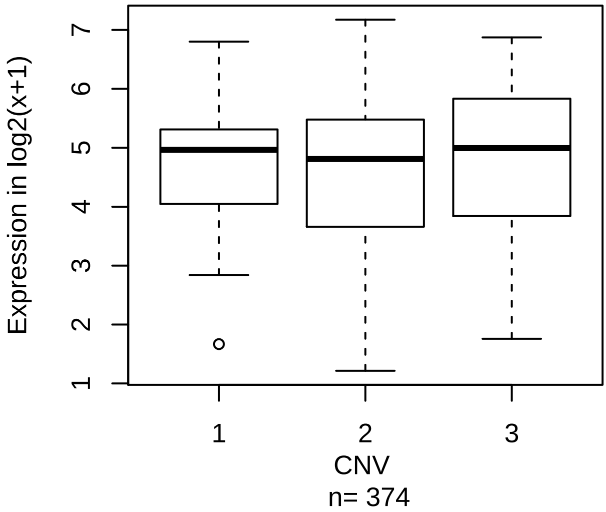

LY6K Kaplan–Meier survival pooled LGG+GBM, Etoposide exposure

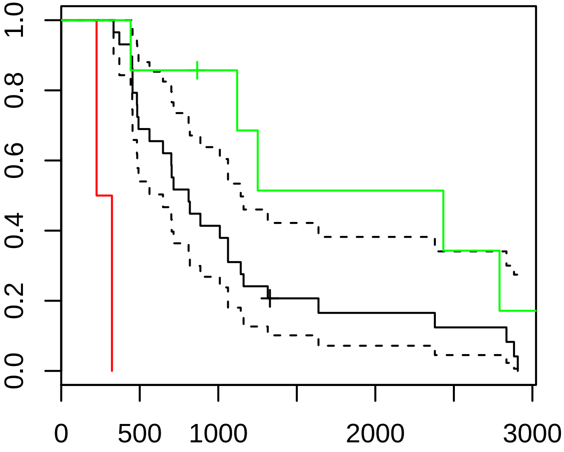

LY6K expression and CNV for pooled LGG+GBM, Etoposide exposure

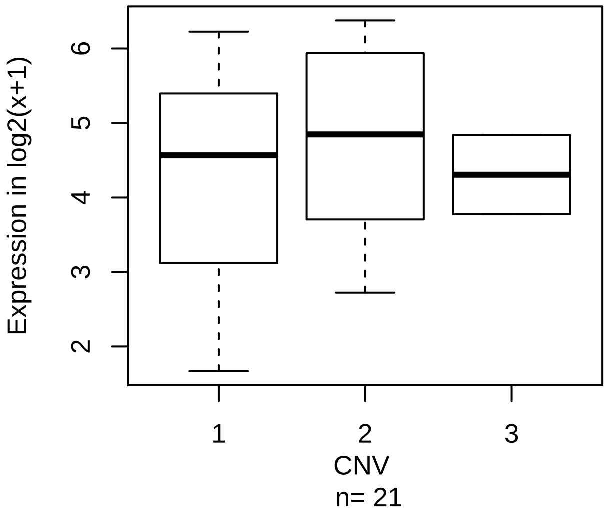

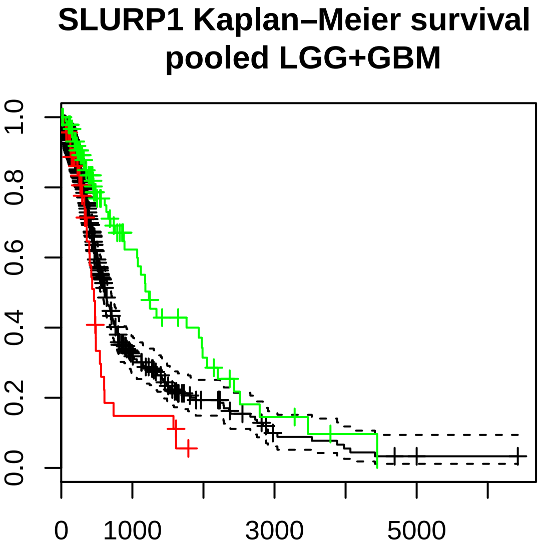
SLURP1 Kaplan–Meier survival
pooled LGG+GBM
0.0
0.2
0.4
0.6
0.8
1.0
0
1000
3000
5000

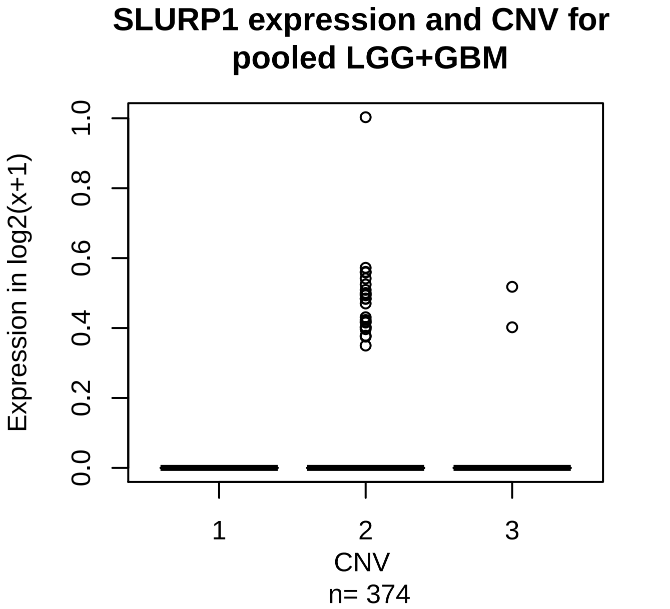
SLURP1 expression and CNV for
pooled LGG+GBM
Expression in log2(x+1)
0.0
0.2
0.4
0.6
0.8
1.0
1
2
3
CNV
n= 374

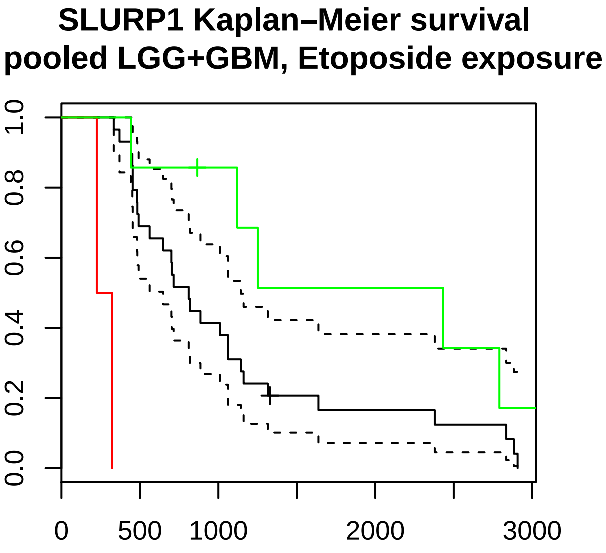
SLURP1 Kaplan–Meier survival
pooled LGG+GBM, Etoposide exposure
0.0
0.2
0.4
0.6
0.8
1.0
0
500
1000
2000
3000

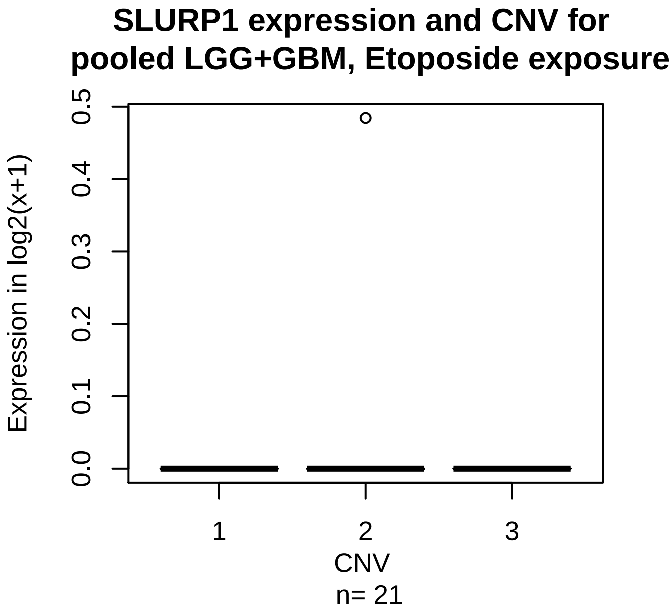
SLURP1 expression and CNV for
pooled LGG+GBM, Etoposide exposure
Expression in log2(x+1)
0.0
0.1
0.2
0.3
0.4
0.5
1
2
3
CNV
n= 21

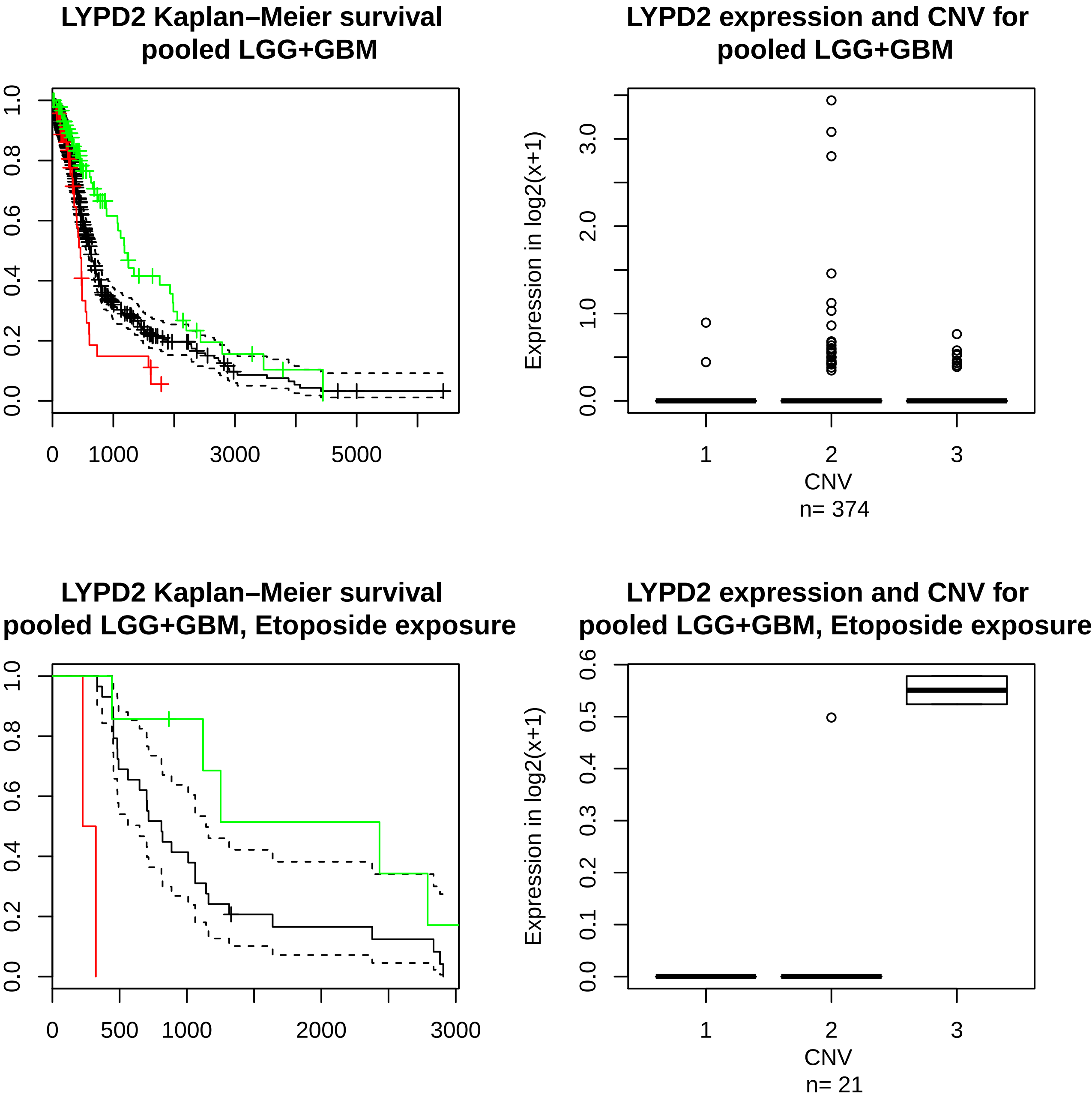
LYPD2 Kaplan–Meier survival
pooled LGG+GBM
0.0
0.2
0.4
0.6
0.8
1.0
0
1000
3000
5000
LYPD2 expression and CNV for
pooled LGG+GBM
Expression in log2(x+1)
0.0
1.0
2.0
3.0
1
2
3
CNV
n= 374
LYPD2 Kaplan–Meier survival
pooled LGG+GBM, Etoposide exposure
0.0
0.2
0.4
0.6
0.8
1.0
0
500
1000
2000
3000
LYPD2 expression and CNV for
pooled LGG+GBM, Etoposide exposure
Expression in log2(x+1)
0.0
0.1
0.2
0.3
0.4
0.5
0.6
1
2
3
CNV
n= 21

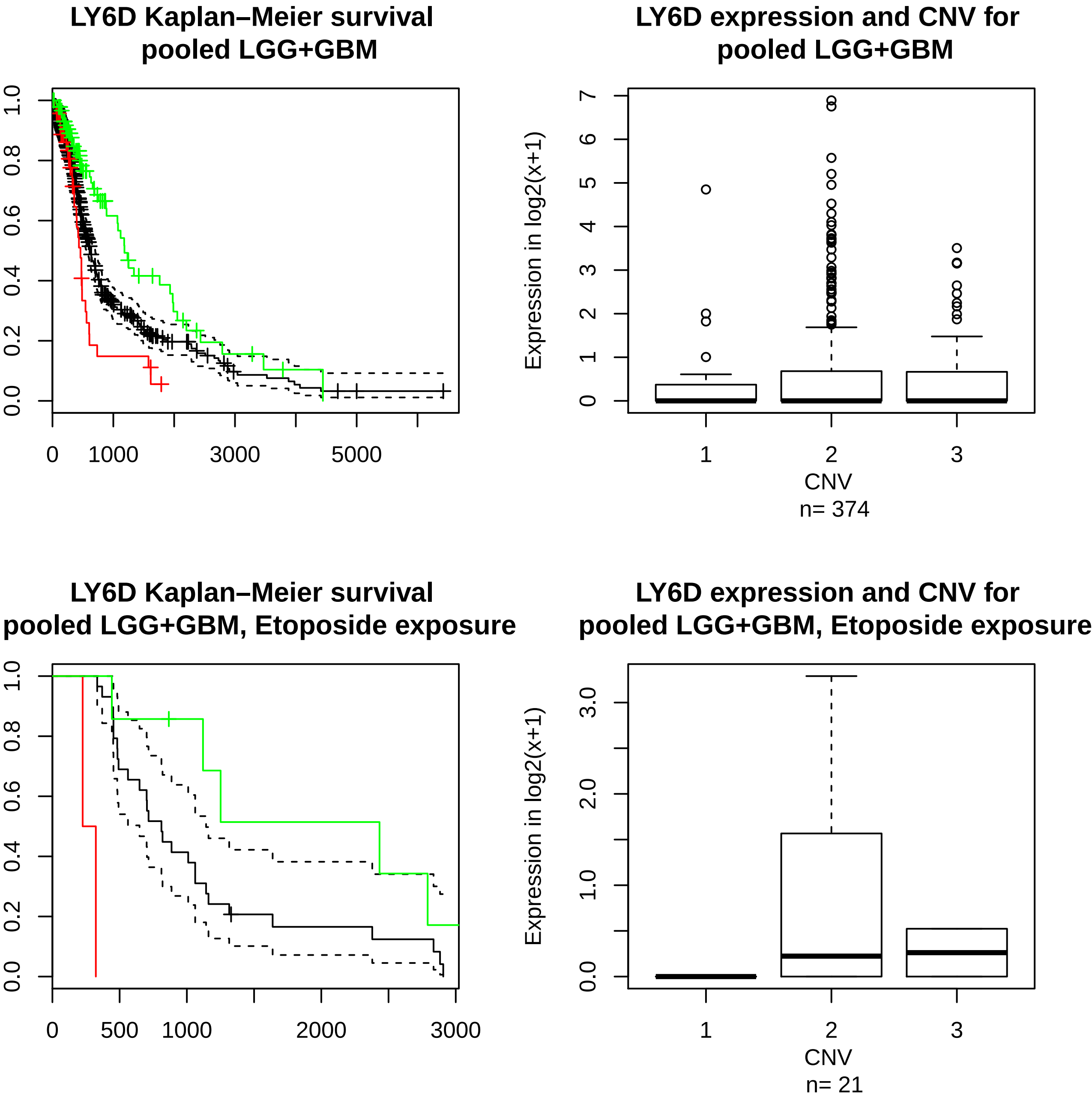
LY6D Kaplan–Meier survival
pooled LGG+GBM
0.0
0.2
0.4
0.6
0.8
1.0
0
1000
3000
5000
LY6D expression and CNV for
pooled LGG+GBM
Expression in log2(x+1)
0
1
2
3
4
5
6
7
1
2
3
CNV
n= 374
LY6D Kaplan–Meier survival
pooled LGG+GBM, Etoposide exposure
0.0
0.2
0.4
0.6
0.8
1.0
0
500
1000
2000
3000
LY6D expression and CNV for
pooled LGG+GBM, Etoposide exposure
Expression in log2(x+1)
0.0
1.0
2.0
3.0
1
2
3
CNV
n= 21

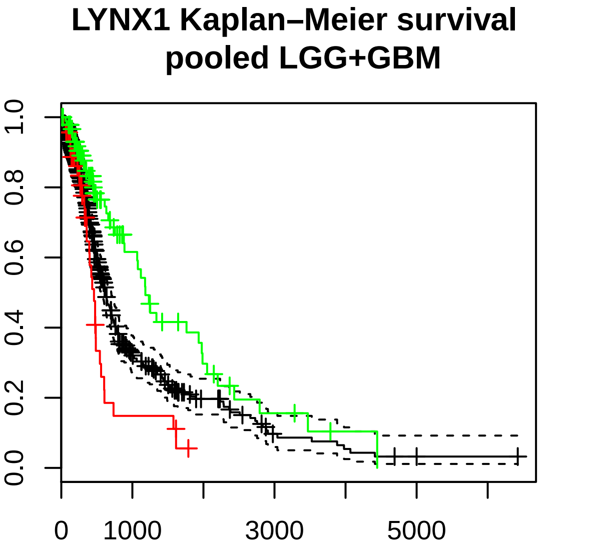
LYNX1 Kaplan–Meier survival
pooled LGG+GBM
0.0
0.2
0.4
0.6
0.8
1.0
0
1000
3000
5000

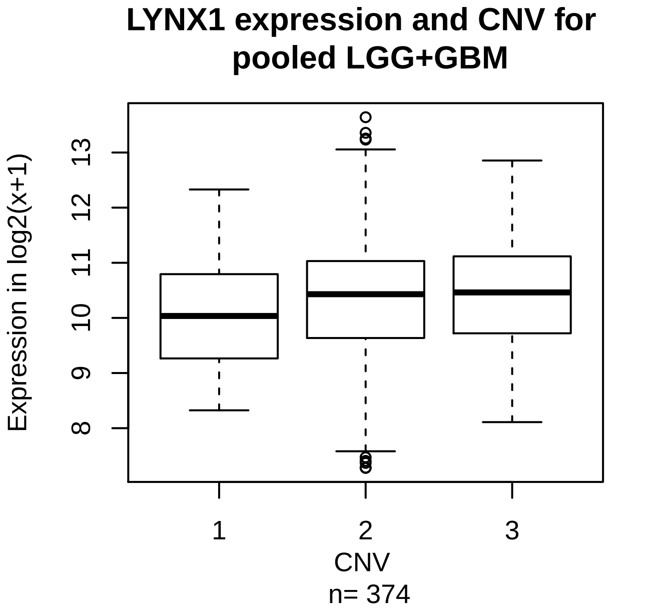
LYNX1 expression and CNV for
pooled LGG+GBM
Expression in log2(x+1)
8
9
10
11
12
13
1
2
3
CNV
n= 374

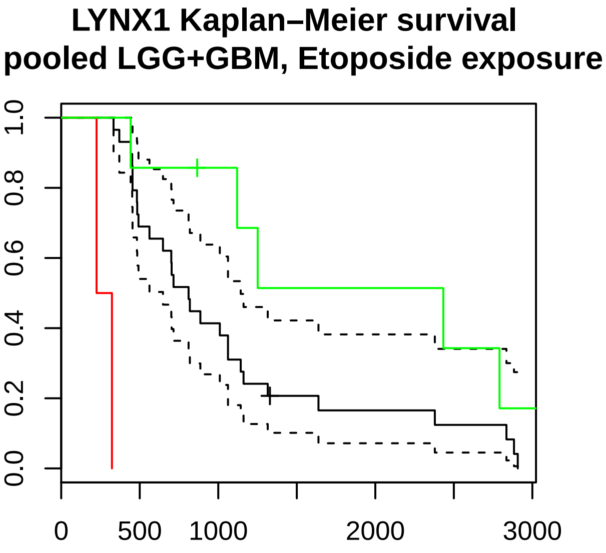
LYNX1 Kaplan–Meier survival
pooled LGG+GBM, Etoposide exposure
0.0
0.2
0.4
0.6
0.8
1.0
0
500
1000
2000
3000

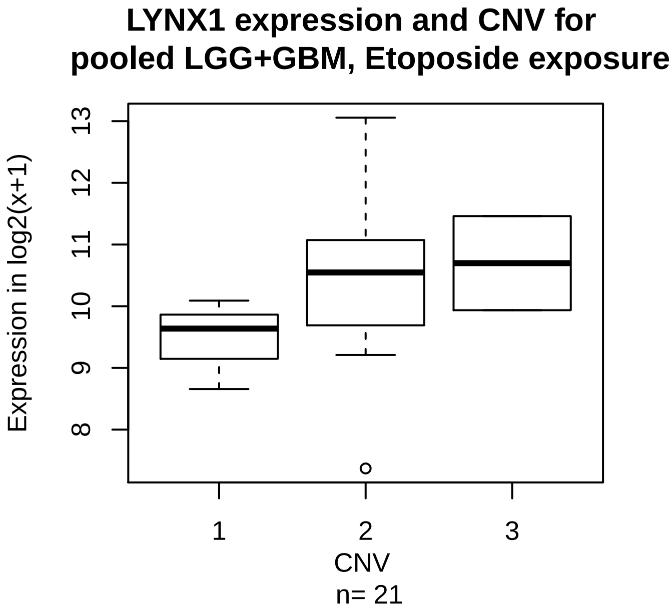
LYNX1 expression and CNV for
pooled LGG+GBM, Etoposide exposure
Expression in log2(x+1)
8
9
10
11
12
13
1
2
3
CNV
n= 21

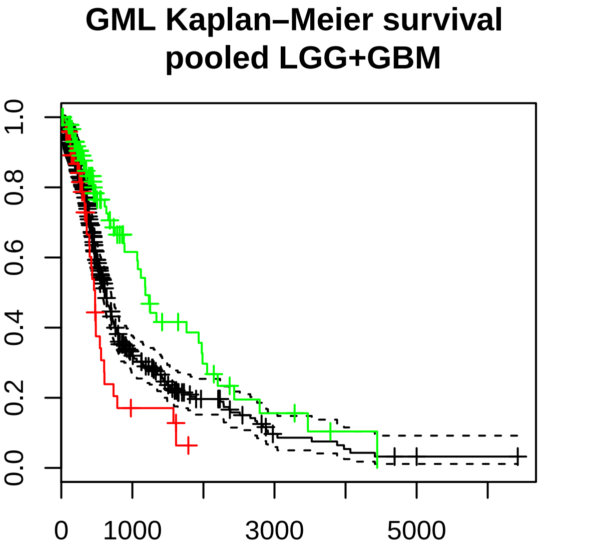
GML Kaplan–Meier survival
pooled LGG+GBM
0.0
0.2
0.4
0.6
0.8
1.0
0
1000
3000
5000

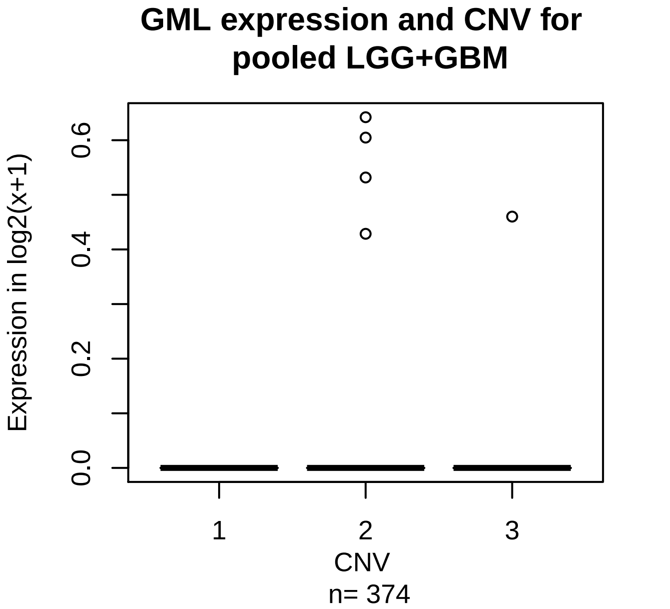
GML expression and CNV for
pooled LGG+GBM
Expression in log2(x+1)
0.0
0.2
0.4
0.6
1
2
3
CNV
n= 374

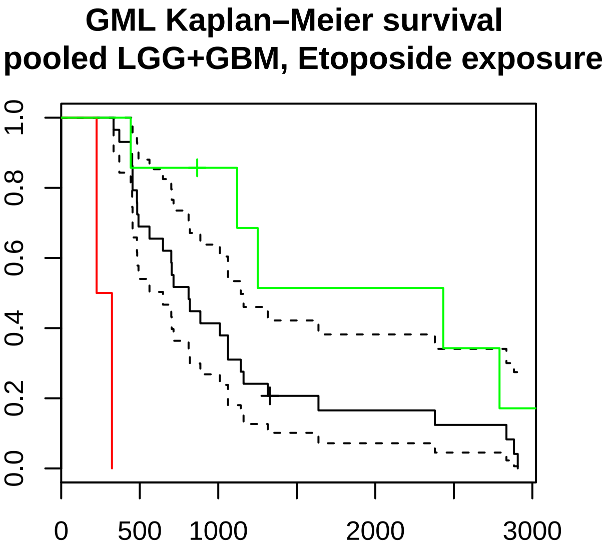
GML Kaplan–Meier survival
pooled LGG+GBM, Etoposide exposure
0.0
0.2
0.4
0.6
0.8
1.0
0
500
1000
2000
3000

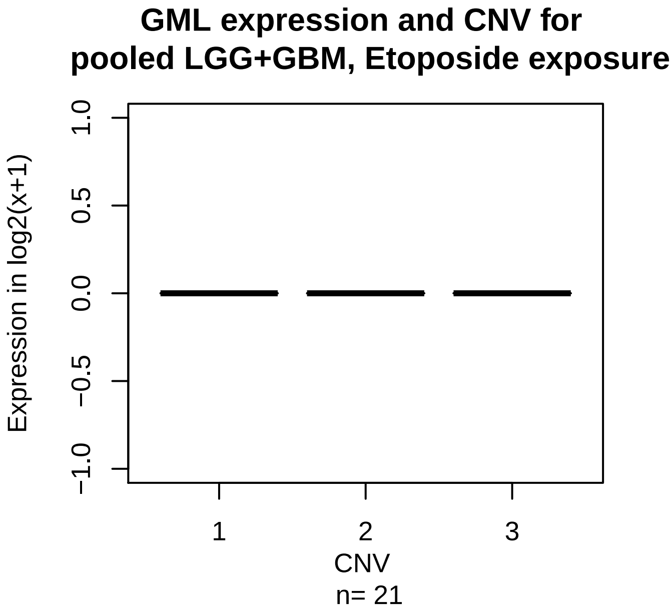
GML expression and CNV for
pooled LGG+GBM, Etoposide exposure
Expression in log2(x+1)
−1.0
−0.5
0.0
0.5
1.0
1
2
3
CNV
n= 21

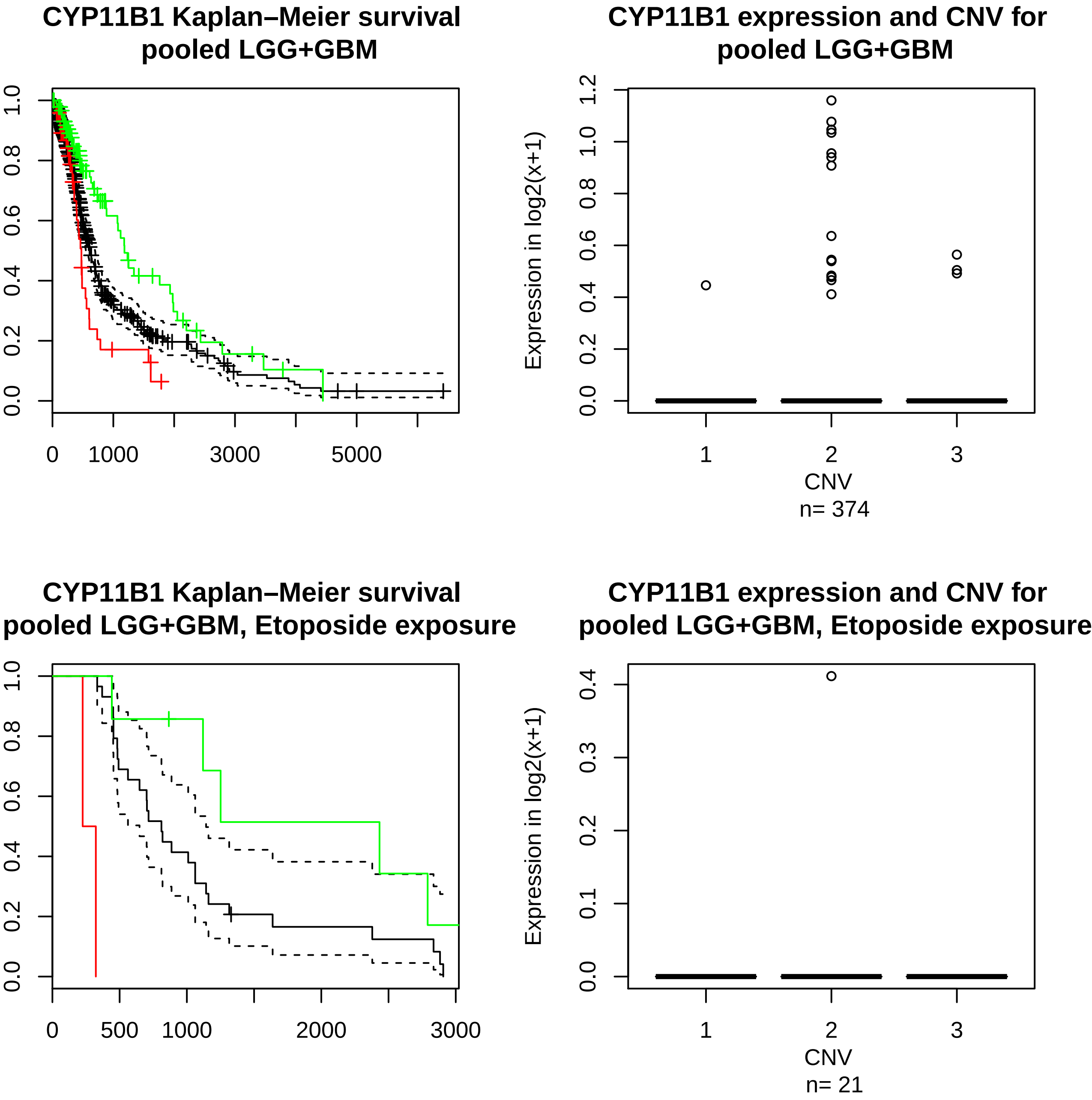

CYP11B1 Kaplan–Meier survival
pooled LGG+GBM
0.0
0.2
0.4
0.6
0.8
1.0
0
1000
3000
5000
CYP11B1 expression and CNV for
pooled LGG+GBM
Expression in log2(x+1)
0.0
0.2
0.4
0.6
0.8
1.0
1.2
1
2
3
CNV
n= 374
CYP11B1 Kaplan–Meier survival
pooled LGG+GBM, Etoposide exposure
0.0
0.2
0.4
0.6
0.8
1.0
0
500
1000
2000
3000
CYP11B1 expression and CNV for
pooled LGG+GBM, Etoposide exposure
Expression in log2(x+1)
0.0
0.1
0.2
0.3
0.4
1
2
3
CNV
n= 21

CYP11B2 Kaplan–Meier survival
pooled LGG+GBM

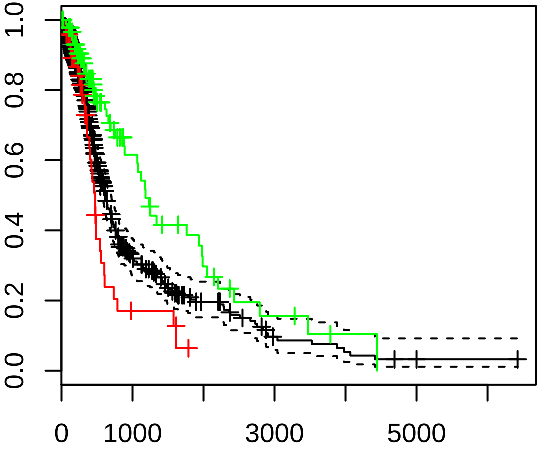

CYP11B2 expression and CNV for
pooled LGG+GBM

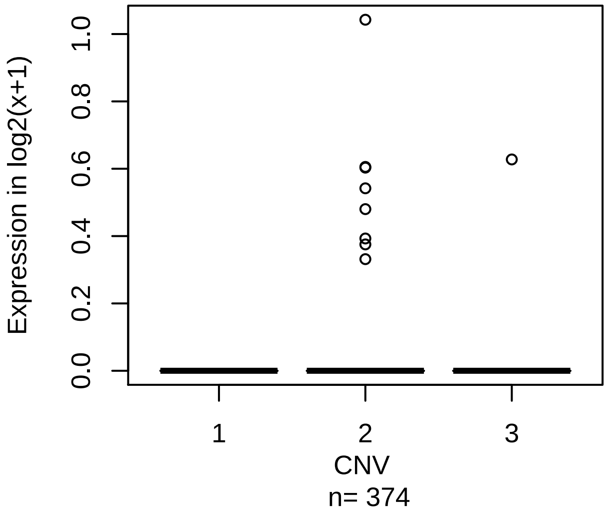

CYP11B2 Kaplan–Meier survival
pooled LGG+GBM, Etoposide exposure

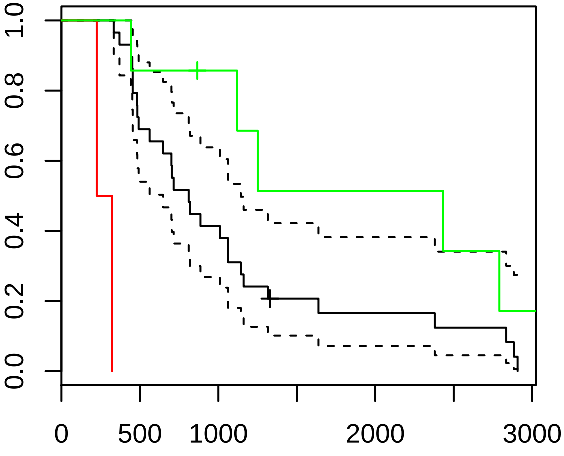

CYP11B2 expression and CNV for
pooled LGG+GBM, Etoposide exposure

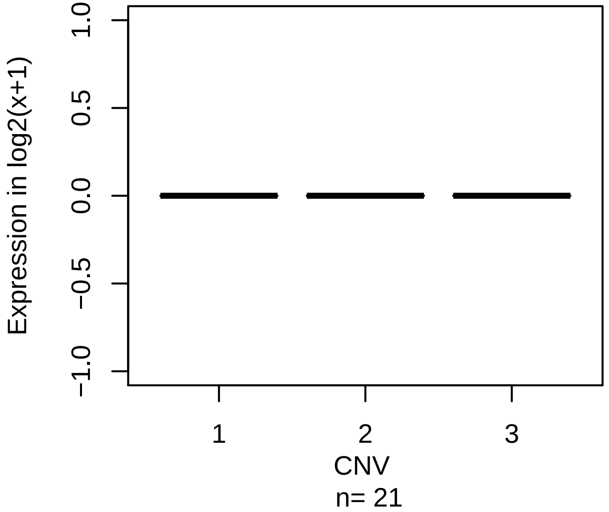

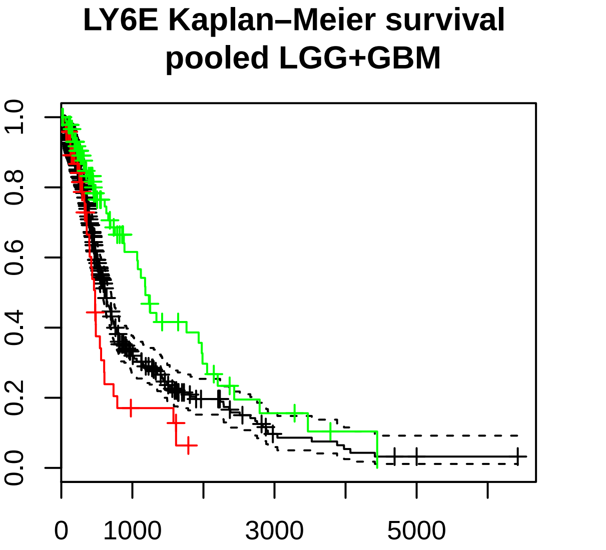
LY6E Kaplan–Meier survival
pooled LGG+GBM
0.0
0.2
0.4
0.6
0.8
1.0
0
1000
3000
5000

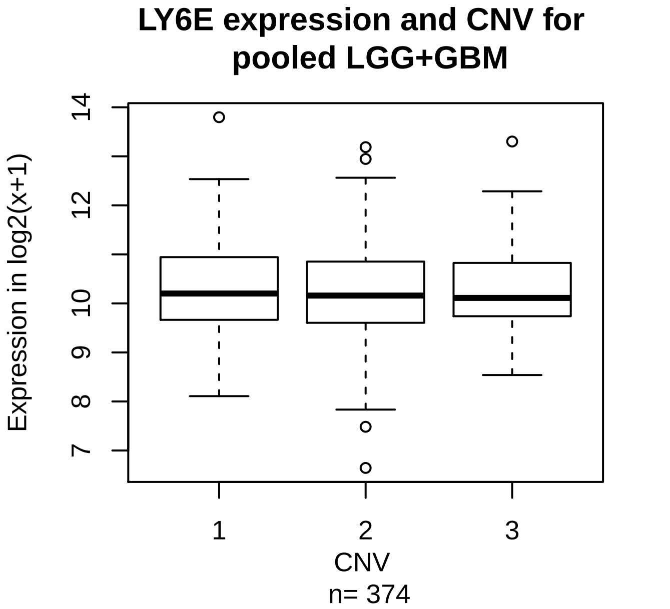
LY6E expression and CNV for
pooled LGG+GBM
Expression in log2(x+1)
7
8
9
10
12
14
1
2
3
CNV
n= 374

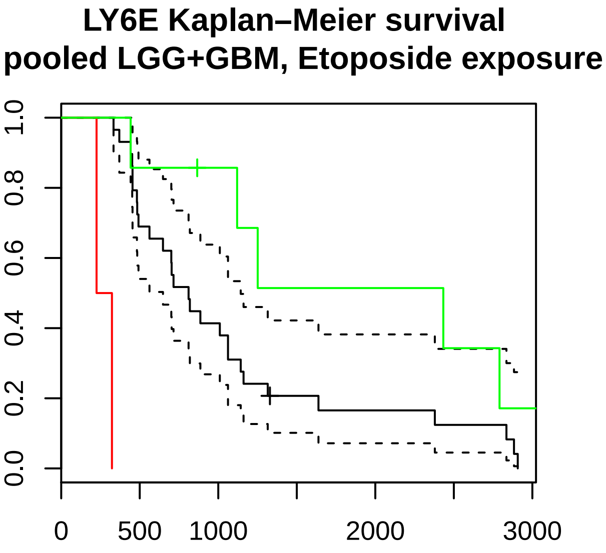
LY6E Kaplan–Meier survival
pooled LGG+GBM, Etoposide exposure
0.0
0.2
0.4
0.6
0.8
1.0
0
500
1000
2000
3000

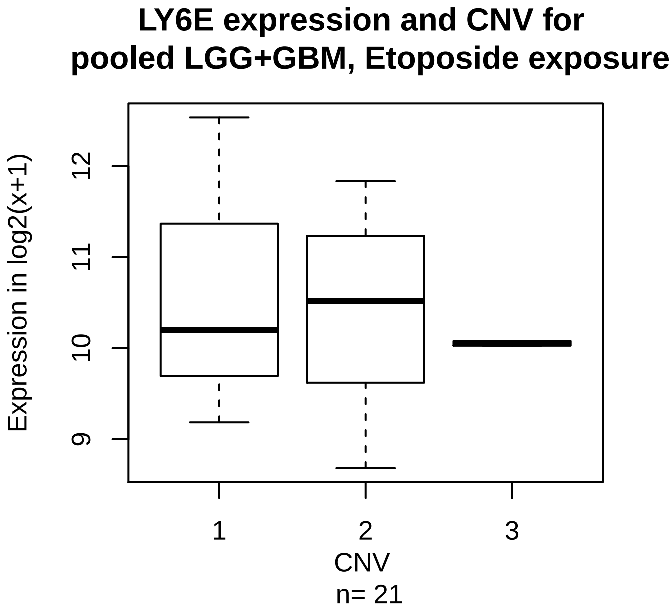
LY6E expression and CNV for
pooled LGG+GBM, Etoposide exposure
Expression in log2(x+1)
9
10
11
12
1
2
3
CNV
n= 21

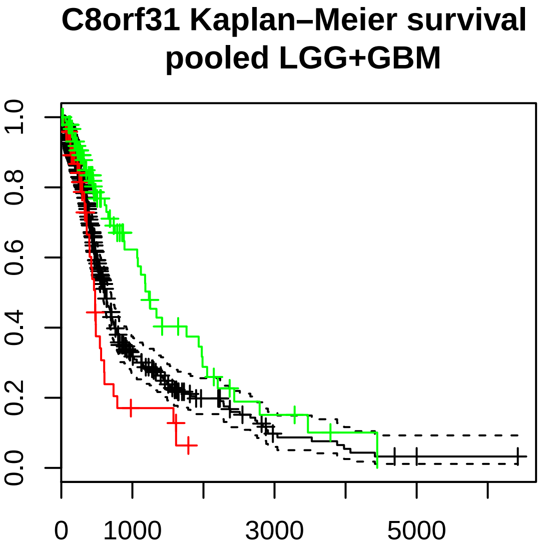
C8orf31 Kaplan–Meier survival
pooled LGG+GBM
0.0 0.2 0.4 0.6 0.8 1.0
0 1000 3000 5000

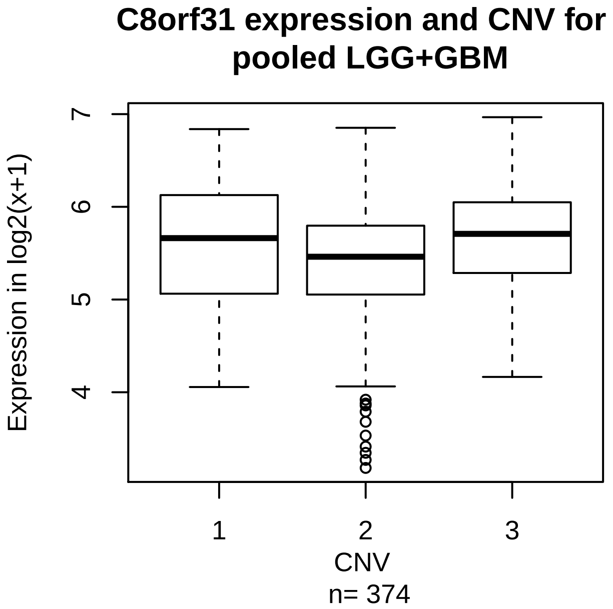
C8orf31 expression and CNV for
pooled LGG+GBM
Expression in log2(x+1)
4 5 6 7
1 2 3
CNV
n= 374

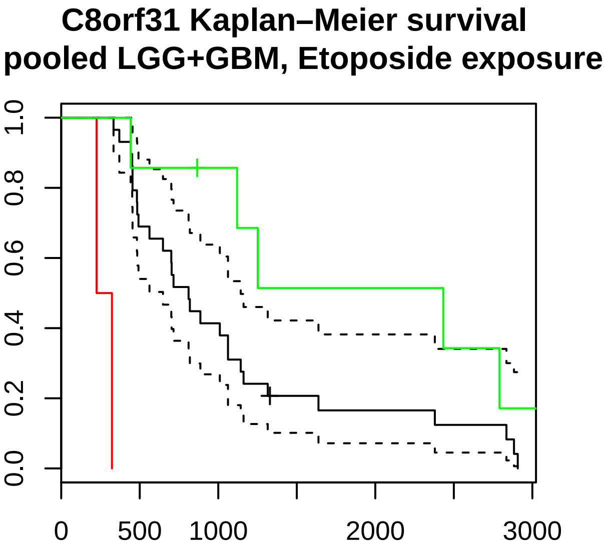
C8orf31 Kaplan–Meier survival
pooled LGG+GBM, Etoposide exposure
0.0 0.2 0.4 0.6 0.8 1.0
0 500 1000 2000 3000

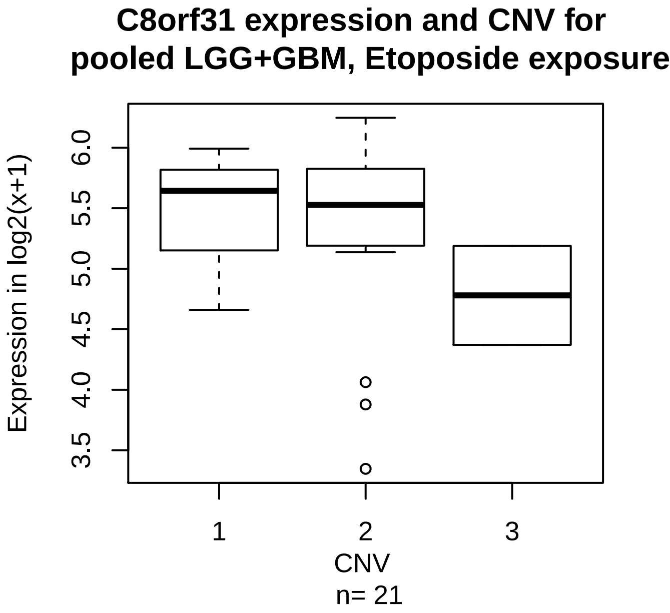
C8orf31 expression and CNV for
pooled LGG+GBM, Etoposide exposure
Expression in log2(x+1)
3.5 4.0 4.5 5.0 5.5 6.0
1 2 3
CNV
n= 21

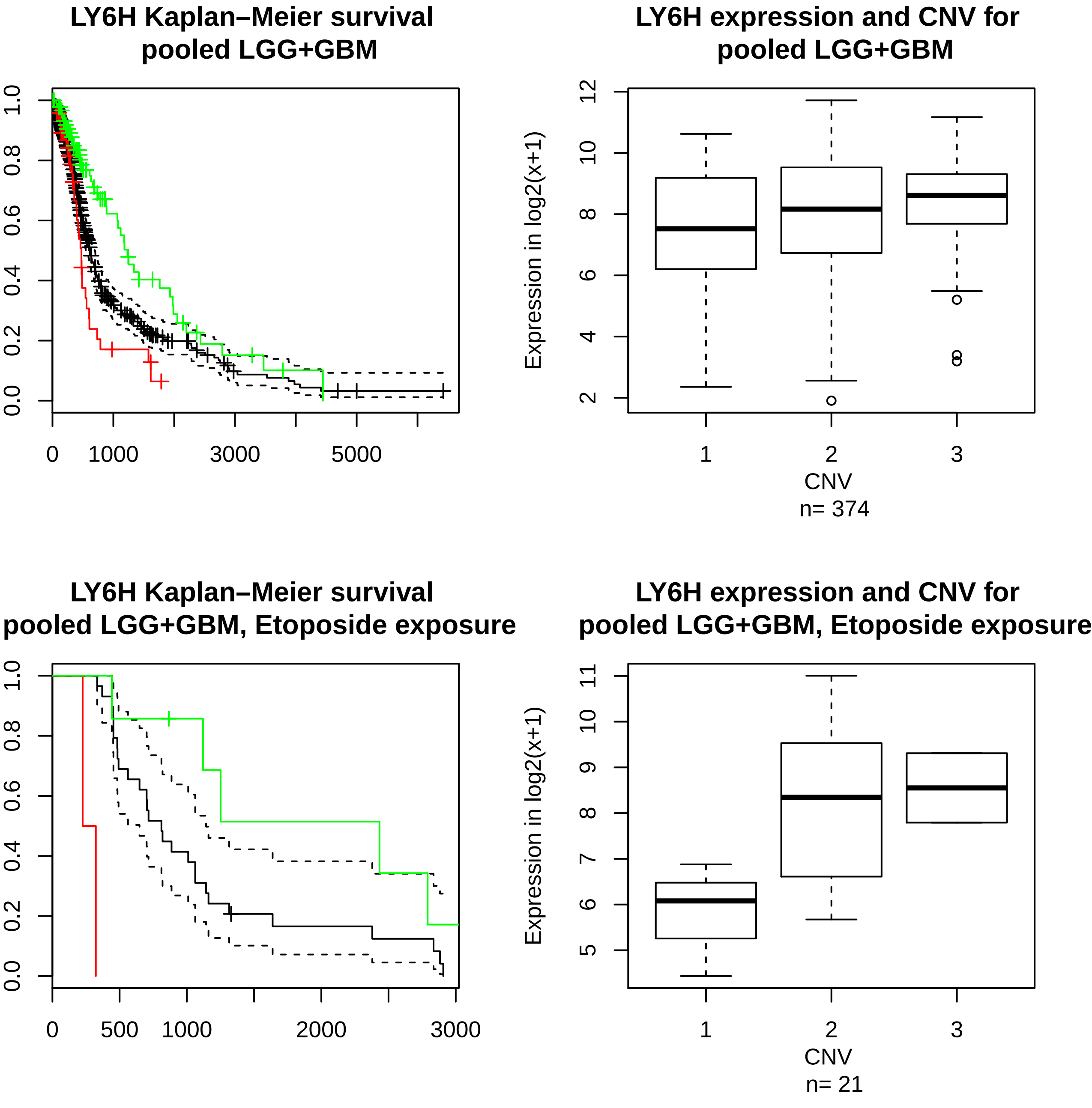
LY6H Kaplan–Meier survival
pooled LGG+GBM
0.0
0.2
0.4
0.6
0.8
1.0
0
1000
3000
5000
LY6H expression and CNV for
pooled LGG+GBM
Expression in log2(x+1)
2
4
6
8
10
12
1
2
3
CNV
n= 374
LY6H Kaplan–Meier survival
pooled LGG+GBM, Etoposide exposure
0.0
0.2
0.4
0.6
0.8
1.0
0
500
1000
2000
3000
LY6H expression and CNV for
pooled LGG+GBM, Etoposide exposure
Expression in log2(x+1)
5
6
7
8
9
10
11
1
2
3
CNV
n= 21

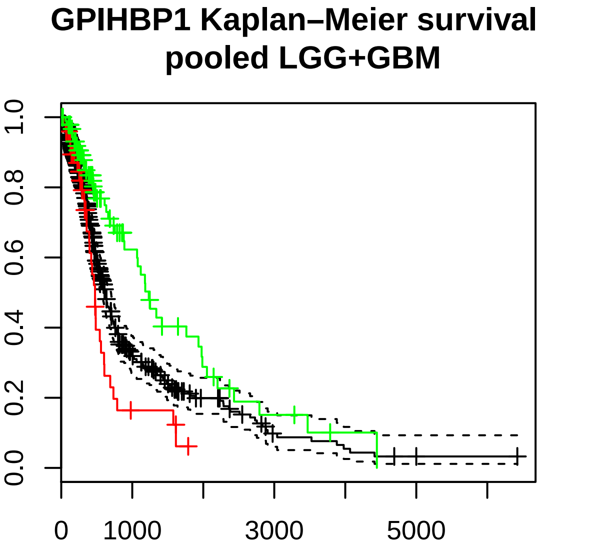
GPIHBP1 Kaplan–Meier survival
pooled LGG+GBM
0.0 0.2 0.4 0.6 0.8 1.0
0 1000 3000 5000

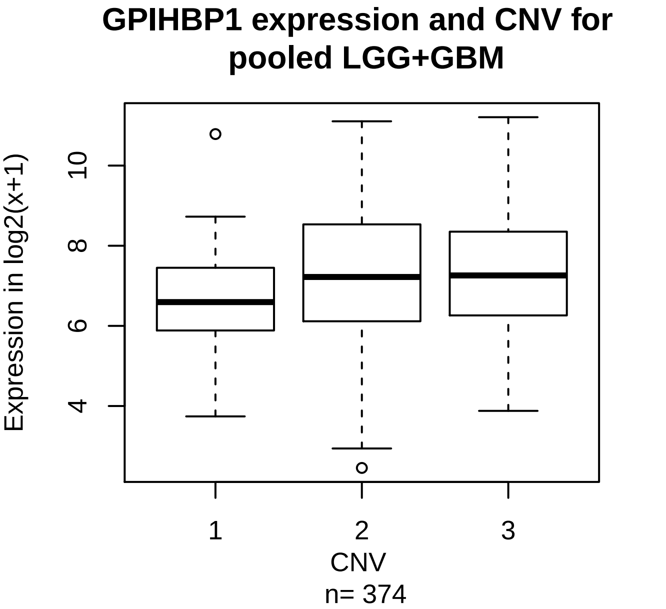
GPIHBP1 expression and CNV for
pooled LGG+GBM
Expression in log2(x+1)
4 6 8 10
1 2 3
CNV
n= 374

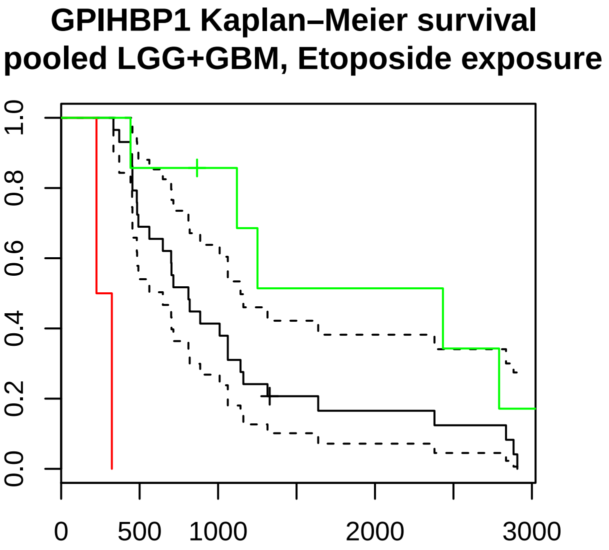
GPIHBP1 Kaplan–Meier survival
pooled LGG+GBM, Etoposide exposure
0.0 0.2 0.4 0.6 0.8 1.0
0 500 1000 2000 3000

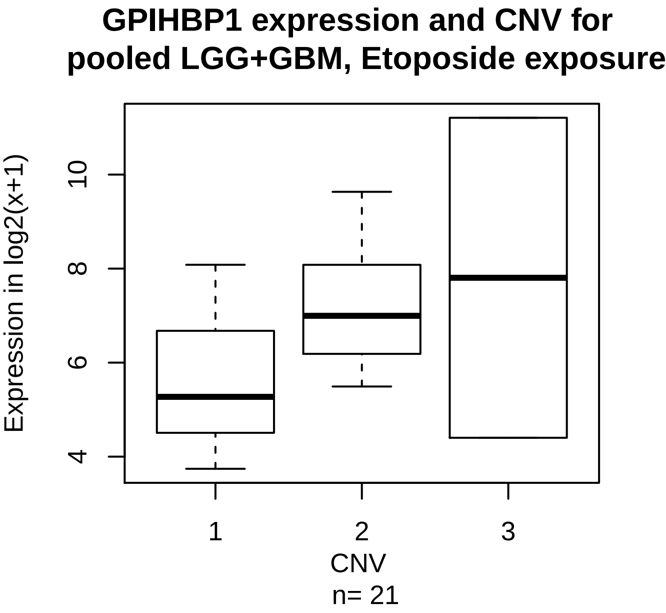
GPIHBP1 expression and CNV for
pooled LGG+GBM, Etoposide exposure
Expression in log2(x+1)
4 6 8 10
1 2 3
CNV
n= 21

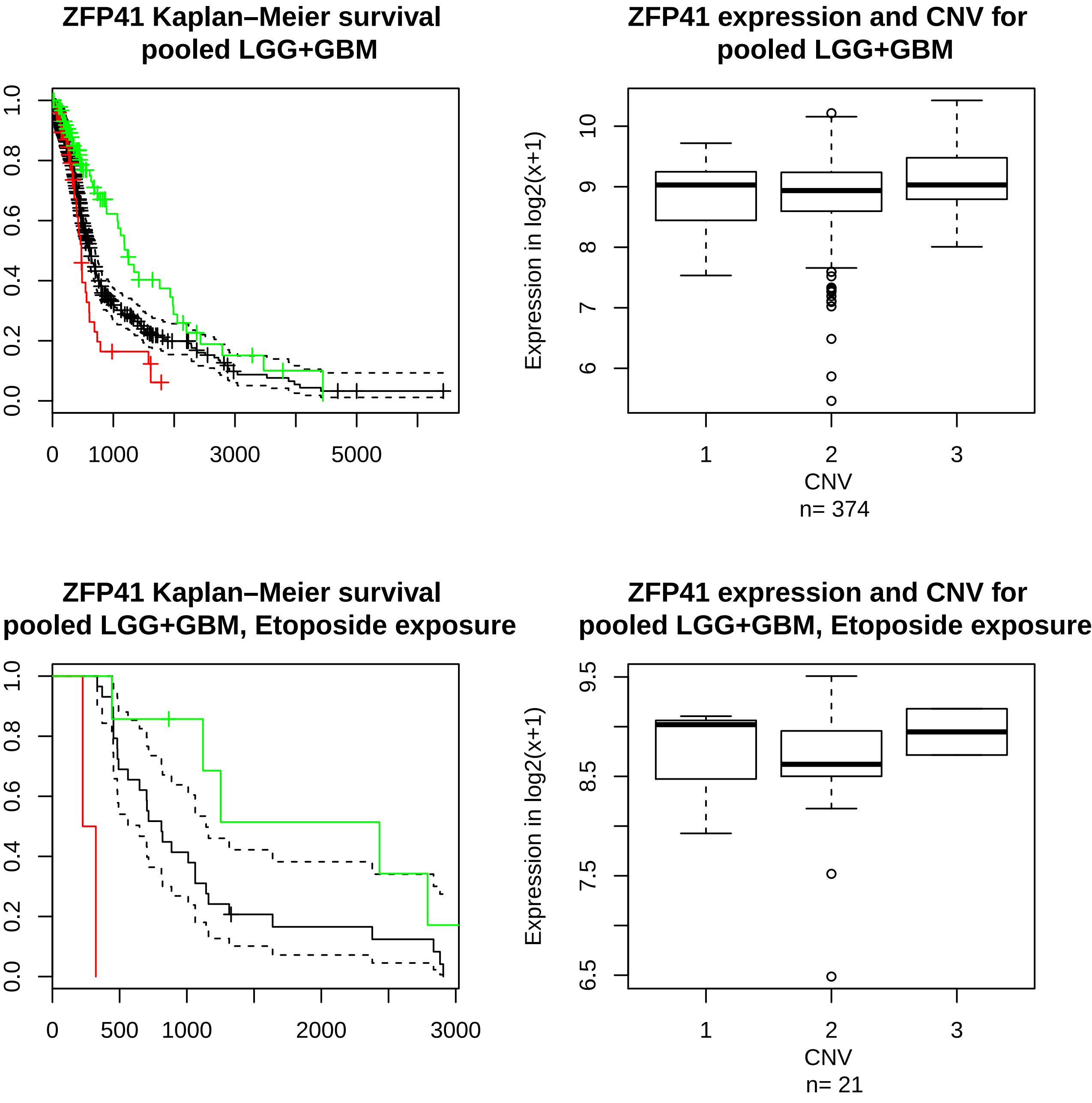
ZFP41 Kaplan–Meier survival
pooled LGG+GBM
0.0
0.2
0.4
0.6
0.8
1.0
0
1000
3000
5000
ZFP41 expression and CNV for
pooled LGG+GBM
Expression in log2(x+1)
6
7
8
9
10
1
2
3
CNV
n= 374
ZFP41 Kaplan–Meier survival
pooled LGG+GBM, Etoposide exposure
0.0
0.2
0.4
0.6
0.8
1.0
0
500
1000
2000
3000
ZFP41 expression and CNV for
pooled LGG+GBM, Etoposide exposure
Expression in log2(x+1)
6.5
7.5
8.5
9.5
1
2
3
CNV
n= 21

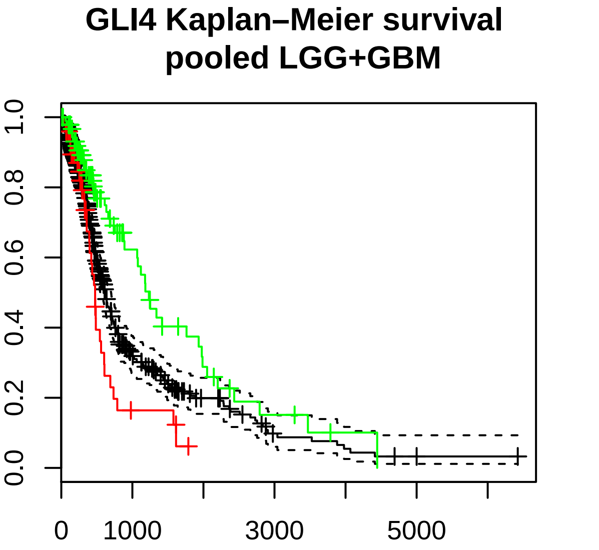

GLI4 Kaplan–Meier survival
pooled LGG+GBM
0.0
0.2
0.4
0.6
0.8
1.0
0
1000
3000
5000

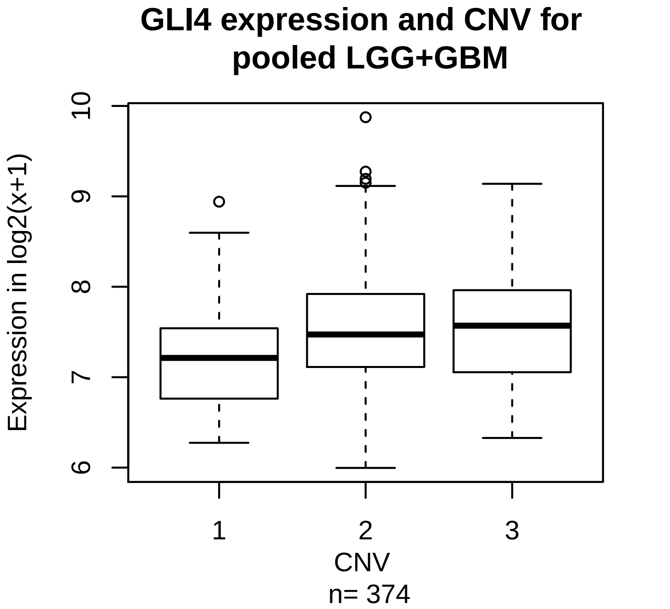

GLI4 expression and CNV for
pooled LGG+GBM
Expression in log2(x+1)
6
7
8
9
10
1
2
3
CNV
n= 374

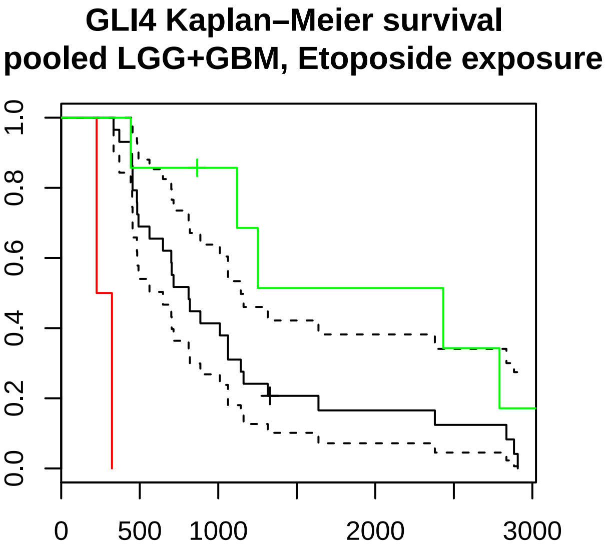

GLI4 Kaplan–Meier survival
pooled LGG+GBM, Etoposide exposure
0.0
0.2
0.4
0.6
0.8
1.0
0
500
1000
2000
3000

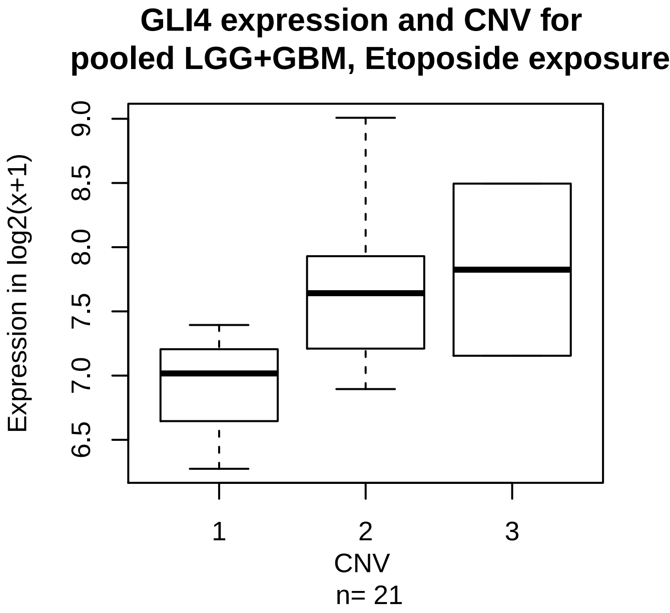

GLI4 expression and CNV for
pooled LGG+GBM, Etoposide exposure
Expression in log2(x+1)
6.5
7.0
7.5
8.0
8.5
9.0
1
2
3
CNV
n= 21

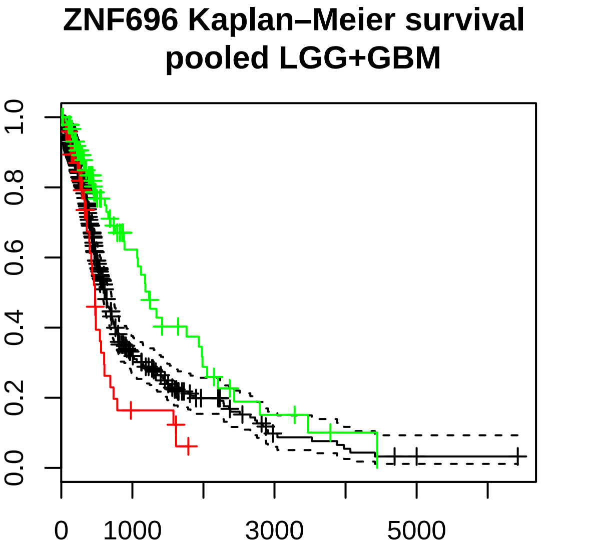
ZNF696 Kaplan–Meier survival
pooled LGG+GBM
0.0
0.2
0.4
0.6
0.8
1.0
0
1000
3000
5000

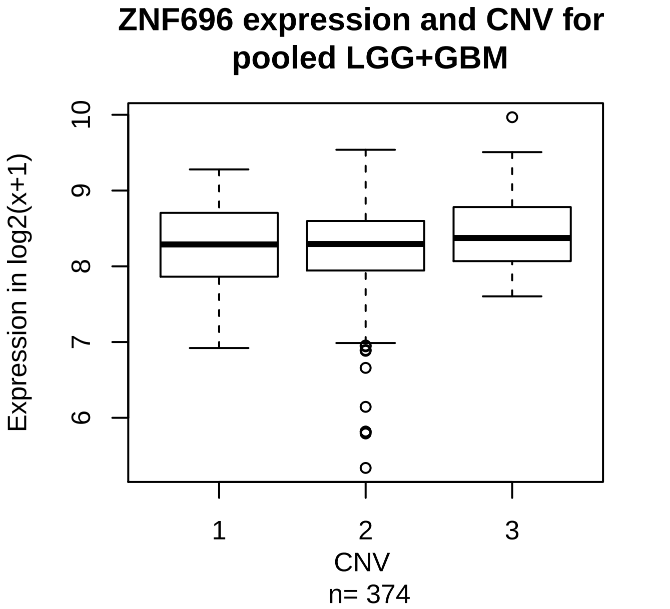
ZNF696 expression and CNV for
pooled LGG+GBM
Expression in log2(x+1)
6
7
8
9
10
1
2
3
CNV
n= 374

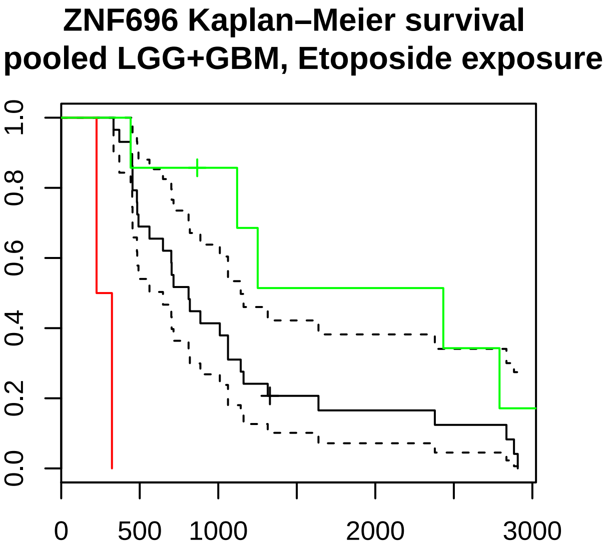
ZNF696 Kaplan–Meier survival
pooled LGG+GBM, Etoposide exposure
0.0
0.2
0.4
0.6
0.8
1.0
0
500
1000
2000
3000

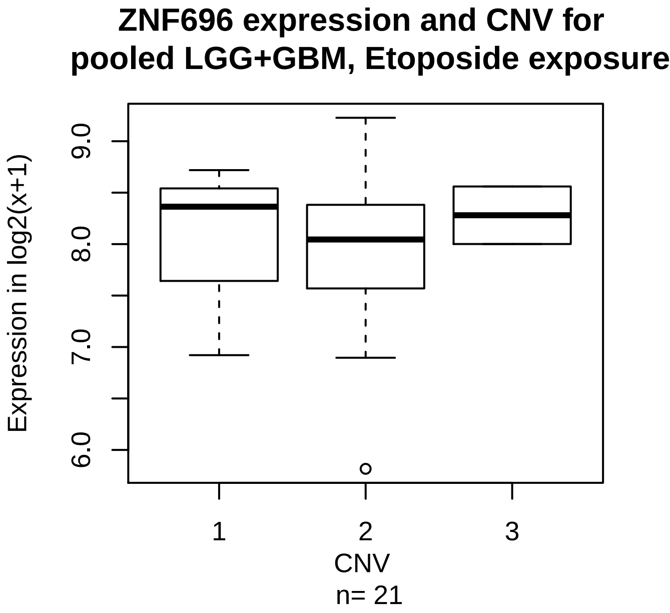
ZNF696 expression and CNV for
pooled LGG+GBM, Etoposide exposure
Expression in log2(x+1)
6.0
7.0
8.0
9.0
1
2
3
CNV
n= 21

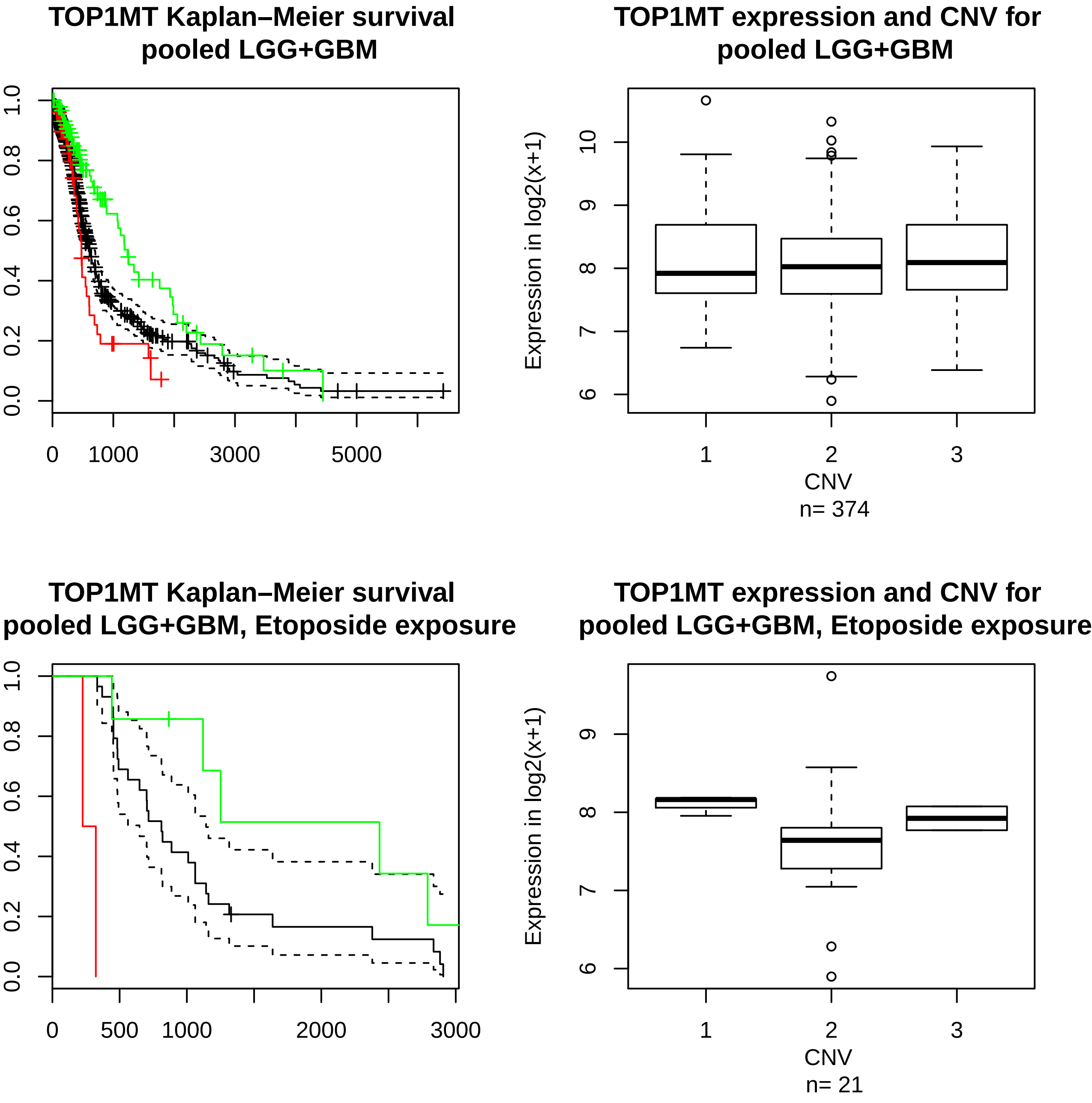
TOP1MT Kaplan–Meier survival
pooled LGG+GBM
0.0
0.2
0.4
0.6
0.8
1.0
0
1000
3000
5000
TOP1MT expression and CNV for
pooled LGG+GBM
Expression in log2(x+1)
6
7
8
9
10
1
2
3
CNV
n= 374
TOP1MT Kaplan–Meier survival
pooled LGG+GBM, Etoposide exposure
0.0
0.2
0.4
0.6
0.8
1.0
0
500
1000
2000
3000
TOP1MT expression and CNV for
pooled LGG+GBM, Etoposide exposure
Expression in log2(x+1)
6
7
8
9
1
2
3
CNV
n= 21

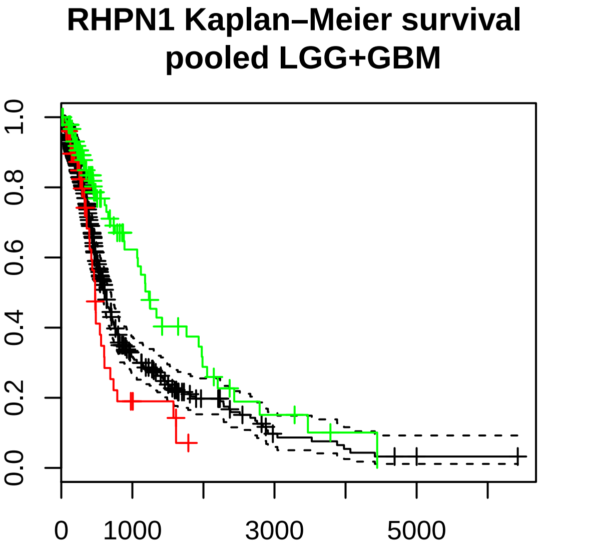
RHPN1 Kaplan–Meier survival
pooled LGG+GBM
0.0
0.2
0.4
0.6
0.8
1.0
0
1000
3000
5000

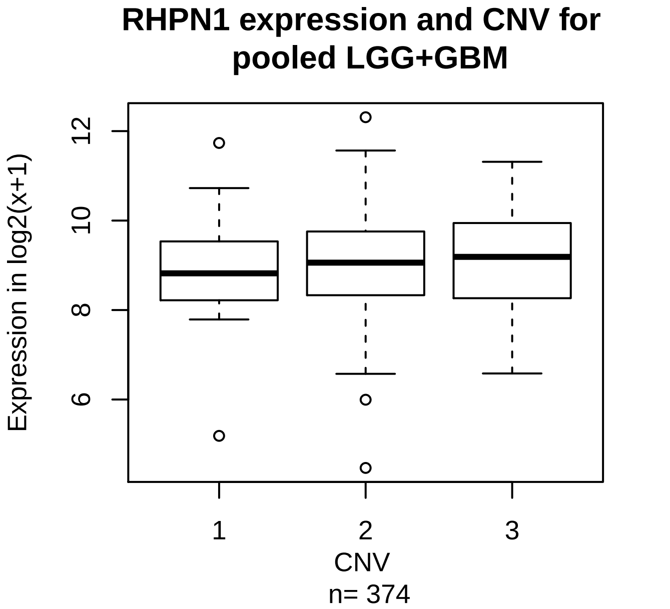
RHPN1 expression and CNV for
pooled LGG+GBM
Expression in log2(x+1)
6
8
10
12
1
2
3
CNV
n= 374

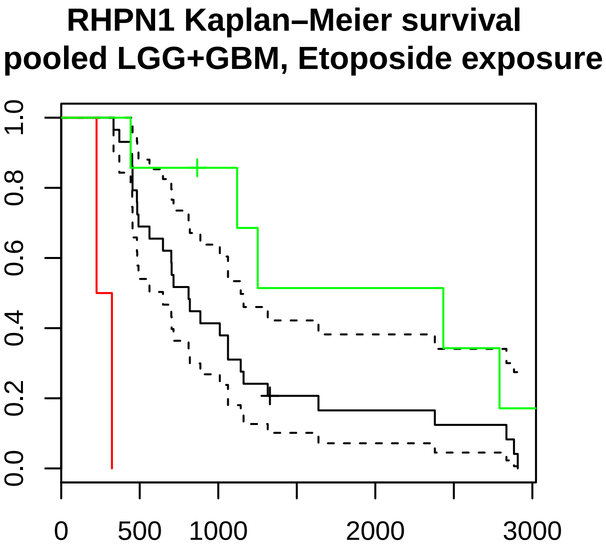
RHPN1 Kaplan–Meier survival
pooled LGG+GBM, Etoposide exposure
0.0
0.2
0.4
0.6
0.8
1.0
0
500
1000
2000
3000

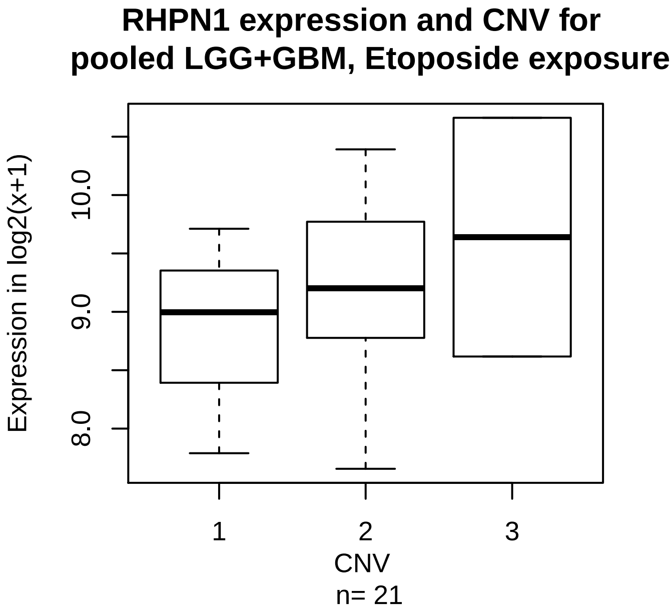
RHPN1 expression and CNV for
pooled LGG+GBM, Etoposide exposure
Expression in log2(x+1)
8.0
9.0
10.0
1
2
3
CNV
n= 21

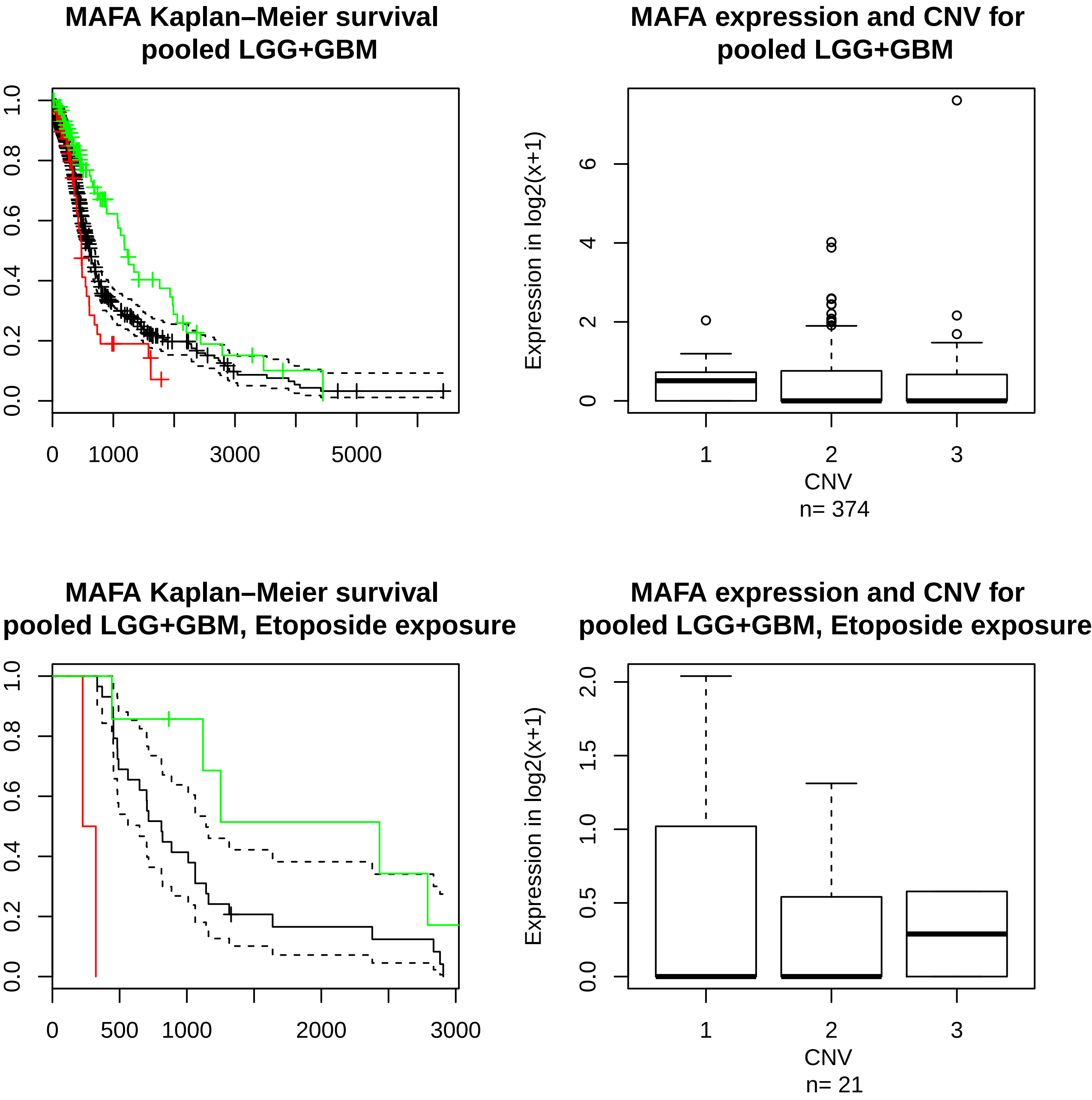
MAFA Kaplan–Meier survival
pooled LGG+GBM
0.0
0.2
0.4
0.6
0.8
1.0
0
1000
3000
5000
MAFA expression and CNV for
pooled LGG+GBM
Expression in log2(x+1)
0
2
4
6
1
2
3
CNV
n= 374
MAFA Kaplan–Meier survival
pooled LGG+GBM, Etoposide exposure
0.0
0.2
0.4
0.6
0.8
1.0
0
500
1000
2000
3000
MAFA expression and CNV for
pooled LGG+GBM, Etoposide exposure
Expression in log2(x+1)
0.0
0.5
1.0
1.5
2.0
1
2
3
CNV
n= 21

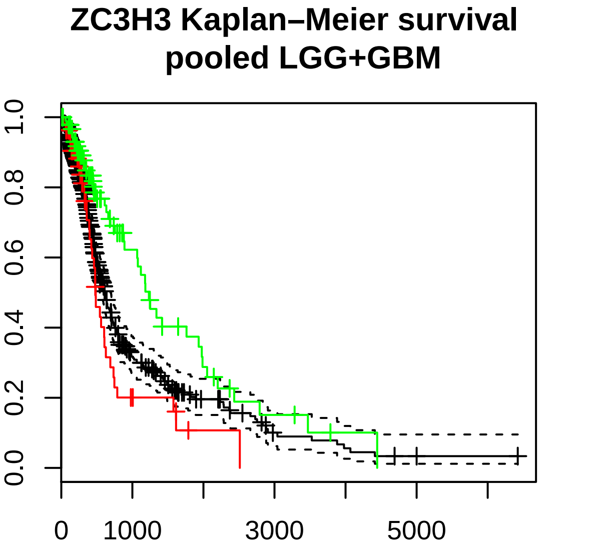
ZC3H3 Kaplan–Meier survival
pooled LGG+GBM
0.0
0.2
0.4
0.6
0.8
1.0
0
1000
3000
5000

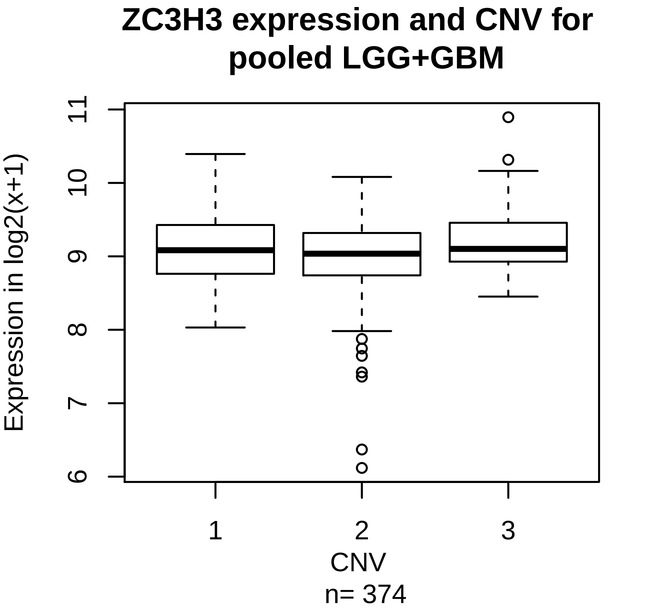
ZC3H3 expression and CNV for
pooled LGG+GBM
Expression in log2(x+1)
6
7
8
9
10
11
1
2
3
CNV
n= 374

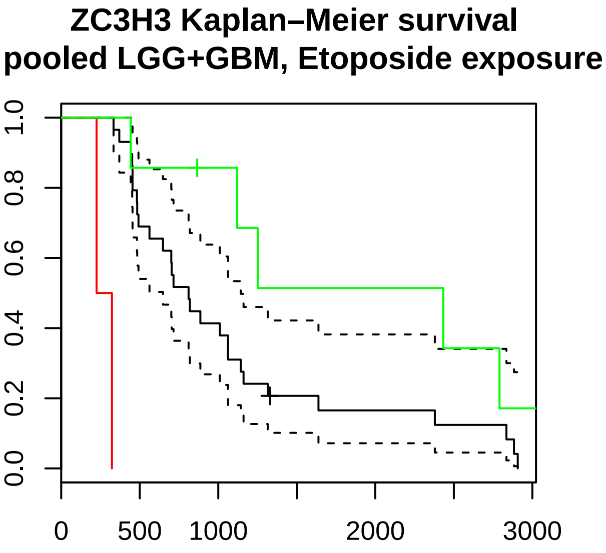
ZC3H3 Kaplan–Meier survival
pooled LGG+GBM, Etoposide exposure
0.0
0.2
0.4
0.6
0.8
1.0
0
500
1000
2000
3000

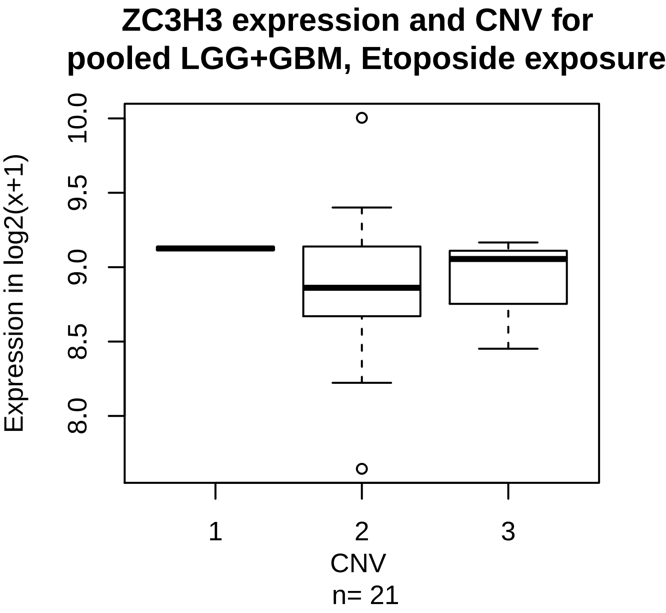
ZC3H3 expression and CNV for
pooled LGG+GBM, Etoposide exposure
Expression in log2(x+1)
8.0
8.5
9.0
9.5
10.0
1
2
3
CNV
n= 21

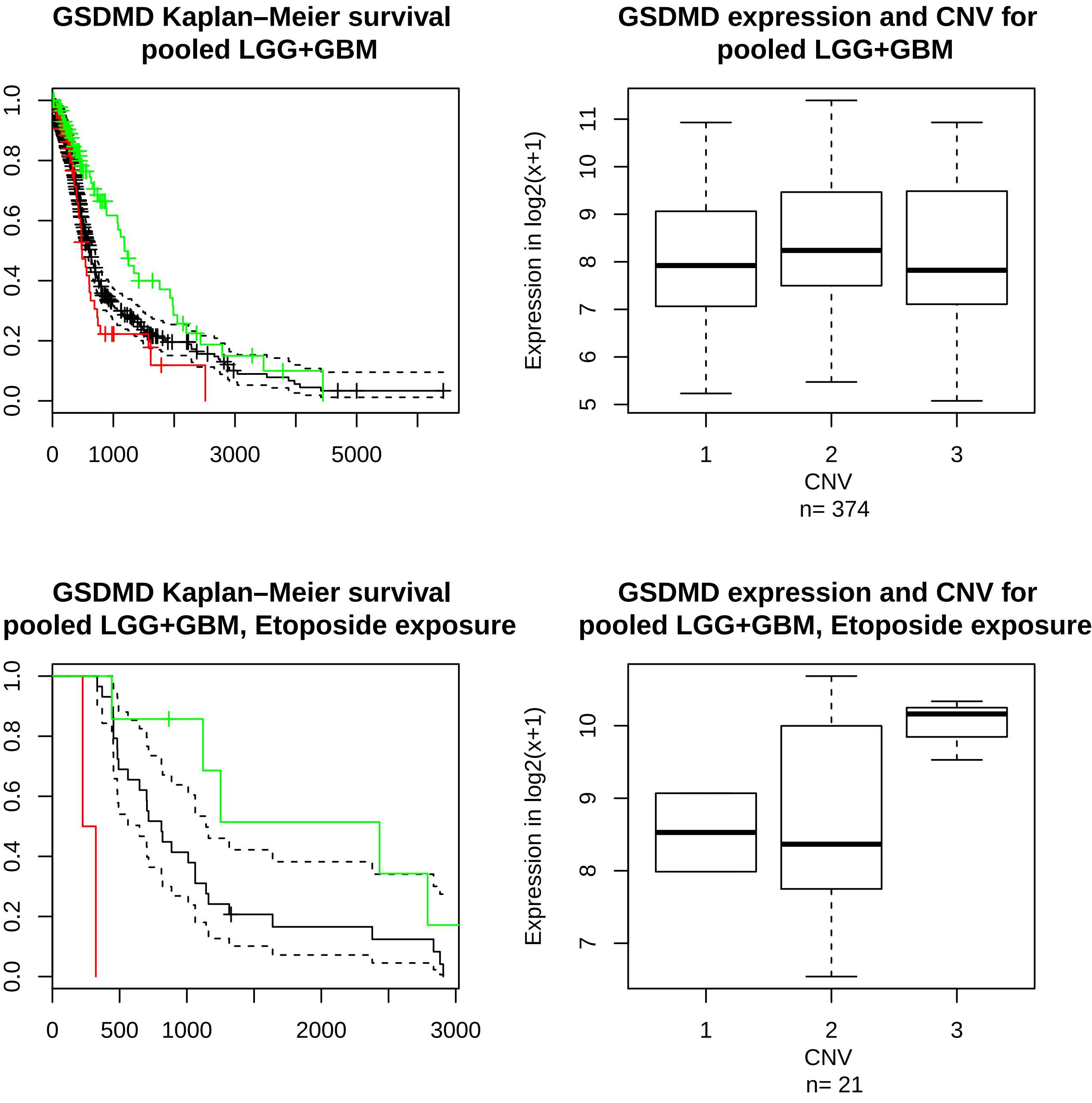
GSDMD Kaplan–Meier survival
pooled LGG+GBM
0.0
0.2
0.4
0.6
0.8
1.0
0
1000
3000
5000
GSDMD expression and CNV for
pooled LGG+GBM
Expression in log2(x+1)
5
6
7
8
9
10
11
1
2
3
CNV
n= 374
GSDMD Kaplan–Meier survival
pooled LGG+GBM, Etoposide exposure
0.0
0.2
0.4
0.6
0.8
1.0
0
500
1000
2000
3000
GSDMD expression and CNV for
pooled LGG+GBM, Etoposide exposure
Expression in log2(x+1)
7
8
9
10
1
2
3
CNV
n= 21

EEF1D Kaplan–Meier survival
pooled LGG+GBM

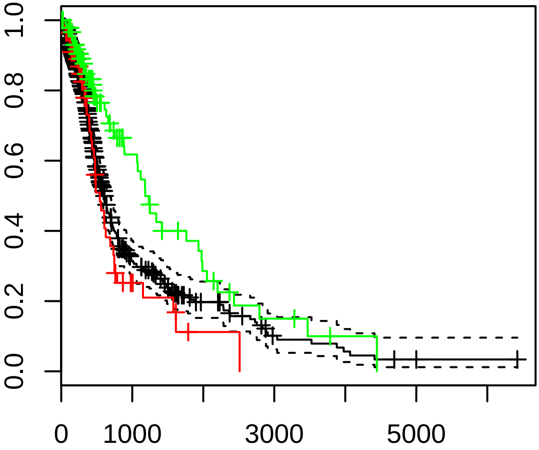

EEF1D expression and CNV for
pooled LGG+GBM

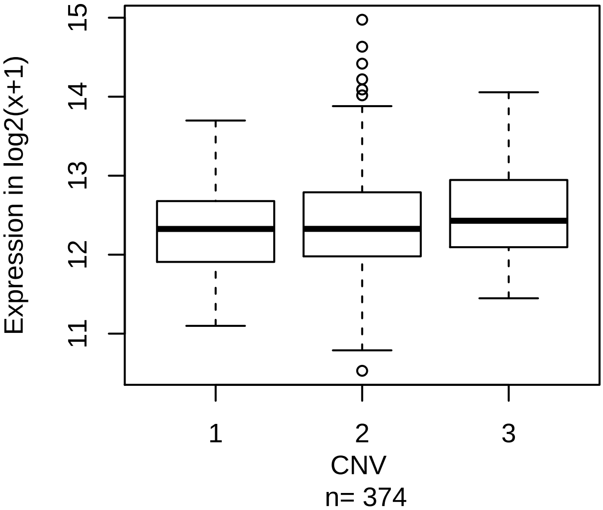

EEF1D Kaplan–Meier survival
pooled LGG+GBM, Etoposide exposure

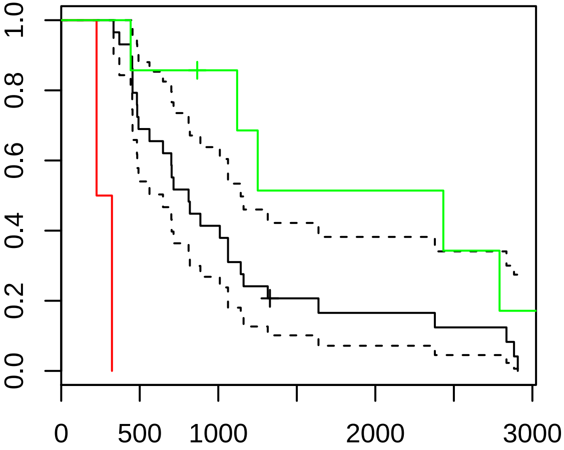

EEF1D expression and CNV for
pooled LGG+GBM, Etoposide exposure

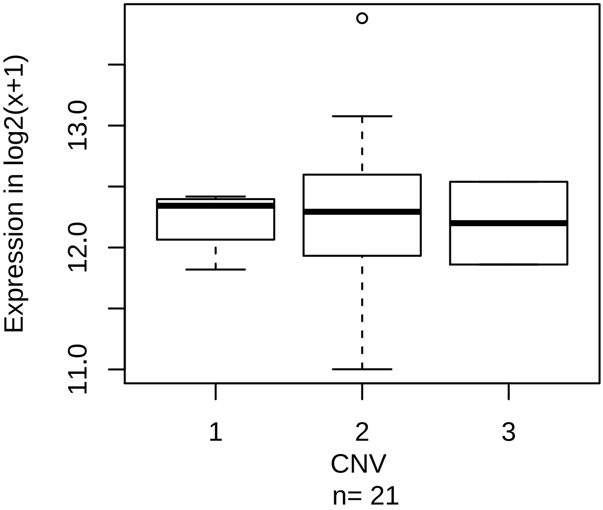

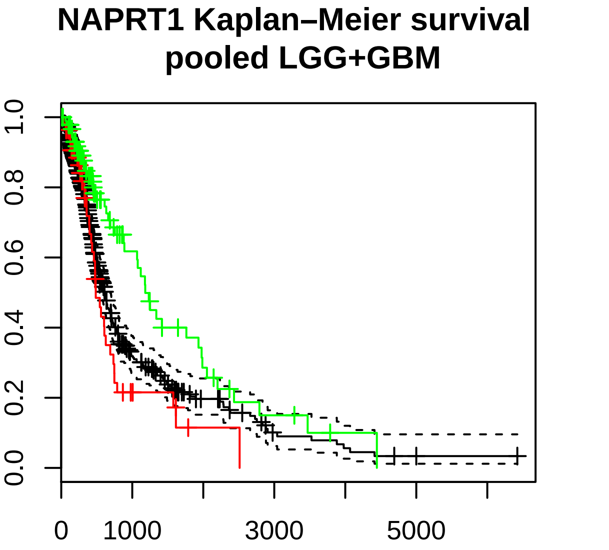
NAPRT1 Kaplan–Meier survival
pooled LGG+GBM
0.0
0.2
0.4
0.6
0.8
1.0
0
1000
3000
5000

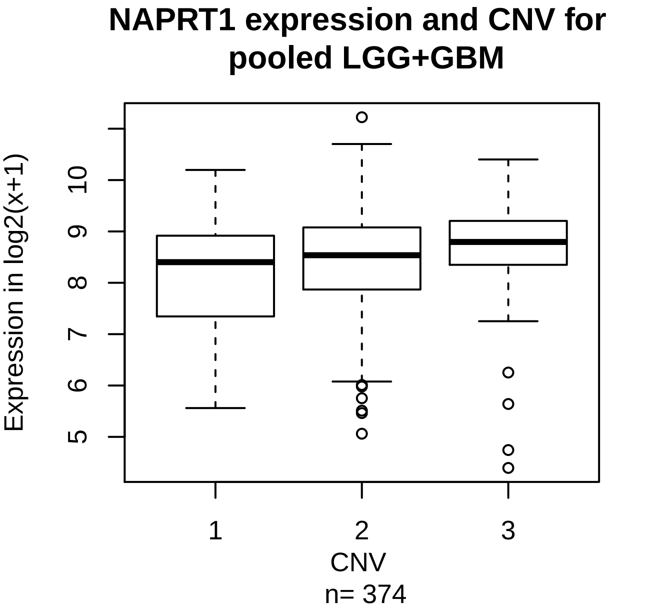
NAPRT1 expression and CNV for
pooled LGG+GBM
Expression in log2(x+1)
5
6
7
8
9
10
1
2
3
CNV
n= 374

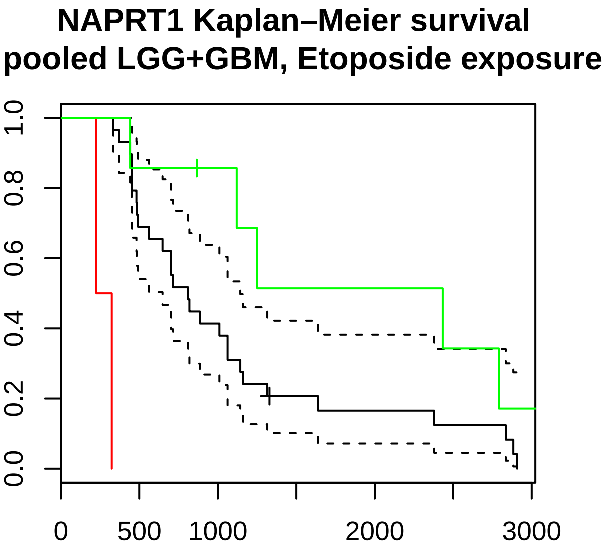
NAPRT1 Kaplan–Meier survival
pooled LGG+GBM, Etoposide exposure
0.0
0.2
0.4
0.6
0.8
1.0
0
500
1000
2000
3000

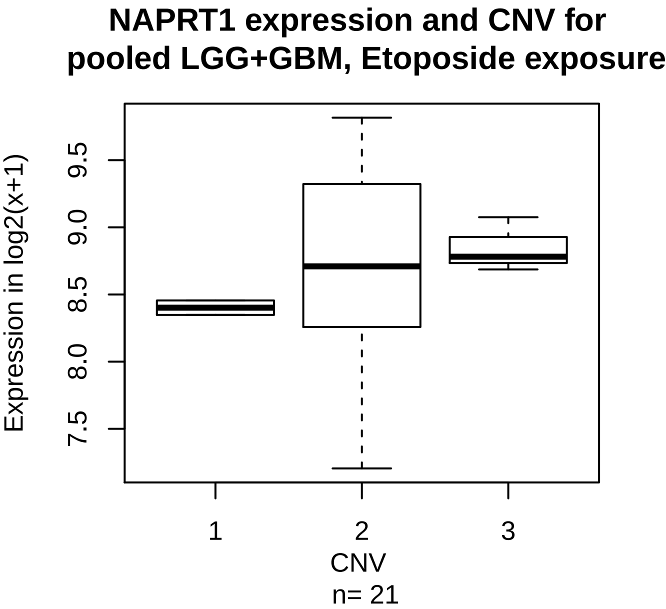
NAPRT1 expression and CNV for
pooled LGG+GBM, Etoposide exposure
Expression in log2(x+1)
7.5
8.0
8.5
9.0
9.5
1
2
3
CNV
n= 21

TIGD5 Kaplan–Meier survival pooled LGG+GBM

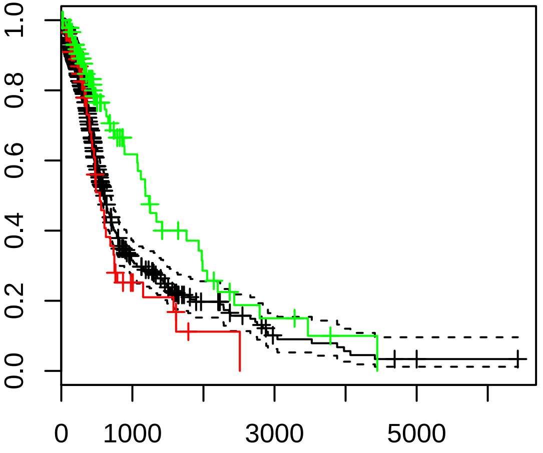

TIGD5 expression and CNV for pooled LGG+GBM

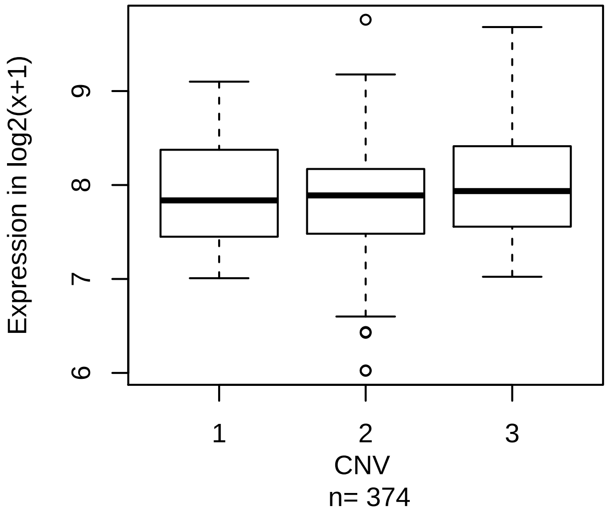

TIGD5 Kaplan–Meier survival pooled LGG+GBM, Etoposide exposure

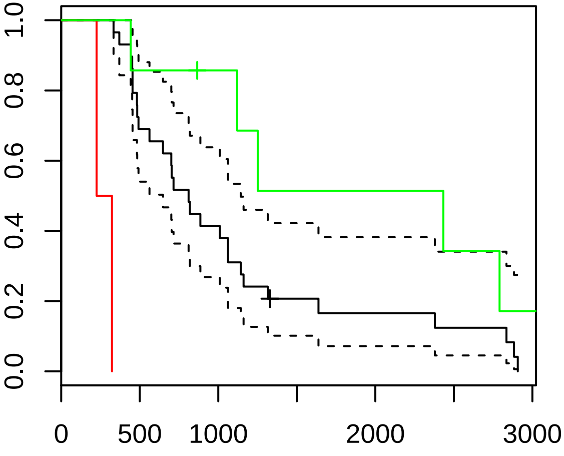

TIGD5 expression and CNV for pooled LGG+GBM, Etoposide exposure

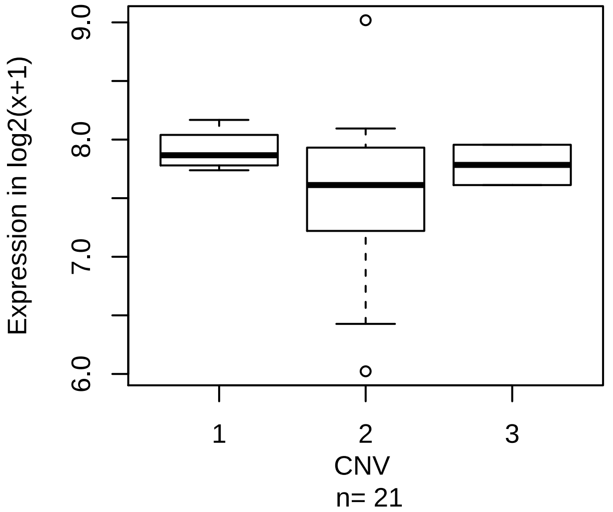

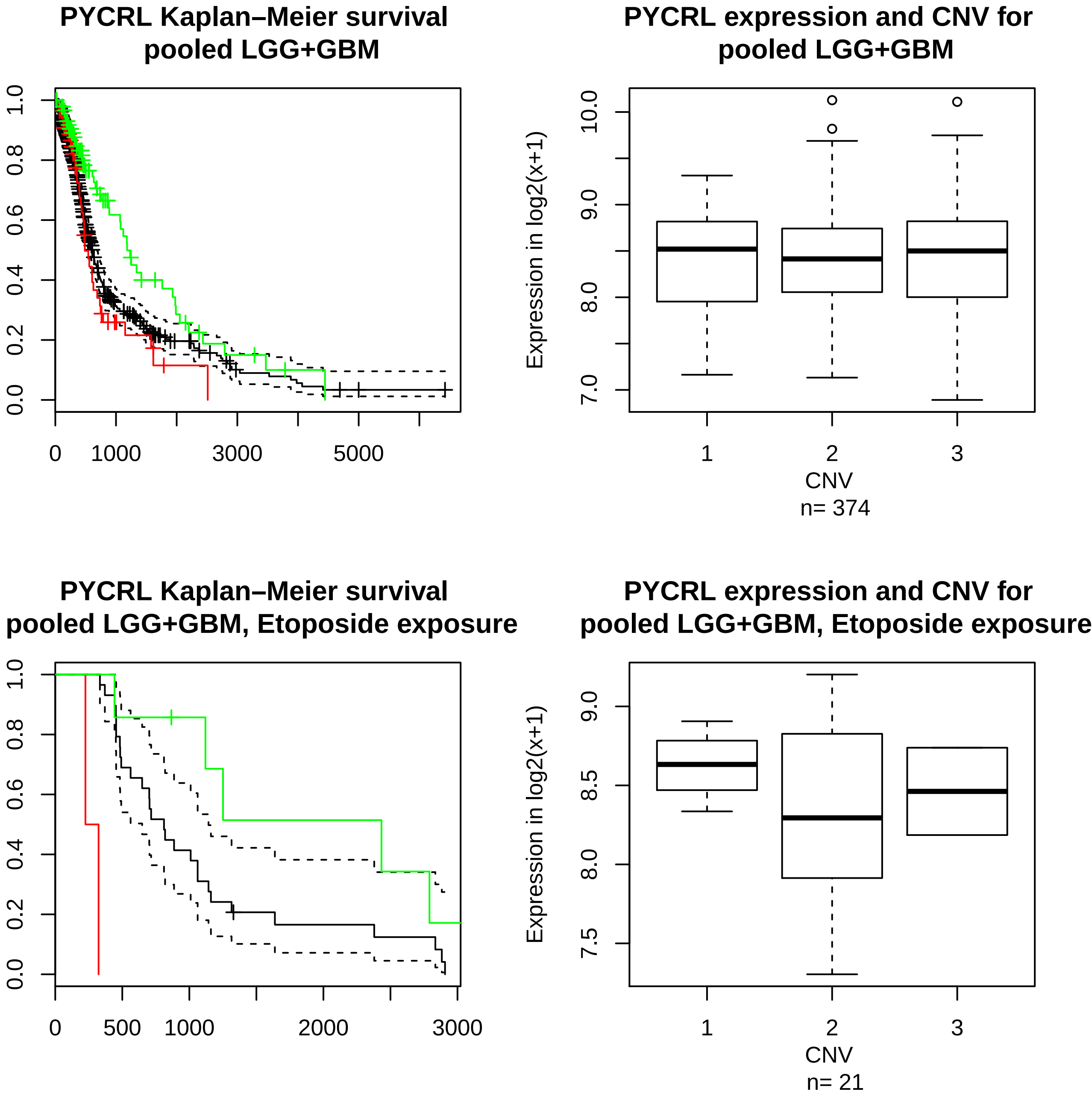
PYCRL Kaplan–Meier survival
pooled LGG+GBM
0.0
0.2
0.4
0.6
0.8
1.0
0
1000
3000
5000
PYCRL expression and CNV for
pooled LGG+GBM
Expression in log2(x+1)
7.0
8.0
9.0
10.0
1
2
3
CNV
n= 374
PYCRL Kaplan–Meier survival
pooled LGG+GBM, Etoposide exposure
0.0
0.2
0.4
0.6
0.8
1.0
0
500
1000
2000
3000
PYCRL expression and CNV for
pooled LGG+GBM, Etoposide exposure
Expression in log2(x+1)
7.5
8.0
8.5
9.0
1
2
3
CNV
n= 21

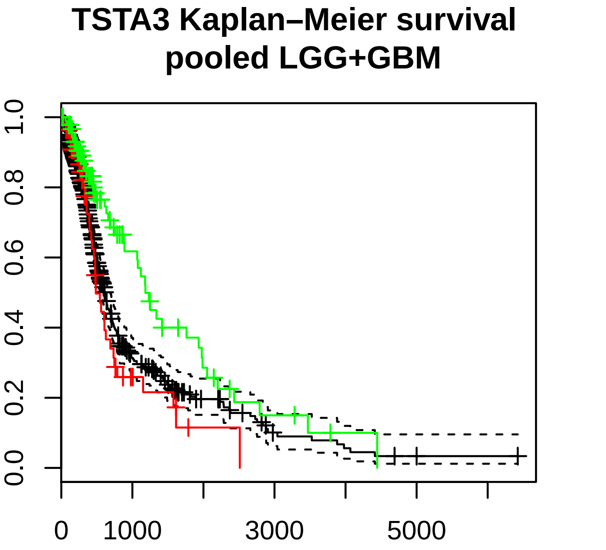
TSTA3 Kaplan–Meier survival
pooled LGG+GBM
0.0
0.2
0.4
0.6
0.8
1.0
0
1000
3000
5000

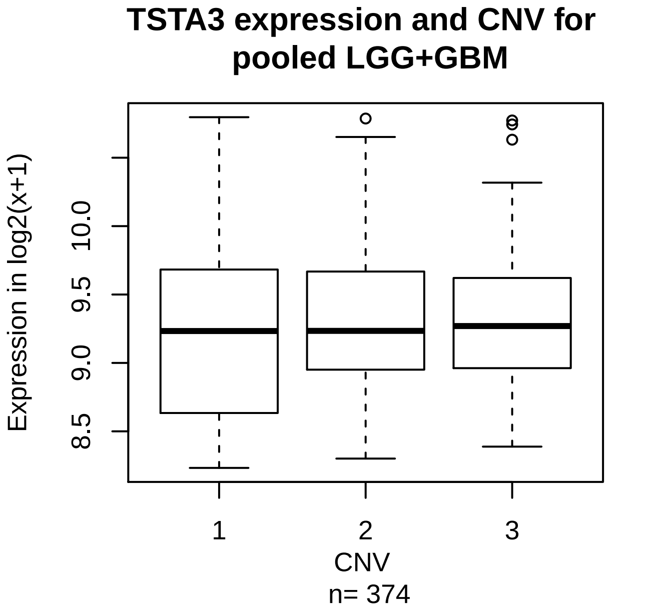
TSTA3 expression and CNV for
pooled LGG+GBM
Expression in log2(x+1)
8.5
9.0
9.5
10.0
1
2
3
CNV
n= 374

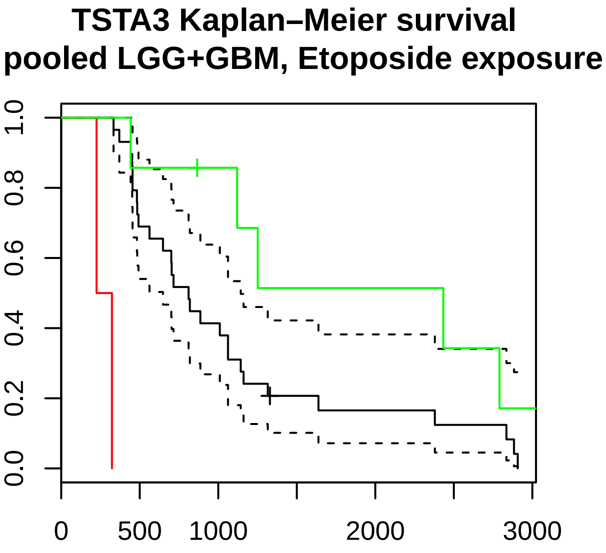
TSTA3 Kaplan–Meier survival
pooled LGG+GBM, Etoposide exposure
0.0
0.2
0.4
0.6
0.8
1.0
0
500
1000
2000
3000

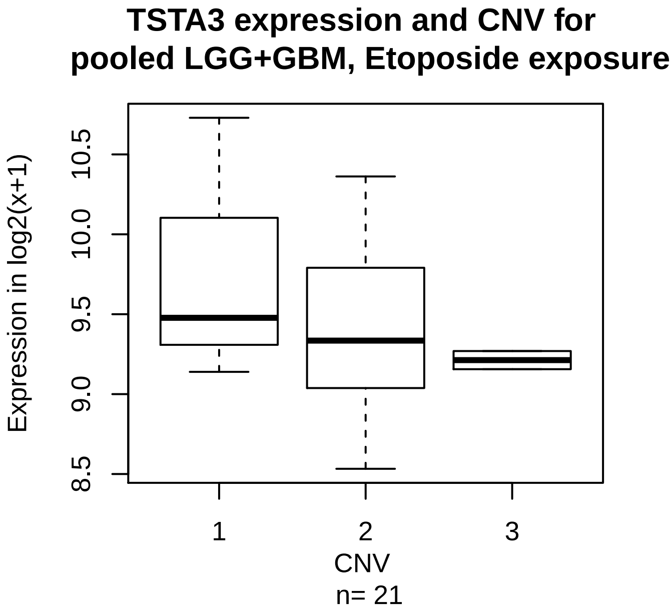
TSTA3 expression and CNV for
pooled LGG+GBM, Etoposide exposure
Expression in log2(x+1)
8.5
9.0
9.5
10.0
10.5
1
2
3
CNV
n= 21

ZNF623 Kaplan–Meier survival pooled LGG+GBM

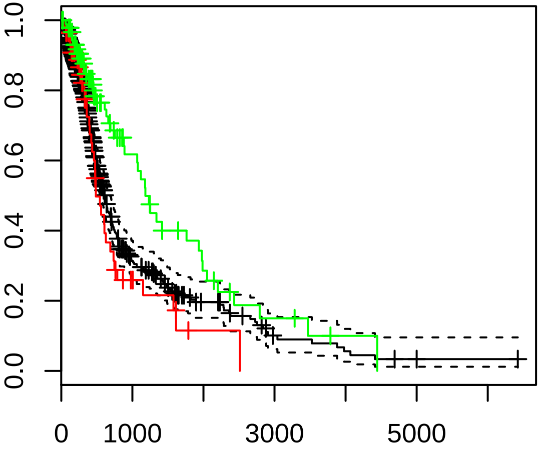

ZNF623 expression and CNV for pooled LGG+GBM

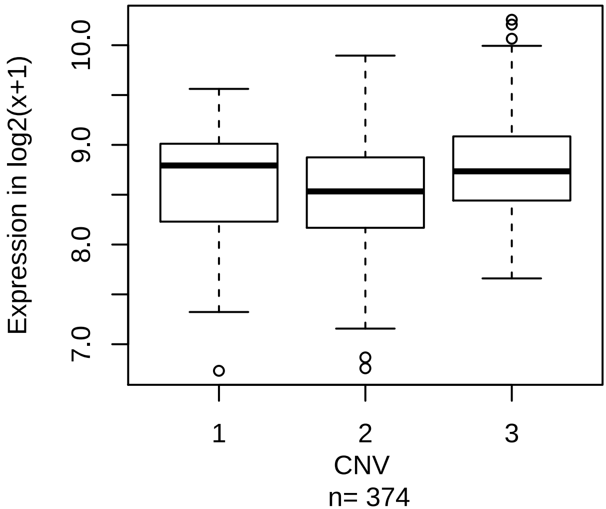

ZNF623 Kaplan–Meier survival pooled LGG+GBM, Etoposide exposure

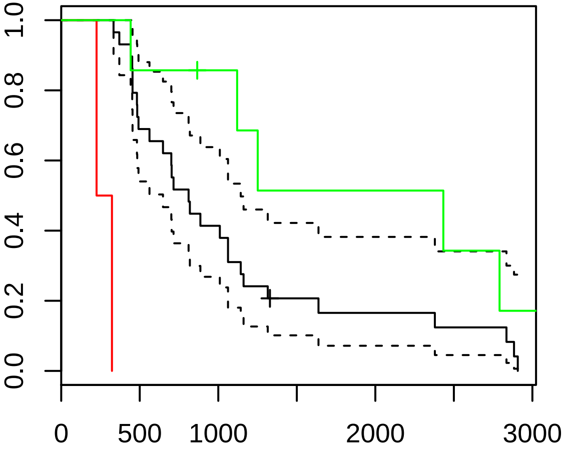

ZNF623 expression and CNV for pooled LGG+GBM, Etoposide exposure

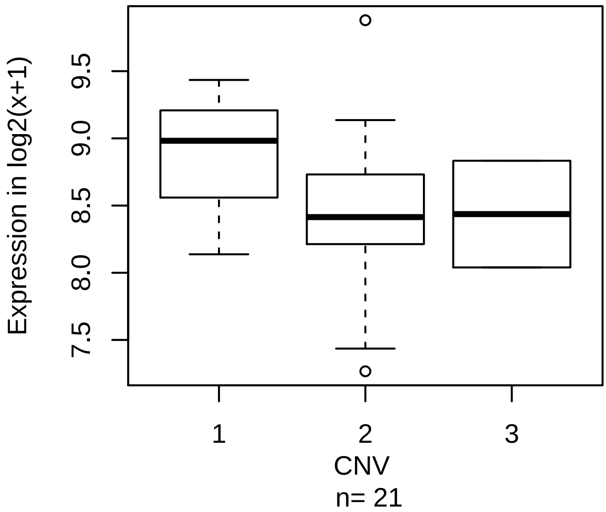

FAM83H Kaplan–Meier survival
pooled LGG+GBM

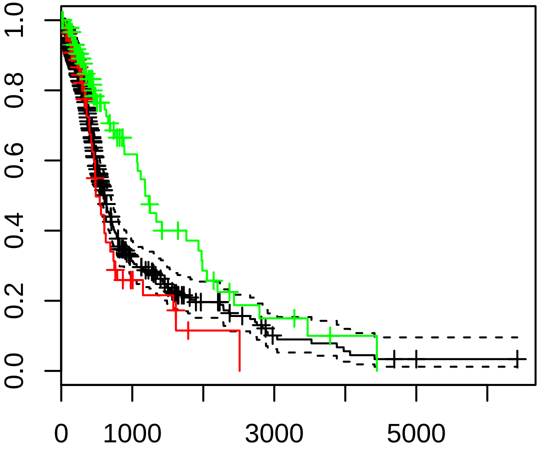

FAM83H expression and CNV for
pooled LGG+GBM

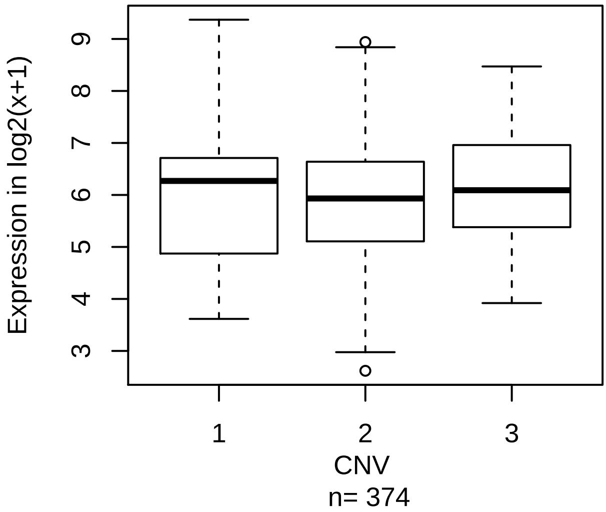

FAM83H Kaplan–Meier survival
pooled LGG+GBM, Etoposide exposure

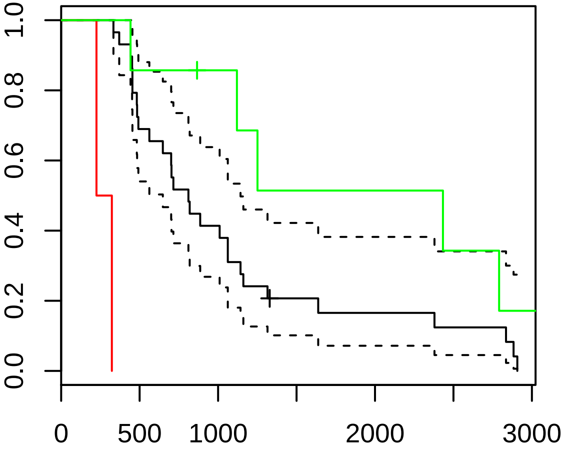

FAM83H expression and CNV for
pooled LGG+GBM, Etoposide exposure

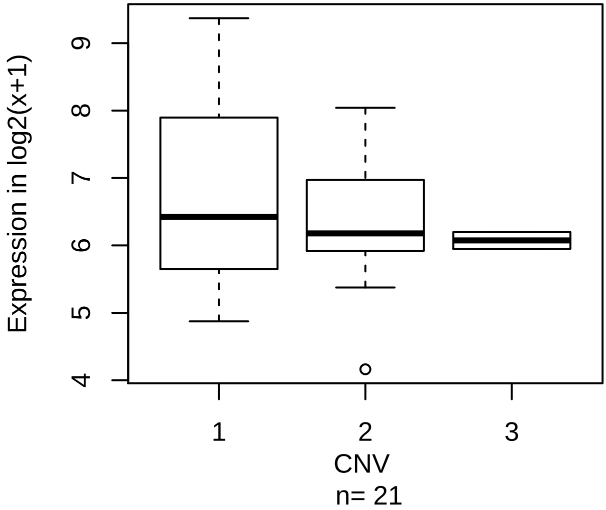

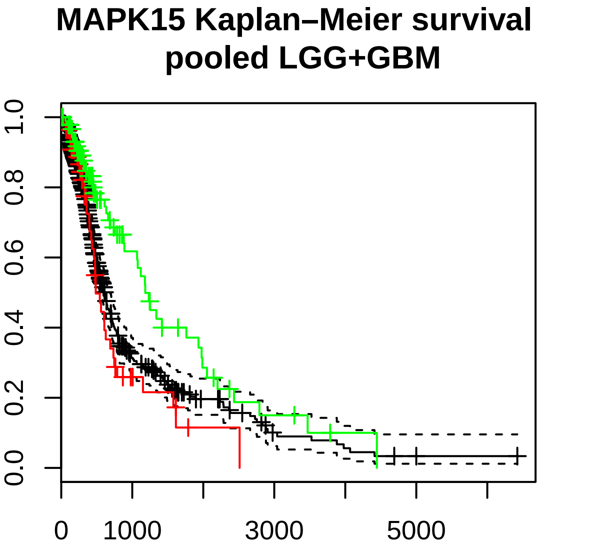
MAPK15 Kaplan–Meier survival
pooled LGG+GBM
1.0
0.8
0.6
0.4
0.2
0.0
0
1000
3000
5000

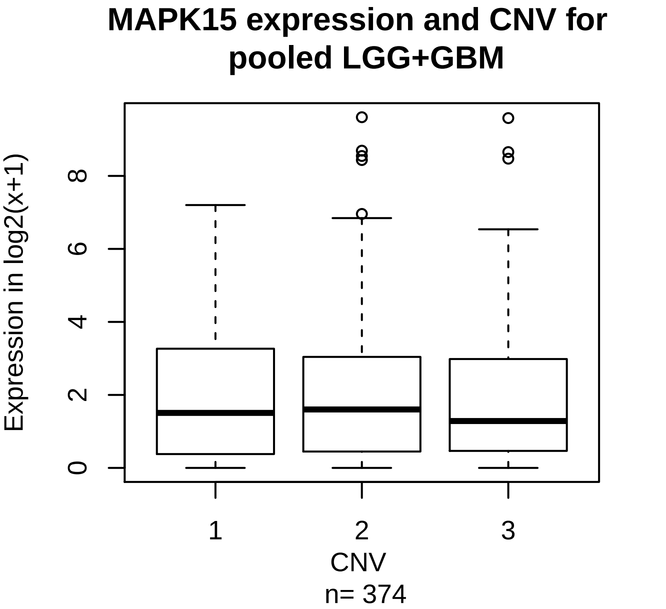
MAPK15 expression and CNV for
pooled LGG+GBM
Expression in log2(x+1)
8
6
4
2
0
1
2
3
CNV
n= 374

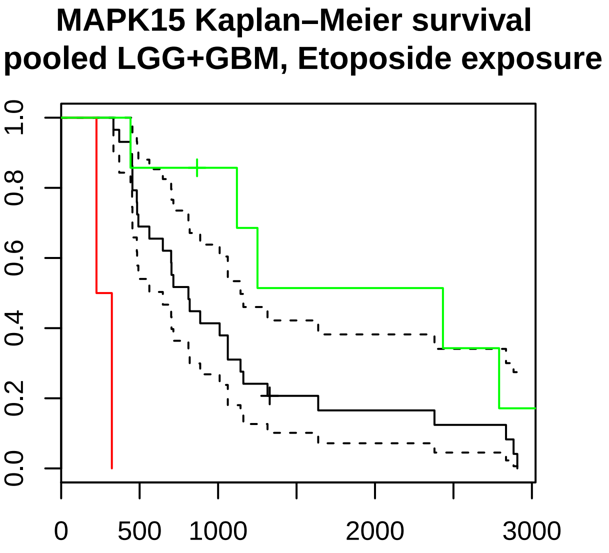
MAPK15 Kaplan–Meier survival
pooled LGG+GBM, Etoposide exposure
1.0
0.8
0.6
0.4
0.2
0.0
0
500
1000
2000
3000

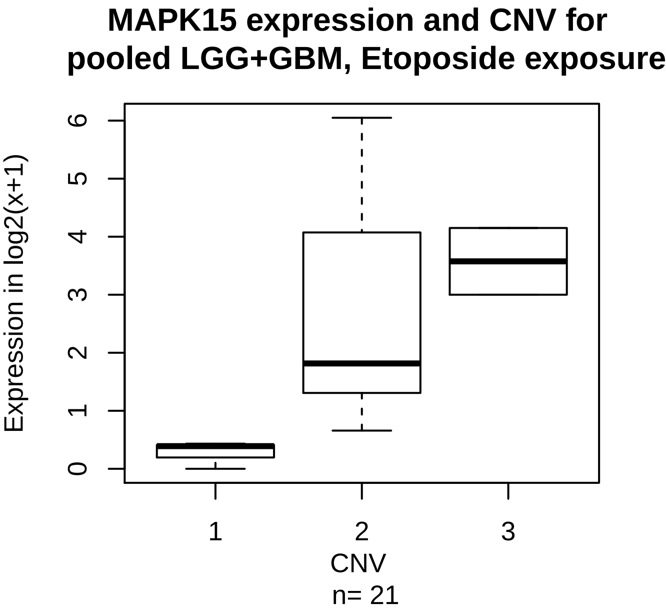
MAPK15 expression and CNV for
pooled LGG+GBM, Etoposide exposure
Expression in log2(x+1)
6
5
4
3
2
1
0
1
2
3
CNV
n= 21

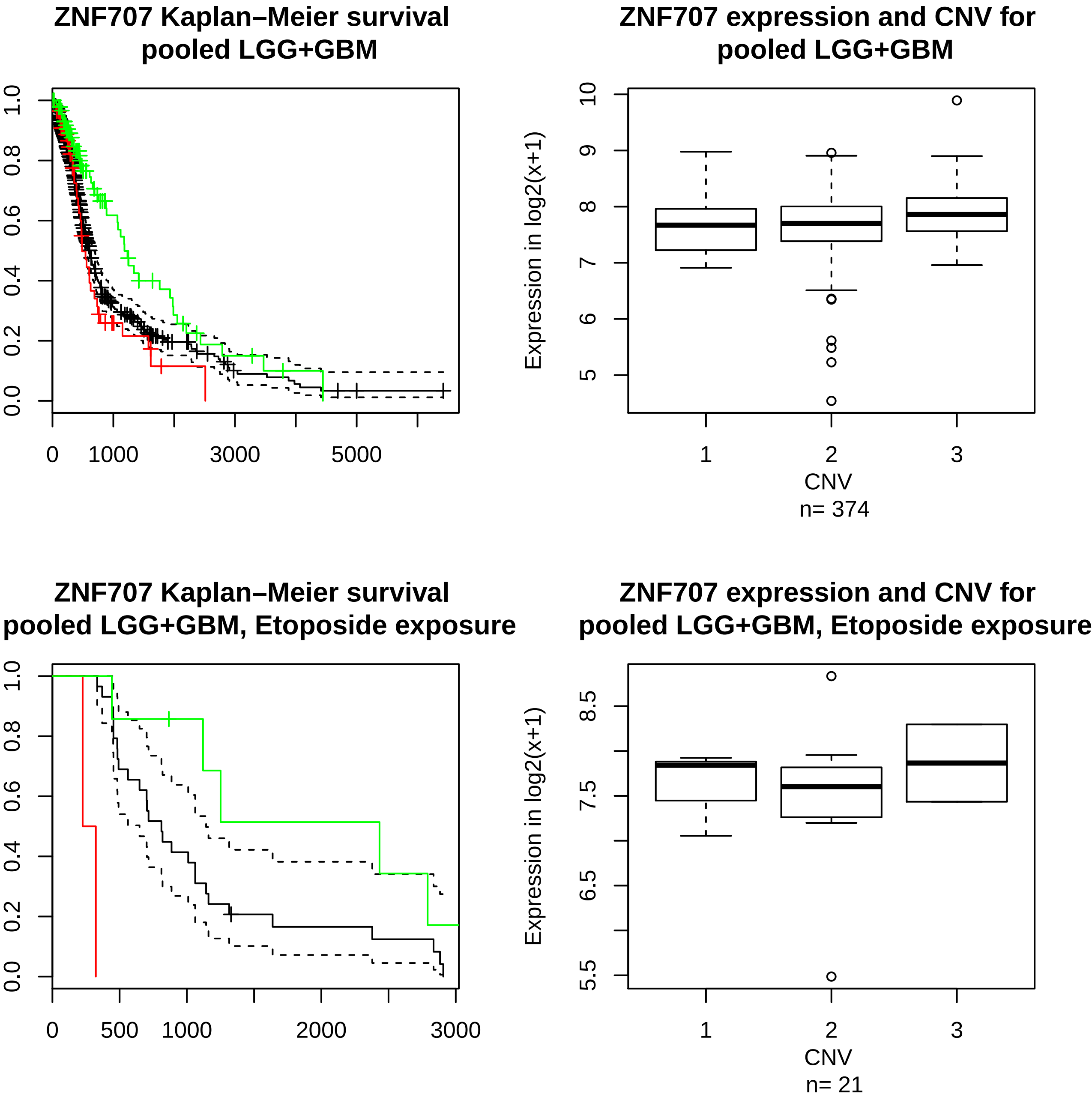
ZNF707 Kaplan–Meier survival
pooled LGG+GBM
0.0
0.2
0.4
0.6
0.8
1.0
0
1000
3000
5000
ZNF707 expression and CNV for
pooled LGG+GBM
Expression in log2(x+1)
5
6
7
8
9
10
1
2
3
CNV
n= 374
ZNF707 Kaplan–Meier survival
pooled LGG+GBM, Etoposide exposure
0.0
0.2
0.4
0.6
0.8
1.0
0
500
1000
2000
3000
ZNF707 expression and CNV for
pooled LGG+GBM, Etoposide exposure
Expression in log2(x+1)
5.5
6.5
7.5
8.5
1
2
3
CNV
n= 21

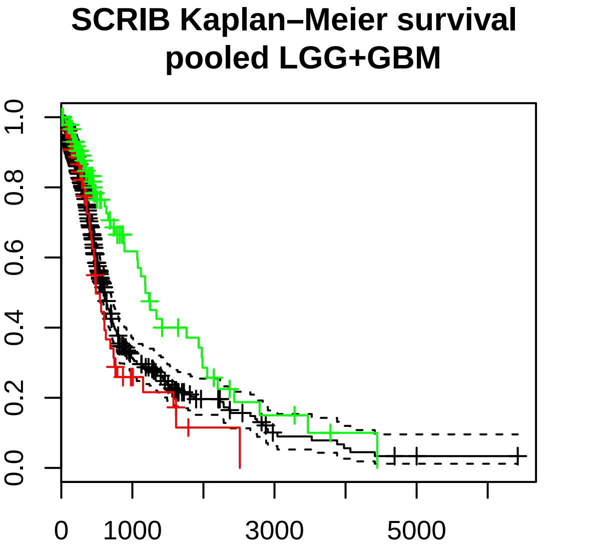
SCRIB Kaplan–Meier survival
pooled LGG+GBM
0.0
0.2
0.4
0.6
0.8
1.0
0
1000
3000
5000

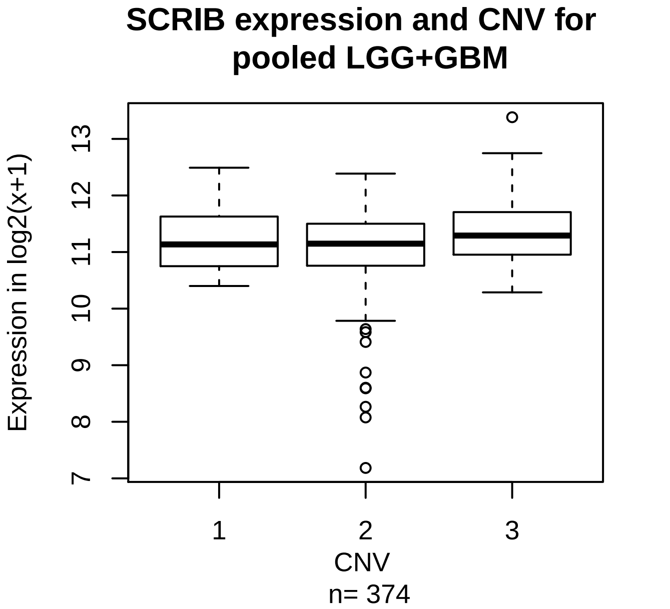
SCRIB expression and CNV for
pooled LGG+GBM
Expression in log2(x+1)
7
8
9
10
11
12
13
1
2
3
CNV
n= 374

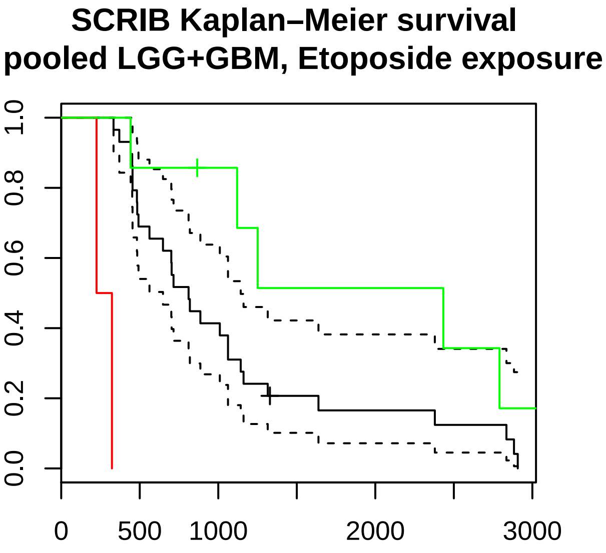
SCRIB Kaplan–Meier survival
pooled LGG+GBM, Etoposide exposure
0.0
0.2
0.4
0.6
0.8
1.0
0
500
1000
2000
3000

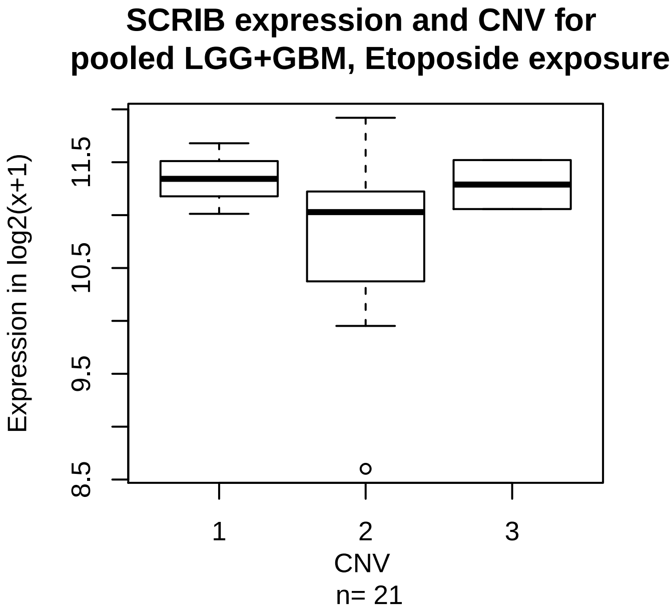
SCRIB expression and CNV for
pooled LGG+GBM, Etoposide exposure
Expression in log2(x+1)
8.5
9.5
10.5
11.5
1
2
3
CNV
n= 21

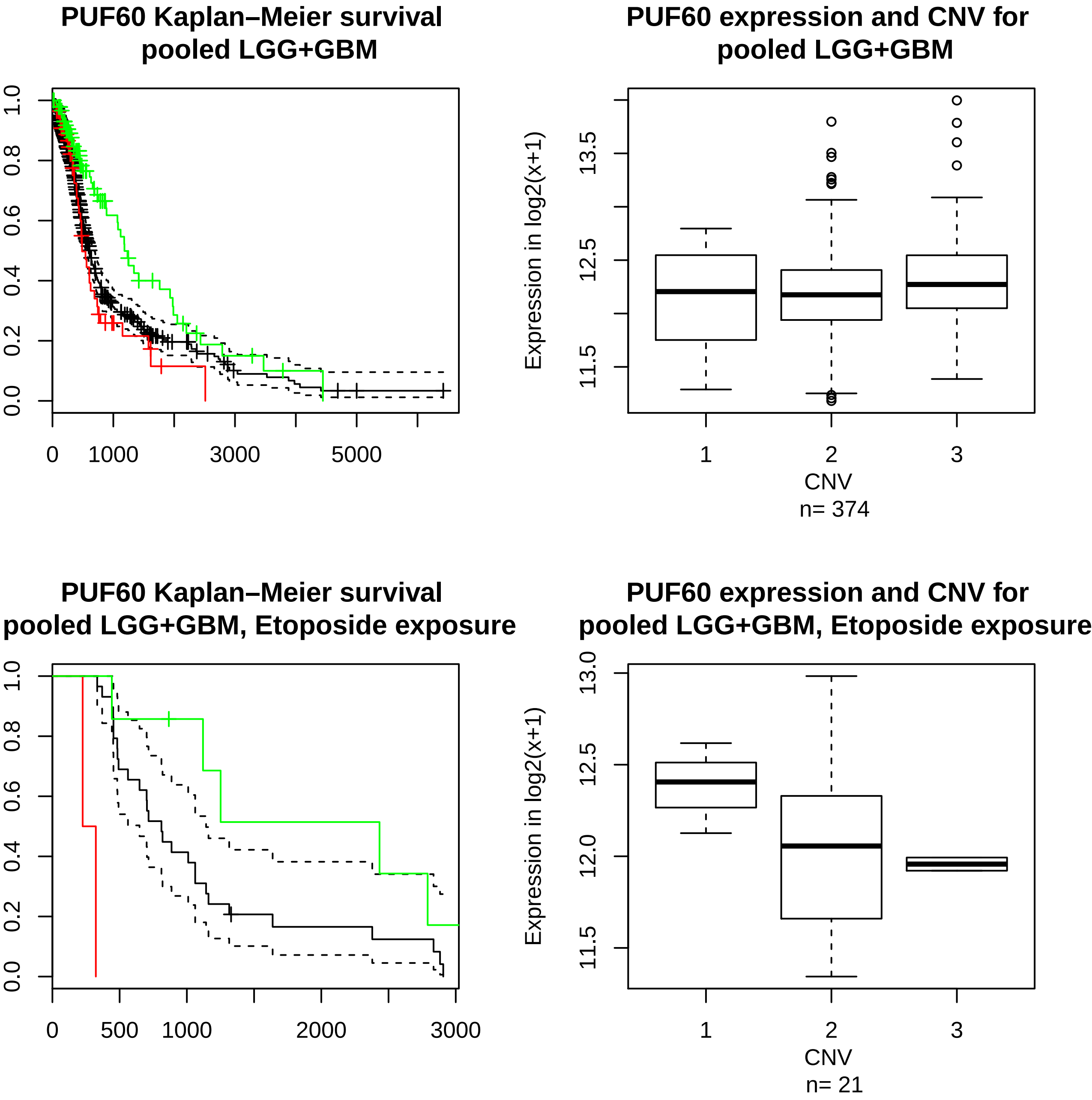
PUF60 Kaplan–Meier survival
pooled LGG+GBM
0.0
0.2
0.4
0.6
0.8
1.0
0
1000
3000
5000
PUF60 expression and CNV for
pooled LGG+GBM
Expression in log2(x+1)
11.5
12.5
13.5
1
2
3
CNV
n= 374
PUF60 Kaplan–Meier survival
pooled LGG+GBM, Etoposide exposure
0.0
0.2
0.4
0.6
0.8
1.0
0
500
1000
2000
3000
PUF60 expression and CNV for
pooled LGG+GBM, Etoposide exposure
Expression in log2(x+1)
11.5
12.0
12.5
13.0
1
2
3
CNV
n= 21

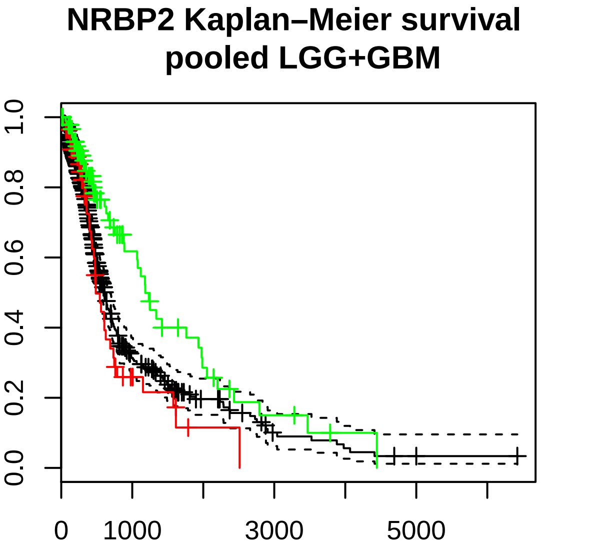
NRBP2 Kaplan–Meier survival
pooled LGG+GBM
0.0
0.2
0.4
0.6
0.8
1.0
0
1000
3000
5000

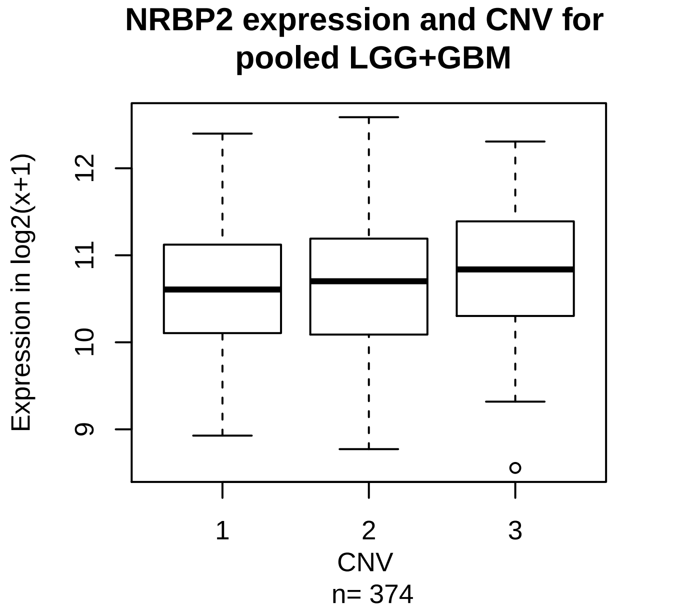
NRBP2 expression and CNV for
pooled LGG+GBM
Expression in log2(x+1)
9
10
11
12
1
2
3
CNV
n= 374

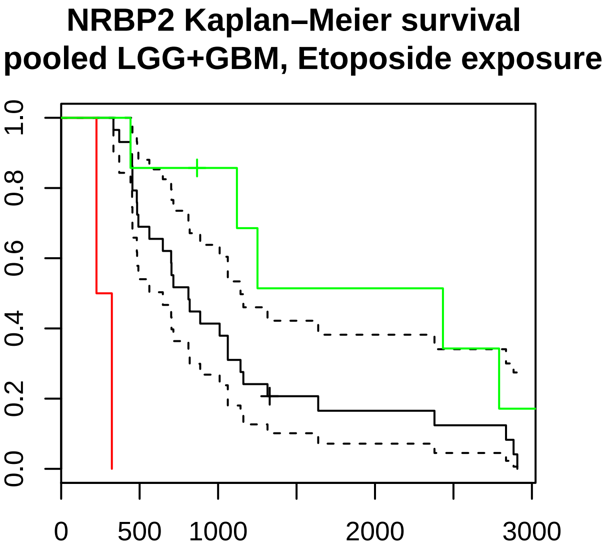
NRBP2 Kaplan–Meier survival
pooled LGG+GBM, Etoposide exposure
0.0
0.2
0.4
0.6
0.8
1.0
0
500
1000
2000
3000

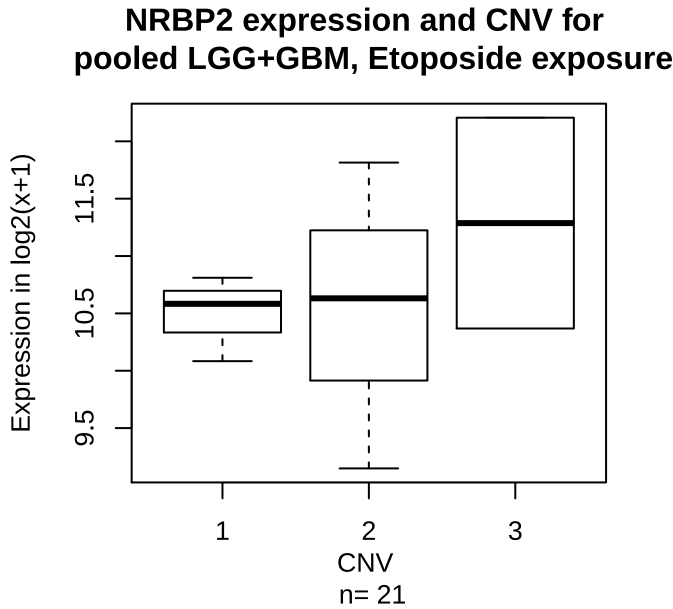
NRBP2 expression and CNV for
pooled LGG+GBM, Etoposide exposure
Expression in log2(x+1)
9.5
10.5
11.5
1
2
3
CNV
n= 21

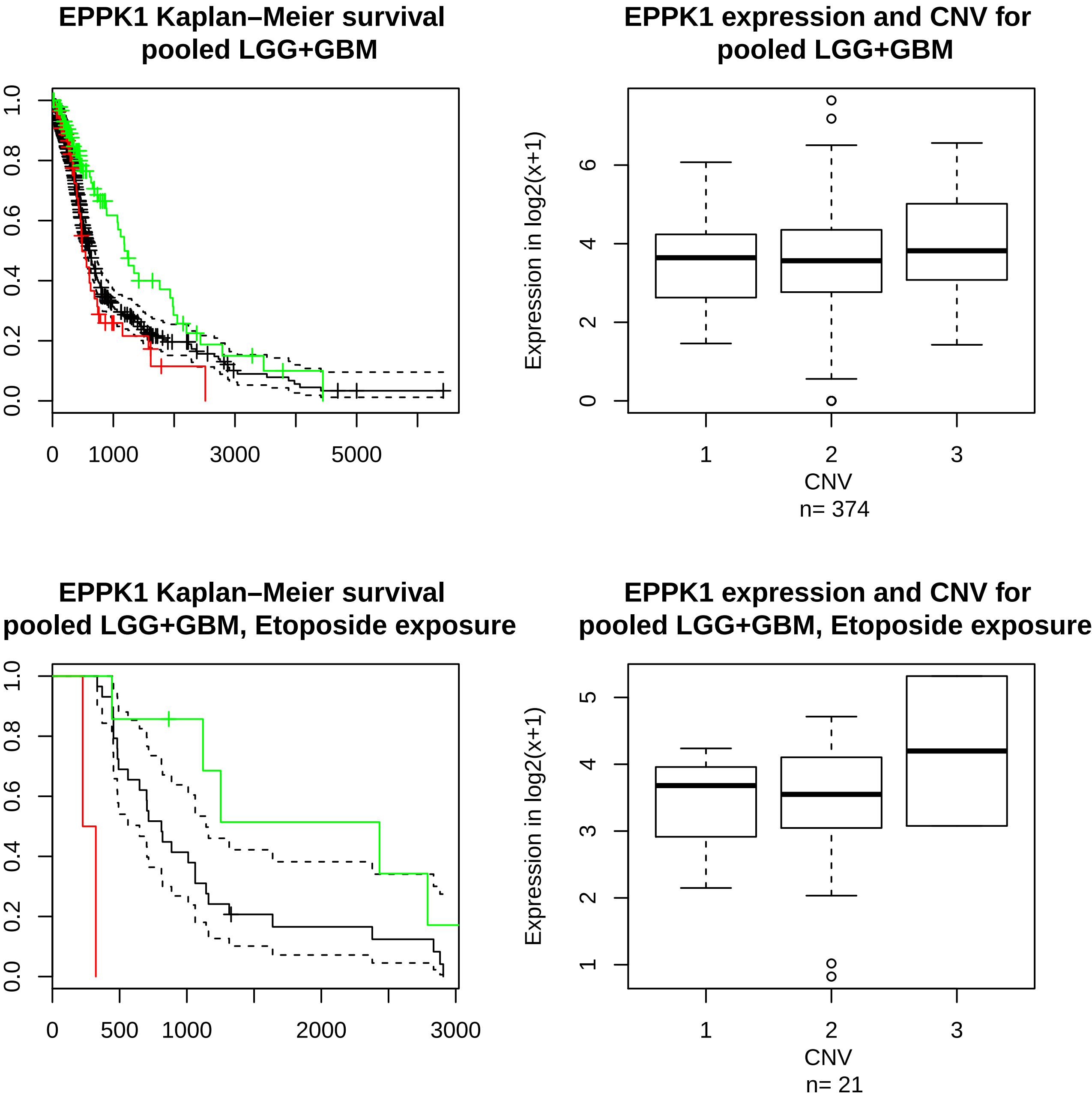
EPPK1 Kaplan–Meier survival
pooled LGG+GBM
0.0
0.2
0.4
0.6
0.8
1.0
0
1000
3000
5000
EPPK1 expression and CNV for
pooled LGG+GBM
Expression in log2(x+1)
0
2
4
6
1
2
3
CNV
n= 374
EPPK1 Kaplan–Meier survival
pooled LGG+GBM, Etoposide exposure
0.0
0.2
0.4
0.6
0.8
1.0
0
500
1000
2000
3000
EPPK1 expression and CNV for
pooled LGG+GBM, Etoposide exposure
Expression in log2(x+1)
1
2
3
4
5
1
2
3
CNV
n= 21

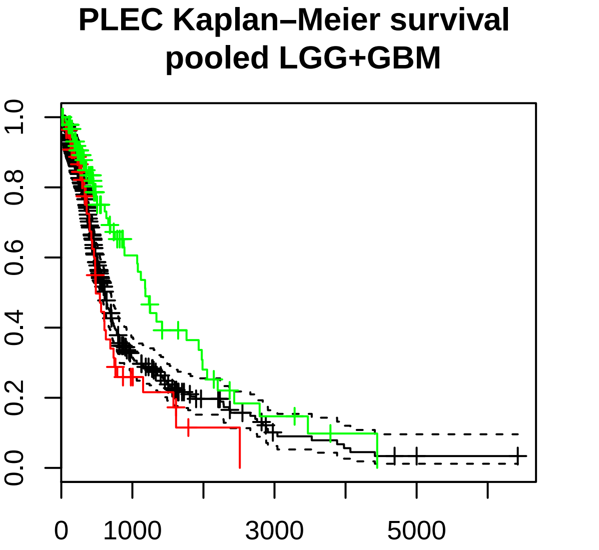
PLEC Kaplan–Meier survival
pooled LGG+GBM
0.0
0.2
0.4
0.6
0.8
1.0
0
1000
3000
5000

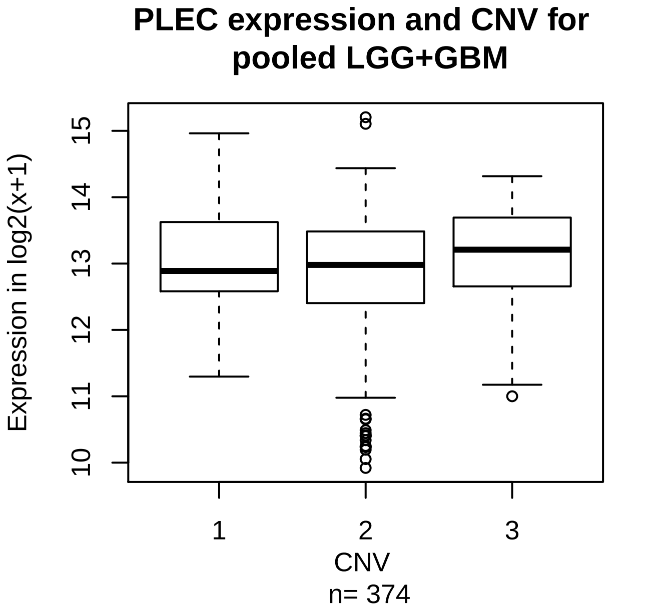
PLEC expression and CNV for
pooled LGG+GBM
Expression in log2(x+1)
10
11
12
13
14
15
1
2
3
CNV
n= 374

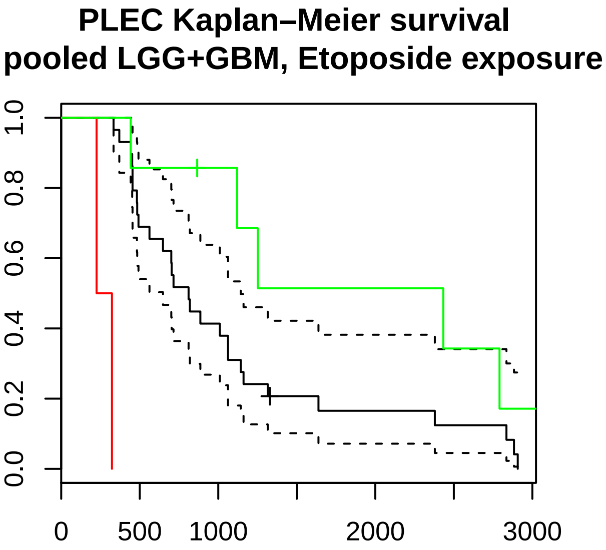
PLEC Kaplan–Meier survival
pooled LGG+GBM, Etoposide exposure
0.0
0.2
0.4
0.6
0.8
1.0
0
500
1000
2000
3000

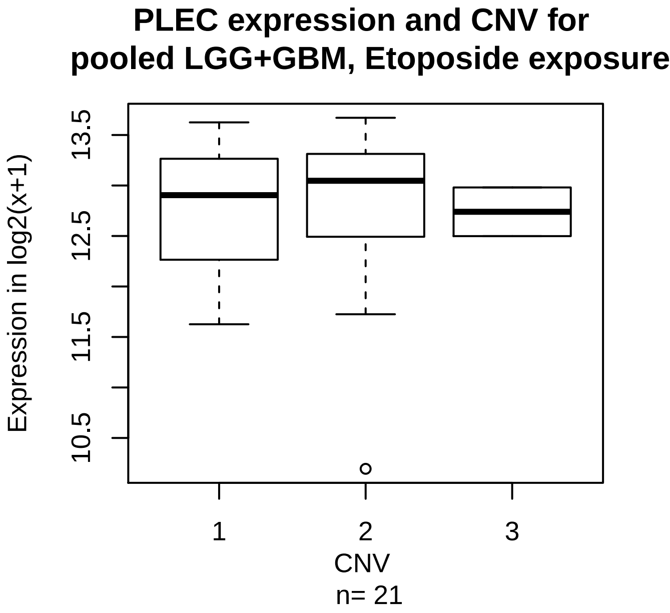
PLEC expression and CNV for
pooled LGG+GBM, Etoposide exposure
Expression in log2(x+1)
10.5
11.5
12.5
13.5
1
2
3
CNV
n= 21

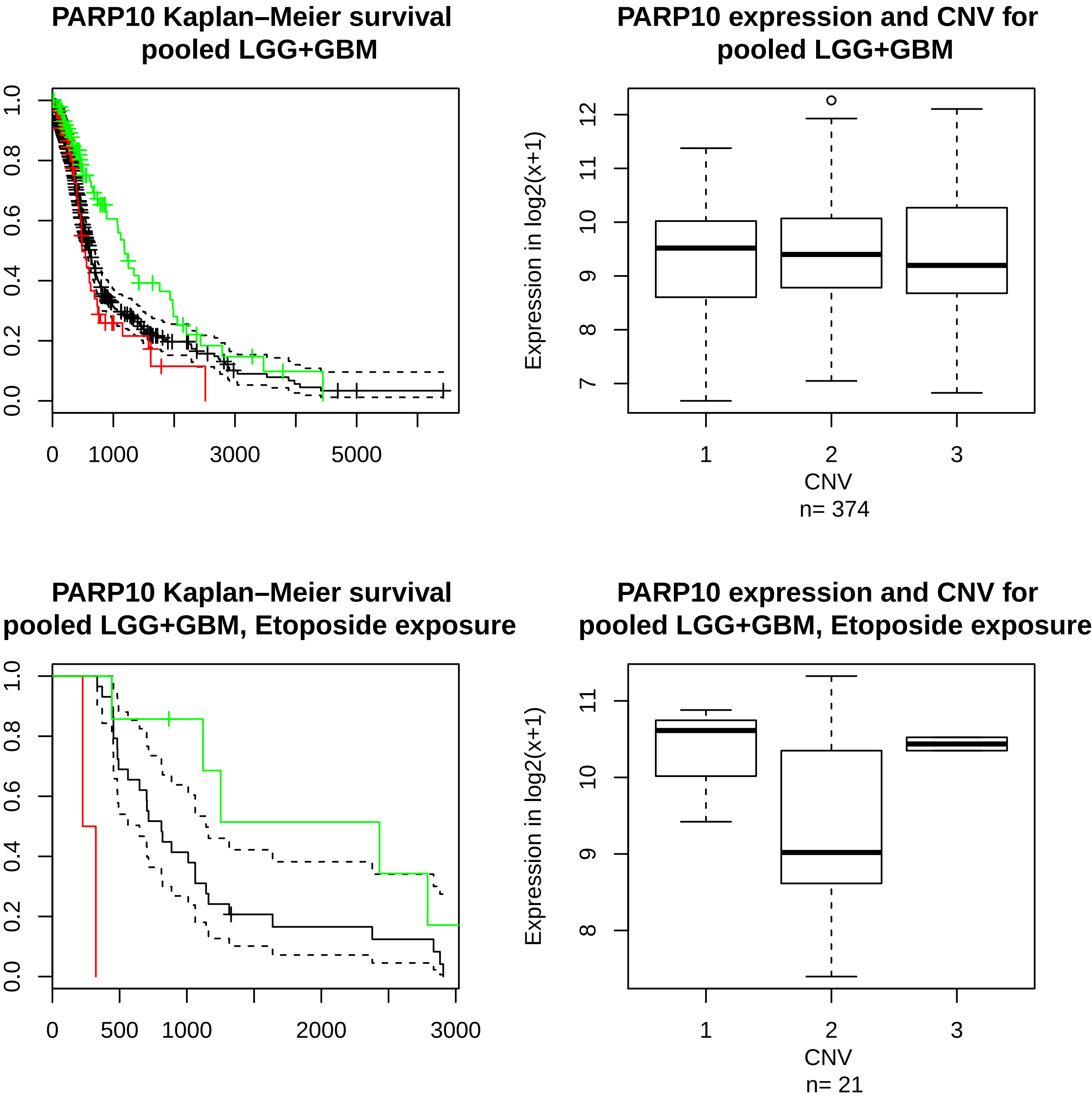
PARP10 Kaplan–Meier survival
pooled LGG+GBM
0.0
0.2
0.4
0.6
0.8
1.0
0
1000
3000
5000
PARP10 expression and CNV for
pooled LGG+GBM
Expression in log2(x+1)
7
8
9
10
11
12
1
2
3
CNV
n= 374
PARP10 Kaplan–Meier survival
pooled LGG+GBM, Etoposide exposure
0.0
0.2
0.4
0.6
0.8
1.0
0
500
1000
2000
3000
PARP10 expression and CNV for
pooled LGG+GBM, Etoposide exposure
Expression in log2(x+1)
8
9
10
11
1
2
3
CNV
n= 21

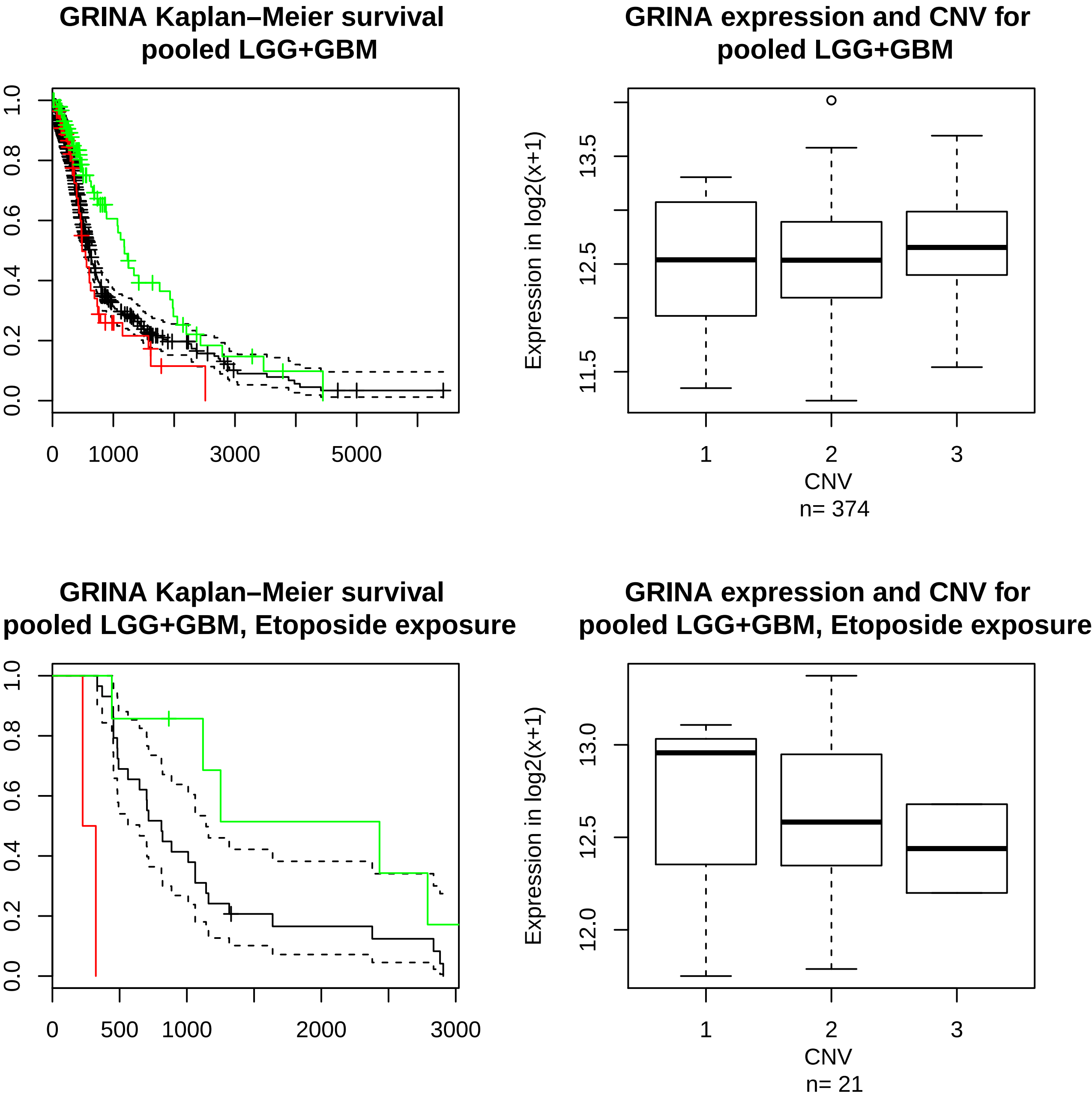
GRINA Kaplan–Meier survival pooled LGG+GBM
1.0
0.8
0.6
0.4
0.2
0.0
0
1000
3000
5000
GRINA expression and CNV for pooled LGG+GBM
Expression in log2(x+1)
13.5
12.5
11.5
1
2
3
CNV
n= 374
GRINA Kaplan–Meier survival pooled LGG+GBM, Etoposide exposure
1.0
0.8
0.6
0.4
0.2
0.0
0
500
1000
2000
3000
GRINA expression and CNV for pooled LGG+GBM, Etoposide exposure
Expression in log2(x+1)
13.0
12.5
12.0
1
2
3
CNV
n= 21

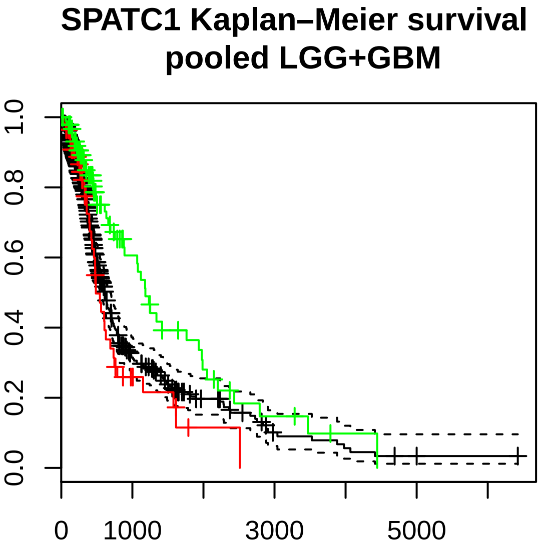
SPATC1 Kaplan–Meier survival
pooled LGG+GBM
0.0
0.2
0.4
0.6
0.8
1.0
0
1000
3000
5000

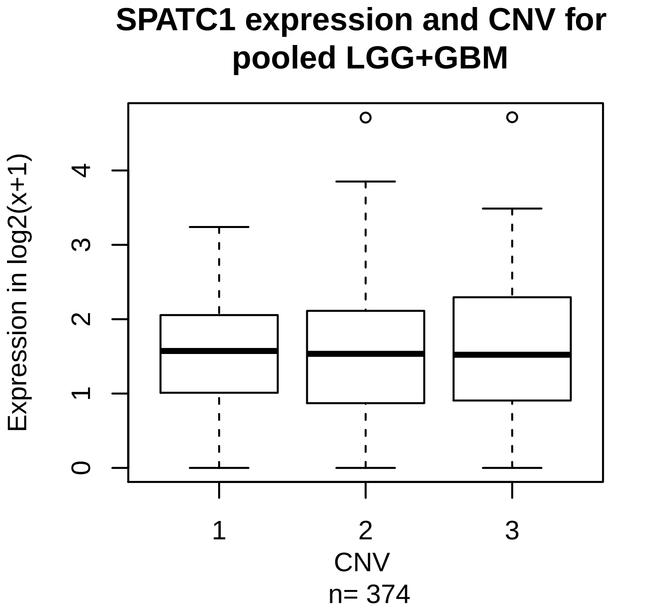
SPATC1 expression and CNV for
pooled LGG+GBM
Expression in log2(x+1)
0
1
2
3
4
1
2
3
CNV
n= 374

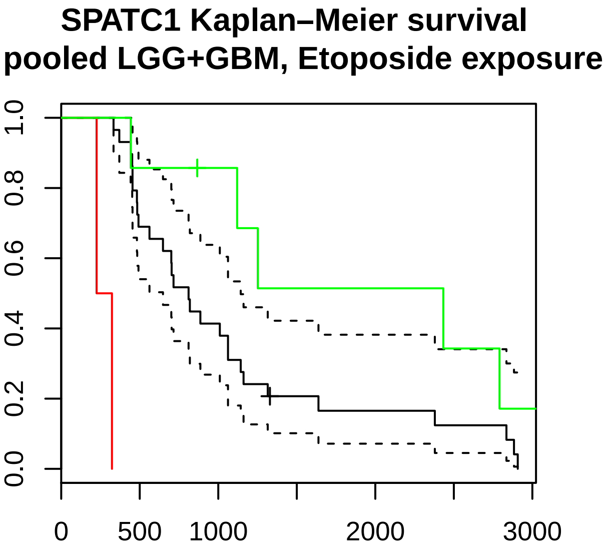
SPATC1 Kaplan–Meier survival
pooled LGG+GBM, Etoposide exposure
0.0
0.2
0.4
0.6
0.8
1.0
0
500
1000
2000
3000

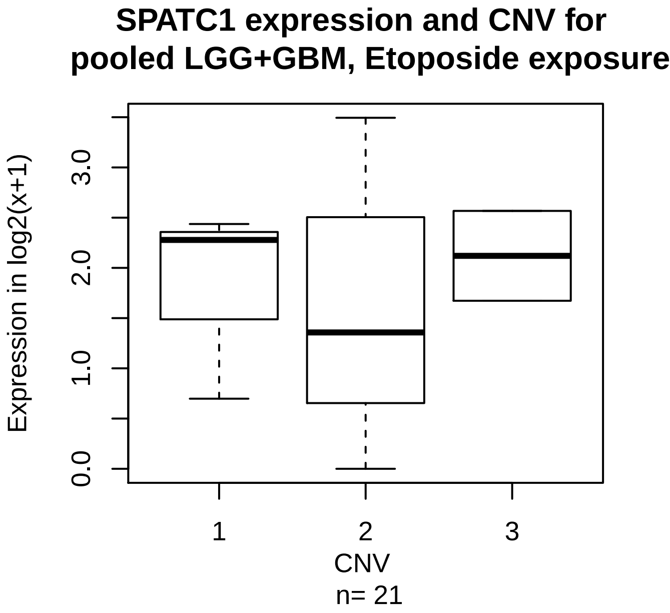
SPATC1 expression and CNV for
pooled LGG+GBM, Etoposide exposure
Expression in log2(x+1)
0.0
1.0
2.0
3.0
1
2
3
CNV
n= 21

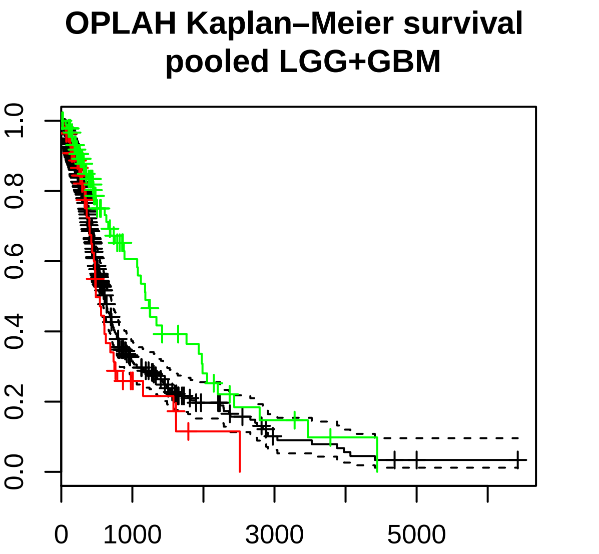
OPLAH Kaplan–Meier survival
pooled LGG+GBM
0.0
0.2
0.4
0.6
0.8
1.0
0
1000
3000
5000

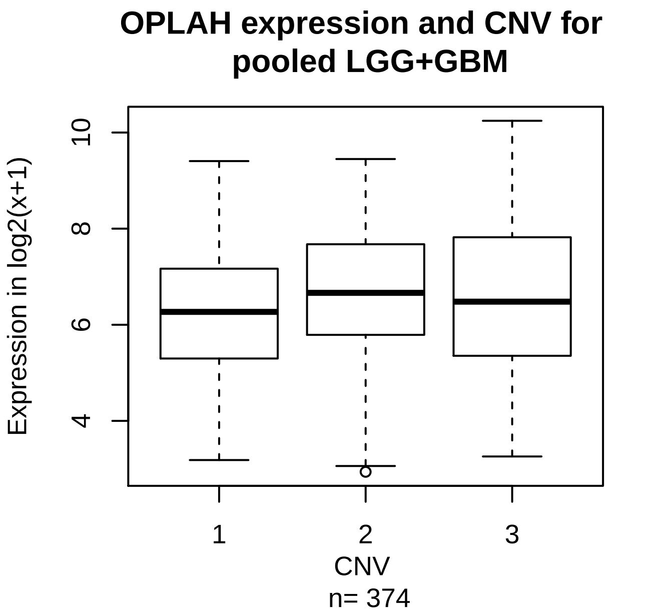
OPLAH expression and CNV for
pooled LGG+GBM
Expression in log2(x+1)
4
6
8
10
1
2
3
CNV
n= 374

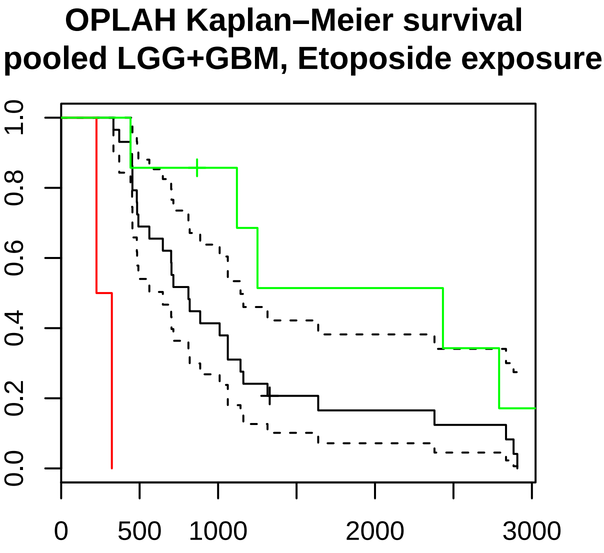
OPLAH Kaplan–Meier survival
pooled LGG+GBM, Etoposide exposure
0.0
0.2
0.4
0.6
0.8
1.0
0
500
1000
2000
3000

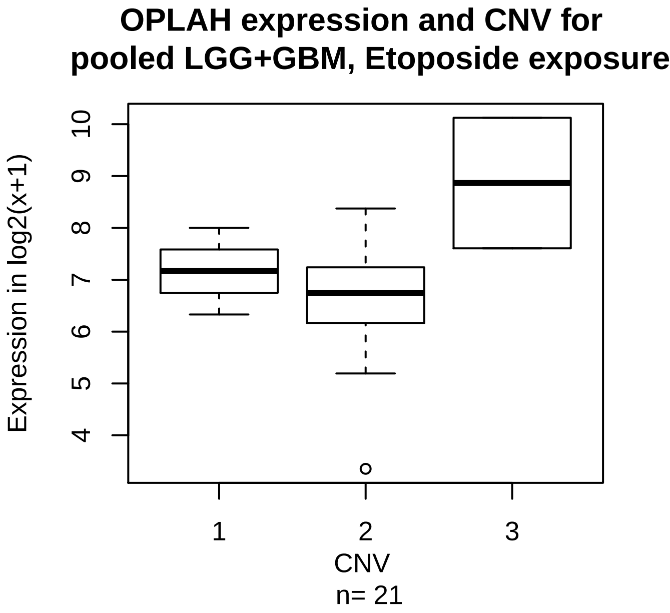
OPLAH expression and CNV for
pooled LGG+GBM, Etoposide exposure
Expression in log2(x+1)
4
5
6
7
8
9
10
1
2
3
CNV
n= 21

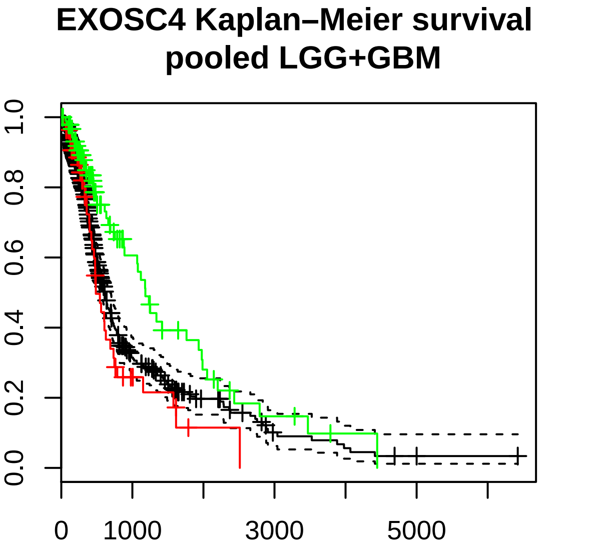
EXOSC4 Kaplan–Meier survival
pooled LGG+GBM
0.0
0.2
0.4
0.6
0.8
1.0
0
1000
3000
5000

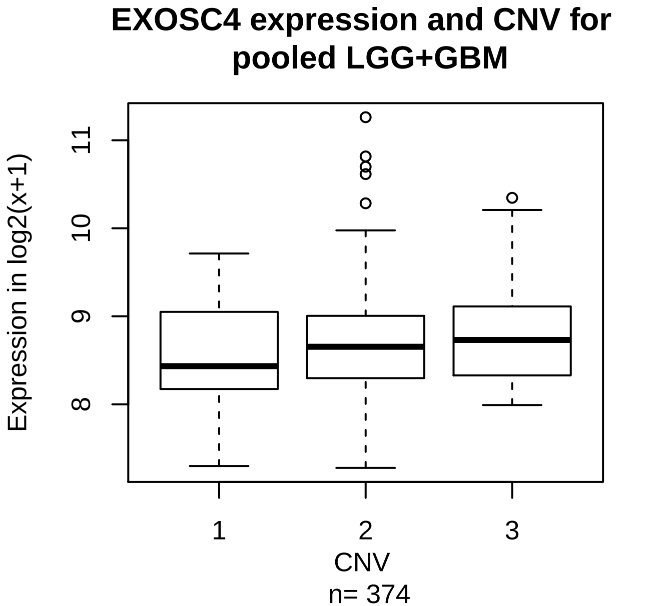
EXOSC4 expression and CNV for
pooled LGG+GBM
Expression in log2(x+1)
8
9
10
11
1
2
3
CNV
n= 374

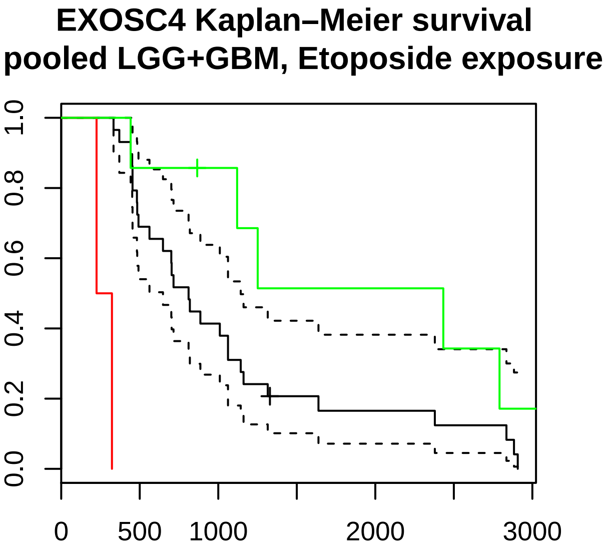
EXOSC4 Kaplan–Meier survival
pooled LGG+GBM, Etoposide exposure
0.0
0.2
0.4
0.6
0.8
1.0
0
500
1000
2000
3000

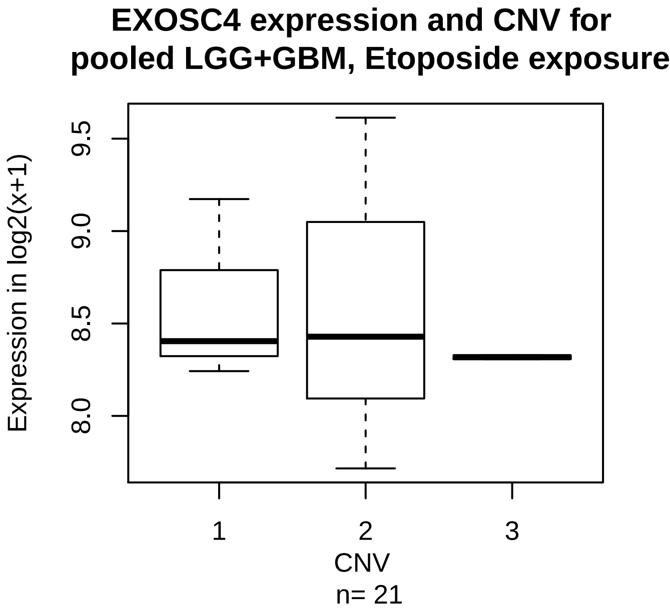
EXOSC4 expression and CNV for
pooled LGG+GBM, Etoposide exposure
Expression in log2(x+1)
8.0
8.5
9.0
9.5
1
2
3
CNV
n= 21

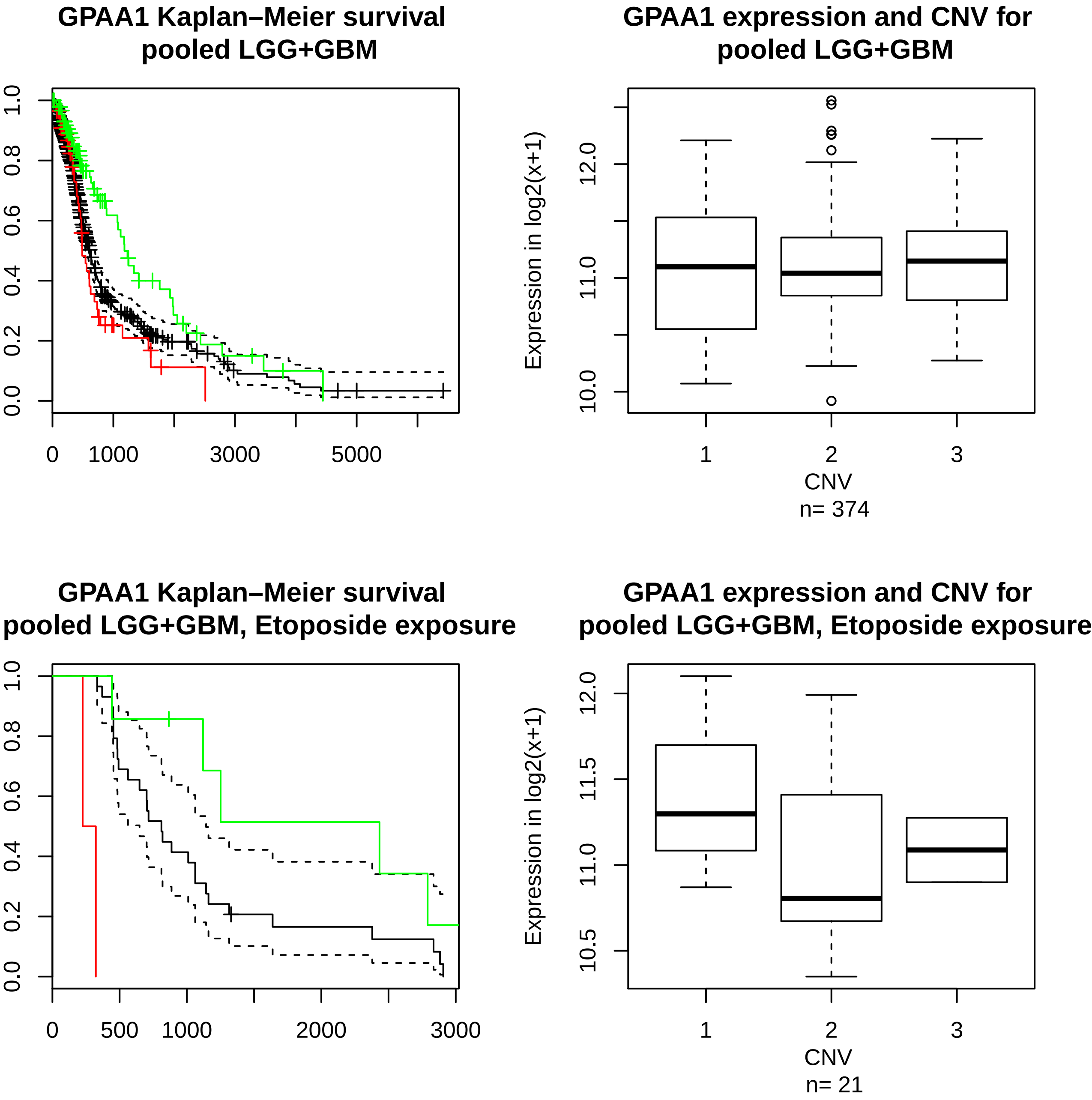
GPAA1 Kaplan–Meier survival pooled LGG+GBM
0.0
0.2
0.4
0.6
0.8
1.0
0
1000
3000
5000
GPAA1 expression and CNV for pooled LGG+GBM
Expression in log2(x+1)
10.0
11.0
12.0
1
2
3
CNV
n= 374
GPAA1 Kaplan–Meier survival pooled LGG+GBM, Etoposide exposure
0.0
0.2
0.4
0.6
0.8
1.0
0
500
1000
2000
3000
GPAA1 expression and CNV for pooled LGG+GBM, Etoposide exposure
Expression in log2(x+1)
10.5
11.0
11.5
12.0
1
2
3
CNV
n= 21

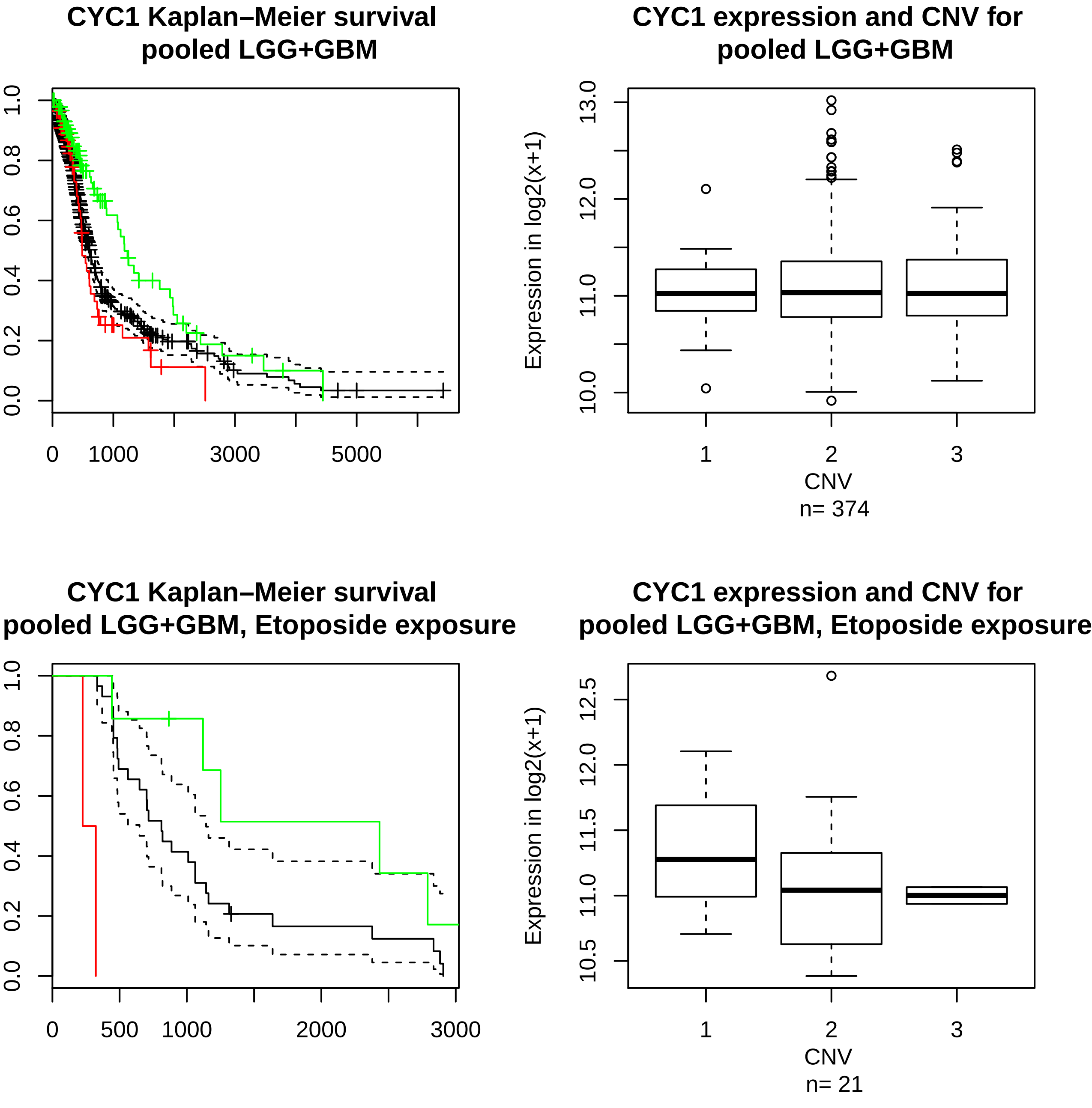

CYC1 Kaplan–Meier survival
pooled LGG+GBM
0.0
0.2
0.4
0.6
0.8
1.0
0
1000
3000
5000
CYC1 expression and CNV for
pooled LGG+GBM
Expression in log2(x+1)
10.0
11.0
12.0
13.0
1
2
3
CNV
n= 374
CYC1 Kaplan–Meier survival
pooled LGG+GBM, Etoposide exposure
0.0
0.2
0.4
0.6
0.8
1.0
0
500
1000
2000
3000
CYC1 expression and CNV for
pooled LGG+GBM, Etoposide exposure
Expression in log2(x+1)
10.5
11.0
11.5
12.0
12.5
1
2
3
CNV
n= 21

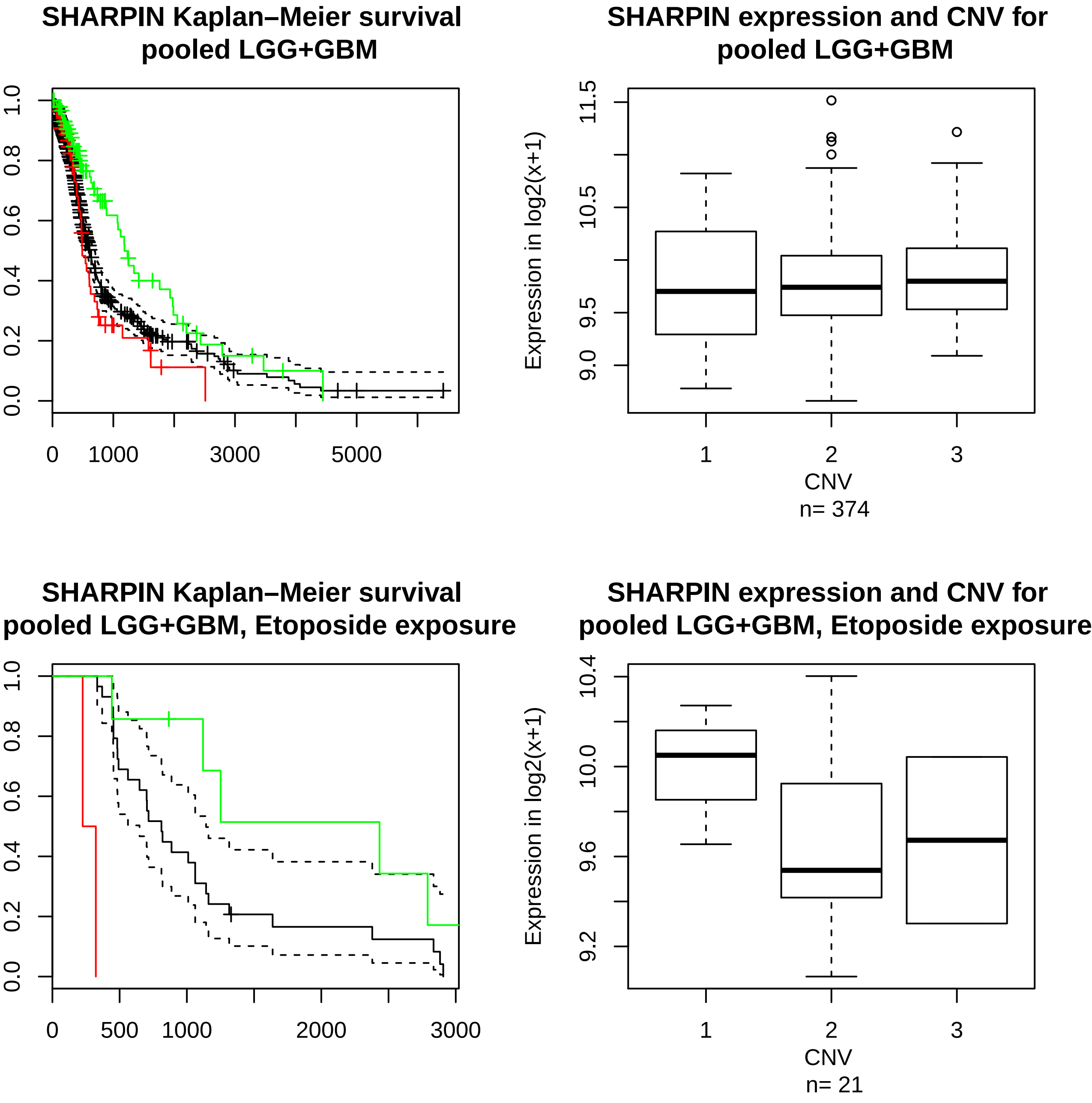
SHARPIN Kaplan–Meier survival
pooled LGG+GBM
0.0 0.2 0.4 0.6 0.8 1.0
0 1000 3000 5000
SHARPIN expression and CNV for
pooled LGG+GBM
Expression in log2(x+1)
9.0 9.5 10.5 11.5
1 2 3
CNV
n= 374
SHARPIN Kaplan–Meier survival
pooled LGG+GBM, Etoposide exposure
0.0 0.2 0.4 0.6 0.8 1.0
0 500 1000 2000 3000
SHARPIN expression and CNV for
pooled LGG+GBM, Etoposide exposure
Expression in log2(x+1)
9.2 9.6 10.0 10.4
1 2 3
CNV
n= 21

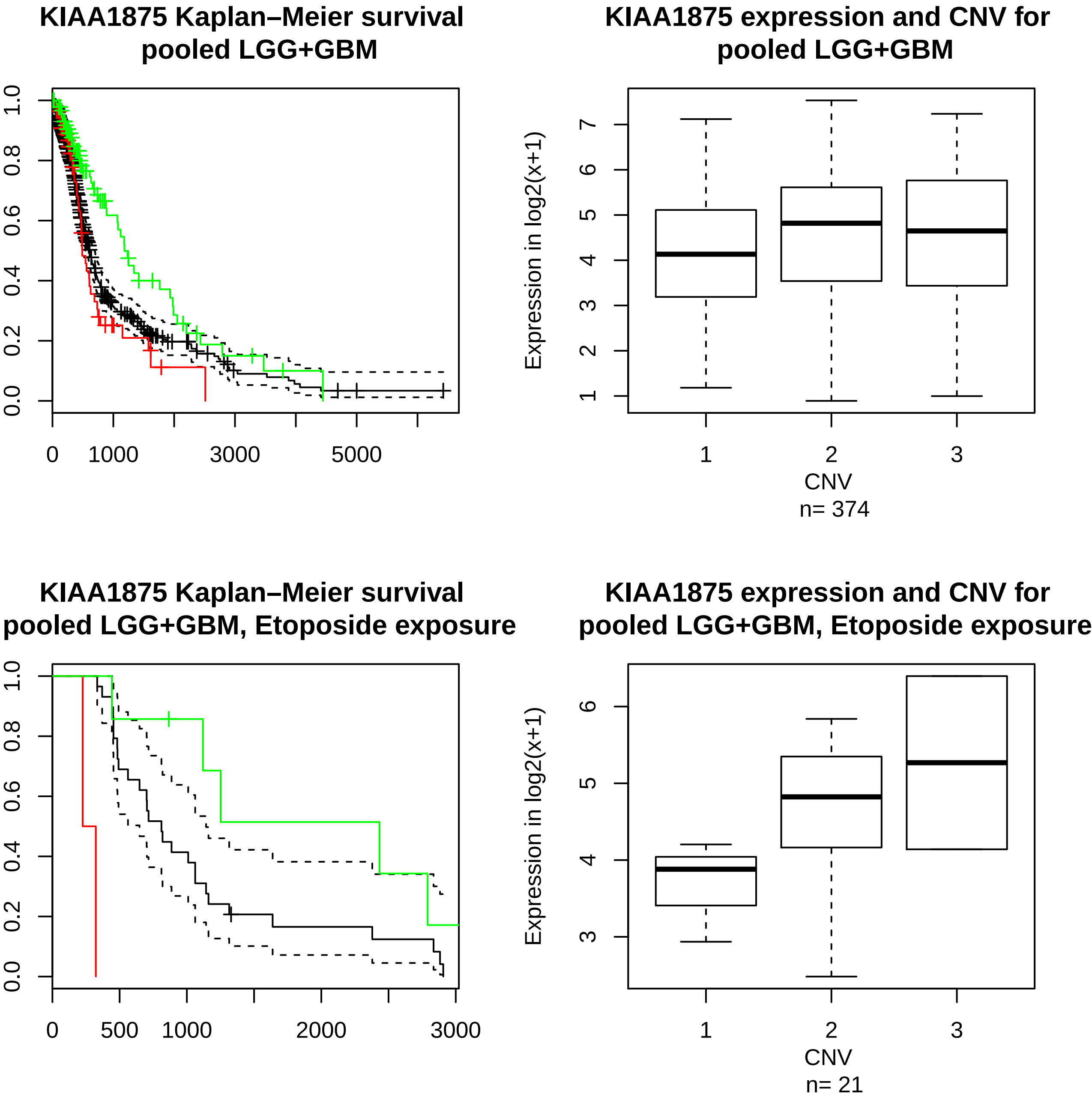
KIAA1875 Kaplan–Meier survival
pooled LGG+GBM
0.0
0.2
0.4
0.6
0.8
1.0
0
1000
3000
5000
KIAA1875 expression and CNV for
pooled LGG+GBM
Expression in log2(x+1)
1
2
3
4
5
6
7
1
2
3
CNV
n= 374
KIAA1875 Kaplan–Meier survival
pooled LGG+GBM, Etoposide exposure
0.0
0.2
0.4
0.6
0.8
1.0
0
500
1000
2000
3000
KIAA1875 expression and CNV for
pooled LGG+GBM, Etoposide exposure
Expression in log2(x+1)
3
4
5
6
1
2
3
CNV
n= 21

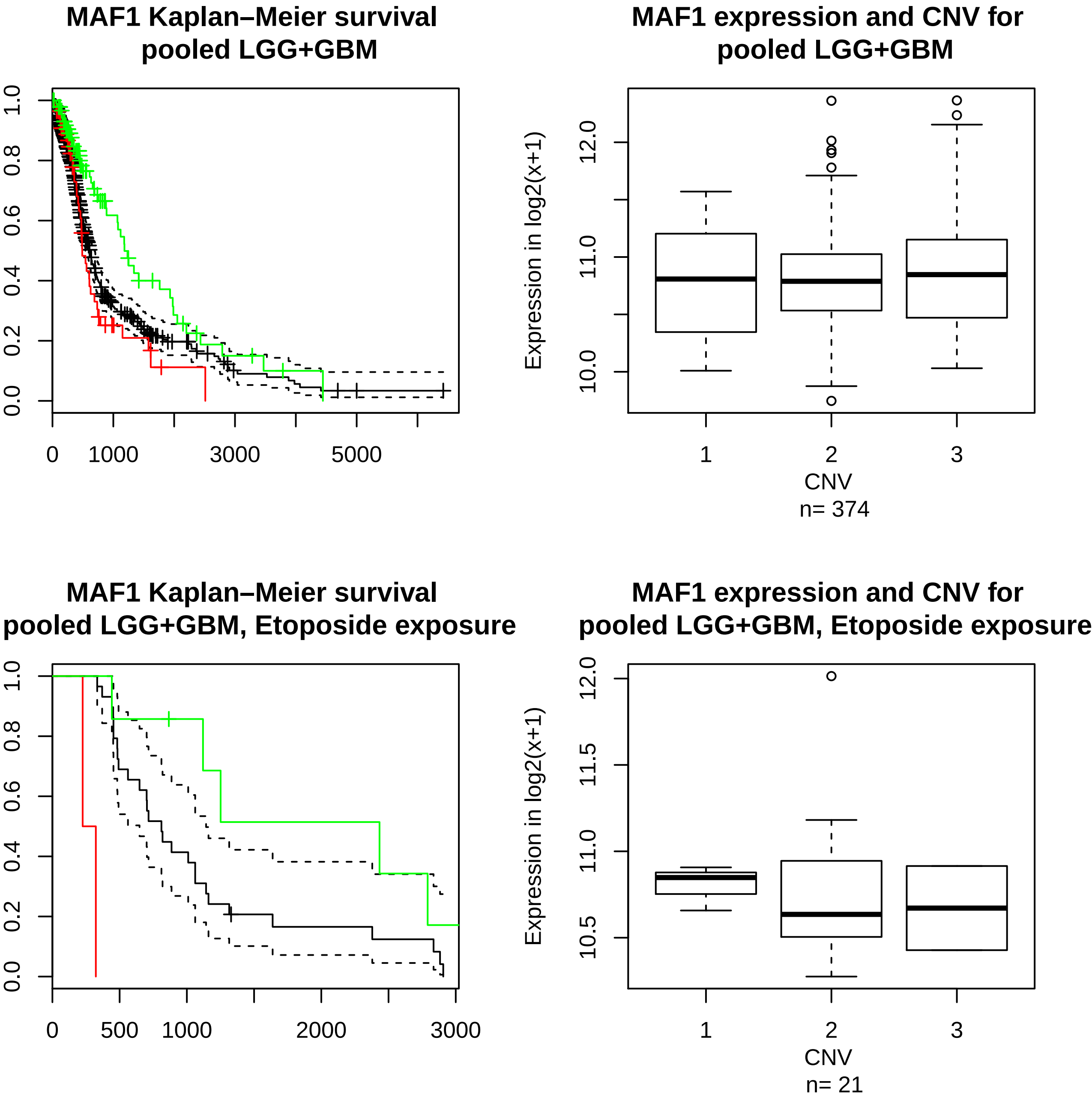

MAF1 Kaplan–Meier survival
pooled LGG+GBM
0.0
0.2
0.4
0.6
0.8
1.0
0
1000
3000
5000
MAF1 expression and CNV for
pooled LGG+GBM
Expression in log2(x+1)
10.0
11.0
12.0
1
2
3
CNV
n= 374
MAF1 Kaplan–Meier survival
pooled LGG+GBM, Etoposide exposure
0.0
0.2
0.4
0.6
0.8
1.0
0
500
1000
2000
3000
MAF1 expression and CNV for
pooled LGG+GBM, Etoposide exposure
Expression in log2(x+1)
10.5
11.0
11.5
12.0
1
2
3
CNV
n= 21

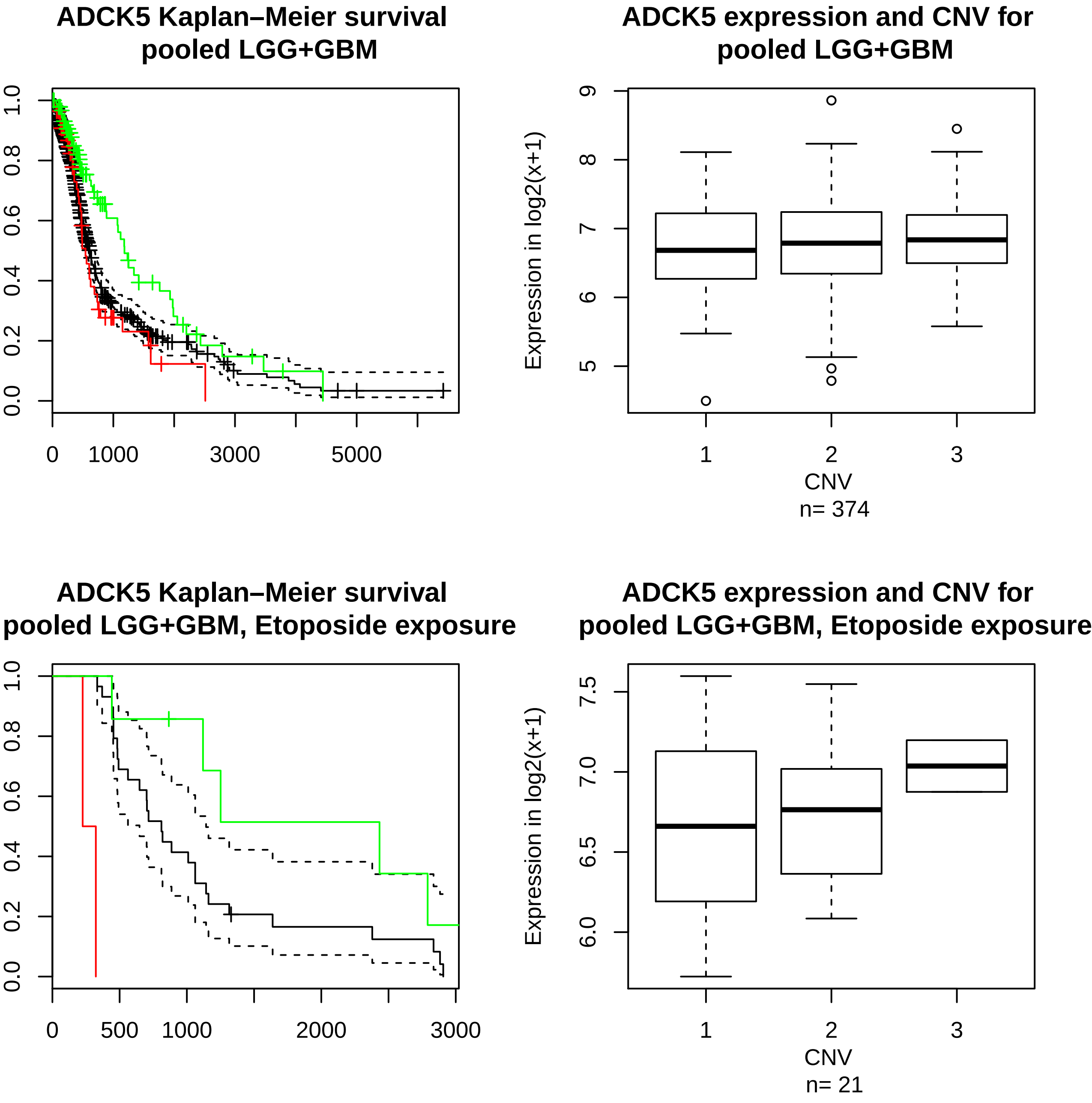
ADCK5 Kaplan–Meier survival
pooled LGG+GBM
0.0
0.2
0.4
0.6
0.8
1.0
0
1000
3000
5000
ADCK5 expression and CNV for
pooled LGG+GBM
Expression in log2(x+1)
5
6
7
8
9
1
2
3
CNV
n= 374
ADCK5 Kaplan–Meier survival
pooled LGG+GBM, Etoposide exposure
0.0
0.2
0.4
0.6
0.8
1.0
0
500
1000
2000
3000
ADCK5 expression and CNV for
pooled LGG+GBM, Etoposide exposure
Expression in log2(x+1)
6.0
6.5
7.0
7.5
1
2
3
CNV
n= 21

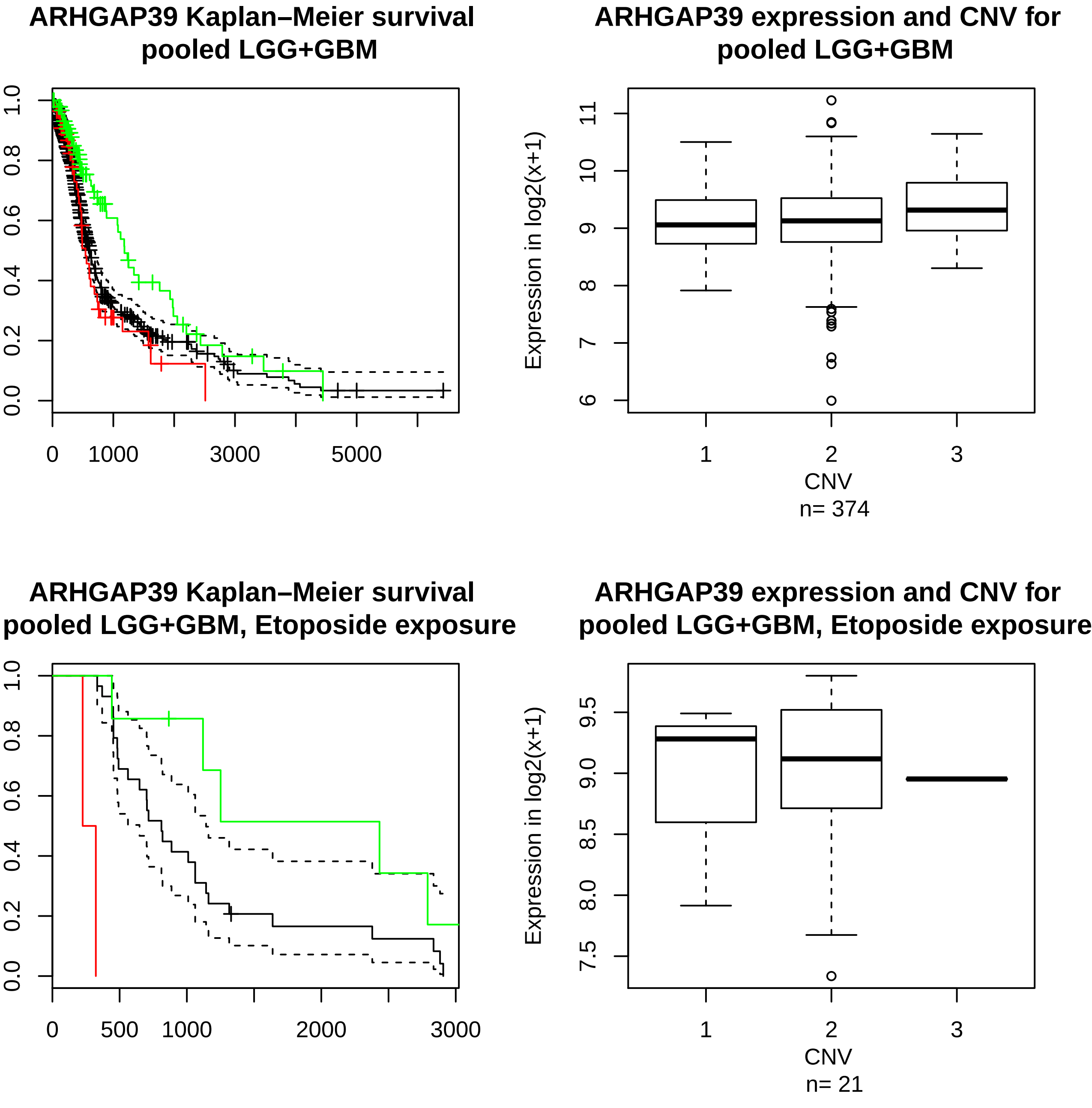
ARHGAP39 Kaplan–Meier survival
pooled LGG+GBM
0.0
0.2
0.4
0.6
0.8
1.0
0
1000
3000
5000
ARHGAP39 expression and CNV for
pooled LGG+GBM
Expression in log2(x+1)
6
7
8
9
10
11
1
2
3
CNV
n= 374
ARHGAP39 Kaplan–Meier survival
pooled LGG+GBM, Etoposide exposure
0.0
0.2
0.4
0.6
0.8
1.0
0
500
1000
2000
3000
ARHGAP39 expression and CNV for
pooled LGG+GBM, Etoposide exposure
Expression in log2(x+1)
7.5
8.0
8.5
9.0
9.5
1
2
3
CNV
n= 21

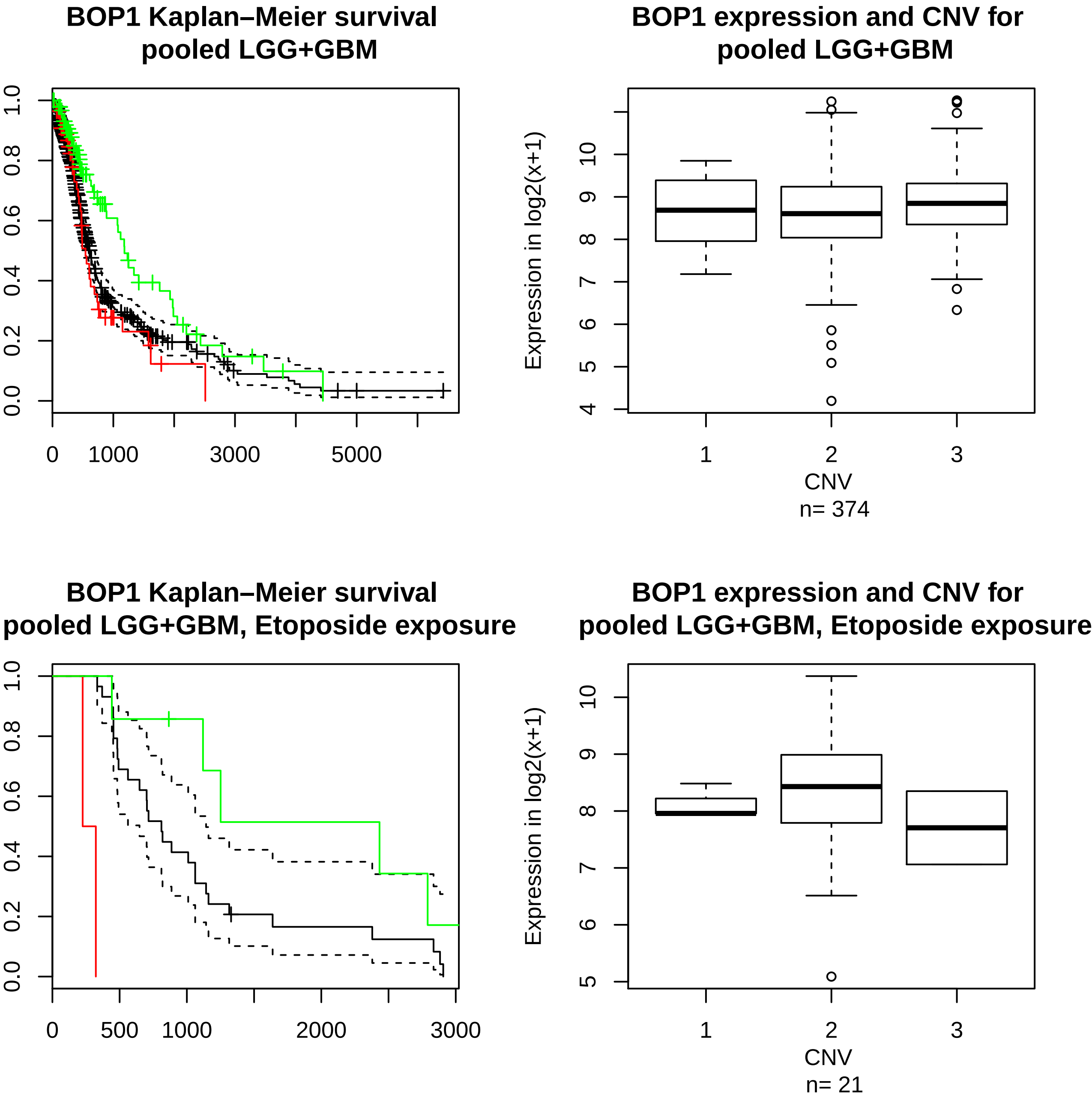

BOP1 Kaplan–Meier survival
pooled LGG+GBM
0.0
0.2
0.4
0.6
0.8
1.0
0
1000
3000
5000
BOP1 expression and CNV for
pooled LGG+GBM
Expression in log2(x+1)
4
5
6
7
8
9
10
1
2
3
CNV
n= 374
BOP1 Kaplan–Meier survival
pooled LGG+GBM, Etoposide exposure
0.0
0.2
0.4
0.6
0.8
1.0
0
500
1000
2000
3000
BOP1 expression and CNV for
pooled LGG+GBM, Etoposide exposure
Expression in log2(x+1)
5
6
7
8
9
10
1
2
3
CNV
n= 21

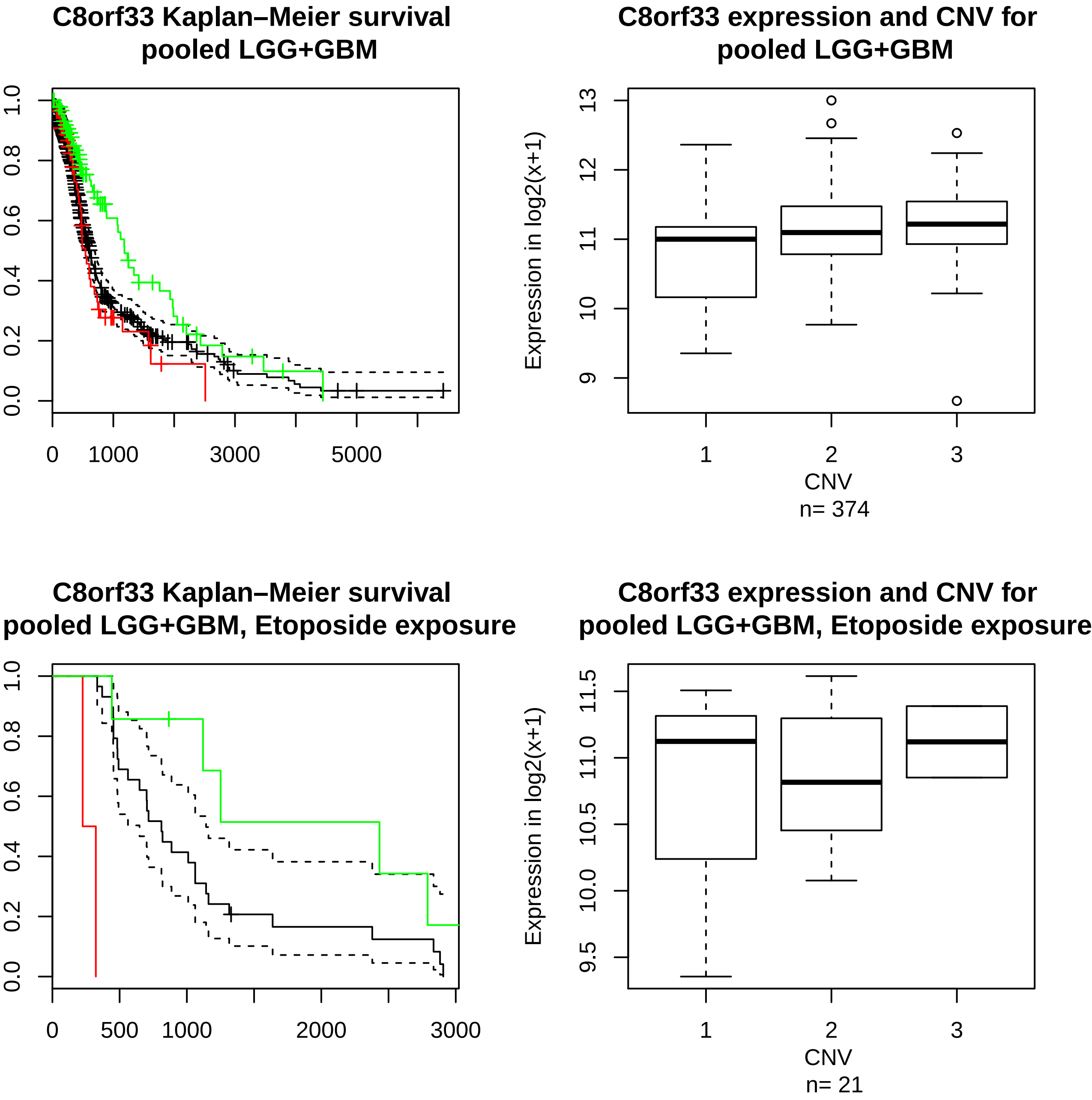
C8orf33 Kaplan–Meier survival
pooled LGG+GBM
0.0
0.2
0.4
0.6
0.8
1.0
0
1000
3000
5000
C8orf33 expression and CNV for
pooled LGG+GBM
Expression in log2(x+1)
9
10
11
12
13
1
2
3
CNV
n= 374
C8orf33 Kaplan–Meier survival
pooled LGG+GBM, Etoposide exposure
0.0
0.2
0.4
0.6
0.8
1.0
0
500
1000
2000
3000
C8orf33 expression and CNV for
pooled LGG+GBM, Etoposide exposure
Expression in log2(x+1)
9.5
10.0
10.5
11.0
11.5
1
2
3
CNV
n= 21

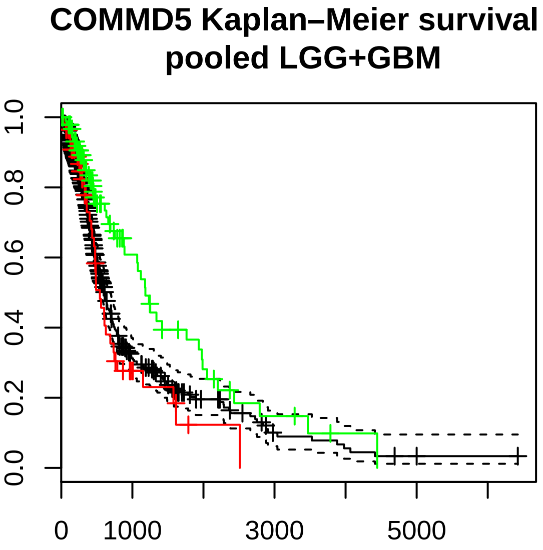
COMMD5 Kaplan–Meier survival
pooled LGG+GBM
0.0
0.2
0.4
0.6
0.8
1.0
0
1000
3000
5000

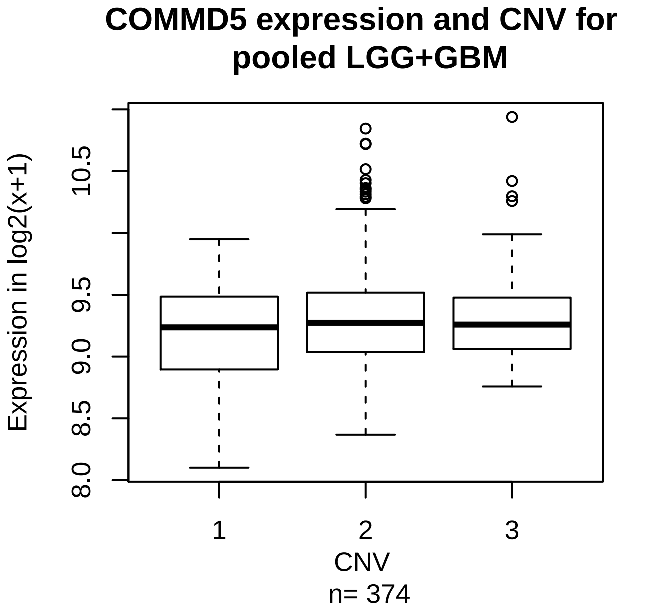
COMMD5 expression and CNV for
pooled LGG+GBM
Expression in log2(x+1)
8.0
8.5
9.0
9.5
10.5
1
2
3
CNV
n= 374

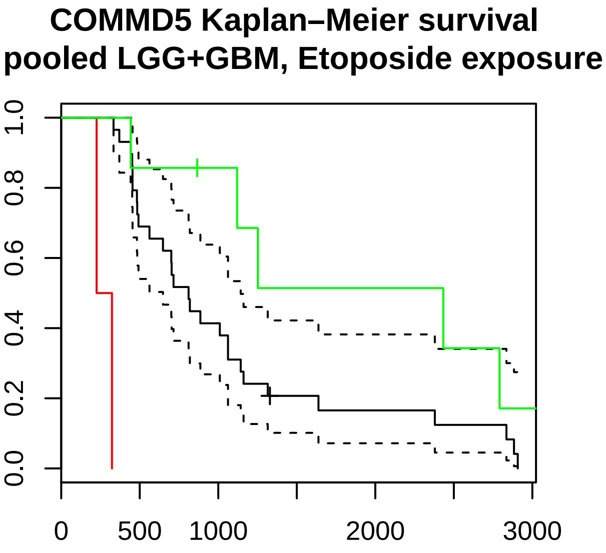
COMMD5 Kaplan–Meier survival
pooled LGG+GBM, Etoposide exposure
0.0
0.2
0.4
0.6
0.8
1.0
0
500
1000
2000
3000

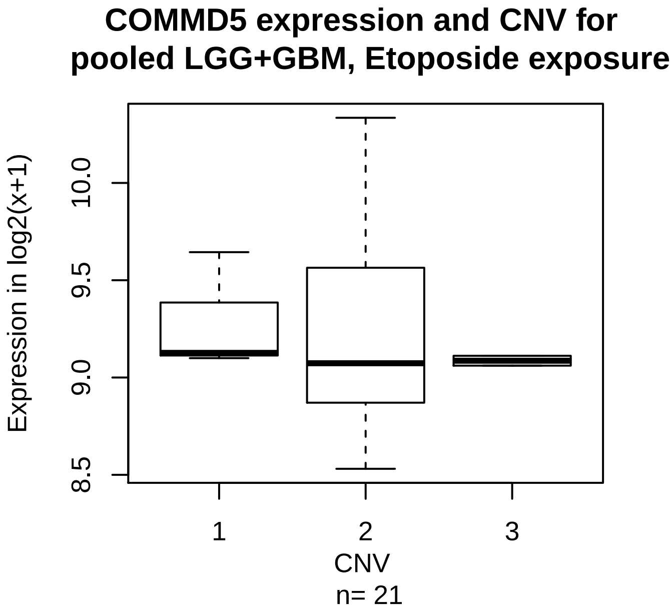
COMMD5 expression and CNV for
pooled LGG+GBM, Etoposide exposure
Expression in log2(x+1)
8.5
9.0
9.5
10.0
1
2
3
CNV
n= 21

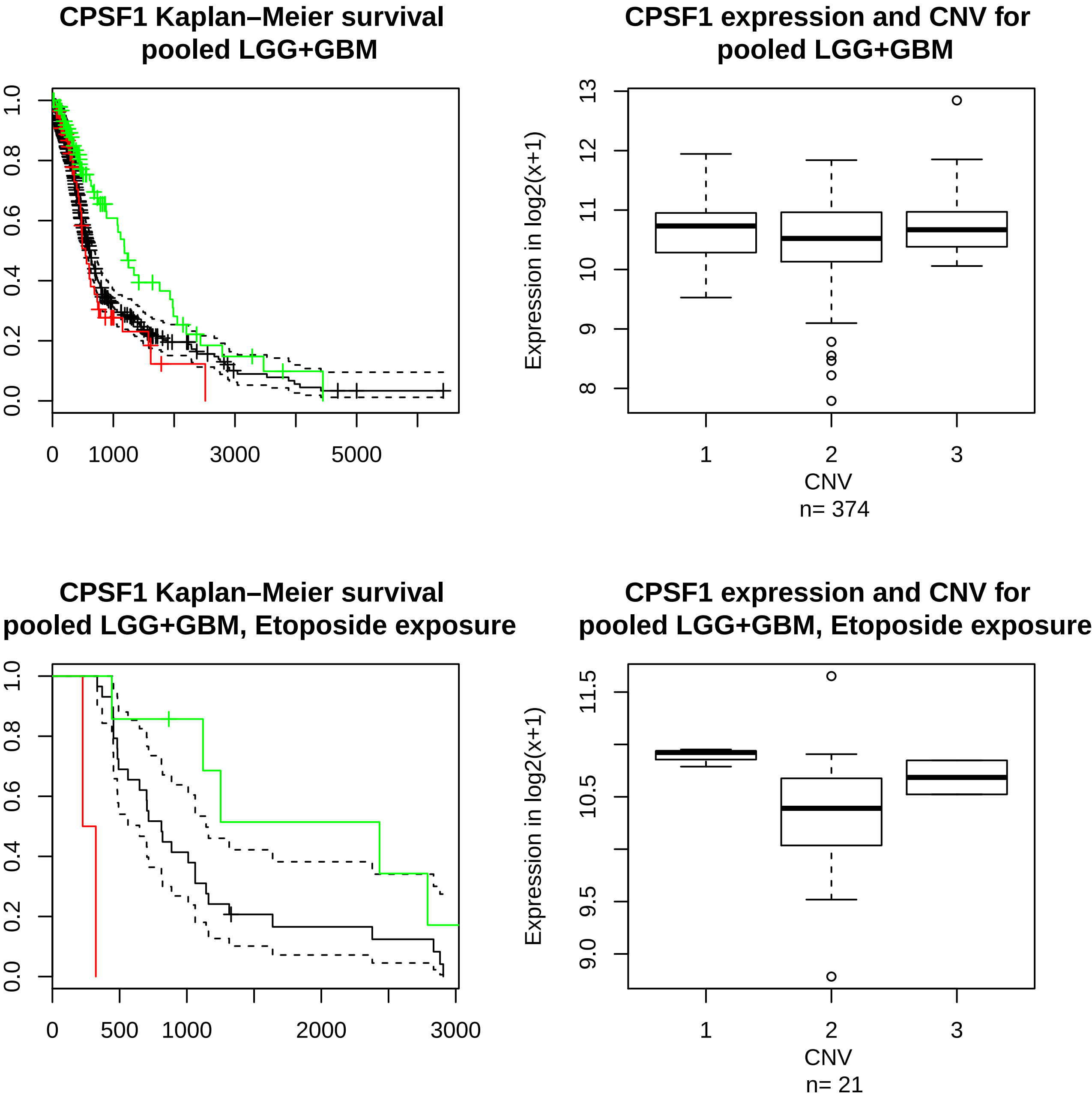

CPSF1 Kaplan–Meier survival
pooled LGG+GBM
0.0
0.2
0.4
0.6
0.8
1.0
0
1000
3000
5000
CPSF1 expression and CNV for
pooled LGG+GBM
Expression in log2(x+1)
8
9
10
11
12
13
1
2
3
CNV
n= 374
CPSF1 Kaplan–Meier survival
pooled LGG+GBM, Etoposide exposure
0.0
0.2
0.4
0.6
0.8
1.0
0
500
1000
2000
3000
CPSF1 expression and CNV for
pooled LGG+GBM, Etoposide exposure
Expression in log2(x+1)
9.0
9.5
10.5
11.5
1
2
3
CNV
n= 21

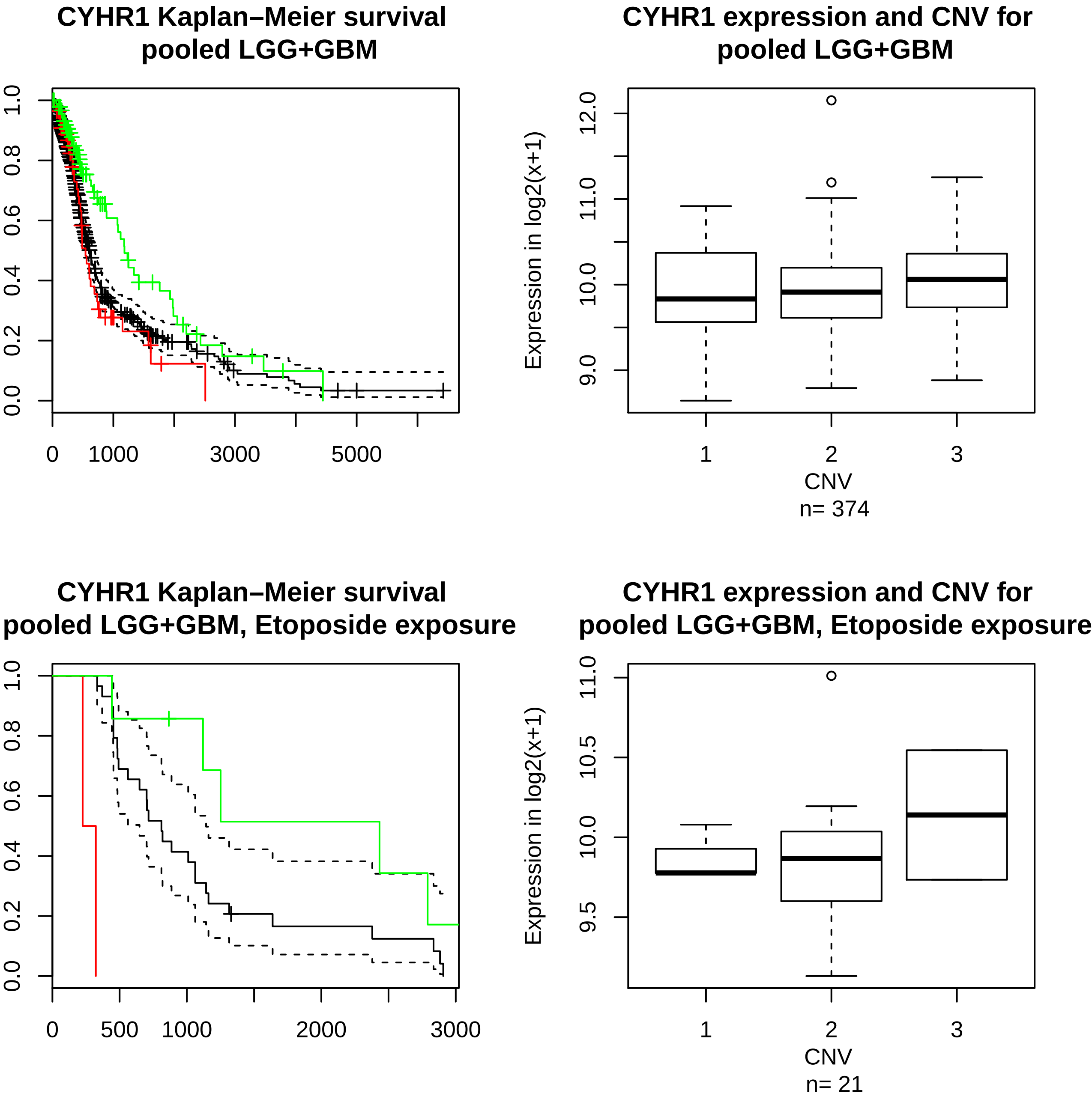

CYHR1 Kaplan–Meier survival
pooled LGG+GBM
0.0
0.2
0.4
0.6
0.8
1.0
0
1000
3000
5000
CYHR1 expression and CNV for
pooled LGG+GBM
Expression in log2(x+1)
9.0
10.0
11.0
12.0
1
2
3
CNV
n= 374
CYHR1 Kaplan–Meier survival
pooled LGG+GBM, Etoposide exposure
0.0
0.2
0.4
0.6
0.8
1.0
0
500
1000
2000
3000
CYHR1 expression and CNV for
pooled LGG+GBM, Etoposide exposure
Expression in log2(x+1)
9.5
10.0
10.5
11.0
1
2
3
CNV
n= 21

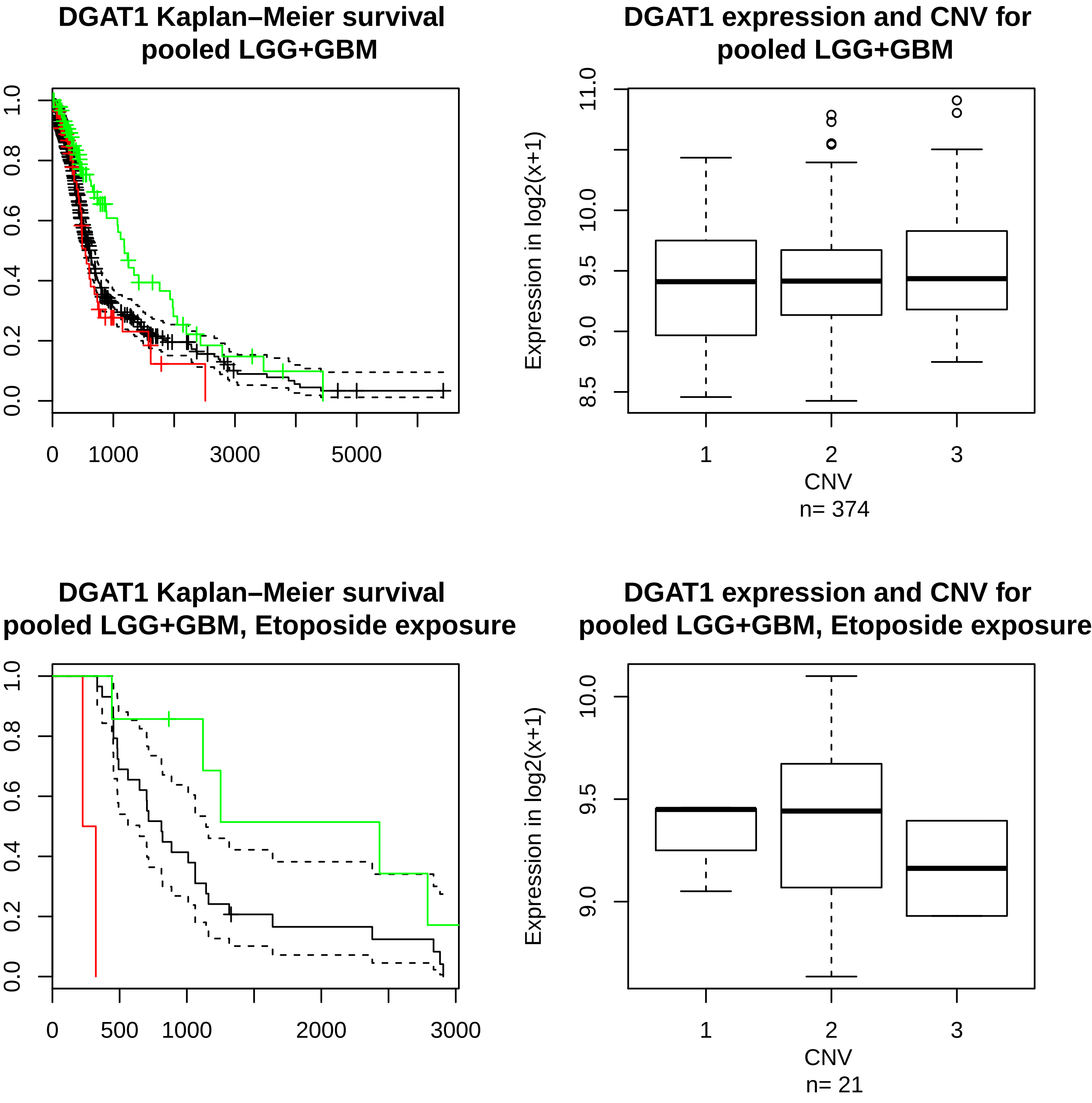
DGAT1 Kaplan–Meier survival
pooled LGG+GBM
0.0
0.2
0.4
0.6
0.8
1.0
0
1000
3000
5000
DGAT1 expression and CNV for
pooled LGG+GBM
Expression in log2(x+1)
8.5
9.0
9.5
10.0
11.0
1
2
3
CNV
n= 374
DGAT1 Kaplan–Meier survival
pooled LGG+GBM, Etoposide exposure
0.0
0.2
0.4
0.6
0.8
1.0
0
500
1000
2000
3000
DGAT1 expression and CNV for
pooled LGG+GBM, Etoposide exposure
Expression in log2(x+1)
9.0
9.5
10.0
1
2
3
CNV
n= 21

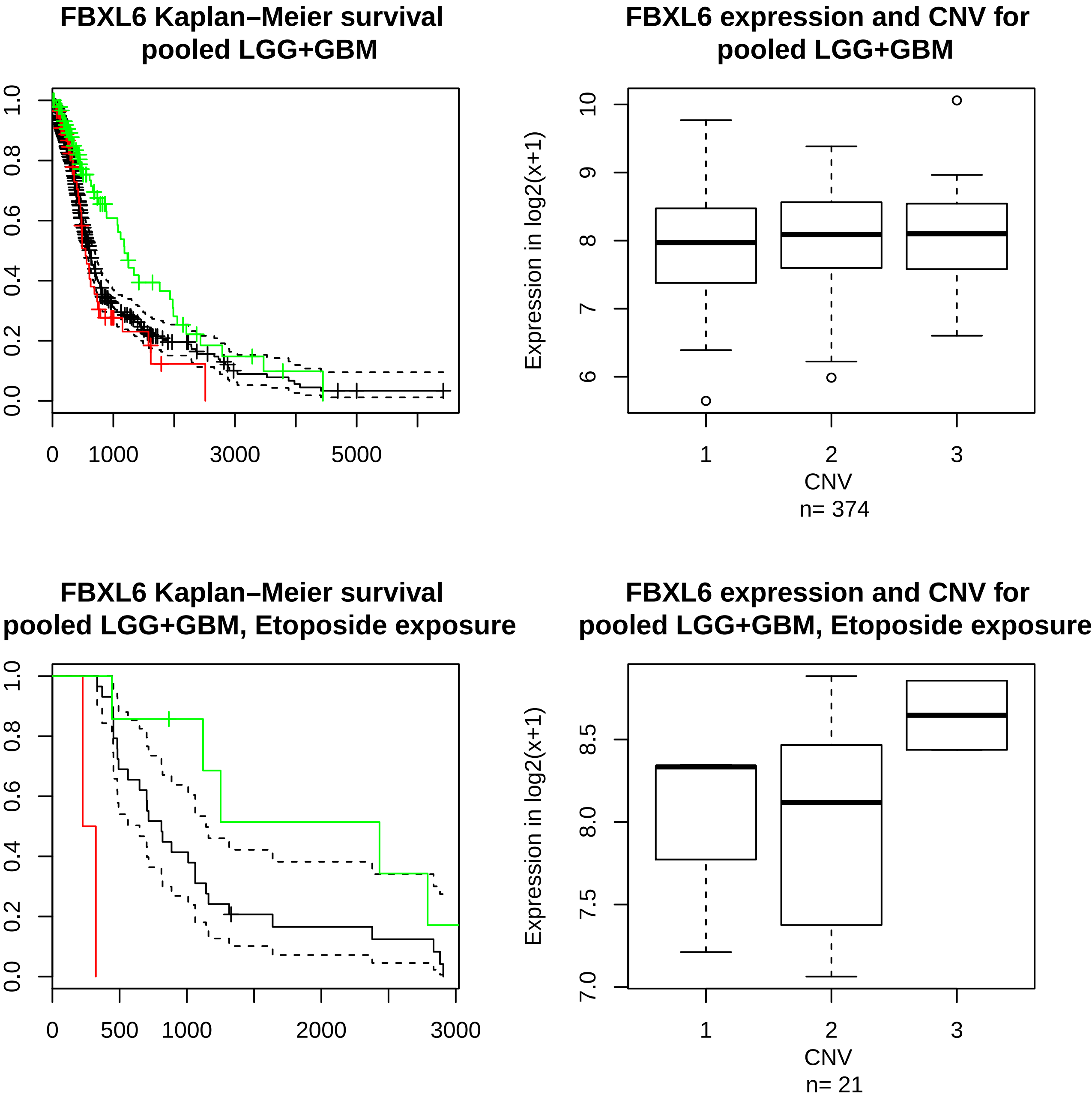
FBXL6 Kaplan–Meier survival
pooled LGG+GBM
0.0
0.2
0.4
0.6
0.8
1.0
0
1000
3000
5000
FBXL6 expression and CNV for
pooled LGG+GBM
Expression in log2(x+1)
6
7
8
9
10
1
2
3
CNV
n= 374
FBXL6 Kaplan–Meier survival
pooled LGG+GBM, Etoposide exposure
0.0
0.2
0.4
0.6
0.8
1.0
0
500
1000
2000
3000
FBXL6 expression and CNV for
pooled LGG+GBM, Etoposide exposure
Expression in log2(x+1)
7.0
7.5
8.0
8.5
1
2
3
CNV
n= 21

FOXH1 Kaplan–Meier survival pooled LGG+GBM

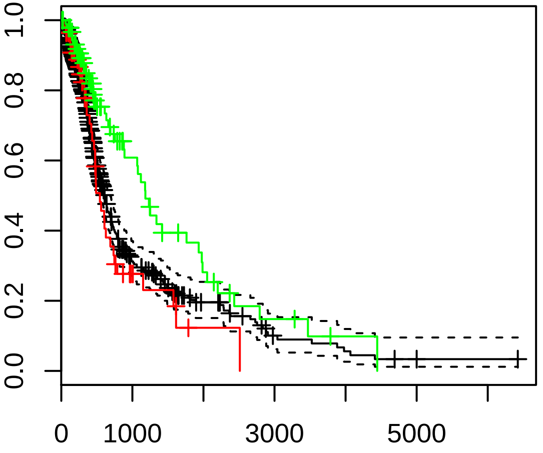

FOXH1 expression and CNV for pooled LGG+GBM

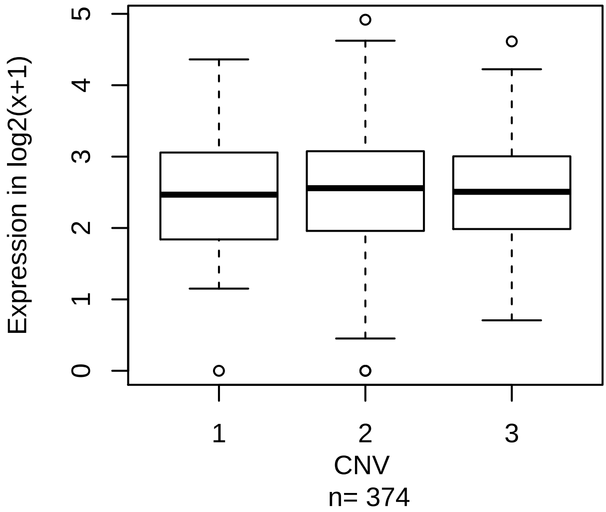

FOXH1 Kaplan–Meier survival pooled LGG+GBM, Etoposide exposure

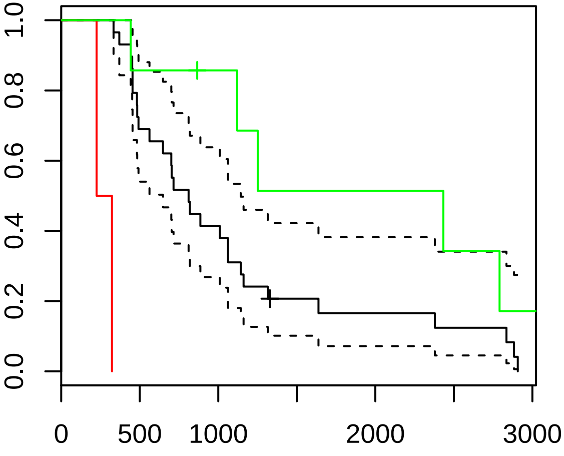

FOXH1 expression and CNV for pooled LGG+GBM, Etoposide exposure

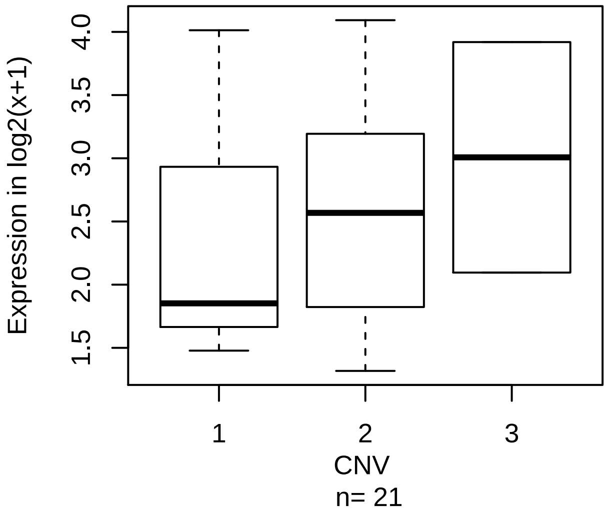

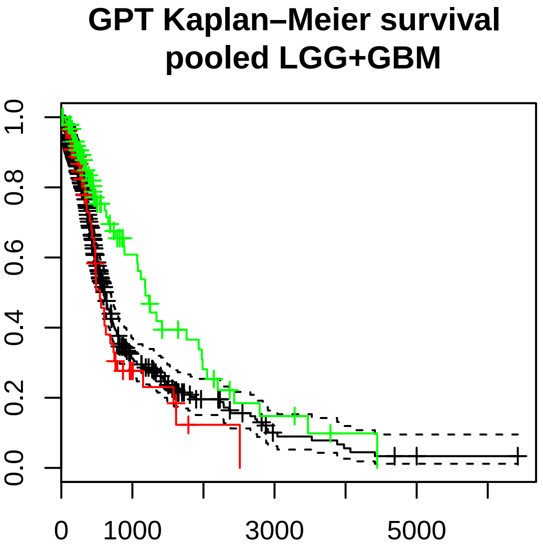
GPT Kaplan–Meier survival
pooled LGG+GBM
0.0
0.2
0.4
0.6
0.8
1.0
0
1000
3000
5000

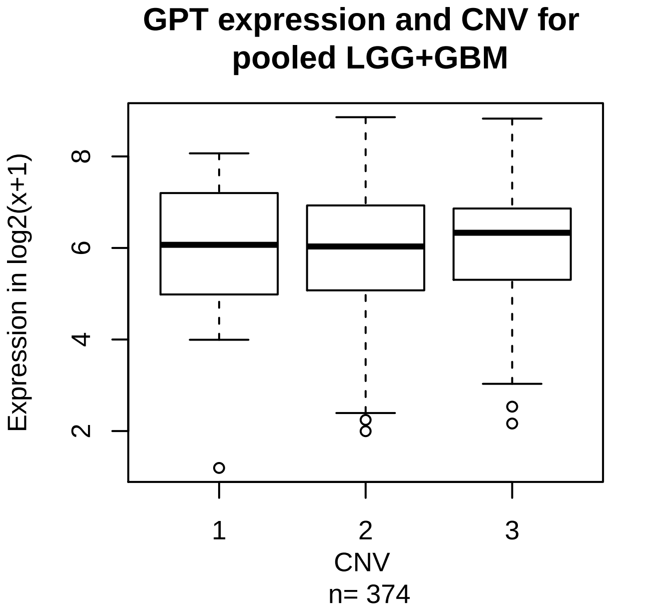
GPT expression and CNV for
pooled LGG+GBM
Expression in log2(x+1)
2
4
6
8
1
2
3
CNV
n= 374

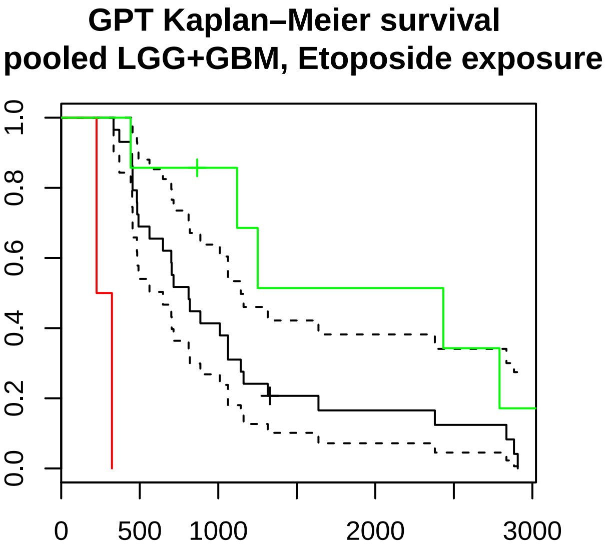
GPT Kaplan–Meier survival
pooled LGG+GBM, Etoposide exposure
0.0
0.2
0.4
0.6
0.8
1.0
0
500
1000
2000
3000

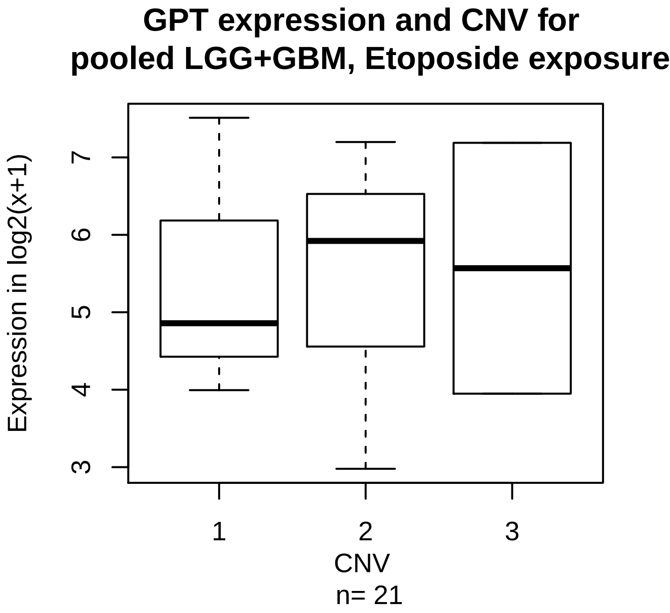
GPT expression and CNV for
pooled LGG+GBM, Etoposide exposure
Expression in log2(x+1)
3
4
5
6
7
1
2
3
CNV
n= 21

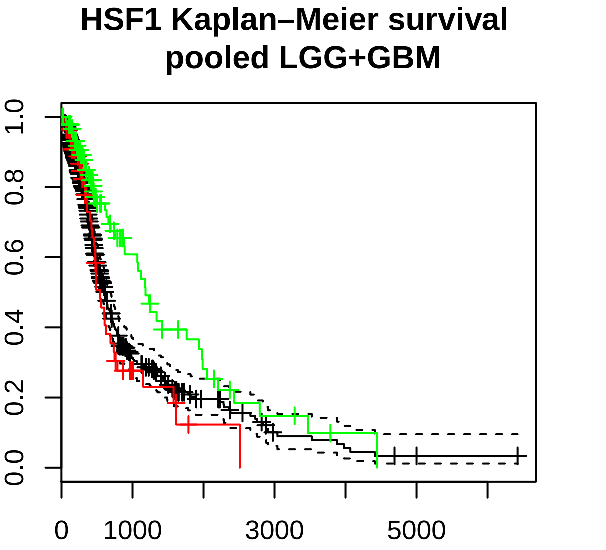

HSF1 Kaplan–Meier survival
pooled LGG+GBM
0.0
0.2
0.4
0.6
0.8
1.0
0
1000
3000
5000

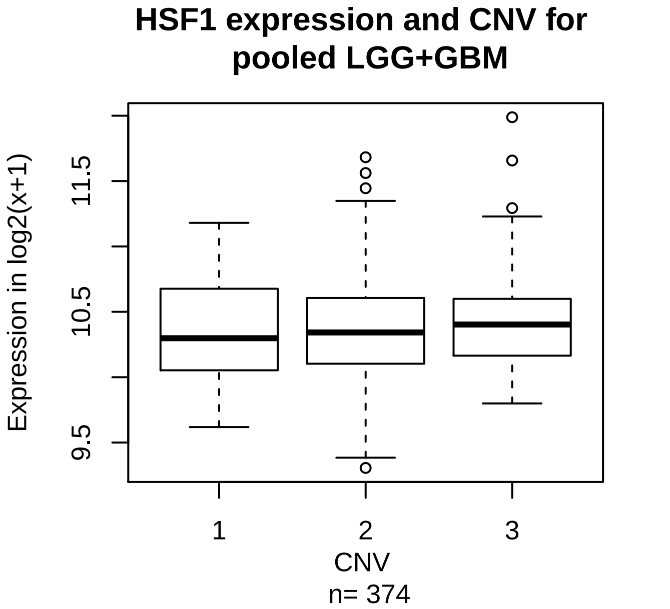

HSF1 expression and CNV for
pooled LGG+GBM
Expression in log2(x+1)
9.5
10.5
11.5
1
2
3
CNV
n= 374

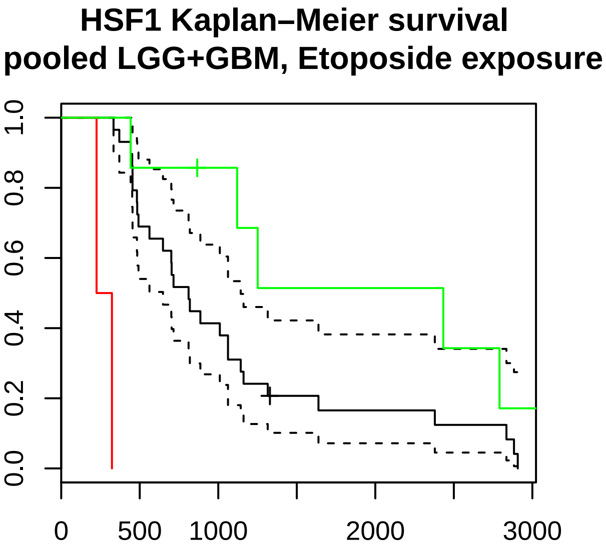

HSF1 Kaplan–Meier survival
pooled LGG+GBM, Etoposide exposure
0.0
0.2
0.4
0.6
0.8
1.0
0
500
1000
2000
3000

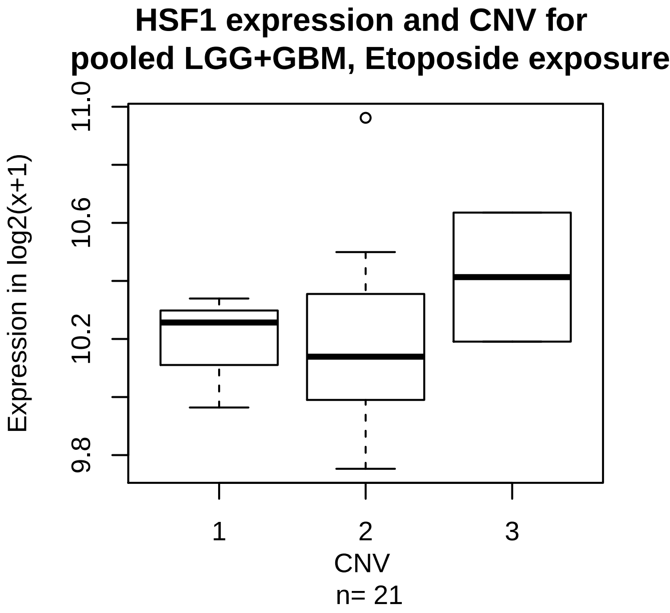

HSF1 expression and CNV for
pooled LGG+GBM, Etoposide exposure
Expression in log2(x+1)
9.8
10.2
10.6
11.0
1
2
3
CNV
n= 21

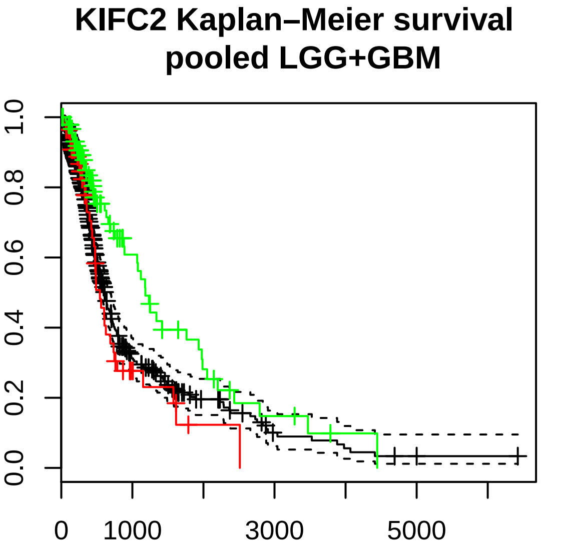
KIFC2 Kaplan–Meier survival
pooled LGG+GBM
0.0
0.2
0.4
0.6
0.8
1.0
0
1000
3000
5000

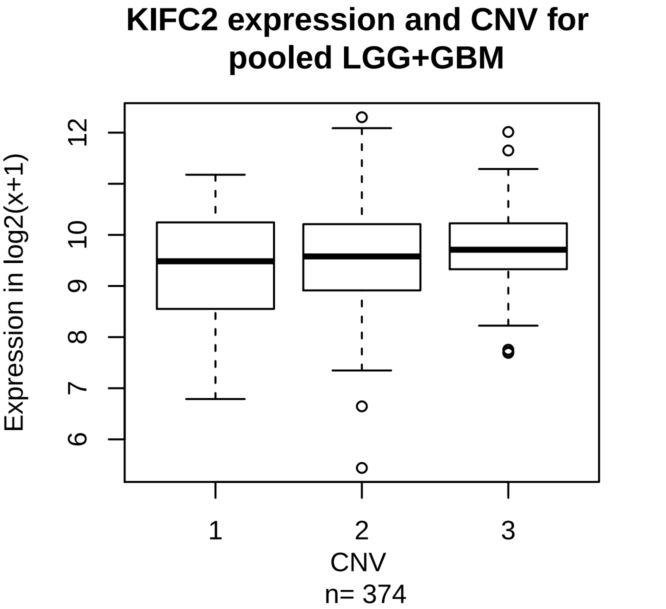
KIFC2 expression and CNV for
pooled LGG+GBM
Expression in log2(x+1)
6
7
8
9
10
12
1
2
3
CNV
n= 374

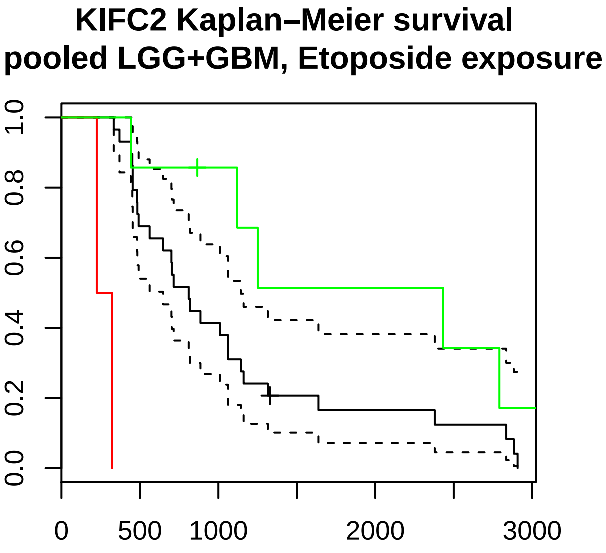
KIFC2 Kaplan–Meier survival
pooled LGG+GBM, Etoposide exposure
0.0
0.2
0.4
0.6
0.8
1.0
0
500
1000
2000
3000

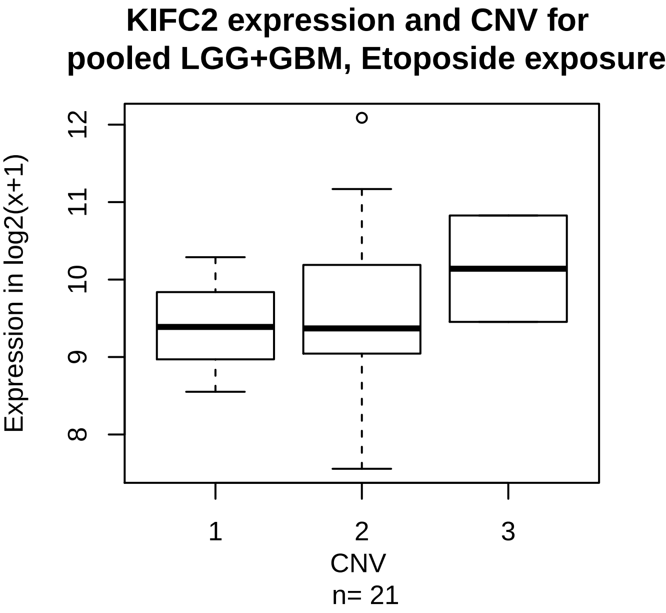
KIFC2 expression and CNV for
pooled LGG+GBM, Etoposide exposure
Expression in log2(x+1)
8
9
10
11
12
1
2
3
CNV
n= 21

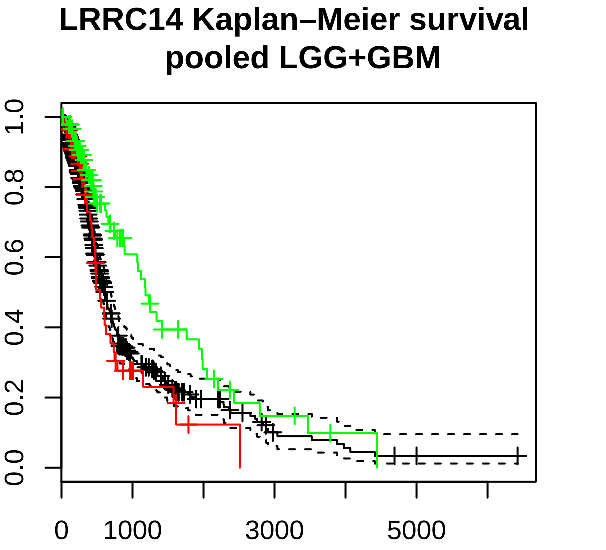
LRRC14 Kaplan–Meier survival
pooled LGG+GBM
0.0
0.2
0.4
0.6
0.8
1.0
0
1000
3000
5000

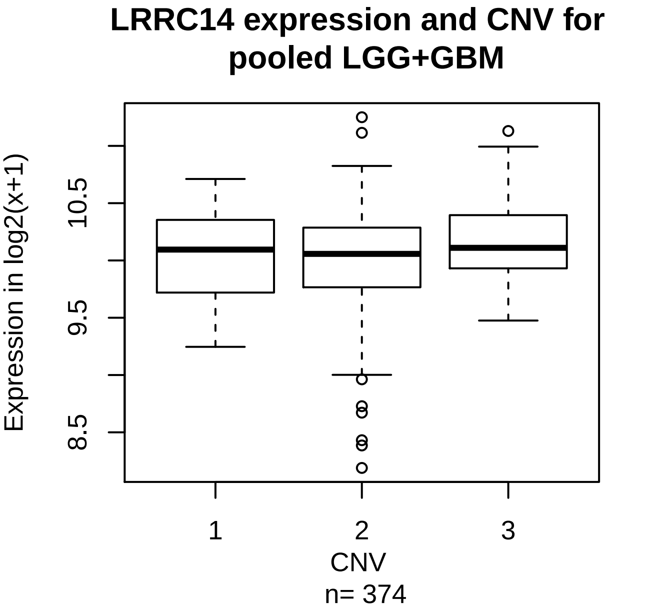
LRRC14 expression and CNV for
pooled LGG+GBM
Expression in log2(x+1)
8.5
9.5
10.5
1
2
3
CNV
n= 374

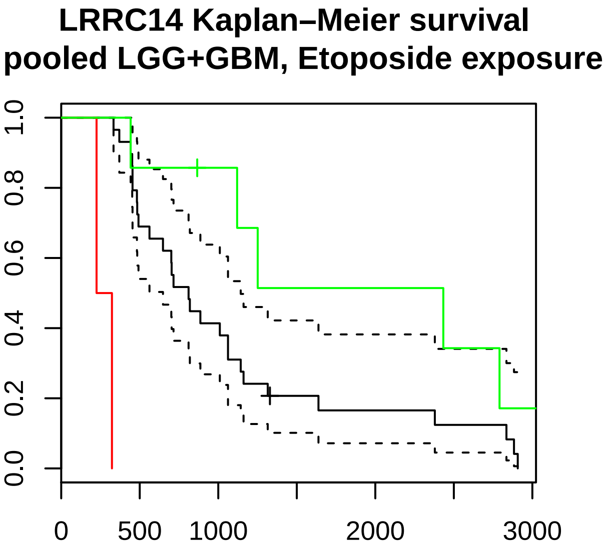
LRRC14 Kaplan–Meier survival
pooled LGG+GBM, Etoposide exposure
0.0
0.2
0.4
0.6
0.8
1.0
0
500
1000
2000
3000

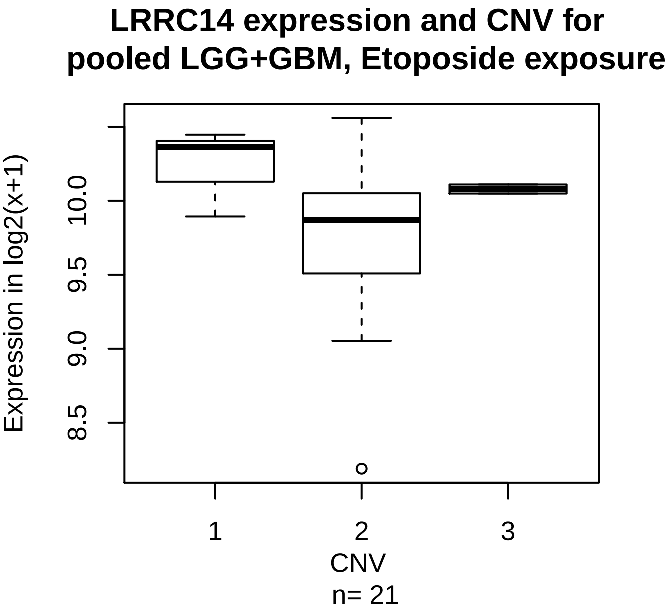
LRRC14 expression and CNV for
pooled LGG+GBM, Etoposide exposure
Expression in log2(x+1)
8.5
9.0
9.5
10.0
1
2
3
CNV
n= 21

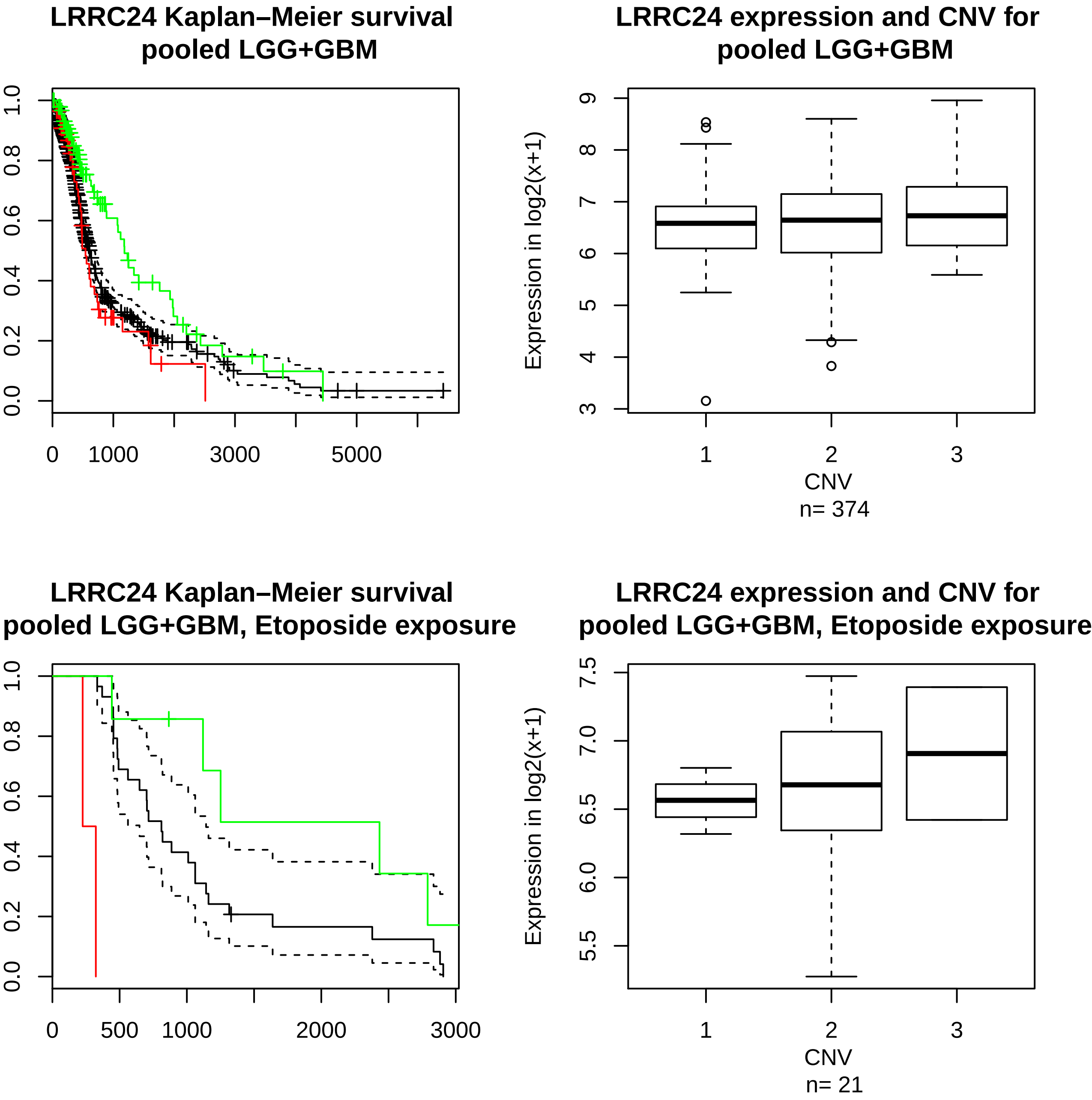
LRRC24 Kaplan–Meier survival
pooled LGG+GBM
0.0
0.2
0.4
0.6
0.8
1.0
0
1000
3000
5000
LRRC24 expression and CNV for
pooled LGG+GBM
Expression in log2(x+1)
3
4
5
6
7
8
9
1
2
3
CNV
n= 374
LRRC24 Kaplan–Meier survival
pooled LGG+GBM, Etoposide exposure
0.0
0.2
0.4
0.6
0.8
1.0
0
500
1000
2000
3000
LRRC24 expression and CNV for
pooled LGG+GBM, Etoposide exposure
Expression in log2(x+1)
5.5
6.0
6.5
7.0
7.5
1
2
3
CNV
n= 21

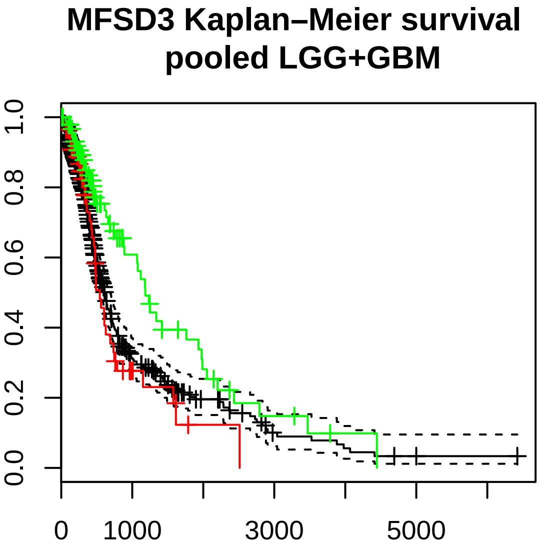
MFSD3 Kaplan–Meier survival
pooled LGG+GBM
0.0
0.2
0.4
0.6
0.8
1.0
0
1000
3000
5000

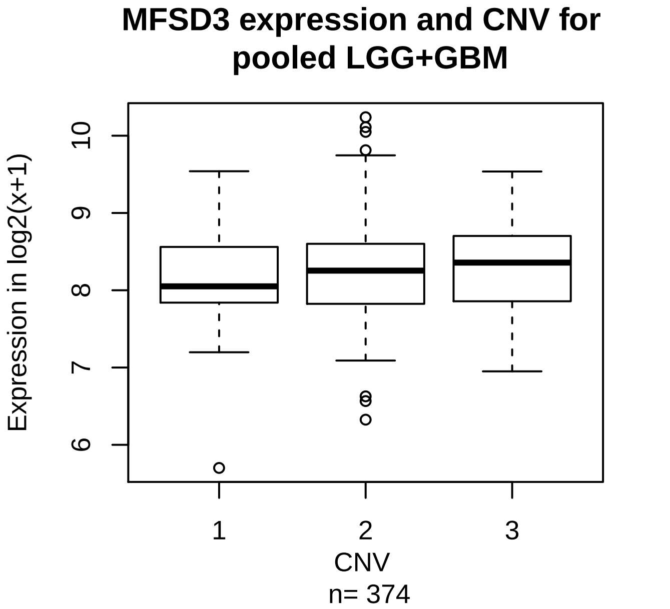
MFSD3 expression and CNV for
pooled LGG+GBM
Expression in log2(x+1)
6
7
8
9
10
1
2
3
CNV
n= 374

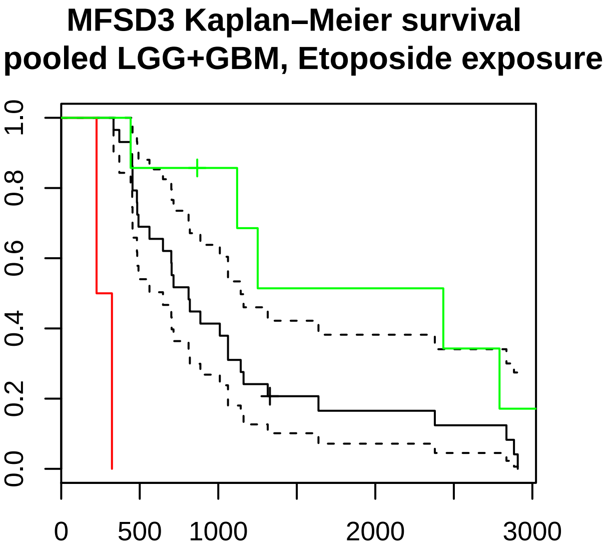
MFSD3 Kaplan–Meier survival
pooled LGG+GBM, Etoposide exposure
0.0
0.2
0.4
0.6
0.8
1.0
0
500
1000
2000
3000

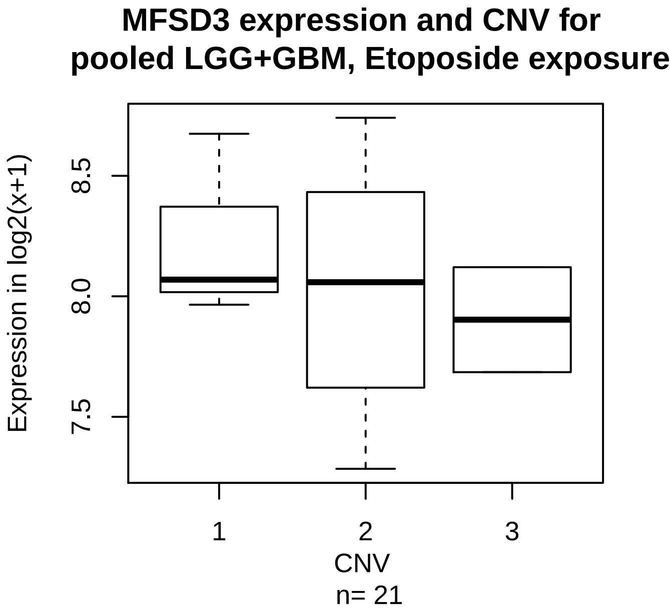
MFSD3 expression and CNV for
pooled LGG+GBM, Etoposide exposure
Expression in log2(x+1)
7.5
8.0
8.5
1
2
3
CNV
n= 21

PPP1R16A Kaplan–Meier survival pooled LGG+GBM

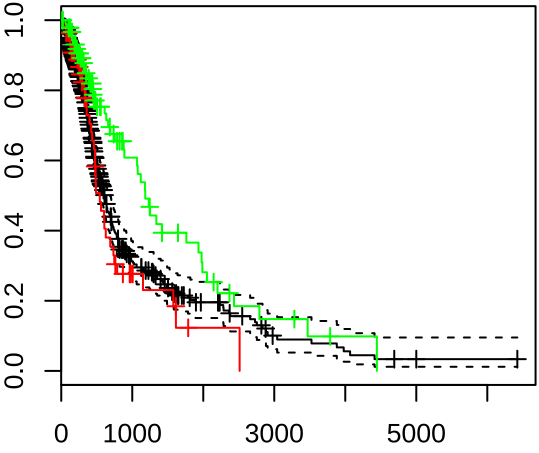

PPP1R16A expression and CNV for pooled LGG+GBM

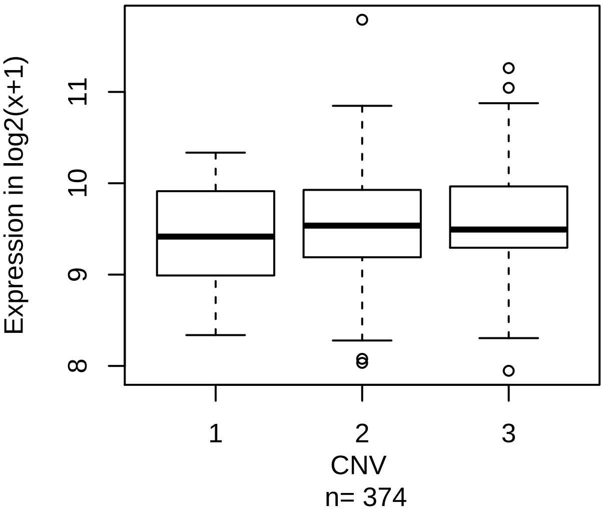

PPP1R16A Kaplan–Meier survival pooled LGG+GBM, Etoposide exposure

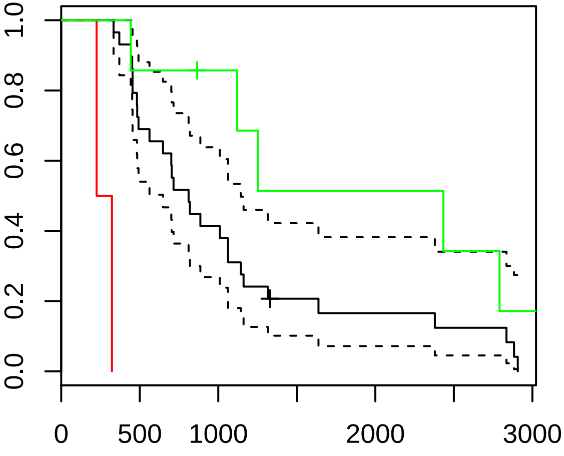

PPP1R16A expression and CNV for pooled LGG+GBM, Etoposide exposure

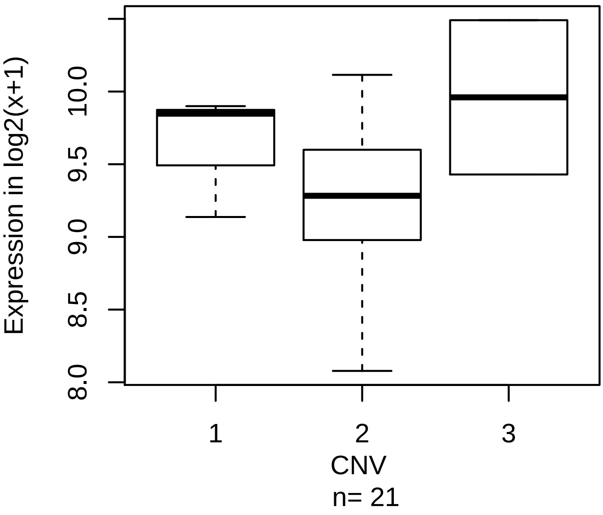

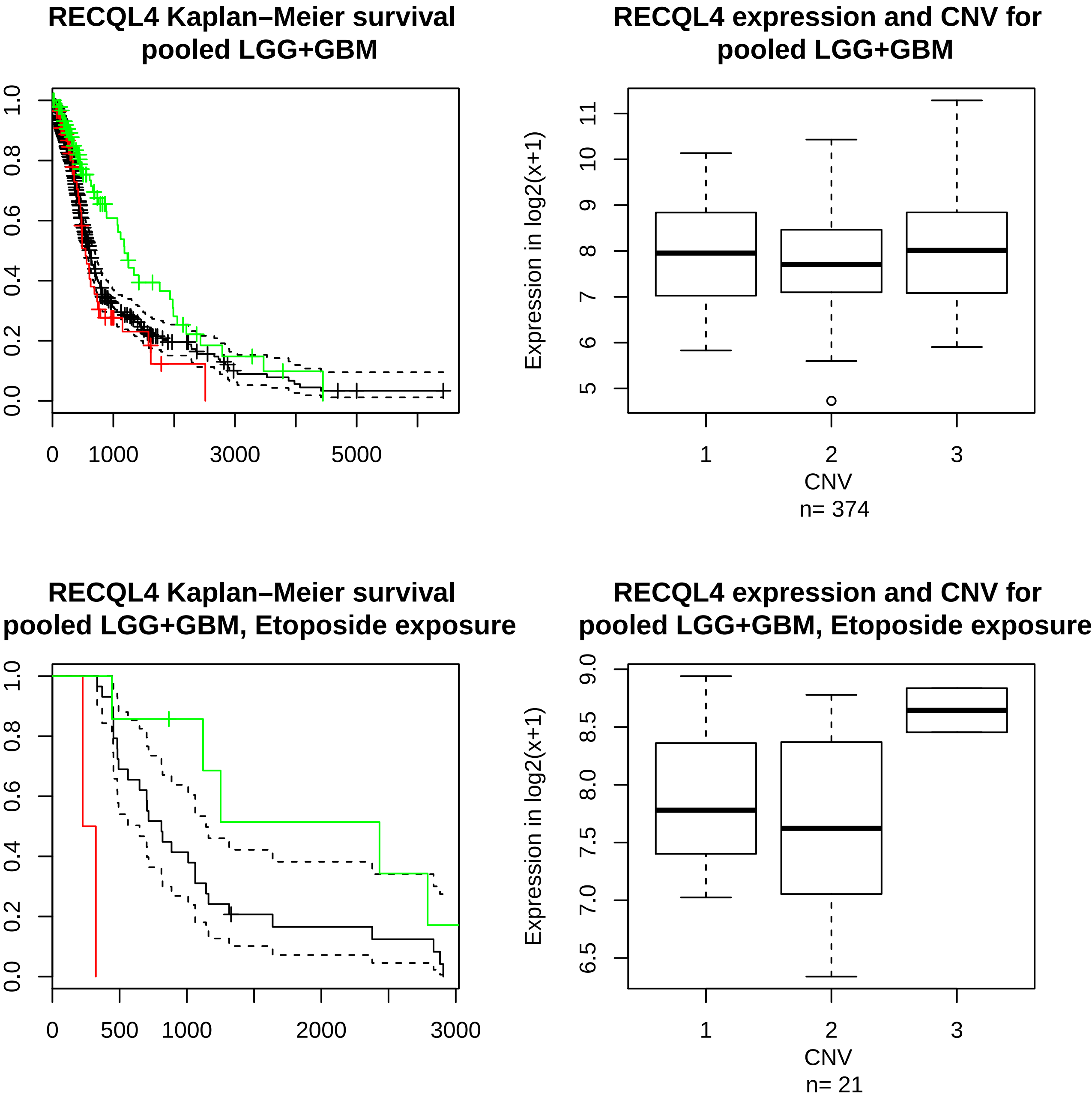
RECQL4 Kaplan–Meier survival
pooled LGG+GBM
0.0 0.2 0.4 0.6 0.8 1.0
0 1000 3000 5000
RECQL4 expression and CNV for
pooled LGG+GBM
Expression in log2(x+1)
5 6 7 8 9 10 11
1 2 3
CNV
n= 374
RECQL4 Kaplan–Meier survival
pooled LGG+GBM, Etoposide exposure
0.0 0.2 0.4 0.6 0.8 1.0
0 500 1000 2000 3000
RECQL4 expression and CNV for
pooled LGG+GBM, Etoposide exposure
Expression in log2(x+1)
6.5 7.0 7.5 8.0 8.5 9.0
1 2 3
CNV
n= 21

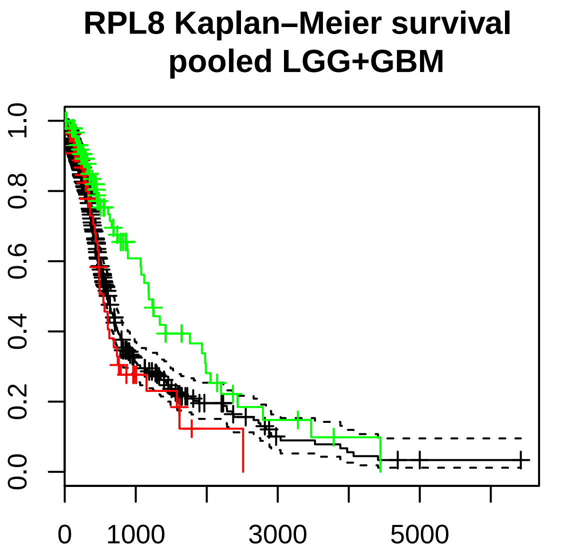
RPL8 Kaplan–Meier survival
pooled LGG+GBM
0.0
0.2
0.4
0.6
0.8
1.0
0
1000
3000
5000

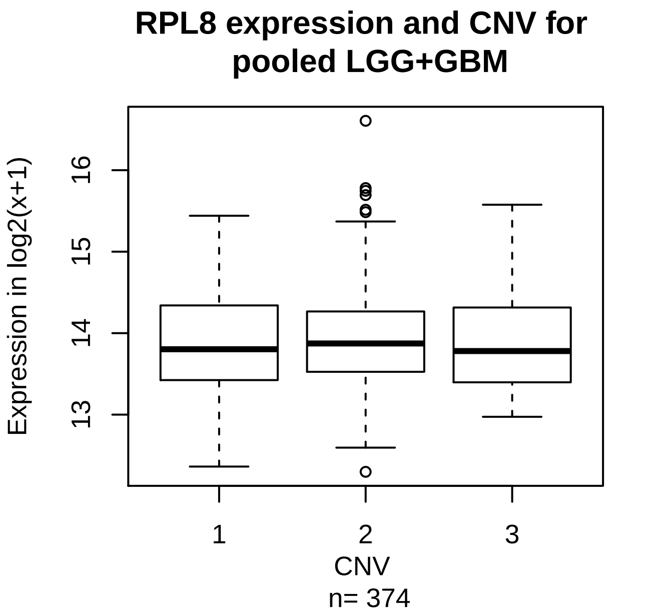
RPL8 expression and CNV for
pooled LGG+GBM
Expression in log2(x+1)
13
14
15
16
1
2
3
CNV
n= 374

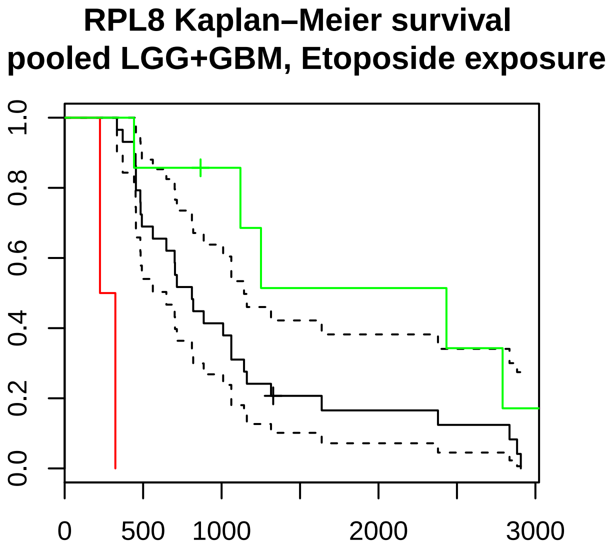
RPL8 Kaplan–Meier survival
pooled LGG+GBM, Etoposide exposure
0.0
0.2
0.4
0.6
0.8
1.0
0
500
1000
2000
3000

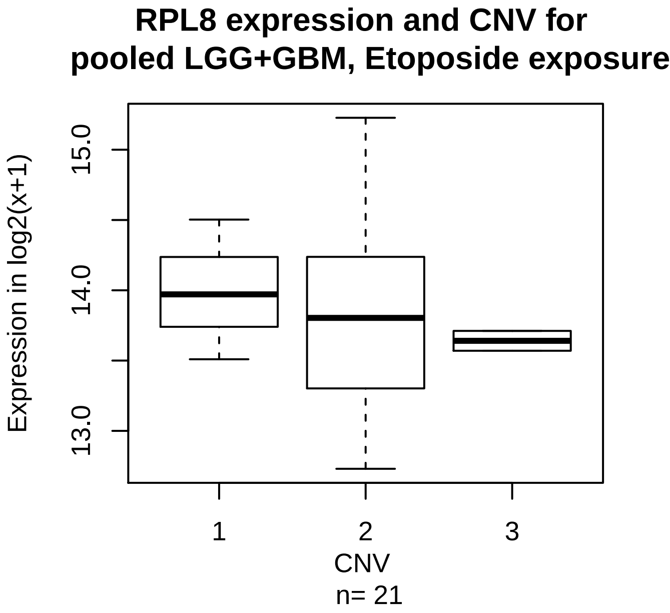
RPL8 expression and CNV for
pooled LGG+GBM, Etoposide exposure
Expression in log2(x+1)
13.0
14.0
15.0
1
2
3
CNV
n= 21

SCRT1 Kaplan–Meier survival pooled LGG+GBM

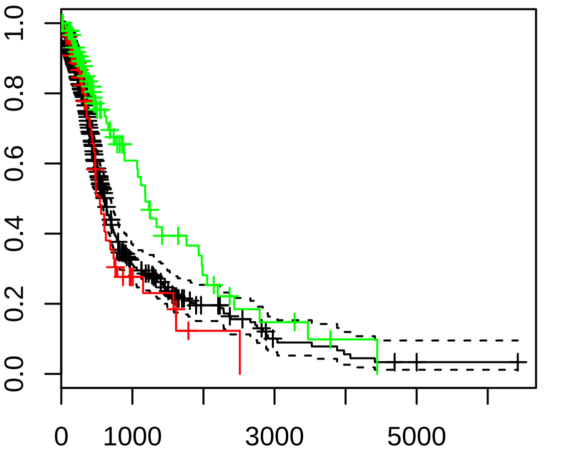

SCRT1 expression and CNV for pooled LGG+GBM

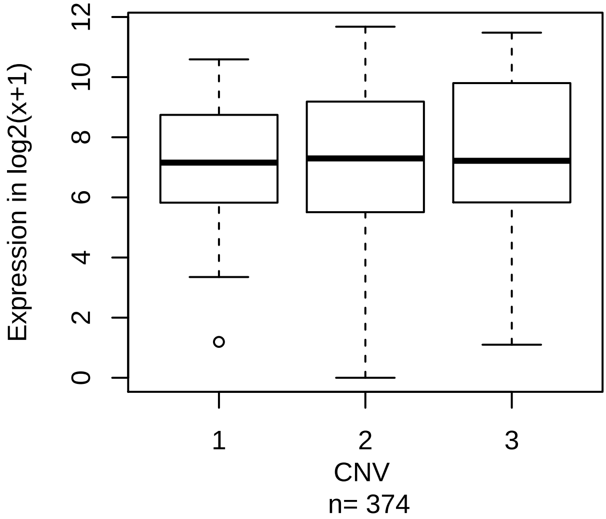

SCRT1 Kaplan–Meier survival pooled LGG+GBM, Etoposide exposure

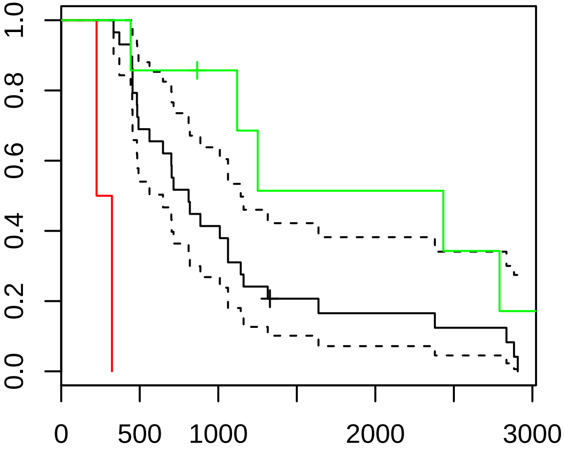

SCRT1 expression and CNV for pooled LGG+GBM, Etoposide exposure

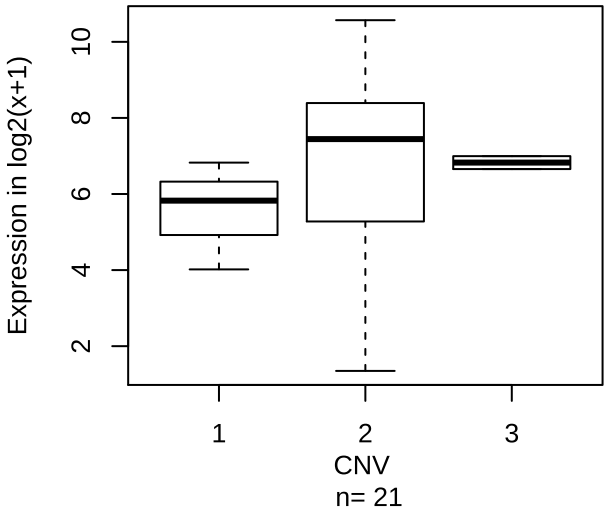

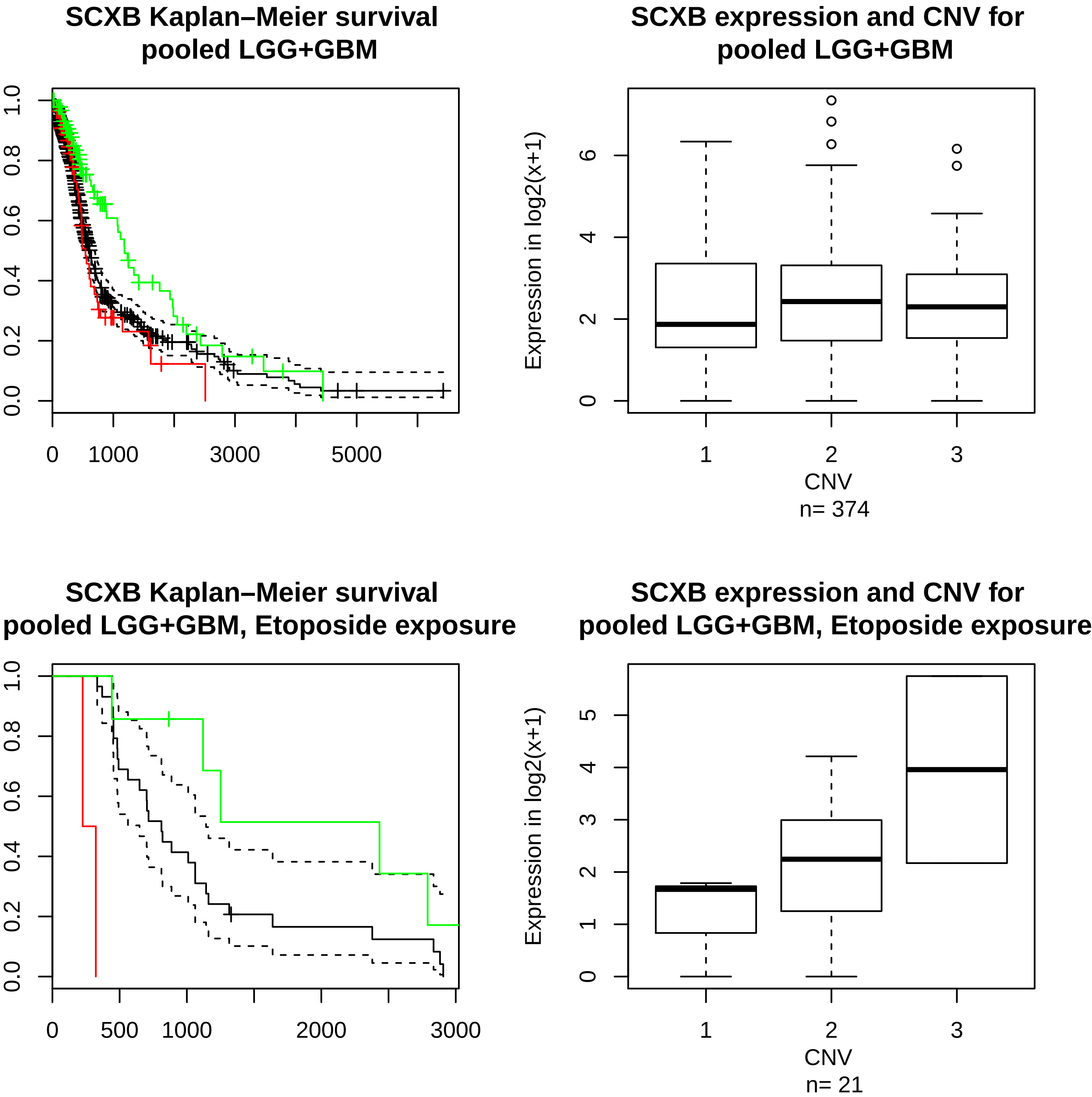
SCXB Kaplan–Meier survival
pooled LGG+GBM
0.0
0.2
0.4
0.6
0.8
1.0
0
1000
3000
5000
SCXB expression and CNV for
pooled LGG+GBM
Expression in log2(x+1)
0
2
4
6
1
2
3
CNV
n= 374
SCXB Kaplan–Meier survival
pooled LGG+GBM, Etoposide exposure
0.0
0.2
0.4
0.6
0.8
1.0
0
500
1000
2000
3000
SCXB expression and CNV for
pooled LGG+GBM, Etoposide exposure
Expression in log2(x+1)
0
1
2
3
4
5
1
2
3
CNV
n= 21

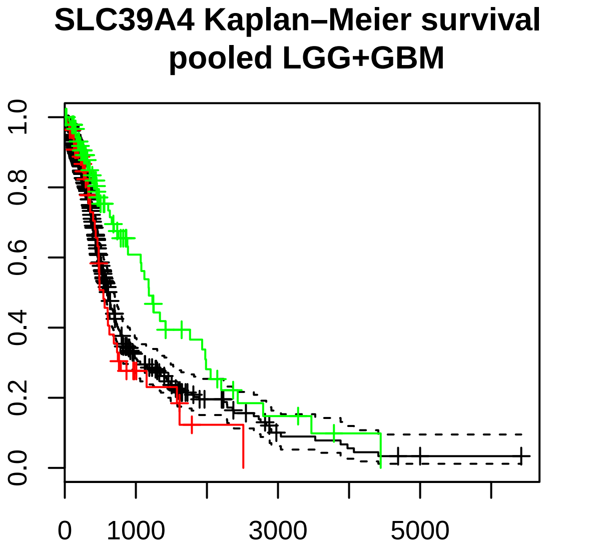
SLC39A4 Kaplan–Meier survival
pooled LGG+GBM
0.0
0.2
0.4
0.6
0.8
1.0
0
1000
3000
5000

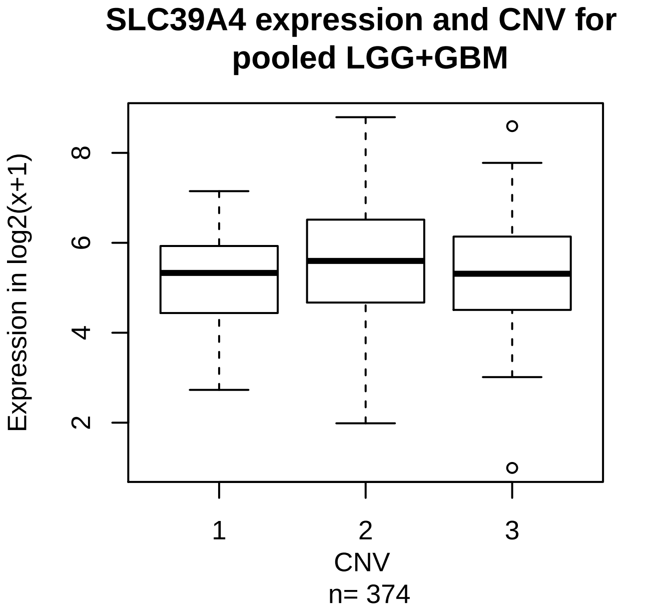
SLC39A4 expression and CNV for
pooled LGG+GBM
Expression in log2(x+1)
2
4
6
8
1
2
3
CNV
n= 374

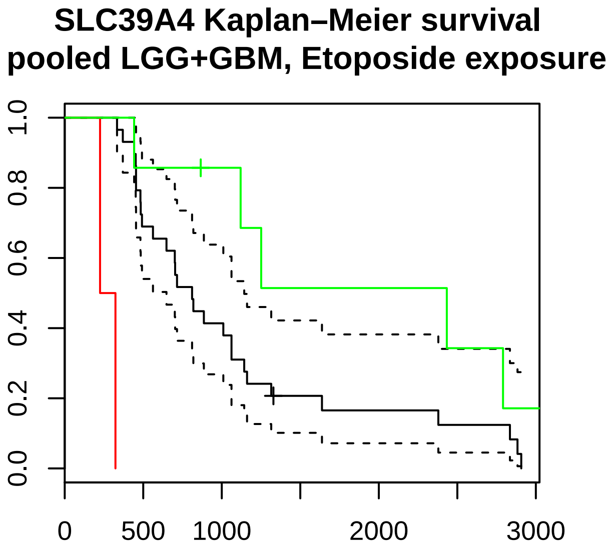
SLC39A4 Kaplan–Meier survival
pooled LGG+GBM, Etoposide exposure
0.0
0.2
0.4
0.6
0.8
1.0
0
500
1000
2000
3000

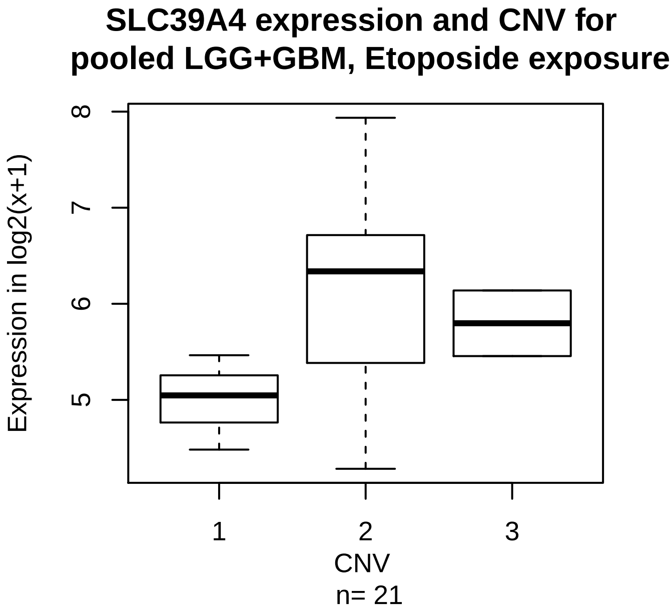
SLC39A4 expression and CNV for
pooled LGG+GBM, Etoposide exposure
Expression in log2(x+1)
5
6
7
8
1
2
3
CNV
n= 21

VPS28 Kaplan–Meier survival pooled LGG+GBM

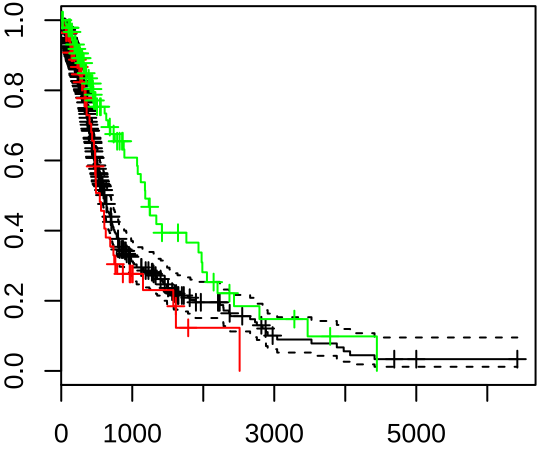

VPS28 expression and CNV for pooled LGG+GBM

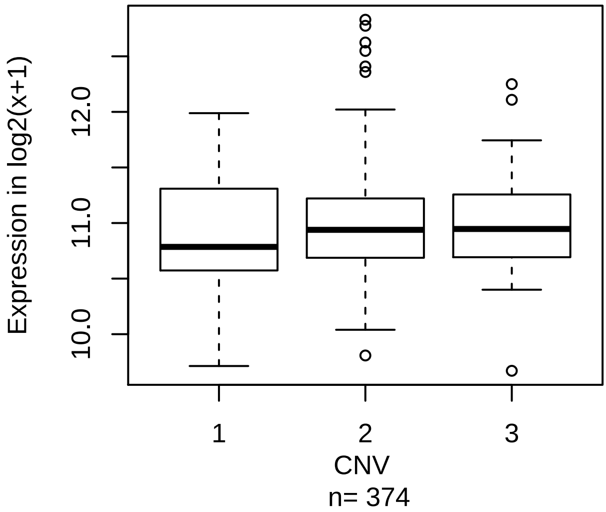

VPS28 Kaplan–Meier survival pooled LGG+GBM, Etoposide exposure

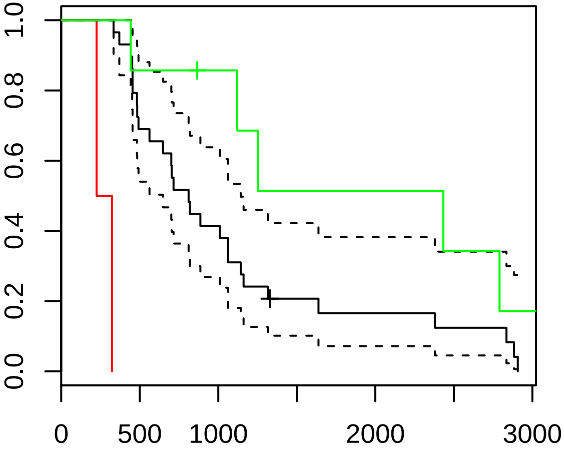

VPS28 expression and CNV for pooled LGG+GBM, Etoposide exposure

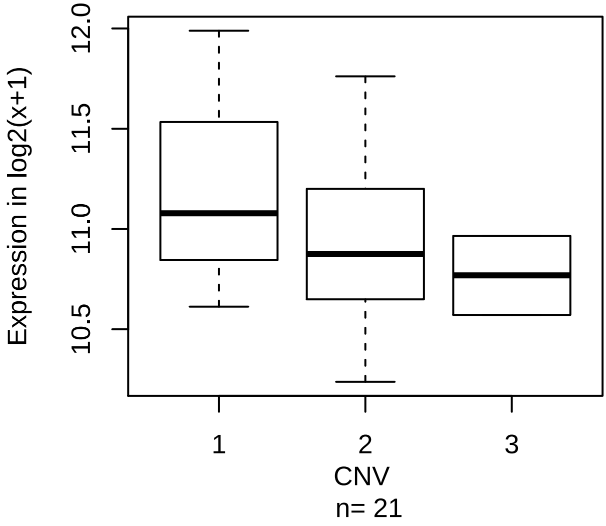

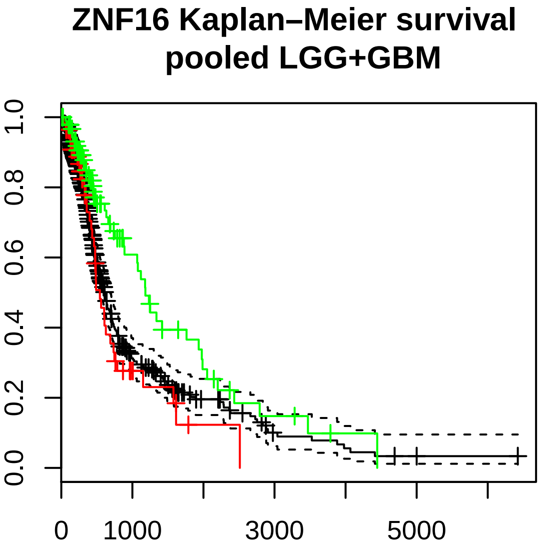
ZNF16 Kaplan–Meier survival
pooled LGG+GBM
0.0
0.2
0.4
0.6
0.8
1.0
0
1000
3000
5000

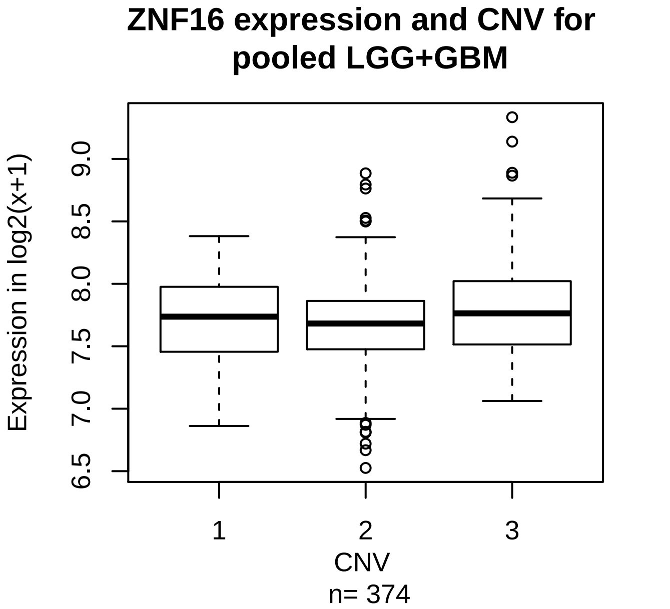
ZNF16 expression and CNV for
pooled LGG+GBM
Expression in log2(x+1)
6.5
7.0
7.5
8.0
8.5
9.0
1
2
3
CNV
n= 374

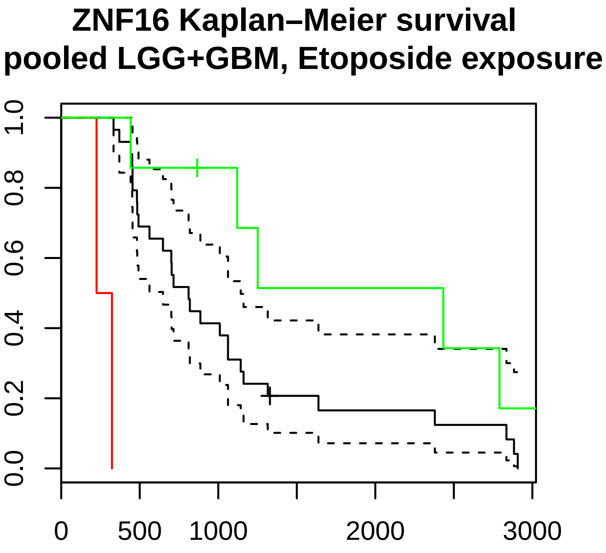
ZNF16 Kaplan–Meier survival
pooled LGG+GBM, Etoposide exposure
0.0
0.2
0.4
0.6
0.8
1.0
0
500
1000
2000
3000

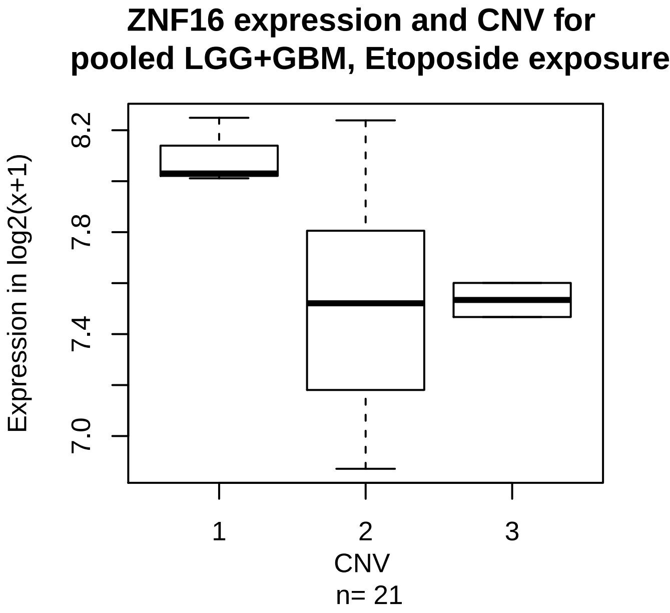
ZNF16 expression and CNV for
pooled LGG+GBM, Etoposide exposure
Expression in log2(x+1)
7.0
7.4
7.8
8.2
1
2
3
CNV
n= 21

ZNF250 Kaplan–Meier survival pooled LGG+GBM

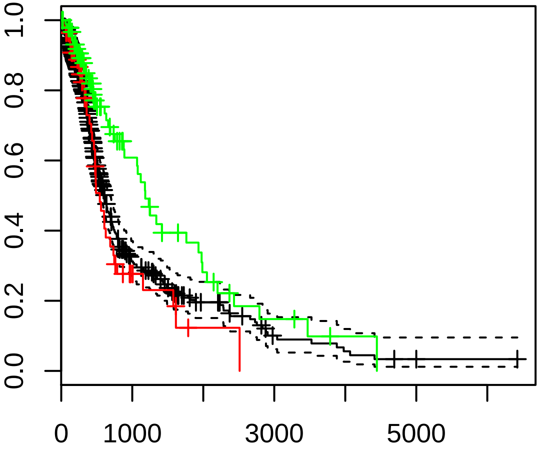

ZNF250 expression and CNV for pooled LGG+GBM

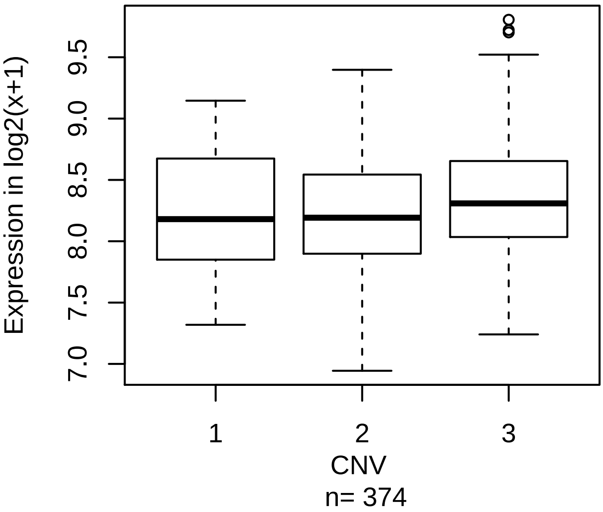

ZNF250 Kaplan–Meier survival pooled LGG+GBM, Etoposide exposure

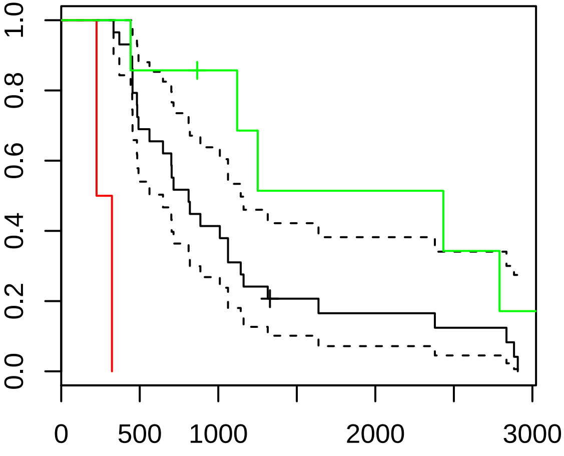

ZNF250 expression and CNV for pooled LGG+GBM, Etoposide exposure

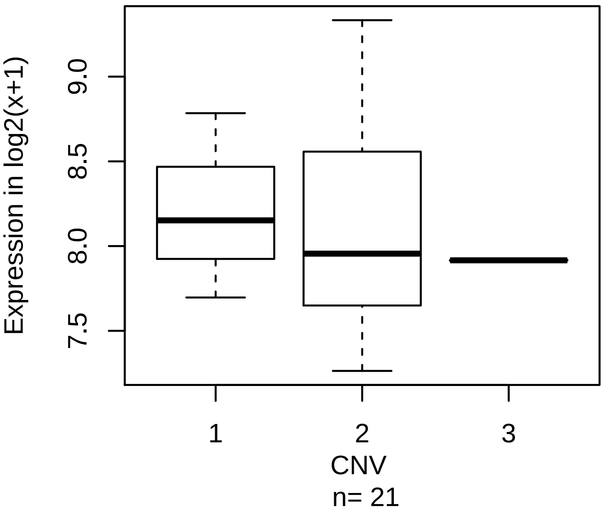

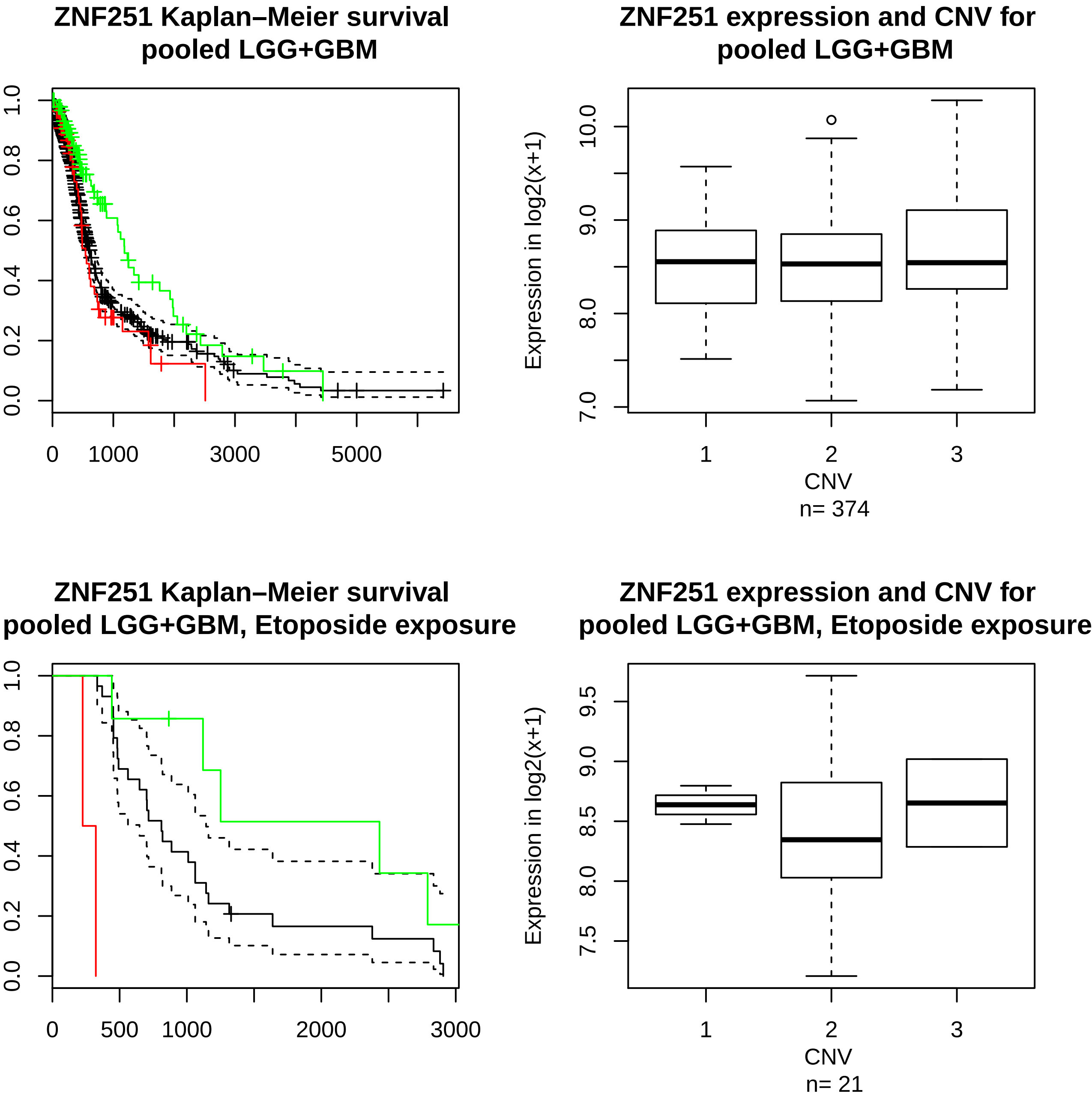
ZNF251 Kaplan–Meier survival
pooled LGG+GBM
0.0
0.2
0.4
0.6
0.8
1.0
0
1000
3000
5000
ZNF251 expression and CNV for
pooled LGG+GBM
Expression in log2(x+1)
7.0
8.0
9.0
10.0
1
2
3
CNV
n= 374
ZNF251 Kaplan–Meier survival
pooled LGG+GBM, Etoposide exposure
0.0
0.2
0.4
0.6
0.8
1.0
0
500
1000
2000
3000
ZNF251 expression and CNV for
pooled LGG+GBM, Etoposide exposure
Expression in log2(x+1)
7.5
8.0
8.5
9.0
9.5
1
2
3
CNV
n= 21

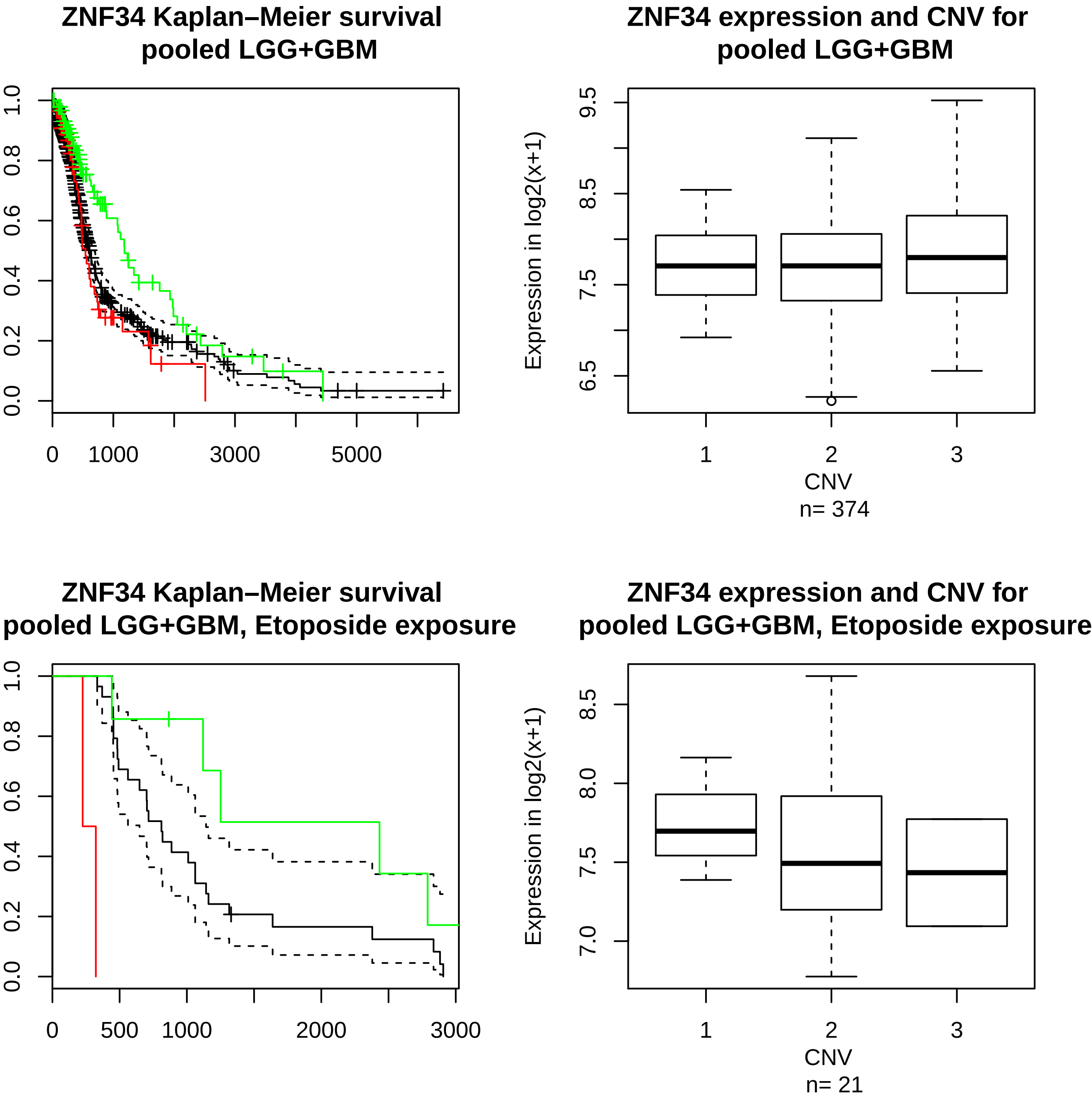
ZNF34 Kaplan–Meier survival
pooled LGG+GBM
0.0
0.2
0.4
0.6
0.8
1.0
0
1000
3000
5000
ZNF34 expression and CNV for
pooled LGG+GBM
Expression in log2(x+1)
6.5
7.5
8.5
9.5
1
2
3
CNV
n= 374
ZNF34 Kaplan–Meier survival
pooled LGG+GBM, Etoposide exposure
0.0
0.2
0.4
0.6
0.8
1.0
0
500
1000
2000
3000
ZNF34 expression and CNV for
pooled LGG+GBM, Etoposide exposure
Expression in log2(x+1)
7.0
7.5
8.0
8.5
1
2
3
CNV
n= 21

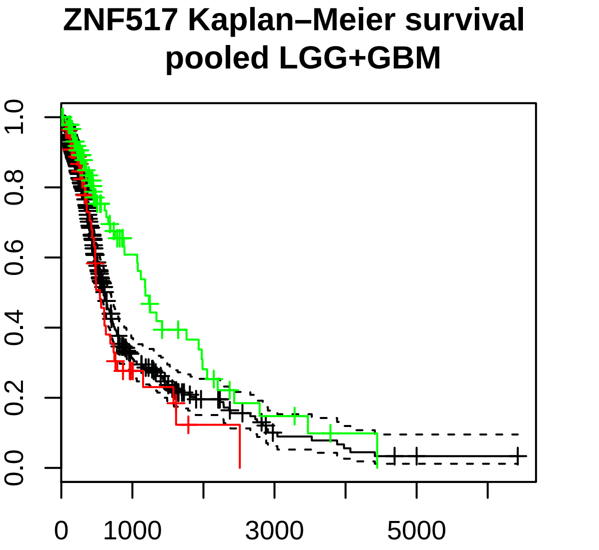
ZNF517 Kaplan–Meier survival
pooled LGG+GBM
0.0
0.2
0.4
0.6
0.8
1.0
0
1000
3000
5000

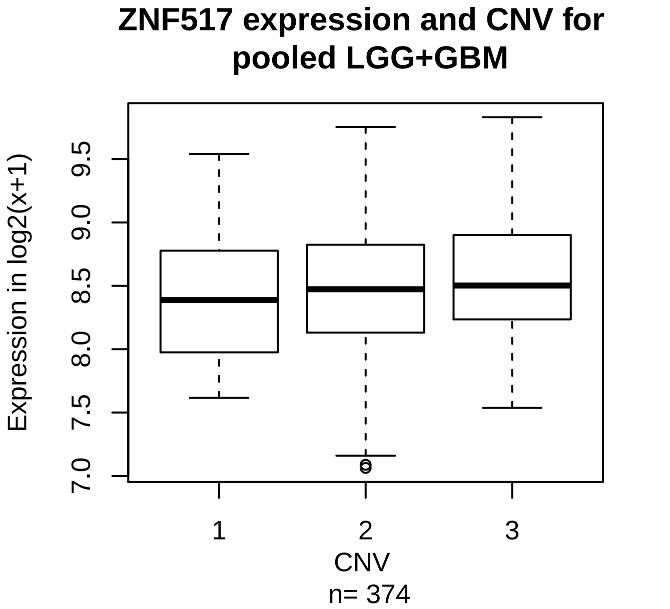
ZNF517 expression and CNV for
pooled LGG+GBM
Expression in log2(x+1)
7.0
7.5
8.0
8.5
9.0
9.5
1
2
3
CNV
n= 374

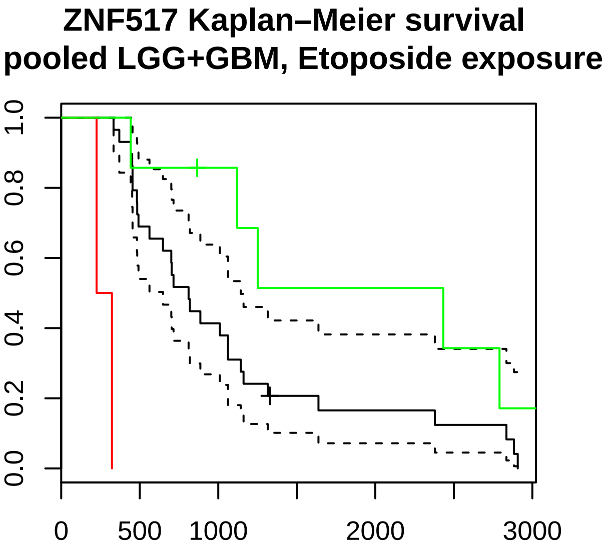
ZNF517 Kaplan–Meier survival
pooled LGG+GBM, Etoposide exposure
0.0
0.2
0.4
0.6
0.8
1.0
0
500
1000
2000
3000

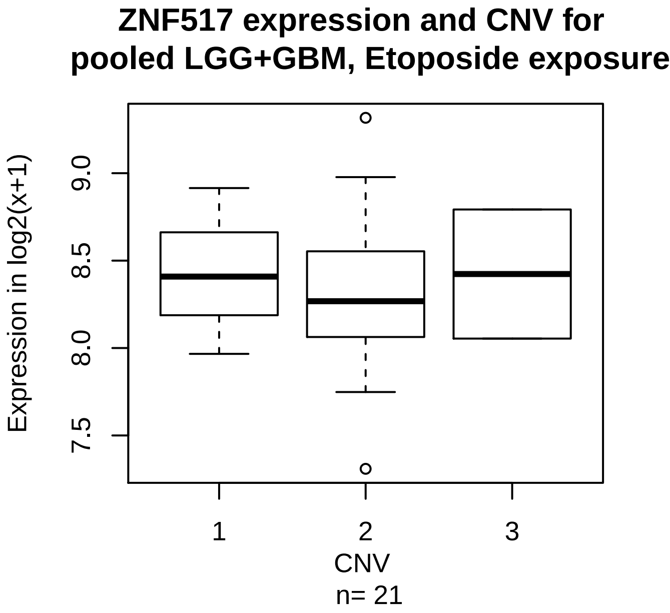
ZNF517 expression and CNV for
pooled LGG+GBM, Etoposide exposure
Expression in log2(x+1)
7.5
8.0
8.5
9.0
1
2
3
CNV
n= 21

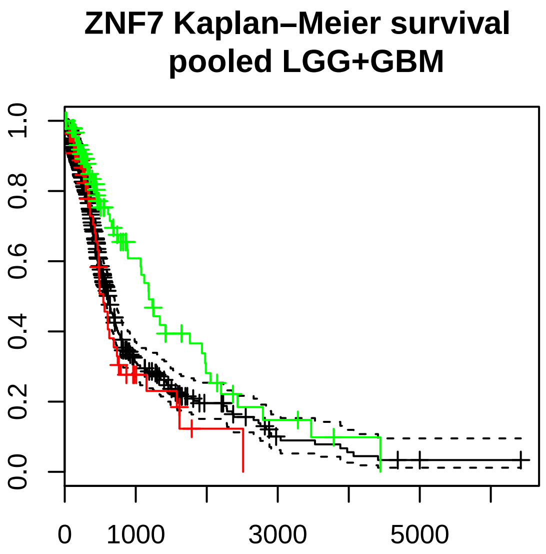

ZNF7 Kaplan–Meier survival
pooled LGG+GBM
0.0
0.2
0.4
0.6
0.8
1.0
0
1000
3000
5000

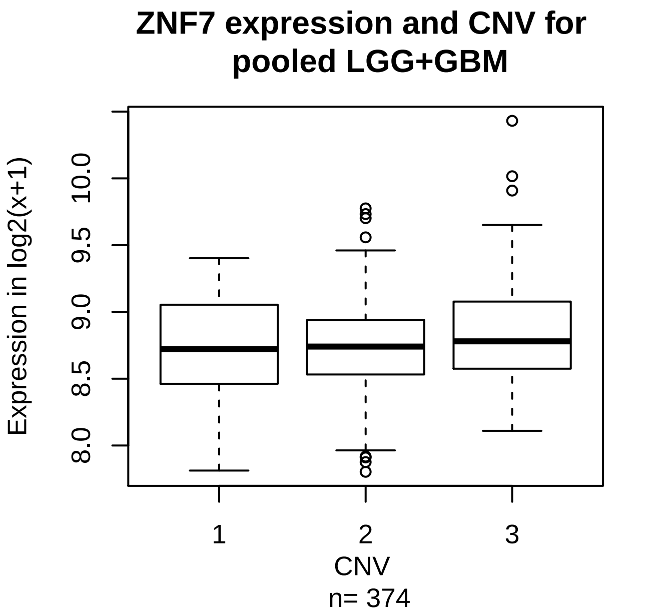

ZNF7 expression and CNV for
pooled LGG+GBM
Expression in log2(x+1)
8.0
8.5
9.0
9.5
10.0
1
2
3
CNV
n= 374

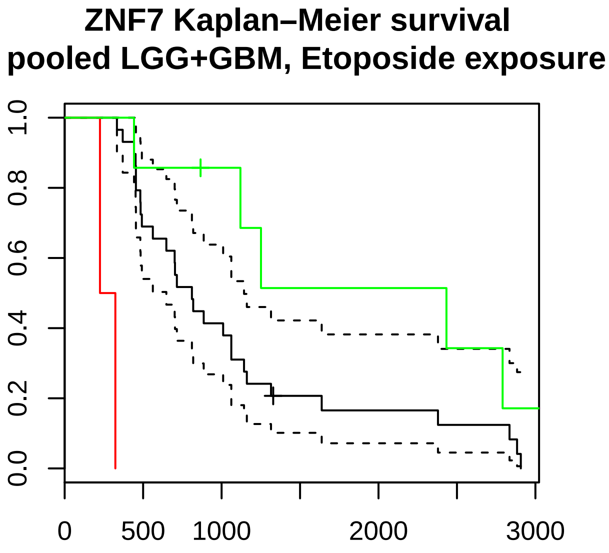

ZNF7 Kaplan–Meier survival
pooled LGG+GBM, Etoposide exposure
0.0
0.2
0.4
0.6
0.8
1.0
0
500
1000
2000
3000

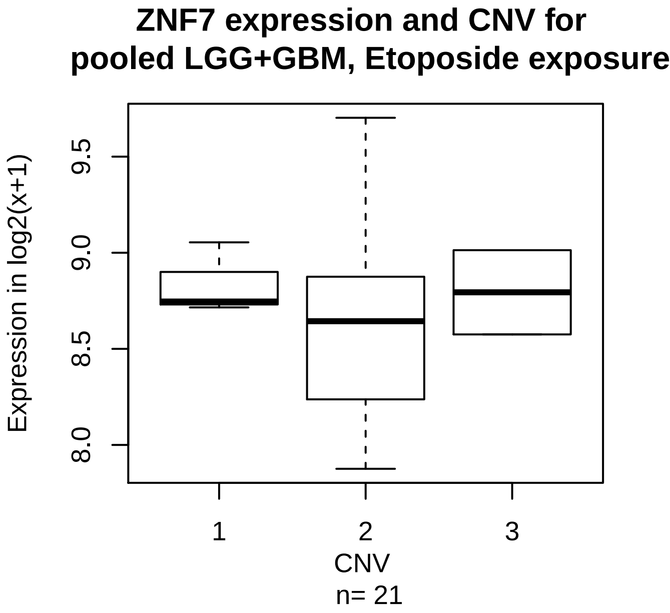

ZNF7 expression and CNV for
pooled LGG+GBM, Etoposide exposure
Expression in log2(x+1)
8.0
8.5
9.0
9.5
1
2
3
CNV
n= 21
