# Supplementary material for: Identification of gene-drug interactions that impact patient survival in TCGA
Source: BMC Bioinformatics. 2016 Oct 6;17:409. doi: 10.1186/s12859-016-1255-7 (PMC5053348; doi:10.1186/s12859-016-1255-7)
Supplement: Additional file 2: — This file contains additional figures, sample code, a gene list for table 2 and a drug name change list. (ZIP 1090 kb) [file 12859_2016_1255_MOESM2_ESM.zip › Supplementary Information.docx]

Supplementary Information

Supplemental Figures:

These figures show one gene per page, following the format of figures 2 and 3 in the main manuscript, for each gene found in the analysis.

Gene List:

A list of the genes found to be significant in the Etoposide drug exposure analysis, a supplementary list for table 2

Drug List:

The list of drugs used in the analysis. The left column designates the drug as called by the software. The right column shows each drug as listed in the data, grouped by our chosen call.

Sample R code:

Sample one contains an example of the analysis and filtering preformed. Sample two shows how our figures were generated.
